# Competitive formation of homocircuit [3]rotaxanes in synthetically useful yields in the bipyridine-mediated active template CuAAC reaction†
†Electronic supplementary information (ESI) available: Full experimental details and characterisation of all novel compounds. See DOI: 10.1039/c4sc03999h



**DOI:** 10.1039/c4sc03999h

**Published:** 2015-02-03

**Authors:** Edward A. Neal, Stephen M. Goldup

**Affiliations:** a School of Biological and Chemical Sciences , Queen Mary University of London , Mile End Road , London , E1 4NS , UK; b Department of Chemistry , University of Southampton , Highfield , Southampton , Hampshire SO17 1BJ , UK . Email: s.goldup@soton.ac.uk

## Abstract

We recently identified competitive formation of doubly interlocked [3]rotaxanes as the origin of the non-linear variation in yield of [2]rotaxane with macrocycle size in the bipyridine-mediated AT-CuAAC reaction. Selection of reaction conditions gave [2]rotaxanes in essentially quantitative yield in all cases and hard to access doubly threaded [3]rotaxanes in up to 50% yield in a single, four component coupling. Based on the effect of macrocycle structure on the reaction outcome we propose a detailed mechanism of [3]rotaxane formation.

## Abstract 2

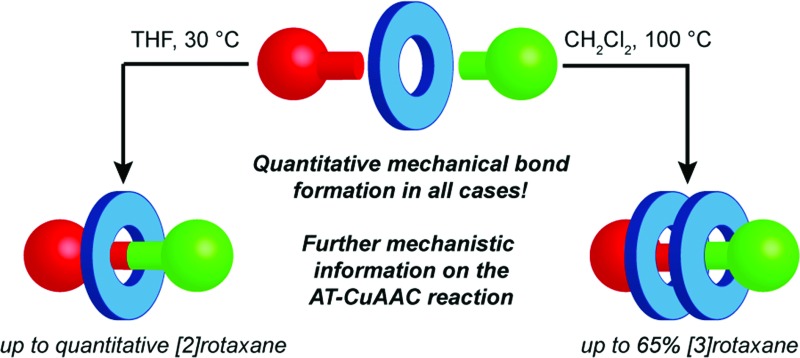
We demonstrate that, depending on reaction conditions, [2]rotaxanes are produced in essentially quantitative yield in the AT-CuAAC reaction regardless of macrocycle size, and hard to access doubly threaded [3]rotaxanes can be synthesised in up to 50% isolated yield in a four component coupling step.

## Introduction

Since Sauvage's first demonstration of a Cu^I^ template,^1^ the explosion of high yielding “passive template” methods for the synthesis of interlocked molecules has facilitated the synthesis of ever more complicated structures and their investigation in areas as diverse molecular electronics, drug delivery, sensors, materials, and as molecular machines.^2^ In 2006, Leigh and co-workers introduced the “active template” concept,^3^ in which the metal ion plays an active role in the final covalent bond-forming reaction that captures the interlocked structure, the first example of which was the active template Cu-mediated azide–alkyne cycloaddition^4^ reaction (AT-CuAAC) for the synthesis of [2]rotaxanes.^5^ This approach has since been applied to ever more complex interlocked molecules^6^ and molecular machines,^7^ as well as being extended to other bond-forming reactions.^8^

During investigations into the effect of macrocycle size in the AT-CuAAC reaction, we recently reported a small bipyridine macrocycle that allows access to functionalised [2]rotaxanes in excellent yield, further increasing the utility of this powerful methodology.^9^ These results have now been extended to the synthesis of a water-stable Cu^I^–triazolide,^10^ and the first scalable synthesis of mechanically chiral rotaxanes.^11^ However, at the time of our first report, the origin of the effect of macrocycle size was unclear, particularly as the yield of [2]rotaxane varied non-linearly with ring size.

Here we report that not only can the yield of [2]rotaxane for *all* macrocycle sizes be increased to >95% but that optimised conditions can produce novel [3]rotaxanes, challenging targets in their own right, in synthetically useful yields, uniting four components in a single step. Furthermore, comparison of the product distribution of macrocycles with and without a key benzylic ether unit allow us to propose detailed structures for the reactive intermediates leading to [3]rotaxane formation.

## Results and discussion

**Scheme 1:**
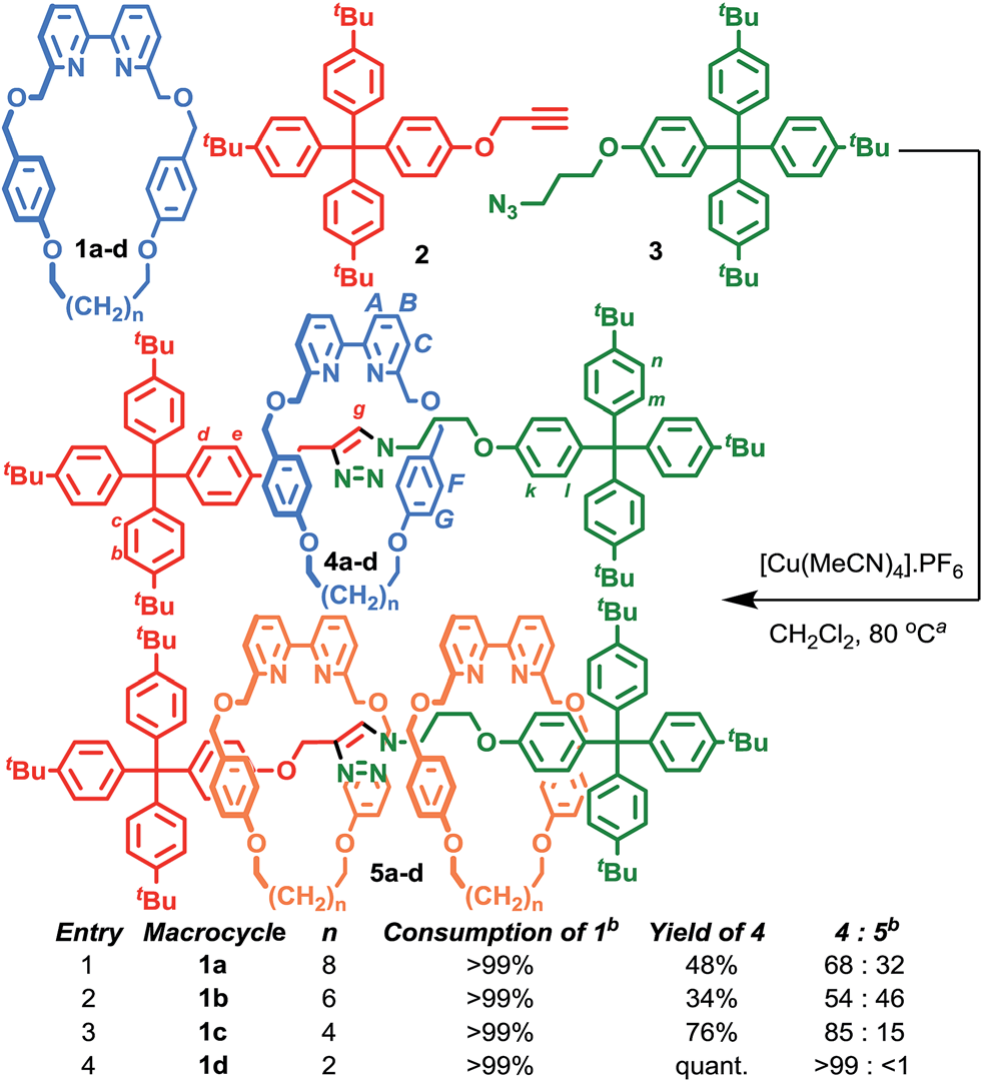
Effect of bipyridine macrocycle size on product distribution in the AT-CuAAC reaction. ^a^1 equiv. each **1**, **2**, **3**, 0.90 equiv. [Cu(MeCN)_4_]·PF_6_, 10 mM conc. of **1**, CH_2_Cl_2_, 80 °C, 72 h. ^b^Determined by ^1^H NMR analysis of the crude mixture.

We reinvestigated the AT-CuAAC reaction between macrocycles **1**, alkyne **2** and azide **3** to determine the origin of lower yields of [2]rotaxanes **4** with macrocycles **1a–c** (Scheme 1). Surprisingly, we found that although **1a** produced [2]rotaxane **4a** in just 48% yield, no **1a** was recovered; typically the balance of macrocycle not incorporated into the rotaxane is recovered unchanged in AT reactions.

**Fig. 1:**
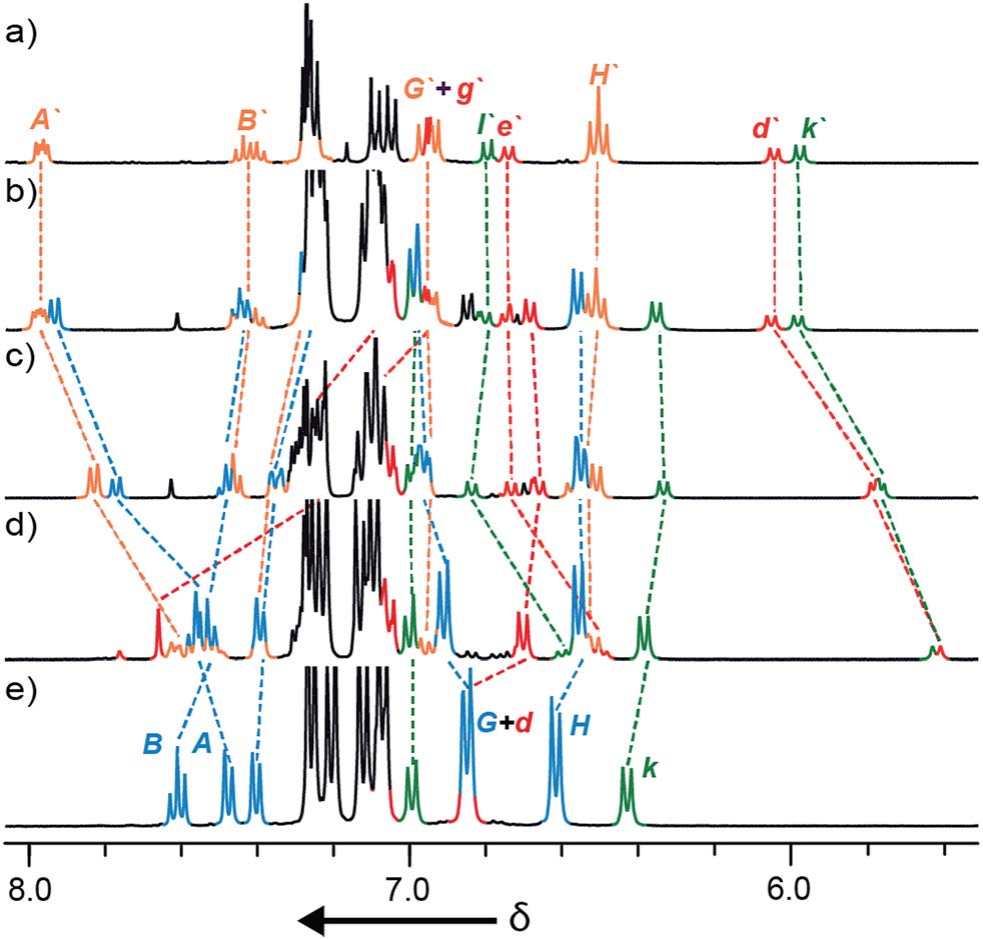
Partial ^1^H NMR (400 MHz, CDCl_3_, 300 K) of (a) pure [3]rotaxane **5a**; (b)–(e) the crude product mixtures obtained from the reactions of macrocycles **1a**, **1b**, **1c**, and **1d** respectively (conditions as shown in Scheme 1). Selected signals are assigned with labelling as shown in Scheme 1. Signals corresponding to [3]rotaxanes **5** are denoted by primes (note: although the two macrocycles in **5** are chemically non-equivalent their signals are poorly separated and they are given a single label here for clarity).

To our further surprise, on raising the polarity of the eluent during flash chromatography, a new interlocked product was unexpectedly isolated and subsequently identified as [3]rotaxane **5a**. Similarly, in the case of macrocycles **1b** and **1c** no non-interlocked macrocycle was recovered, with *all* of the starting materials accounted for by the formation of novel [3]rotaxanes **5b** and **5c** respectively. Only in the case of macrocycle **1d** was no [3]rotaxane observed; indeed **4d** is the only species observed in the ^1^H NMR spectrum of the crude reaction product (Fig. 1e). With pure [3]rotaxanes **5a–c** in hand, ^1^H NMR analysis of the crude reaction mixture revealed that this unexpected product accounted fully for the balance of the macrocycle added (Fig. 1). Thus, contrary to our original conclusion, the AT-CuAAC reaction with bipyridine macrocycles is *essentially 100% efficient* in the formation of the mechanical bond regardless of the size of the macrocycle, but the *product distribution* between [2]- and [3]rotaxane varies dramatically with macrocycle size.

Although Leigh and co-workers previously observed the formation of [3]rotaxanes during the AT-CuAAC reaction of monodentate pyridine macrocycles, this was thought not be possible in the case of bidentate macrocycles.^5*b*^ Furthermore, the yield of the [3]rotaxane product, based on the synthetically expensive macrocycle component, was extremely low (<7%; 33% with respect to the azide and alkyne components).^12^ Thus, having identified competitive [3]rotaxane formation in significant yield with a *single equivalent* of all reaction components as the origin of the variation in yield of [2]rotaxane with macrocycle size, we set out to investigate the effect of conditions and substrate on the product distribution, with a view to further optimising this efficient, versatile and reliable reaction.

### Effect of reaction conditions on the product distribution

We selected the AT-CuAAC reaction of macrocycle **1a** with half threads **2** and **3** to investigate the effect of conditions on the product distribution. The reactions were assessed by ^1^H NMR analysis of the crude reaction mixture after aqueous work up, as the signals for macrocycle, [2]- and [3]rotaxane, particularly those of the thread subunit (H_k_ of **5** and H_d_ and H_k_ of **6**) are clearly separated (Fig. 1).

We began our study by investigating the effect of reaction stoichiometry on the product distribution (Table 1), as this had previously been shown to have a large effect in the case of pyridine macrocycles, where high macrocycle–Cu ratios led to increased yields of [3]rotaxane.^5*b*^ Unexpectedly, in the case of macrocycle **1a** *decreasing* the equivalents of Cu^I^ from 0.9 (entry 1) led to an *increase* in the proportion of [2]rotaxane **4a** formed (entries 2–4) and *vice versa* (entry 5). Variation of the number of equivalents of the half threads (entries 6–8), led to small changes in the ratio of [2]- and [3]rotaxane formed, although a larger change was observed when both were used in excess (entry 8). Conducting the standard reaction at higher concentration (entry 9) did not significantly affect the reaction outcome.

**Table 1:** The effect of stoichiometry on the AT-CuAAC reaction of **1a**

Entry	Cu^I^ : **1a** : **2** : **3**	*t*/h	Conv. **1a** ^*c*^	**4a** : **5a** ^*c*^
1^*a*^	0.90 : 1 : 1 : 1	6	>99%	65 : 35
2^*a*^	0.50 : 1 : 1 : 1	51	>99%	85 : 15
3^*a*^	0.18 : 1 : 1 : 1	290	92%	97 : 3
4^*b*^	0.90 : 5 : 5 : 5	6	>99%	97 : 3
5^*a*^	0.96 : 1 : 1 : 1	6	>99%	62 : 38
6^*a*^	0.90 : 1 : 5 : 1	6	>99%	63 : 37
7^*a*^	0.90 : 1 : 1 : 5	6	>99%	65 : 35
8^*a*^	0.90 : 1 : 5 : 5	6	>99%	76 : 24
9^*b*^	4.50 : 5 : 5 : 5	6	>99%	68 : 32

^*a*^80 °C in CH_2_Cl_2_, 10 mM conc. of **1a**.

^*b*^50 mM concentration of **1a**.

^*c*^Determined by ^1^H NMR analysis of the crude mixture.

The AT-CuAAC reaction tolerates a wide range of solvents with excellent conversion of **1a** to interlocked products observed in all solvents investigated (Table 2). The slightly reduced selectivity for interlocked products in the case of the strongly coordinating solvent NMP (entry 7) can be attributed to a small proportion of the catalytically competent Cu^I^ remaining bound to solvent rather than **1a**. However, the product distribution shows a strong solvent dependency with, in general, less coordinating solvents (entries 1–4) favouring [3]rotaxane relative to more coordinating solvents (entries 5–7). In this regard PhMe (entry 4) is an outlier, being relatively non-coordinating but also producing less [3]rotaxane than CH_2_Cl_2_, EtOAc or CHCl_3_ (entries 1–3). However, it should be noted that the solubility of the [**1a**·Cu]·PF_6_ complex is poor in PhMe.

**Table 2:** The effect of solvent on the AT-CuAAC reaction of **1a** ^*a*^

Entry	Solvent	Conv. **1a** ^*b*^	**4a** : **5a** ^*b*^
1	CH_2_Cl_2_	>99%	65 : 35
2	CHCl_3_	>99%	64 : 36
3	EtOAc	>99%	65 : 35
4	PhMe	>99%	76 : 24
5	DME	>99%	72 : 28
6	THF	>99%	86 : 14
7	NMP	91%	94 : 6

^*a*^1 equiv. each **1a**, **2**, **3**, 0.90 equiv. [Cu(MeCN)_4_]·PF_6_, 10 mM conc. of **1a**, 80 °C.

^*b*^Determined by ^1^H NMR analysis of the crude mixture.

The reaction outcome shows a large dependence on temperature with a maximum in [3]rotaxane formation being observed between 60 and 100 °C (Table 3, entries 1–4). Reducing the temperature below 60 °C led to increased quantities of [2]rotaxane formation (entries 5–7). Although lowering the reaction temperature also decreased the reaction rate significantly this could be overcome by addition of base (N^*i*^Pr_2_Et), which we have previously shown dramatically enhances the rate of the AT-CuAAC reaction.^10^ The addition of base does not alter the product distribution (entry 8).

**Table 3:** The effect of temperature on the AT-CuAAC reaction of **1a** ^*a*^

Entry	*T*/°C	*t*/h	Conv. **1a** ^*b*^	**4a** : **5a** ^*b*^
1	100	6	>99%	66 : 34
2	80	6	>99%	67 : 33
3	70	8	>99%	65 : 35
4	60	26	98%	67 : 33
5	50	54	96%	70 : 30
6	40	116	89%	81 : 19
7	30	116	80%	86 : 14
8	30	3	>99%	86 : 14

^*a*^1 equiv. each **1**, **2**, **3**, 0.90 equiv. [Cu(MeCN)_4_]·PF_6_, 10 mM conc. of **1a**, CH_2_Cl_2_.

^*b*^Determined by ^1^H NMR analysis of the crude mixture.

In summary, the formation of [3]rotaxane is favoured by (i) a macrocycle : Cu^I^ ratio close to unity; (ii) less coordinating solvents; and (iii) higher reaction temperatures. Combining these results allowed us to design new reaction conditions, A and B, that maximised the formation [2]rotaxane and [3]rotaxane respectively (Table 4).^13^ Using conditions A, all [2]rotaxanes **4** were isolated in excellent yield (>94%) with a reaction time of 6 h at rt, a significant improvement over our previously published conditions (Table 4, entries 1–4). Alternatively, adopting conditions B allowed the formation of [3]rotaxanes **5** in good yield; 53% and 65% for macrocycles **1a** and **1b** respectively (49% and 50% isolated yields; entries 5–6). However, even under these optimised conditions, **5d** was not observed (entry 8).

**Table 4:** Optimised syntheses of rotaxanes **4** and **5**

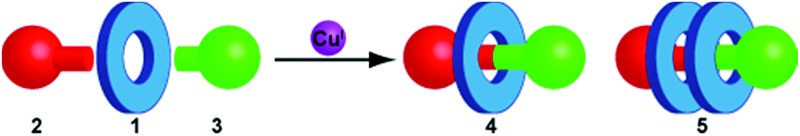				
Entry	Macrocycle	**4** : **5** ^*c*^	Isolated product	Yield
**Conditions A: THF, 0.10 equiv. Cu** ^**I**^ **, N** ^***i***^ **Pr**_**2**_ **Et, rt, 6 h** ^*a*^				
1	**1a**	99 : 1	**4a**	99^*c*^% (95%)^*d*^
2	**1b**	99 : 1	**4b**	99^*c*^% (94%)^*d*^
3	**1c**	>99 : <1	**4c**	>99^*c*^% (99%)^*d*^
4^*e*^	**1d**	>99 : <1	**4d**	>99^*c*^% (99%)^*d*^

**Conditions B: CH**_**2**_ **Cl**_**2**_ **, 0.96 equiv. Cu** ^**I**^ **, 100 °C (μW), 20 min** ^*b*^				
5	**1a**	64 : 36	**5a**	53^*c*^% (49%)^*d*^
6	**1b**	52 : 48	**5b**	65^*c*^% (50%)^*d*^
7	**1c**	87 : 13	**5c**	23^*c*^% (19%)^*d*^
8^*f*^	**1d**	>99 : <1	**4d**	>99^*c*^% (quant.^*d*^)

^*a*^1 equiv. **1**, 1.2 equiv. **2** and **3**, 50 mM conc. of **1**.

^*b*^1 equiv. **1**, 1.2 equiv. **2** and **3**, 10 mM conc. of **1**.

^*c*^Determined by ^1^H NMR analysis of the crude mixture.

^*d*^Isolated yield.

^*e*^20 h.

^*f*^2 h.

### The effect of thread structure on [3]rotaxane formation

Having identified conditions that favour [3]rotaxane formation, and given that doubly threaded [3]rotaxanes are challenging synthetic targets in their own right,^14^ we turned our attention to the effect of substrate structure on the reaction outcome with a view to determining the generality of the procedure. Pleasingly, elongation of the linker between the azide and stopper moieties (Table 5, entry 1) did not lead to a significant change in the product distribution compared with the reaction between alkyne **2** and azide **3** suggesting that there is relative freedom in the choice of the azide component. Conversely, lengthening the spacer between the tritylphenoxy head group and the acetylene moiety from a single methylene to a (CH_2_)_4_ (entry 2) or (CH_2_)_9_ (entry 3) chain revealed that longer spacers lead to decreased quantities of [3]rotaxane. Thus, it appears that the structure of the azide component is largely irrelevant to the reaction outcome whereas the acetylene component plays a key role in [3]rotaxane formation, with longer linkers between the alkyne and stopper units, counter-intuitively, favouring less sterically demanding [2]rotaxane. In keeping with these results, when azide **6** was employed with alkyne **7**, the ratio of interlocked products was largely unchanged from the reaction with the same acetylene and shorter azide **3** (entry 4). Finally, no [3]rotaxane product was formed in the reaction between **1d**, azide **6** and alkyne **7**, affording complete conversion to [2]rotaxane, despite the potential of the longer thread length to better accommodate a second macrocyclic component (entry 5).

**Table 5:** Effect of half thread structure on product distribution in the AT-CuAAC reaction^*a*^

Entry	Macrocycle	Alkyne	Azide	[2] : [3] rotaxane^*b*^	Yield of [3]rotaxane
1	**1a**	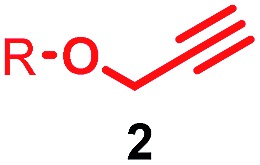	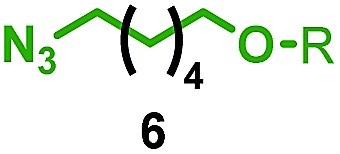	65 : 35	52^*b*^% (48%)^*c*^
2	**1a**	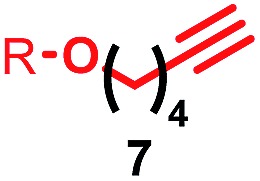	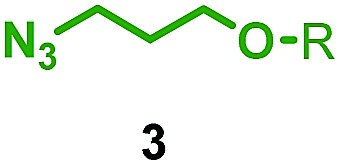	71 : 29	45^*b*^% (32%)^*c*^
3	**1a**	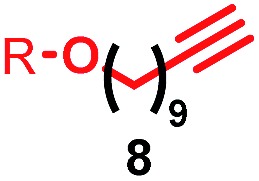	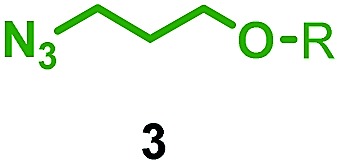	76 : 24	39^*b*^% (34%)^*c*^
4	**1a**	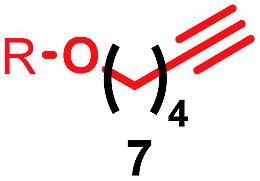	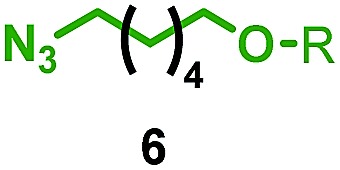	76 : 24	39^*b*^% (35%)^*c*^
5	**1d**	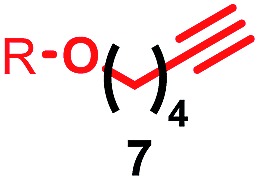	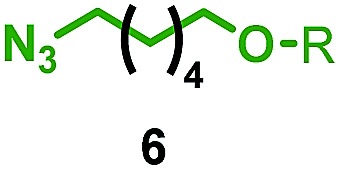	>99 : <1	—

^*a*^1 equiv. macrocycle, 1.2 equiv. alkyne and azide, 0.96 equiv. [Cu(MeCN)_4_]·PF_6_, 10 mM of macrocycle, 100 °C (μW), 2 h. R = 4-(4^*t*^BuC_6_H_4_)_3_CC_6_H_4_–.

^*b*^Determined by ^1^H NMR analysis of the crude reaction mixture.

^*c*^Isolated yield. See ESI for further information.

### The effect of macrocycle structure – a mechanistic clue

**Scheme 2:**
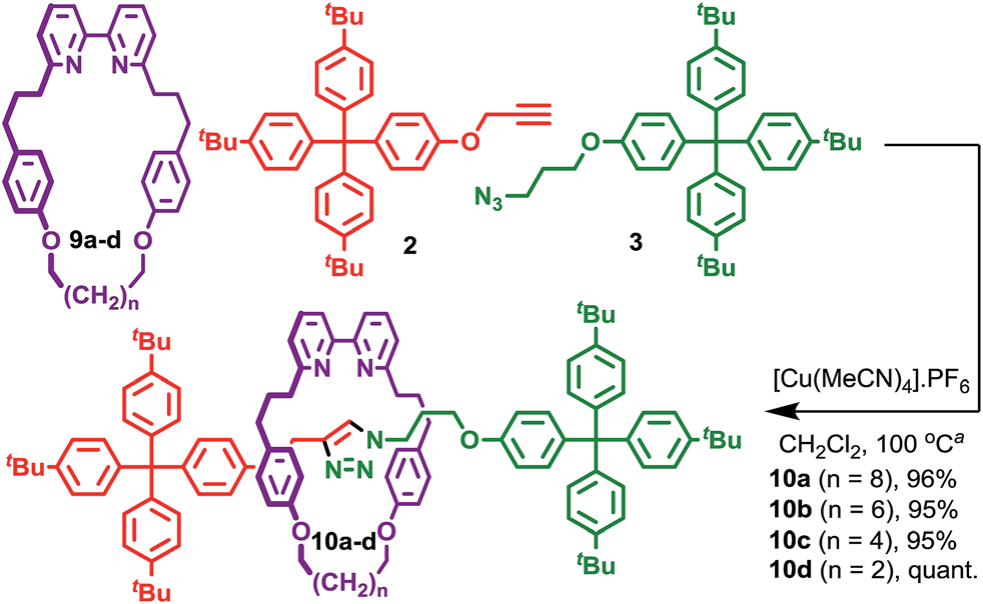
Selective synthesis of [2]rotaxanes **10** with macrocycles **9**. ^a^1 equiv. **9**, 1.2 equiv. each **2** and **3**, 0.96 equiv. [Cu(MeCN)_4_]·PF_6_, 50 mM conc. of **9**, CH_2_Cl_2_ 100 °C (μW), 15 min. The ratio of [2] : [3]rotaxane was >98 : <2 in all cases.

Having established that [3]rotaxane formation is relatively general with respect to the thread structure, we returned to the structure of the macrocycle in an effort to understand the origin on the doubly interlocked product. Working on the hypothesis that the benzylic ether oxygens in macrocycles **1** may play a role in assembling the key reactive intermediate, and that this is the interaction that coordinating solvents serve to disrupt, we investigated the AT-CuAAC reactions of macrocycles **9** in which this moiety was replaced with a simple methylene. Sure enough, macrocycles **9** give rise to [2]rotaxanes **10** in excellent yield with only traces (<2%) of the corresponding [3]rotaxane observed, even under conditions previously found to favour [3]rotaxane formation with macrocycles **1** (Scheme 2). The reactions of macrocycle **9** were slower than macrocycle **1**, requiring a higher concentration to reach completion in a comparable time. No significant variation in product distribution with macrocycle size was observed; all [2]rotaxanes **10** were isolated in excellent yield (>95%). This observation, combined with the effect of coordinating solvents on the product distribution for macrocycles **1** strongly supports the proposal that the benzylic ether units help assemble the key reactive species *en route* to the doubly interlocked product.

### A mechanistic model of [3]rotaxane formation

**Scheme 3:**
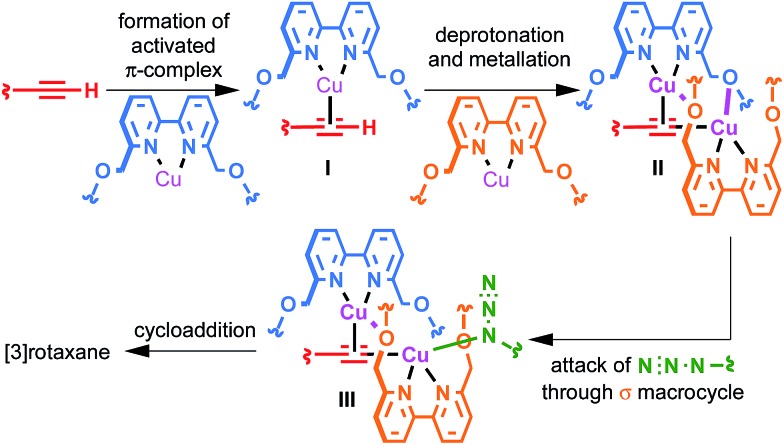
Proposed mechanism of [3]rotaxane formation.

**Fig. 2:**
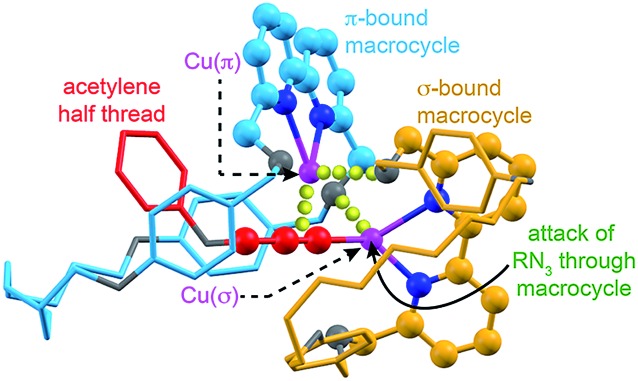
Simple computational model (MM2)^16^ of proposed intermediate **II** indicating the attack of the azide component through the cavity of the σ–bound macrocycle. O, N and Cu atoms shown in grey, dark blue, and pink respectively. Macrocycles coloured light blue (π-bound) and orange (σ-bound) for clarity. Bonding interactions between Cu(π) and the acetylenic triple bond; and O of the σ-bound macrocycle and Cu(σ) and the O of the π-bound macrocycle are represented by yellow spheres. The acetylene component has been truncated for clarity.

The surprisingly large effect of the benzylic ether oxygens on the outcome of the AT-CuAAC reaction suggests that they play a key role in stabilising the intermediate leading to [3]rotaxane formation. By analogy to Leigh's proposed pathway in the case of monopyridine macrocycles,^5*b*^ and in keeping with previous work on the mechanism of the CuAAC reaction,^15^ this leads us tentatively to propose a mechanism for [3]rotaxane formation (Scheme 3). Initial π-coordination of the acetylene by one equivalent of macrocycle–Cu complex leads to threaded intermediate **I**, lowering the p*K*_a_ of the acetylenic C–H. Subsequent deprotonation and trapping with a second equivalent of macrocycle-bound Cu leads to key intermediate **II** that is stabilised by reciprocal Cu–O interactions bridging the two macrocycles. Finally, coordination of the azide component through the cavity of the σ-bound macrocycle, displacing the interaction between the σ-bound Cu and the benzylic ether unit of the π-bound macrocycle leads to doubly threaded intermediate **III** that collapses *via* a 1,3-dipolar cycloaddition to give the observed [3]rotaxane product. A simple computational model was constructed (Fig. 2), demonstrating the shape of intermediate **II** and the open face of the σ-bound macrocycle through which the azide can approach.

This mechanistic scheme accounts for many of our observations: (i) the effect of coordinating solvents is explained by their tendency to competitively disrupt the key Cu–O interaction, destabilising **II**; (ii) the failure of smaller macrocycles to produce significant quantities of [3]rotaxane is accounted for by increased steric interactions in **I** that prevent formation of a threaded π-complex in the case of the smallest macrocycles employed; (iii) the slight reduction in [3]rotaxane yield with the largest macrocycle **1a** compared with **1b** may be due to the ability of larger macrocycles to adopt an unthreaded “side saddle” coordination mode in intermediate **II** leading to [2]rotaxane; (iv) similarly, the counterintuitive tendency of less sterically hindered acetylenes to produce more [2]rotaxane is rationalised by their added flexibility, facilitating the side saddle coordination of the π-bound macrocycle; (v) the insensitivity of the reaction to the structure of the azide component arises naturally from this model as the doubly threaded structure is assembled prior to azide coordination.

The mechanistic origin of the [2]rotaxane product is less clear. Leigh and co-workers previously proposed that, in the case of pyridine macrocycles, [2]rotaxane is formed by intermediates in which π-activation of the acetylene is achieved by a Cu centre not coordinated by a macrocyclic ligand, and the addition of excess macrocycle leading to enhanced yields of both [2]- and [3]rotaxanes was provided as evidence.^5*b*^ However, in the case of bipyridine macrocycles the opposite trend was observed: high Cu–macrocycle loadings led to enhanced [3]rotaxane formation. This suggests that, in keeping with the stronger chelating nature of the bipyridine ligand, such “free” Cu is not involved in the reaction.

**Scheme 4:**
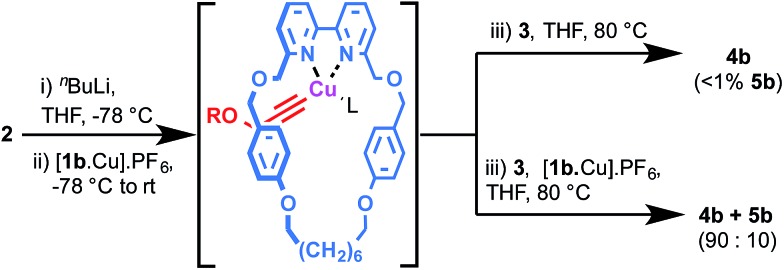
Effect of alkyne lithiation on rotaxane product distribution (see ESI† for full details). In both cases quantitative (>99%) conversion of **2** and **3** to interlocked products was observed. R = (4-^*t*^BuC_6_H_4_)_3_CC_6_H_4_–. L = THF or MeCN.

Although [2]rotaxane could potentially arise by side saddle coordination of the macrocycle in intermediate **II**, based on the effect of Cu loading it seems likely that a mono-metallic pathway is also in operation. This is supported by two simple experiments under strongly basic conditions (Scheme 3). Lithiation of acetylene **2** followed by trapping with [**1b**·Cu]·PF_6_ and then addition of azide **3** led almost exclusively to the expected [2]rotaxane, suggesting that the σ-bound macrocycle–Cu–acetylide complex formed quantitatively by transmetallation from the lithium acetylide is unable to rearrange to give a bimetallic intermediate such as **II** and, therefore, can only progress directly to [2]rotaxane. Conversely, repeating the same reaction with an additional equivalent [**1b**·Cu]·PF_6_ added along with azide **3** (Scheme 4b) led to partial recovery of [3]rotaxane formation, presumably as additional macrocycle–Cu^I^ complex allows the system to rearrange *via* ligand transfer to produce intermediates of the form of **II**.

## Conclusions

In conclusion, we have demonstrated that, through optimisation of the reaction conditions, [2]rotaxanes can be produced in essentially quantitative yields in the AT-CuAAC reaction with all macrocycles investigated. Furthermore, by performing the reaction at higher temperature and Cu loading with larger macrocycles **1a** and **1b**, we have demonstrated the synthesis of seven novel doubly interlocked [3]rotaxanes in up to 65% yield (50% isolated yield), through a convergent four component coupling, the first time that such complex structures have been obtained in high yield in an active template reaction. Finally, we have presented a relatively detailed structure for the key reactive intermediate leading to [3]rotaxanes in the bipyridine-mediated AT-CuAAC reaction that provides an explanation for the effect of macrocycle and thread structure on the product distribution between [2]- and [3]rotaxane.
