# Supplementary material for: Competitive formation of homocircuit [3]rotaxanes in synthetically useful yields in the bipyridine-mediated active template CuAAC reaction
Source: Chem Sci. 2015 Feb 3;6(4):2398–404. doi: 10.1039/c4sc03999h (PMC5645920; doi:10.1039/c4sc03999h)
Supplement: Supplementary file 1 [file SC-006-C4SC03999H-s001.pdf]

Electronic Supplementary Material (ESI) for Chemical Science.
This journal is © The Royal Society of Chemistry 2015

Supporting Information

Competitive Formation of Homocircuit [3]Rotaxanes in Synthetically Useful Yields in the Bipyridine-Mediated Active Template CuAAC Reaction

Edward A. Neal[a] and Stephen M. Goldup*[b]

[a]School of Biological and Chemical Sciences, Queen Mary, University of London, Mile End, London, UK E1 4NS.
[b]School of Chemistry, University of Southampton, University Road, Southampton, UK SO17 1BJ.

Contents

General Experimental Information **S-3**

Experimental Procedures **S-3**

Lithiation Experiments (Scheme 4) **S-30**

NMR Data for All Novel Compounds **S-31**

References **S-141**

General Experimental Information

Unless otherwise stated, all reagents were purchased from commercial sources and used without further purification. All reactions were carried out under an atmosphere of N_2 using anhydrous solvents unless otherwise stated. Anhydrous THF, toluene, DMF, diethyl ether and methylene chloride were either obtained by passing the solvent through an activated alumina column on an MBRAUN MB SPS-800 solvent purification system or purchased from Acros, otherwise solvents were used without further purification. Petrol refers to the fraction of petroleum ether boiling in the range 40-60 °C. Microwave-assisted reactions were undertaken on a CEM Discover SP reactor. Flash column chromatography was performed using a Biotage Isolera 4 automated chromatography system, employing Biotage ZIP or SNAP KP-SIL cartridges. Analytical NMR spectra were recorded on Bruker AV400, AMX400 or AV600 instruments, at a constant temperature of 300 K, by the NMR service at Queen Mary, University of London. ^{13}C-NMR were typically recorded as DEPT-Q135 experiments (phased spectrum including quaternary Cs). Chemical shifts are reported in parts per million from low to high field and referenced to residual solvent. Coupling constants (J) are reported in Hertz (Hz). Standard abbreviations indicating multiplicity were used as follows: m = multiplet, quint. = quintet, q = quartet, t = triplet, d = doublet, s = singlet, br = broad. All melting points were determined using a Sanyo Gallenkamp apparatus and are uncorrected. Low resolution mass spectrometry was carried out by the mass spectrometry service at Queen Mary, University of London using an Agilent SL Ion Trap MSD (**5-8**, **S9-S16**) or the EPSRC National Mass Spectrometry Service Centre in Swansea (**10**). High resolution mass spectrometry was carried out by the EPSRC National Mass Spectrometry Service Centre in Swansea.

Macrocycles **1a-d**,[1,2] alkyne **2**,[3] azide **3**,[3] thread **S1**,[1,3] 1-(4-(tri(4-*tert*-butylphenyl)methyl)phenoxy)hexyl bromide (**S2**),[4] 4-(tri(4-*tert*-butylphenyl)methyl)phenol (**S3**),[5] 6-tosyloxyhex-1-yne (**S4**),[6] 11-tosyloxyundec-1-yne (**S5**),[7] 6,6'-di(3-(4-hydroxyphenyl)propyl)-2,2'-bipyridine (**S18**)[8] and macrocycle **9d**[8] were synthesised according to literature procedures.

Experimental Procedures

General Procedure for Scheme 1 and Screening of Reaction Conditions

The desired macrocycle (1 eq.), azide (1 eq.), alkyne (1 eq.) and $Cu(MeCN)_4.PF_6$ (0.90 eq.) were weighed dry into an 8 mL CEM microwave vial. solvent was added to make a 0.01M solution (wrt. macrocycle), the vial sealed under N_2 and the mixture stirred at the desired temperature to react for the specified time. The solution was allowed to return to room temperature before dilution with CH_2Cl_2 (50 mL) and washing with 16% aqueous EDTA tetrasodium-saturated ammonia solution (50 mL). The organic layer was retained and the aqueous layer extracted twice further with CH_2Cl_2 (50 mL portions). The organic extracts were combined, dried over $MgSO_4$ and dried *in vacuo*.

Conditions A

A 0.05 M solution of the desired macrocycle (1 eq.), azide (1.20 eq.), alkyne (1.20 eq.), N^iPr_2Et (1.10 eq.) and $Cu(MeCN)_4.PF_6$ (0.10 eq.) in anhydrous THF was stirred at 30 °C for 6 hours (unless specified otherwise). The solution was allowed to return to room temperature before dilution with CH_2Cl_2 (50 mL) and washing with 16% aqueous EDTA tetrasodium-saturated ammonia solution (50 mL). The organic layer was retained and the aqueous layer extracted twice further with CH_2Cl_2 (50 mL portions). The organic extracts were combined, dried over $MgSO_4$ and dried *in vacuo*.

Conditions B

A 0.01M solution of the desired macrocycle (1 eq.), azide (1.20 eq.), alkyne (1.20 eq.) and $Cu(MeCN)_4.PF_6$ (0.96 eq.) in anhydrous CH_2Cl_2 was stirred at in an 8 mL CEM microwave vial at 100 °C with microwave heating (150W) for 20 minutes (unless specified otherwise). The solution was allowed to return to room temperature before dilution with further CH_2Cl_2 (50 mL) and washing with 16% aqueous EDTA tetrasodium-saturated ammonia solution (50 mL). The organic layer was retained and the aqueous layer extracted twice further with CH_2Cl_2 (50 mL portions). The organic extracts were combined, dried over $MgSO_4$ and dried *in vacuo*.

Selective Synthesis of [2]Rotaxanes 4

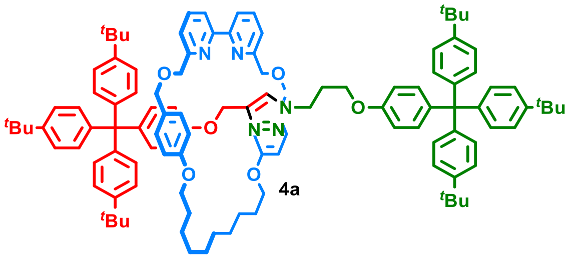

4a

[2]Rotaxane **4a** was made according to conditions A, using macrocycle **1a** (14.1 mg, 0.025 mmol), alkyne **2** (16.3 mg, 0.030 mmol), azide **3** (17.6 mg, 0.030 mmol), N^iPr_2Et (4.8 μL, 0.028 mmol), and $Cu(MeCN)_4.PF_6$ (0.93 mg, 0.0025 mmol) in THF (0.5 mL) stirred at rt for 6 h. Flash column chromatography (0-30% MeCN/1:1 hexane:CH_2Cl_2 (+0.25% EtOH)) afforded 40.3 mg (95% yield) of [2]rotaxane 4a as a white foam: All analytical data was in accord with previously reported literature data.[1]

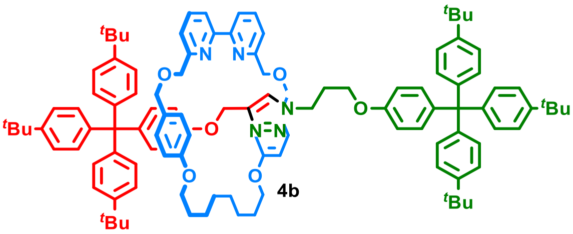

4b

[2]Rotaxane **4b** was made according to conditions A, using macrocycle **1b** (13.4 mg, 0.025 mmol), alkyne **2** (16.3 mg, 0.030 mmol), azide **3** (17.6 mg, 0.030 mmol), N^iPr_2Et (4.8 μL, 0.028 mmol), and $Cu(MeCN)_4.PF_6$ (0.93 mg, 0.0025 mmol) in THF (0.5 mL) stirred at rt for 6 h. Flash column chromatography (0-30% MeCN/1:1 hexane:CH_2Cl_2 (+0.25% EtOH)) afforded 39.3 mg (94% yield) of [2]rotaxane **4b** as a white foam. All analytical data was in accord with previously reported literature data.[2]

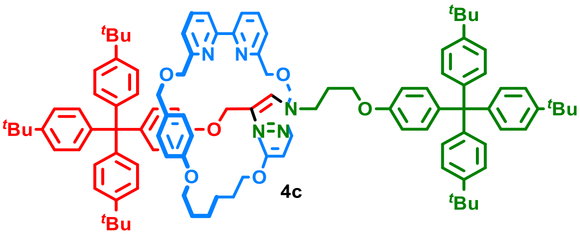

4c

[2]Rotaxane **4c** was made according to conditions A, using macrocycle **1b** (12.7 mg, 0.025 mmol), alkyne **2** (16.3 mg, 0.030 mmol), azide **3** (17.6 mg, 0.030 mmol), N^iPr_2Et (4.8 μL, 0.028 mmol), and $Cu(MeCN)_4.PF_6$ (0.93 mg, 0.0025 mmol) in THF (0.5 mL) stirred at rt for 6 h. Flash column chromatography (0-30% MeCN/1:1 hexane:CH_2Cl_2 (+0.25% EtOH)) afforded 40.6 mg (99% yield) of [2]rotaxane **4c** as a white foam. All analytical data was in accord with previously reported literature data.[2]

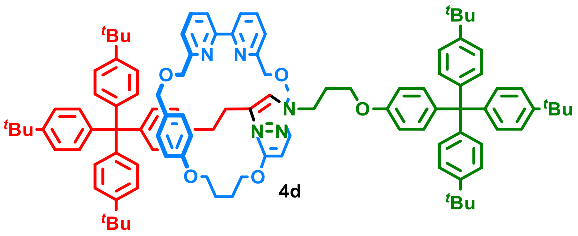

4d

[2]Rotaxane **4d** was made according to conditions A, using macrocycle **1d** (1.4 mg, 0.025 mmol), alkyne **2** (16.3 mg, 0.030 mmol), azide **3** (17.6 mg, 0.030 mmol), N^iPr_2Et (4.8 μL, 0.028 mmol), and $Cu(MeCN)_4.PF_6$ (0.93 mg, 0.0025 mmol) in THF (0.5 mL) stirred at rt for 6 h. Flash column chromatography (0-30% MeCN/1:1 hexane:CH_2Cl_2 (+0.25% EtOH)) afforded 39.9 mg (99% yield) of [2]rotaxane **4d** as a white foam: All analytical data was in accord with previously reported literature data.[2]

Synthesis of [3]Rotaxane 5a

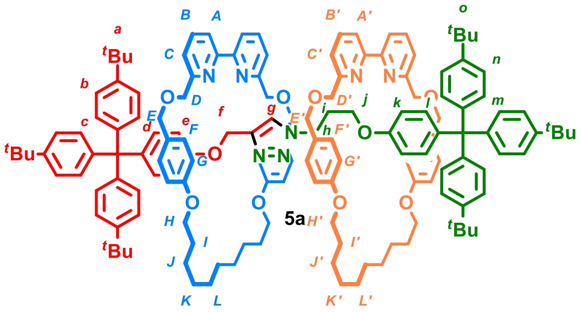

[3]Rotaxane **5a** was made according to conditions B, using macrocycle **1a** (14.1 mg, 0.025 mmol), alkyne **2** (16.3 mg, 0.030 mmol), azide **3** (17.6 mg, 0.030 mmol) and $Cu(MeCN)_4.PF_6$ (8.9 mg, 0.024 mmol) in CH_2Cl_2 (2.5 mL) stirred at 100°C in a 150W microwave reactor for 20 minutes. Flash column chromatography (0-30% MeCN/1:1 hexane:CH_2Cl_2 (+0.25% EtOH)) afforded 14.1 mg (49% yield) of [3]rotaxane **5a** as a white foam: ^{1}H NMR (400 MHz, $CDCl_3$) δ ppm 7.97 (d, *J*=7.6, 2H, H_A), 7.96 (d, *J*=7.6, 2H, $H_{A'}$), 7.43 (t, *J*=7.6, 2H, H_B), 7.40 (t, *J*=7.6, 2H, $H_{B'}$), 7.21 - 7.30 (m, 16H, H_C, $H_{C'}$, H_b and H_n), 7.09 (d, *J*=8.7, 6H, H_c or H_m), 7.05 (d, *J*=8.7, 6H, H_c or H_m), 6.96 (d, *J*=8.7, 4H, H_F), 6.94 (s, 1H, H_g), 6.93 (d, *J*=8.7, 4H, $H_{F'}$), 6.80 (d, *J*=9.1, 2H, H_d or H_l), 6.74 (d, *J*=9.1, 2H, H_d or H_l), 6.51 (d, *J*=8.7, 4H, H_G), 6.49 (d, *J*=8.7, 4H, $H_{G'}$), 6.04 (d, *J*=9.1, 2H, H_e or H_l), 5.97 (d, *J*=9.1, 2H, H_e or H_l), 4.56 – 4.58 (m, 8H, H_D and $H_{D'}$), 4.52 –4.56 (m, 8H, H_E and $H_{E'}$), 3.58 - 3.76 (m, 10H, H_H, $H_{H'}$ and H_j), 2.99 (t, *J*=5.7, 2H, H_h), 1.52 – 1.60 (m, 8H, H_I and $H_{I'}$), 1.39 – 1.46 (m, 2H, H_i), 1.28 – 1.37 (m, 54H, H_a and H_o), 1.17 – 1.28 (m, 8H, H_J and $H_{J'}$), 1.00 – 1.15 (m, 16H, H_K, $H_{K'}$, H_L and $H_{L'}$); ^{13}C NMR (100 MHz, $CDCl_3$) δ ppm 158.7 (C), 158.7 (C), 158.4 (C), 158.3 (C), 156.2 (C), 156.1 (C), 155.6 (C), 155.4 (C), 148.5 (C), 148.4 (C), 148.3 (C), 144.5 (2 x C), 139.4 (C), 139.3 (C), 137.3 (CH), 137.2 (CH), 131.9 (CH), 130.9 (2 x CH), 129.8 (CH), 129.8 (CH), 129.7 (C), 124.2 (CH), 124.2 (CH), 122.9 (CH), 121.6 (CH), 121.5 (CH), 120.0 (CH), 119.9 (CH), 114.5 (CH), 114.5 (CH), 112.8 (CH), 112.8 (CH), 72.6 (CH_2), 72.5 (2 x CH_2), 72.4 (CH_2), 67.8 (CH_2), 67.7 (CH_2), 64.0 (CH_2), 63.2 (C), 63.1 (C), 61.4 (CH_2), 47.0 (CH_2), 34.5 (2 x C), 31.6 (CH_3), 31.6 (CH_3), 29.8 (CH_2), 29.5 (2 x CH_2), 29.0 (CH_2), 29.0 (CH_2), 28.9 (CH_2), 28.9 (CH_2), 25.9 (CH_2), 25.9 (CH_2); LRMS (ESI+) m/z 1154.7 $[M+2Na]^{2+}$.

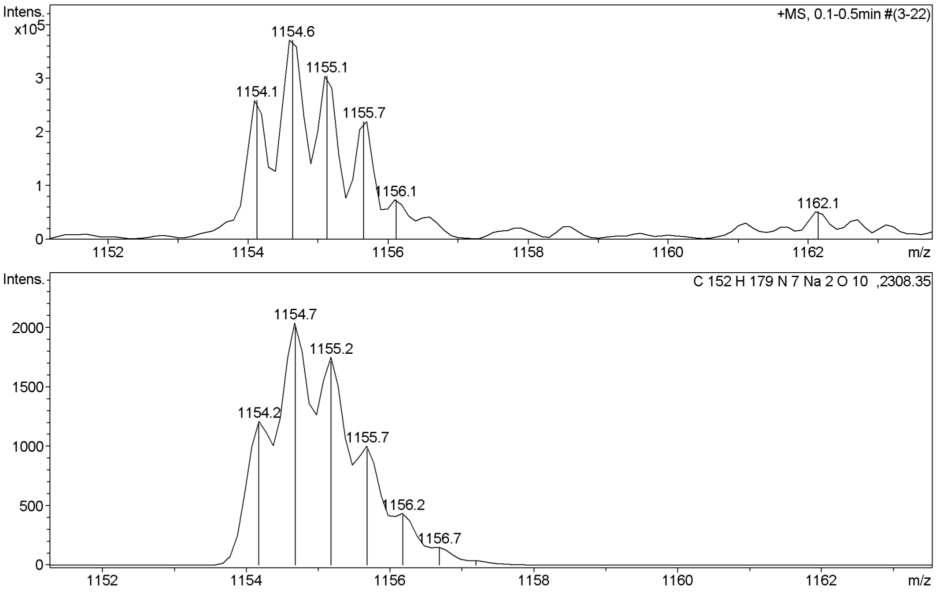

Bruker Daltonics DataAnalysis 3.4 printed: 24/03/2014 12:14:38 Page 1 of 1

Synthesis of [3]Rotaxane 5b

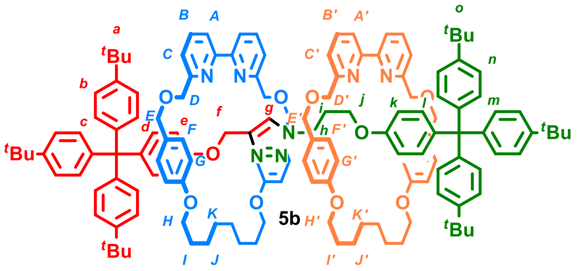

[3]Rotaxane **5b** was made according to conditions B, using macrocycle **1b** (13.4 mg, 0.025 mmol), alkyne **2** (16.3 mg, 0.030 mmol), azide **3** (17.6 mg, 0.030 mmol) and $Cu(MeCN)_4.PF_6$ (8.9 mg, 0.024 mmol) in CH_2Cl_2 (2.5 mL) stirred at 100°C in a 150W microwave reactor for 20 minutes. Flash column chromatography (0-30% MeCN/1:1 hexane:CH_2Cl_2 (+0.25% EtOH)) afforded 13.9 mg (50%) of the target material as a white foam: ^{1}H NMR (400 MHz, $CDCl_3$) δ ppm 7.83 (d, *J*=7.8, 4H, H_A and $H_{A'}$), 7.48 (t, *J*=7.8, 2H, H_B or $H_{B'}$), 7.46 (t, *J*=7.8, 2H, H_B or $H_{B'}$), 7.36 (d, *J*=7.8, 2H, H_C or $H_{C'}$), 7.35 (d, *J*=7.8, 2H, H_C or $H_{C'}$), 7.21 - 7.33 (m, 20H, H_b and H_n), 7.14 (s, 1H, H_g), 7.10 (d, *J*=8.7, 6H, H_c or H_m), 7.06 (d, *J*=8.7, 6H, H_c or H_m), 6.99 (d, *J*=8.7, 4H, H_F or H_F’), 6.96 (d, *J*=8.7, 4H, H_F or H_F’), 6.69 (d, *J*=8.7, 2H, H_d or H_l), 6.58 (d, *J*=8.7, 2H, H_d or H_l), 6.55 (d, *J*=8.7, 4H, H_G or H_G’), 6.51 (d, *J*=8.7, 4H, H_G or H_G’), 5.79 (d, *J*=8.7, 2H, H_e or H_k), 5.78 (d, *J*=8.7, 2H, H_e or H_k), 4.49 - 4.62 (m, 16H, H_D, $H_{D'}$, H_E and $H_{E'}$), 4.24 (s, 2H, H_f), 3.61 - 3.82 (m, 8H, H_H and $H_{H'}$), 3.55 - 3.60 (m, 2H, H_j), 2.90 (t, *J*=5.7, 2H, H_h), 1.45 - 1.64 (m, 8H, H_I and $H_{I'}$), 1.34 - 1.39 (m, 2H, H_i), 1.36 (s, 27H, H_a and H_o), 1.35 (s, 27H, H_a and H_o), 0.87 - 0.96 (m, 10H, H_J, $H_{J'}$, H_K and $H_{K'}$); ^{13}C NMR (100 MHz, $CDCl_3$) δ ppm 158.8 (C), 158.8 (C), 158.6 (C), 158.6 (C), 156.0 (C), 156.0 (C), 155.7 (C), 155.5 (C), 148.4 (C), 148.3 (C), 148.2 (C), 144.6 (2 x C), 139.0 (C), 137.2 (CH), 137.1 (CH), 131.7 (CH), 131.6 (CH), 131.0 (CH), 131.0 (CH), 129.9 (2 x CH), 129.7 (C), 129.5 (C), 124.2 (CH), 124.2 (CH), 123.2 (CH), 120.9 (CH), 120.7 (CH), 120.1 (CH), 119.9 (CH), 114.7 (CH), 114.6 (CH), 112.8 (CH), 112.7 (CH), 72.6 (CH_2), 72.5 (CH_2), 71.8 (CH_2), 71.5 (CH_2), 67.8 (CH_2), 67.7 (CH_2), 63.9 (CH_2), 63.2 (C), 63.1 (C), 61.2 (CH_2), 47.0 (CH_2), 34.5 (2 x C), 31.6 (CH_3), 31.6 (CH_3), 29.7 (CH_2), 29.3 (CH_2), 29.2 (CH_2), 29.0 (CH_2), 28.9 (CH_2), 25.9 (CH_2), 25.9 (CH_2); LRMS (ESI+) m/z 1104.5 $[M+2H]^{2+}$

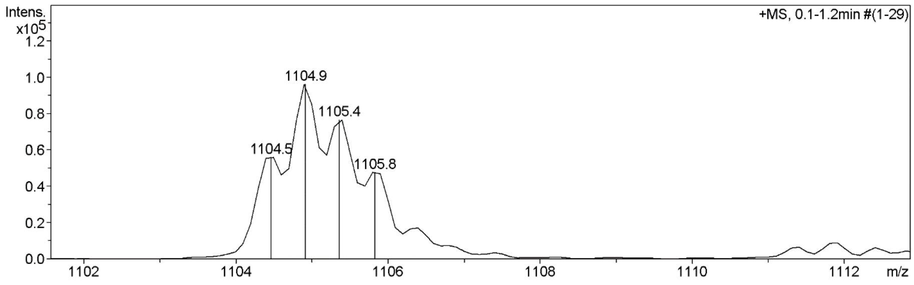

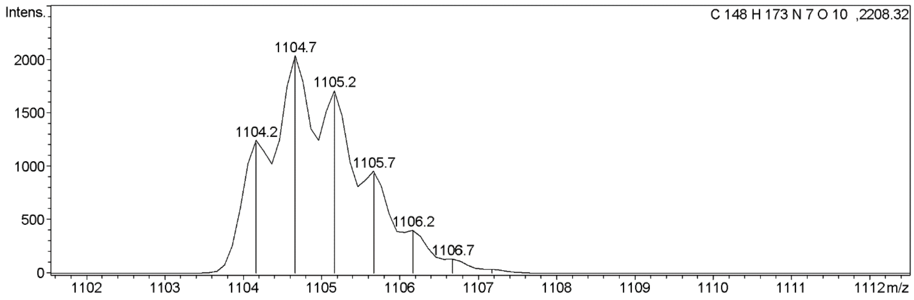

Synthesis of [3]Rotaxane 5c

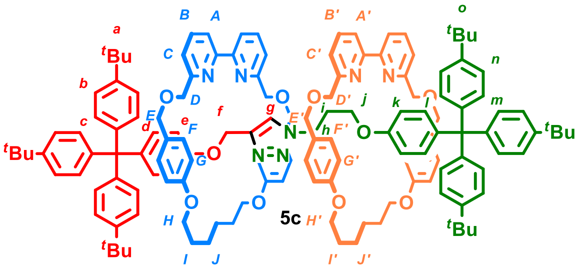

[3]Rotaxane **5c** was made according to conditions B, using macrocycle **1a** (12.7 mg, 0.025 mmol), alkyne **2** (16.3 mg, 0.030 mmol), azide **3** (17.6 mg, 0.030 mmol) and $Cu(MeCN)_4.PF_6$ (8.9 mg, 0.024 mmol) in CH_2Cl_2 (2.5 mL) stirred at 100°C in a 150W microwave reactor for 20 minutes. Flash column chromatography (0-30% MeCN/1:1 hexane:CH_2Cl_2 (+0.25% EtOH)) afforded 5.2 mg (23%) of the target material as a white foam: ^{1}H NMR (400 MHz, $CDCl_3$) δ ppm 7.61 (d, *J*=7.6, 2H, H_A), 7.62 (d, *J*=7.6, 2H, $H_{A'}$), 7.48 - 7.54 (m, 4H, H_B and $H_{B'}$), 7.39 (d, *J*=7.6, 2H, H_C), 7.38 (d, *J*=7.6, 2H, $H_{C'}$), 7.25 - 7.29 (m, 6H, H_b or H_n), 7.29 (d, *J*=8.7, 6H, H_b or H_n), 7.17 (s, 1H, H_g), 7.10 (d, *J*=8.7, 6H, H_c or H_m), 7.05 (d, *J*=8.7, 6H, H_c or H_m), 6.97 (d, *J*=8.7, 2H, H_F or $H_{F'}$), 6.93 (d, *J*=8.7, 2H, H_F or $H_{F'}$), 6.60 (d, *J*=9.1, 2H, H_d or H_l), 6.54 (d, *J*=8.6, 2H, H_G or $H_{G'}$), 6.51 (d, *J*=8.6, 2H, H_G or $H_{G'}$), 5.58 - 5.66 (m, 4H, H_e and H_k), 4.36 - 4.61 (m, 16H, H_D, $H_{D'}$, H_E and $H_{E'}$), 4.22 (s, 2H, H_f), 3.74 - 3.87 (m, 4H, H_H or $H_{H'}$), 3.70 (dt, *J*=9.7, 6.6, 2H, H_H or $H_{H'}$), 3.69 (dt, *J*=9.7, 6.6, 2H, H_H or $H_{H'}$), 3.50 (m, *J*=7.9, 2H, H_h), 2.83 (t, *J*=6.0, 2H, H_j), 1.48 - 1.58 (m, 8H, H_I and $H_{I'}$), 1.35 (s, 27H, H_a), 1.35 (s, 27H, H_o), 1.25 – 1.29 (m, 2H, H_i), 0.87 – 0.96 (m, 8H, H_J and $H_{J'}$); ^{13}C NMR (100 MHz, $CDCl_3$) δ ppm 158.9 (C), 158.9 (C), 158.9 (C), 158.7 (C), 156.0 (C), 155.8 (C), 155.7 (C), 155.6 (C), 148.2 (C), 148.2 (C), 148.1 (C), 144.4 (2 x C), 138.5 (C), 137.0 (CH), 136.9 (CH), 131.4 (CH), 130.9 (CH), 130.8 (CH), 129.9 (2 x CH), 129.3 (C), 129.1 (C), 124.1 (CH), 124.0 (CH), 124.0 (CH), 124.0 (CH), 120.2 (CH), 120.0 (CH), 119.9 (CH), 115.0 (CH), 114.9 (CH), 112.7 (CH), 112.7 (CH), 72.4 (CH_2), 72.4 (CH_2), 70.8 (CH_2), 70.5 (CH_2), 67.8 (CH_2), 63.8 (CH_2), 63.0 (C), 62.9 (C), 60.7 (CH_2), 46.7 (CH_2), 34.3 (2 x C), 31.5 (CH_3), 31.4 (CH_3), 29.7 (CH_2), 29.3 (CH_2), 29.0 (CH_2), 29.0 (CH_2), 25.3 (CH_2), 25.2 (CH_2); LRMS (ESI+) m/z 2152.5 $[M+H]^+$.

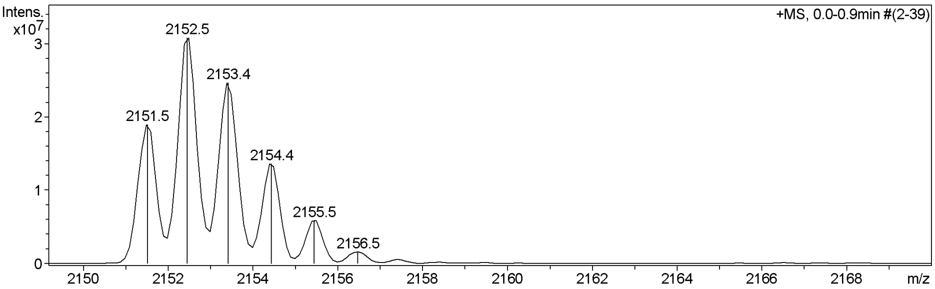

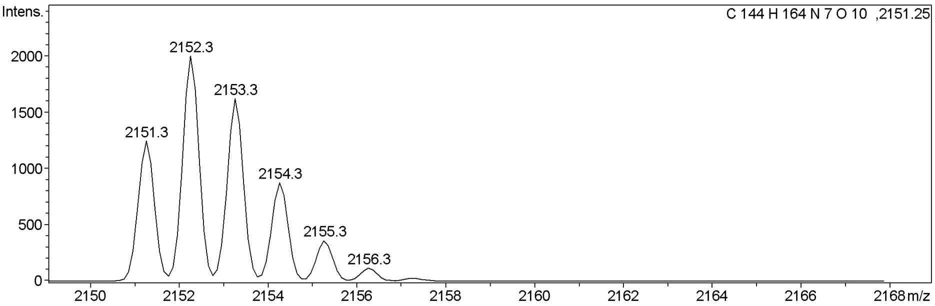

Synthesis of 6-(4-(Tri(4-*tert*-butylphenyl)methyl)phenoxy)hexyl azide (6)

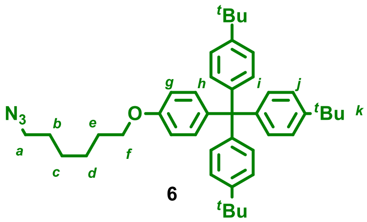

6

A solution of 6-(4-(tri(4-*tert*-butylphenyl)methyl)phenoxy)hexyl bromide (**S2**) (725 mg, 1.086 mmol) and NaN_3 (141.2 mg, 2.172 mmol) in dry NMP (16 mL) was stirred at 80 °C for 18 hours. Water was added (50 mL) and the reaction mixture extracted twice with CH_2Cl_2 (50 mL). The organic layers were then combined, dried over $MgSO_4$ and reduced *in vacuo* to afford a cream solid (CAUTION: Care should be taken with azides under reduced pressure). Recrystallisation from hot $MeCN/CHCl_3$ afforded 253.2 mg (38%) of the target material as a white solid: m.p. 200-202 °C (dec.); ^{1}H NMR (600 MHz, $CDCl_3$) δ ppm 7.26 (d, *J*=8.7, 6H, H_j), 7.06 - 7.14 (m, 8H, H_i and H_g or H_h), 6.78 (d, *J*=8.9, 2H, H_g or H_h), 3.96 (t, *J*=6.3, 2H, H_a), 3.31 (t, *J*=6.9, 2H, H_f), 1.77 - 1.85 (m, 2H, H_b), 1.66 (m, 2H, H_e), 1.44 - 1.55 (m, 4H, H_c and H_d), 1.33 (s, 27H, H_k); ^{13}C NMR (150 MHz, $CDCl_3$) δ 157.0 (C), 148.5 (C), 144.3 (C), 139.6 (C), 132.4 (CH), 130.9 (CH), 124.2 (CH), 113.1 (CH), 67.7 (CH_2), 63.2 (C), 52.0 (CH_2), 34.4 (C), 31.5 (CH_3), 29.4 (CH_2), 29.0 (CH_2), 26.7 (CH_2), 25.9 (CH_2); HRMS (APCI+) m/z 647.4691 $[M+NH_4]^+$ (calc. for $C_{43}H_{59}N_4O$ 647.4683).

Synthesis of 6-(4-(Tri(4-*tert*-butylphenyl)methyl)phenoxy)hex-1-yne (7)

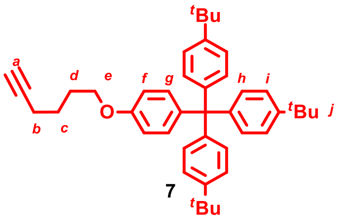

7

A solution of 4-(tri(4-*tert*-butylphenyl)methyl)phenol (**S3**) (510 mg, 1.01 mmol), 6-tosyloxyhex-1-yne (**S4**) (255 mg, 1.01 mmol) and K_2CO_3 (698 mg, 5.05 mmol) in acetone (10 mL) was refluxed with stirring for 18 hours. The reaction mixture was filtered, dried ($MgSO_4$) then reduced *in vacuo* to afford a beige solid. Recrystallisation from hot $MeCN/CHCl_3$ afforded 94.3 mg (16%) of the target material as a white solid: m.p. 231-233 °C; ^{1}H NMR (600 MHz, $CDCl_3$) δ ppm 7.24 (d, *J*=8.7, 6H, H_h or H_i), 6.99 - 7.16 (m, 8H, H_f or H_g and H_h or H_i), 6.76 (d, *J*=8.9, 2H, H_f or H_g), 3.97 (t, *J*=6.2, 2H, H_e), 2.28 (td, *J*=7.1, 2.6, 2H, H_b), 1.97 (t, *J*=2.6 Hz, 1H, H_a), 1.86 - 1.95 (m, 2H, H_d), 1.73 (m, 2H, H_c), 1.31 (s, 27H, H_j); ^{13}C NMR (150 MHz, $CDCl_3$) δ ppm 157.0 (C), 148.5 (C), 144.3 (C), 139.7 (C), 132.4 (CH), 130.9 (CH), 124.2 (CH), 113.1 (CH), 68.7 (C), 67.3 (CH_2), 63.2 (C), 34.4 (C), 31.5 (CH_3), 28.5 (CH_2), 25.3 (CH_2), 18.3 (CH_2); HRMS (APCI+) m/z 585.4092 $[M+H]^+$ (calc. for $C_{43}H_{53}O$ 585.4091).

Synthesis of 11-(4-(Tri(4-*tert*-butylphenyl)methyl)phenoxy)undec-1-yne (8)

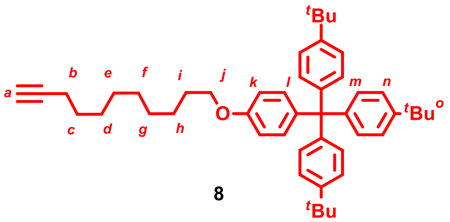

8

A solution of 4-(tri(4-*tert*-butylphenyl)methyl)phenol (**S3**) (252 mg, 0.05 mmol), 11-tosyloxyundec-1-yne (**S5**) (161 mg, 0.05 mmol) and K_2CO_3 (276 mg, 2 mmol) in acetone (5 mL) was refluxed with stirring for 18 hours. The reaction mixture was filtered, dried ($MgSO_4$) then reduced *in vacuo* to afford a beige solid. Column chromatography

(0-10% CH_2Cl_2/petrol) afforded 111.7 mg (34%) of the target material as a white solid: m.p. 155-159 °C; ^{1}H NMR (400 MHz, $CDCl_3$) δ ppm 7.24 (d, *J*=8.6, 6H, H_m or H_n), 7.10 (d, *J*=8.6, 6H, H_m or H_n), 7.09 (d, *J*=8.8, 2H, H_k or H_l), 6.77 (d, *J*=8.8, 2H, H_k or H_l), 3.94 (t, *J*=6.5, 2H, H_j), 2.19 (td, *J*=7.0, 2.5, 2H, H_b), 1.94 (t, *J*=2.5, 1H, H_a), 1.77 (m, 2H, H_i), 1.12 - 1.60 (m, 12H, H_c,H_d, H_e, H_f, H_g and H_h), 1.31 (s, 27H, H_o); ^{13}C NMR (150 MHz, $CDCl_3$) δ ppm 157.1 (C), 148.4 (C), 144.4 (C), 139.5 (C), 132.4 (CH), 130.9 (CH), 124.2 (CH), 113.1 (CH), 68.2 (C), 68.0 (CH_2), 63.2 (C), 34.4 (3 x C), 31.5 (9 x CH_3), 29.6 (CH_2), 29.5 (2 x CH_2), 29.2 (CH_2), 28.9 (CH_2), 28.6 (CH_2), 26.2 (CH_2), 18.5 (CH_2); HRMS (APCI+) m/z 672.5136 $[M+NH_4]^+$ (calc. for $C_{43}H_{53}O$ 672.5139).

Synthesis of [3]Rotaxane S6

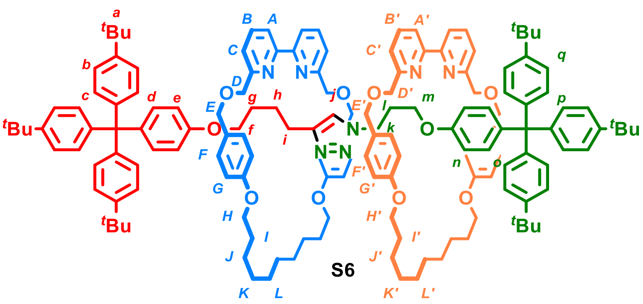

[3]Rotaxane **S6** was made according to conditions B, using macrocycle **1a** (14.2 mg, 0.025 mmol), alkyne **7** (17.5 mg, 0.030 mmol), azide **3** (17.6 mg, 0.030 mmol) and $Cu(MeCN)_4.PF_6$ (8.9 mg, 0.024 mmol) in CH_2Cl_2 (2.5 mL) stirred at 100°C in a 150W microwave reactor for 1 hour. Flash column chromatography (0-30% MeCN/1:1 hexane:CH_2Cl_2 (+0.25% EtOH)) afforded 9.4 mg (32%) of the target material as a white foam: ^{1}H NMR (400 MHz, $CDCl_3$) δ ppm 7.98 (d, J=8.1, 2H, H_A or $H_{A'}$), 8.00 (d, J=8.1, 2H, H_A or $H_{A'}$), 7.44 (t, J=8.1, 2H, H_B or $H_{B'}$), 7.47 (t, J=8.1, 2H, H_B or $H_{B'}$), 7.27 (d, J=8.6, 6H, H_b or H_q), 7.25 (d, J=8.6, 6H, H_b or H_q), 7.21-7.31 (m, 4H, H_C and $H_{C'}$), 7.10 (d, J=8.2, 6H, H_c or H_p), 7.08 (d, J=8.5, 6H, H_c or H_p), 6.99 (d, J=8.6, 4H, H_F or $H_{F'}$), 6.97 (d, J=8.6, 4H, H_F or $H_{F'}$), 6.84 (d, J=8.8, 2H, H_d), 6.79 (d, J=8.8, 2H, H_o), 6.67 (s, 1H, H_j), 6.55 (d, J=8.6, 4H, H_G or $H_{G'}$), 6.54 (d, J=8.6, 4H, H_G or $H_{G'}$), 6.06 (d, J=8.8, 2H, H_e), 6.05 (d, J=8.8, 2H, H_n), 4.57 (s, 8H, H_D and $H_{D'}$ or H_E and $H_{E'}$), 4.56 (s, 8H, H_D and $H_{D'}$ or H_E and $H_{E'}$), 3.64 - 3.79 (m, 10H, H_H), 3.10 (t, J=6.1, 2H, H_f), 3.06 (t, J=5.7, 2H, H_m), 2.26 (t, J=7.2, 2H, H_i), 1.47 - 1.66 (m, 10H, H_I, $H_{I'}$ and H_l), 1.19 - 1.39 (m, 12H, H_J, $H_{J'}$, H_g and H_h), 1.33 (s, 27H, H_a or H_r), 1.33 (s, 27H, H_a or H_r), 1.03 - 1.18 (m, 16H, H_K, $H_{K'}$, H_L and $H_{L'}$); ^{13}C NMR (150 MHz, $CDCl_3$) δ ppm 158.7 (C), 158.7 (C), 158.4 (2 x C), 156.7 (C), 156.2 (C), 155.6 (C), 155.4 (C), 148.4 (C), 148.3 (C), 147.4 (C), 144.5 (C), 144.4 (C), 139.4 (C), 138.8 (C), 137.3 (CH), 137.2 (CH), 131.9 (CH), 131.8 (CH), 130.9 (CH), 130.9 (CH), 129.8 (2 x C), 129.8 (CH), 129.8 (CH), 124.2 (CH), 124.2 (CH), 121.6 (CH), 121.5 (CH), 120.8 (CH), 120.0 (CH), 119.8 (CH), 114.5 (CH), 114.5 (CH), 112.9 (CH), 72.6 (CH_2), 72.5 (CH_2), 72.5 (CH_2), 72.4 (CH_2), 67.8 (CH_2), 67.8 (CH_2), 66.9 (CH_2), 63.9 (CH_2), 63.2 (C), 63.2 (C), 46.8 (CH_2), 34.5 (C), 34.5 (C), 31.6 (2 x CH_3), 29.9 (CH_2), 29.6 (CH_2), 29.5 (CH_2), 29.1 (2 x CH_2), 29.0 (CH_2), 29.0 (CH_2), 29.0 (CH_2), 26.0 (CH_2), 26.0 (CH_2), 25.7 (CH_2), 25.4 (CH_2); LRMS (ESI+) 1153.2 m/z $[M+2H]^{2+}$.

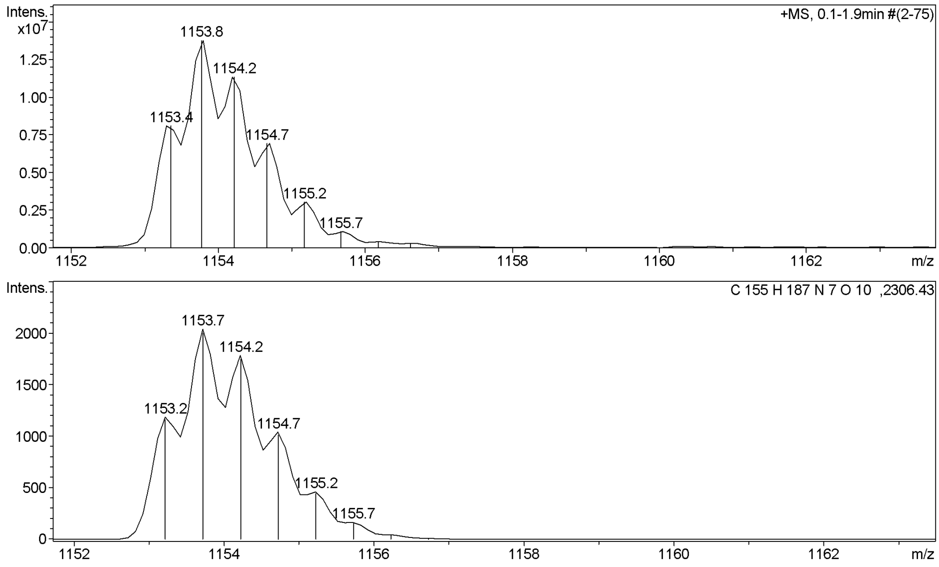

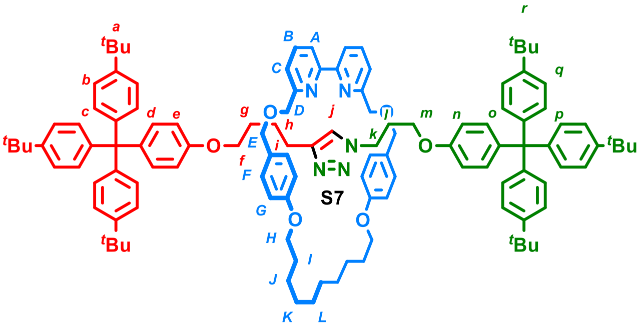

[2]Rotaxane **S7** was also isolated (17.9 mg, 41%) as a white foam: ^{1}H NMR (400 MHz, $CDCl_3$) δ ppm 7.97 (d, J=7.8, 2H, H_A), 7.49 (t, J=7.8, 2H, H_B), 7.29 (d, J=7.8, 2H, H_C), 7.25 (d, J=8.7, 6H, H_b or H_q), 7.24 (d, J=8.6, 6H, H_b or H_q), 7.10 (d, J=8.6, 6H, H_c or H_p), 7.11 (d, J=8.7, 6H, H_c or H_p), 6.97 - 7.05 (m, 8H, H_F, H_d and H_o), 6.78 (s, 1H, H_j), 6.58 (d, J=9.0, 2H, H_e), 6.59 (d, J=8.7, 4H, H_G), 6.42 (d, J=9.0, 2H H_n), 4.57 (s, 4H, H_D or H_E), 4.55 (s, 4H, H_D or H_E), 3.90 (t, J=7.3, 2H, H_k), 3.76 (t, J=6.5, 4H, H_H), 3.66 (t, J=5.8, 2H, H_f), 3.40 (t, J=5.7, 2H, H_m), 2.45 (t, J=7.1, 2H, H_i), 1.73 - 1.82 (tt, J=7.3, 5.7, 2H, H_l), 1.45 - 1.65 (m, 8H, H_I, H_g and H_h), 1.22 - 1.37 (m, 4H, H_J), 1.32 (s, 27H, H_a or H_r), 1.31 (s, 27H, H_a or H_r), 1.09 - 1.21 (m, 8H, H_K and H_L); ^{13}C NMR (100 MHz, $CDCl_3$) δ ppm 158.6 (C), 158.2 (C), 156.8 (C), 156.2 (C), 155.5 (C), 148.3 (C), 148.3 (C), 147.4 (C), 144.3 (C), 144.2 (C), 139.7 (C), 139.3 (C), 137.1 (CH), 132.1 (CH), 130.8 (CH), 130.7 (CH), 129.7 (C), 129.7 (CH), 124.1 (CH), 124.0 (CH), 121.5 (CH), 121.0 (CH), 119.9 (CH), 114.4 (CH), 112.9 (CH), 112.9 (CH), 72.5 (CH_2), 72.3 (CH_2), 67.7 (CH_2), 67.2 (CH_2), 63.9 (CH_2), 63.1 (2 x C), 46.6 (CH_2), 34.3 (2 x C), 31.4 (2 x CH_3), 29.8 (CH_2), 29.4 (CH_2), 28.9 (CH_2), 28.8 (CH_2), 28.8 (CH_2), 25.8 (CH_2), 25.8 (CH_2), 25.2 (CH_2); LRMS (ESI+) m/z 870.1 $[M+2H]^+$.

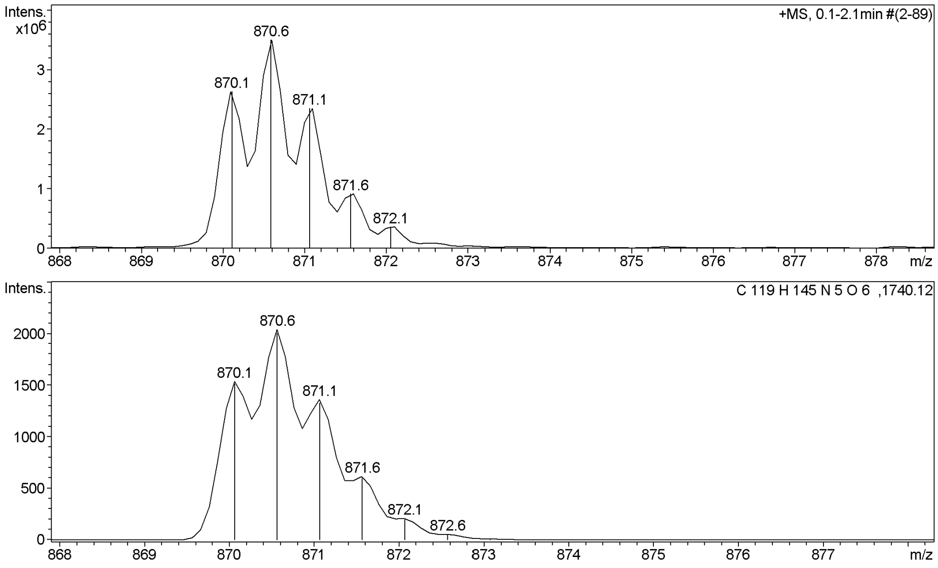

Synthesis of [3]Rotaxane S8

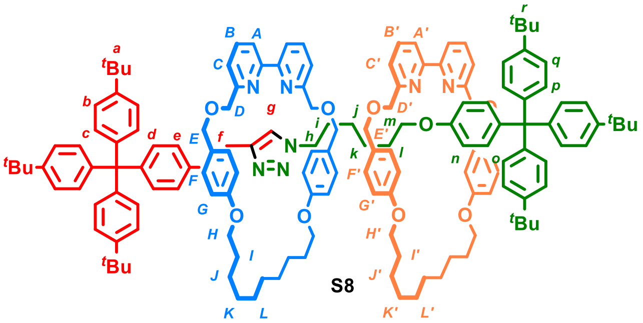

[3]Rotaxane **S8** was made according to conditions B, using macrocycle **1a** (14.2 mg, 0.025 mmol), alkyne **2** (16.3 mg, 0.030 mmol), azide **6** (18.9 mg, 0.030 mmol) and $Cu(MeCN)_4.PF_6$ (8.9 mg, 0.024 mmol) in CH_2Cl_2 (2.5 mL) stirred at 100°C in a 150W microwave reactor for 1 hour. Flash column chromatography (0-30% MeCN/1:1 hexane:CH_2Cl_2 (+0.25% EtOH)) afforded 13.7 mg (48%) of the target material as a white foam: ^{1}H NMR (400 MHz, $CDCl_3$) δ ppm 8.01 (d, *J*=7.7, 2H, H_A or $H_{A'}$), 7.96 (d, *J*=7.7, 2H, H_A or $H_{A'}$), 7.47 (t, *J*=7.7, 2H, H_B or $H_{B'}$), 7.46 (t, *J*=7.7, 2H, H_B or $H_{B'}$), 7.31 – 7.22 (m, 4H, H_C and $H_{C'}$), 7.25 (d, *J*=8.7, 6H, H_b or H_q), 7.25 (d, *J*=8.7, 6H, H_b or H_q), 7.09 (d, *J*=8.7, 6H, H_c or H_p), 7.07 (d, *J*=8.7, 6H, H_c or H_p), 7.06 (s, 1H, H_g), 7.00 (d, *J*=8.7, 4H, H_F or $H_{F'}$), 6.97 (d, *J*=8.8, 4H, H_F or $H_{F'}$), 6.84 (d, *J*=8.8, 2H, H_d), 6.85 (d, *J*=8.8, 2H, H_o), 6.54 (d, *J*=8.7, 4H, H_G or $H_{G'}$), 6.56 (d, *J*=8.8, 4H, H_G or $H_{G'}$), 6.26 (d, *J*=8.8, 2H, H_e), 6.14 (d, *J*=8.8, 2H, H_n), 4.45 - 4.62 (m, 16H, H_D, $H_{D'}$, H_E and $H_{E'}$), 4.49 (s, 2H, H_f), 3.66 - 3.78 (m, 8H, H_H and $H_{H'}$), 3.52 (t, *J*=7.7, 2H, H_h), 3.06 (t, *J*=6.5, 2H, H_m), 1.51 - 1.66 (m, 8H, H_I and $H_{I'}$), 1.32 (s, 54H, H_a and H_r), 1.19 - 1.37 (m, 8H, H_J and $H_{J'}$), 1.12 (d, *J*=7.7, 18H, H_K, $H_{K'}$, H_L, $H_{L'}$ and H_i), 0.55 - 0.76 (m, 4H, H_k and H_j); ^{13}C NMR (150 MHz, $CDCl_3$) δ ppm 158.7 (C), 158.7 (C), 158.4 (C), 158.4 (C), 156.8 (C), 156.2 (C), 155.5 (2 x C), 148.3 (2 x C), 147.4 (C), 144.5 (C), 144.4 (C), 139.6 (C), 139.0 (C), 137.2 (CH), 137.2 (CH), 132.0 (CH), 131.9 (CH), 130.9 (2 x CH), 129.8 (2 x C), 129.8 (CH), 129.8 (CH), 124.2 (CH), 124.2 (CH), 122.9 (CH), 121.5 (CH), 121.5 (CH), 120.0 (CH), 119.9 (CH), 114.5 (CH), 114.5 (CH), 113.0 (CH), 112.9 (2 x CH), 72.6 (CH_2), 72.5 (CH_2), 72.4 (CH_2), 72.4 (CH_2), 67.8 (CH_2), 67.7 (CH_2), 67.0 (CH_2), 63.2 (2 x C), 61.6 (CH_2), 49.7 (CH_2), 34.5 (2 x C), 31.6 (2 x CH_3), 29.8 (CH_2), 29.6 (CH_2), 29.5 (CH_2), 29.1 (CH_2), 29.0 (2 x CH_2), 28.9 (CH_2), 28.9 (CH_2), 26.2 (CH_2), 26.0 (CH_2), 25.9 (CH_2), 25.2 (CH_2); LRMS (ESI+) 1153.2 m/z $[M+2H]^{2+}$.

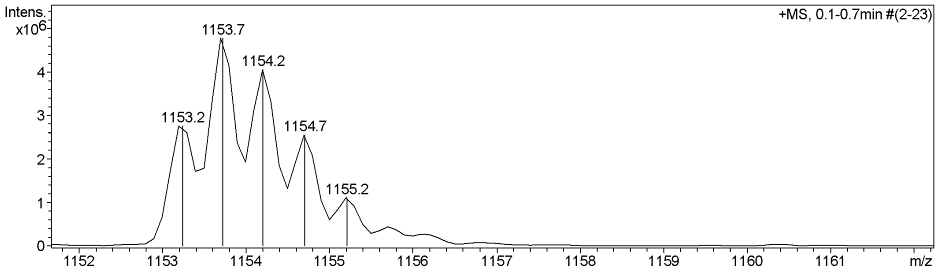

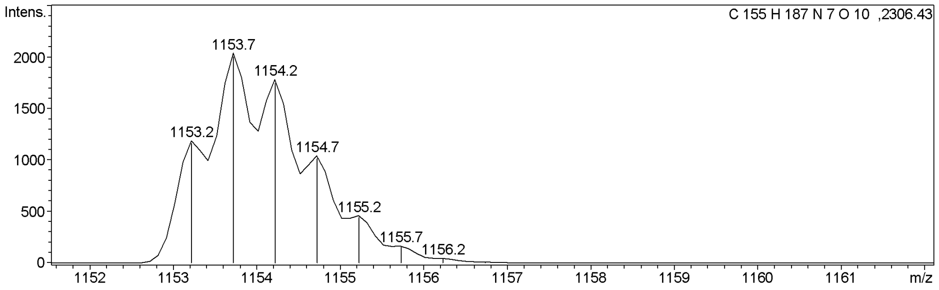

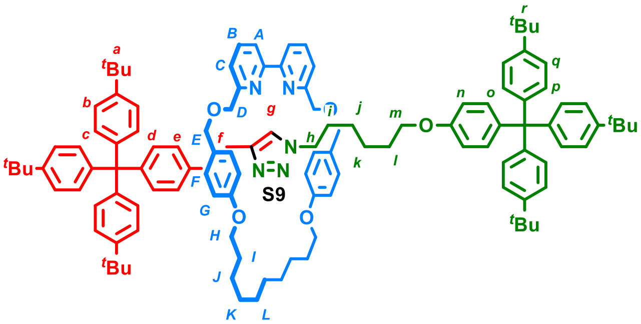

[2]Rotaxane **S9** was also isolated (19.6 mg, 45%) as a white foam: ^{1}H NMR (400 MHz, $CDCl_3$) δ ppm 7.97 (d, *J*=7.8, 2H, H_A), 7.52 (t, *J*=7.8, 2H, H_B), 7.31 (d, *J*=7.8, 2H, H_C), 7.25 (d, *J*=8.6, 6H, H_b or H_q), 7.24 (d, *J*=8.6, 6H, H_b or H_q), 7.12 (d, *J*=8.6, 6H, H_c or H_p), 7.09 (d, *J*=8.6, 6H, H_c or H_p), 7.04 (d, *J*=8.6, 4H, H_F), 7.00 - 7.16 (m, 4H H_g, H_d and H_o), 6.74 (d, *J*=9.0, 2H, H_e), 6.61 (d, *J*=8.6, 4H, H_G), 6.59 (d, *J*=9.1, 2H, H_n), 4.94 (s, 2H, H_f), 4.57 (s, 4H, H_D or H_E), 4.56 (s, 4H, H_D or H_E), 3.79 (t, *J*=6.4, 4H, H_H), 3.61 (t, *J*=7.6, 2H, H_h), 3.51 (t, *J*=6.4, 2H, H_m), 1.62 (tt, *J*=7.2, 6.4, 4H, H_I), 1.06 - 1.37 (m, 16H, H_J, H_K, H_L, H_i and H_l), 1.32 (s, 27H, H_a), 1.31 (s, 27H, H_r), 0.90 (m, 2H, H_j), 0.74 (m, 2H, H_k); ^{13}C NMR (100 MHz, $CDCl_3$) δ ppm 158.8 (C), 158.4 (C), 157.0 (C), 156.4 (C), 155.7 (C), 148.5 (C), 148.4 (C), 144.4 (C), 144.3 (C), 143.8 (C), 140.1 (C), 139.4 (C), 137.2 (CH), 132.4 (CH), 132.3 (CH), 130.9 (2 x CH), 129.8 (CH), 129.8 (C), 124.2 (2 x CH), 122.9 (CH), 121.6 (CH), 120.0 (CH), 114.5 (CH), 113.3 (CH), 113.1 (CH), 72.6 (CH_2), 72.3 (CH_2), 67.8 (CH_2), 67.3 (CH_2), 63.2 (2 x C), 62.0 (CH_2), 49.8 (CH_2), 34.4 (2 x C), 31.5 (2 x CH_3), 29.7 (CH_2), 29.5 (CH_2), 29.1 (CH_2), 28.9 (CH_2), 28.9 (CH_2), 26.0 (CH_2), 26.0 (CH_2), 25.2 (CH_2); LRMS (ESI+) 869.9 m/z $[M+2H]^{2+}$.

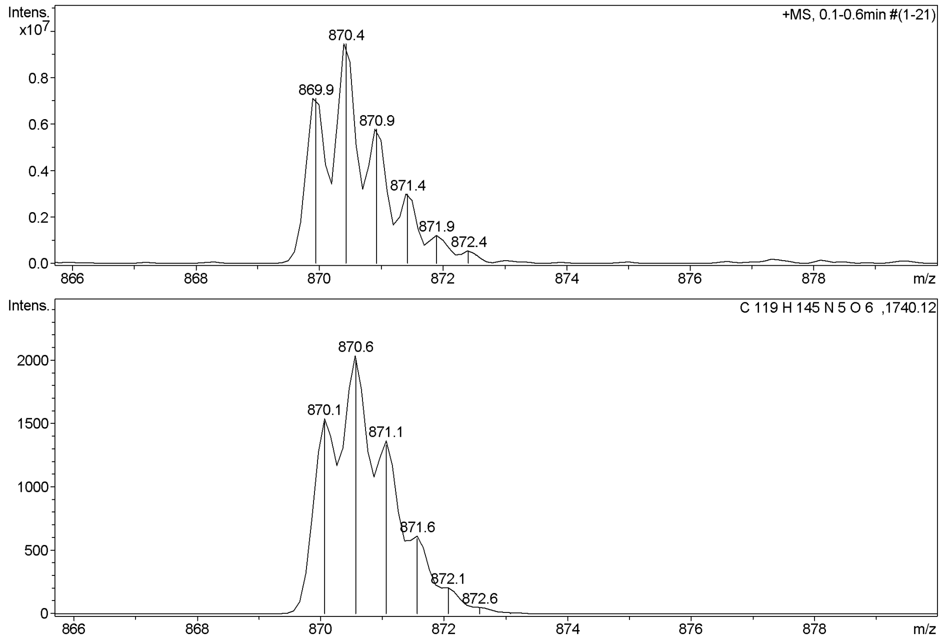

Synthesis of [3]Rotaxane S10a

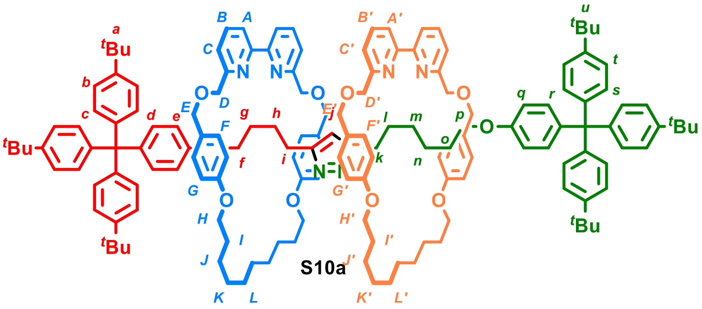

[3]Rotaxane **S10a** was made according to conditions B, using macrocycle **1a** (14.2 mg, 0.025 mmol), alkyne **7** (17.5 mg, 0.030 mmol), azide **6** (18.9 mg, 0.030 mmol) and $Cu(MeCN)_4.PF_6$ (8.9 mg, 0.024 mmol), in CH_2Cl_2 (2.5 mL) stirred at 100°C in a 150W microwave reactor for 45 minutes. Flash column chromatography (0-30% MeCN/1:1 hexane:CH_2Cl_2 (+0.25% EtOH)) afforded 11.3 mg (35%) of the target material as a white foam: ^{1}H NMR (400 MHz, $CDCl_3$) δ ppm 8.01 (d, *J*=7.8, 2H, H_A or $H_{A'}$), 8.00 (d, *J*=7.8, 2H, H_A or $H_{A'}$), 7.51 (t, *J*=7.8, 2H, H_B or $H_{B'}$), 7.48 (t, *J*=7.8, 2H, H_B or $H_{B'}$), 7.31 (d, *J*=7.8, 2H, H_C or $H_{C'}$), 7.29 (d, *J*=7.8, 2H, H_C or $H_{C'}$), 7.25 (d, *J*=8.5, 12H, H_b and H_t), 7.10 (d, *J*=8.5, 6H, H_c or H_s), 7.09 (d, *J*=8.5, 6H, H_c or H_s), 7.02 (d, *J*=8.7, 4H, H_F or $H_{F'}$), 6.99 (d, *J*=8.7, 4H, H_F or $H_{F'}$), 6.90 (d, *J*=8.8, 2H, H_d), 6.85 (d, *J*=8.8, 2H, H_r), 6.80 (s, 1H, H_j), 6.59 (d, *J*=8.7, 4H, H_G or $H_{G'}$), 6.57 (d, *J*=8.7, 4H, H_G or $H_{G'}$), 6.25 (d, *J*=8.8, 2H, H_e), 6.17 (d, *J*=8.8, 2H, H_q), 4.54 - 4.61 (m, 16H, H_D, $H_{D'}$, H_E and $H_{E'}$), 3.70 - 3.81 (m, 8H, H_H), 3.65 (m, *J*=7.7, 2H, H_p), 3.21 (t, *J*=5.8, 2H, H_f), 3.16 (t, *J*=6.5, 2H, H_k), 2.32 (t, *J*=7.1, 2H, H_i), 1.51 - 1.68 (m, 6H, H_h, H_I), 1.08 - 1.37 (m, 14H, H_g, H_l, H_o, H_J and H_K), 1.32 (s, 54H, H_a and H_u), 0.80 - 0.96 (m, 4H, H_L), 0.62 - 0.80 (m, 4H, H_m and H_n); ^{13}C NMR (150 MHz, $CDCl_3$) δ ppm 158.7 (2 x C), 158.4 (2 x C), 156.8 (C), 156.8 (C), 155.6 (C), 155.5 (C), 148.4 (C), 148.3 (C), 147.4 (C), 144.5 (C), 144.5 (C), 139.1 (C), 139.0 (C), 137.2 (CH), 137.2 (CH), 132.0 (CH), 131.9 (CH), 130.9 (2 x CH), 129.9 (2 x C), 129.8 (CH), 129.8 (CH), 124.2 (CH), 124.2 (CH), 121.5 (2 x CH), 120.6 (CH), 119.9 (CH), 119.9 (CH), 114.5 (CH), 114.5 (CH), 113.0 (CH), 112.9 (CH), 72.5 (2 x CH_2), 72.4 (CH_2), 72.3 (CH_2), 67.8 (CH_2), 67.8 (CH_2), 67.1 (CH_2), 67.0 (CH_2), 63.2 (2 x C), 49.7 (CH_2), 34.5 (2 x C), 31.6 (2 x CH_3), 30.0 (CH_2), 29.8 (CH_2), 29.6 (CH_2), 29.6 (CH_2), 29.1 (CH_2), 29.1 (CH_2), 29.0 (CH_2), 29.0 (CH_2), 28.9 (CH_2), 26.2 (CH_2), 26.0 (CH_2), 26.0 (CH_2), 25.8 (CH_2), 25.5 (CH_2), 25.2 (CH_2); LRMS (ESI+) m/z 1185.7 $[M+Na+H]^{2+}$.

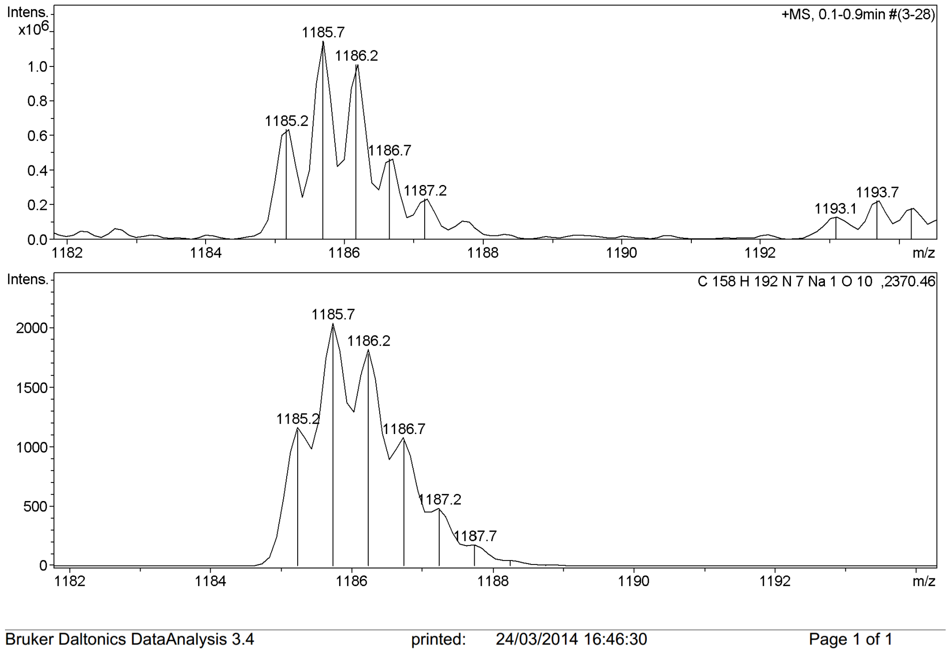

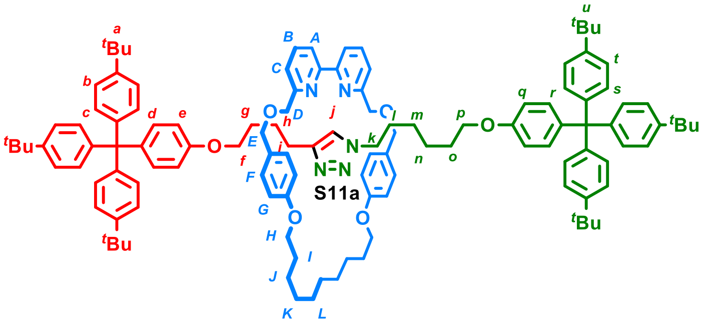

[2]Rotaxane **S11a** was also isolated (27.5 mg, 55%) as a white foam: ^{1}H NMR (400 MHz, $CDCl_3$) δ ppm 7.99 (d, *J*=7.7, 2H, H_A), 7.55 (t, *J*=7.7, 2H, H_B), 7.33 (d, *J*=7.7, 2H, H_C), 7.21 - 7.26 (m, 12H, H_b and H_t), 7.10 (t, *J*=8.2, 12H, H_c and H_s), 7.00 - 7.06 (m, 8H, H_F, H_d and H_r), 6.93 (s, 1H, H_j), 6.62 (d, *J*=8.5, 4H, H_G), 6.59 (d, *J*=9.1, 2H, H_e), 6.57 (d, *J*=8.9, 2H, H_q), 4.57 (s, 4H, H_D or H_E), 4.56 (s, 4H, H_D or H_E), 3.74 - 3.81 (m, 6H, H_H and H_k), 3.71 (t, *J*=5.8, 2H, H_f), 3.53 (t, *J*=6.4, 2H, H_p), 2.54 (t, *J*=7.1, 2H, H_i), 1.59 - 1.66 (m, 8H, H_I, H_g and H_h), 1.23 - 1.43 (m, 8H, H_J, H_l and H_o), 1.31 (s, 27H, H_a or H_u), 1.31 (s, 27H, H_a or H_u), 1.11 - 1.22 (m, 8H, H_K and H_L), 1.00 (m, 2H, H_n), 0.80 - 0.96 (m, 2H, H_m); ^{13}C NMR (100 MHz, $CDCl_3$) δ ppm 158.8 (C), 158.5 (C), 156.9 (2 x C), 155.7 (C), 148.4 (C), 148.4 (C), 147.6 (C), 144.4 (C), 144.2 (C), 139.4 (C), 139.4 (C), 137.2 (CH), 132.2 (CH), 130.9 (2 x CH), 129.8 (C), 129.8 (CH), 124.2 (2 x CH), 121.5 (CH), 120.7 (CH). 120.0 (CH), 114.5 (CH), 113.0 (CH), 72.6 (CH_2), 72.3 (CH_2), 67.8 (CH_2), 67.3 (CH_2), 63.2 (2 x C), 49.8 (CH_2), 34.4 (2 x C), 31.5 (2 x CH_3), 30.0 (CH_2), 29.6 (CH_2), 29.1 (CH_2), 29.0 (CH_2), 29.0 (2 x CH_2), 26.2 (CH_2), 26.0 (CH_2), 26.0 (2 x CH_2), 25.5 (CH_2), 25.4 (CH_2); LRMS (ESI+) 891.0 m/z $[M+2H]^{2+}$

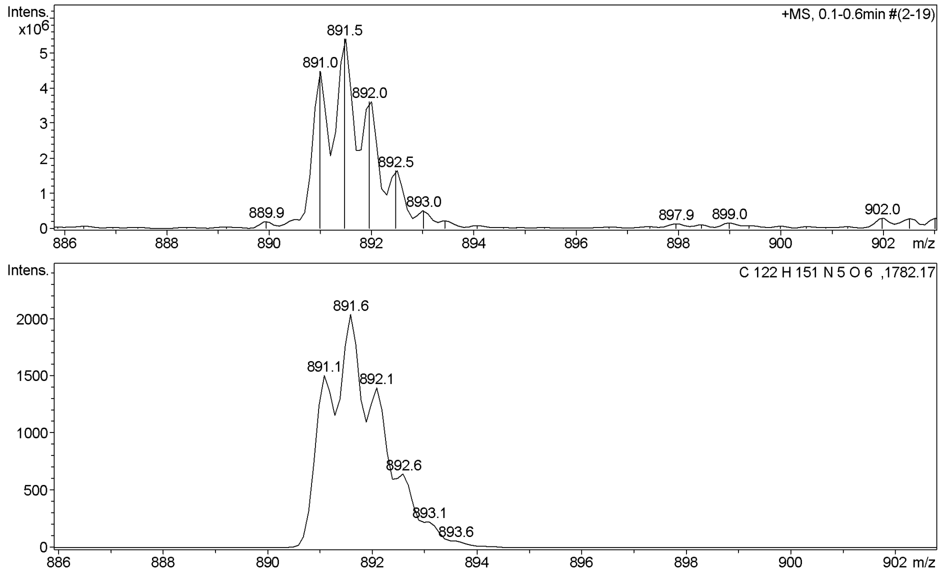

Bruker Daltonics DataAnalysis 3.4 printed: 02/08/2013 15:38:05 Page 1 of 1

Synthesis of [3]Rotaxane S12

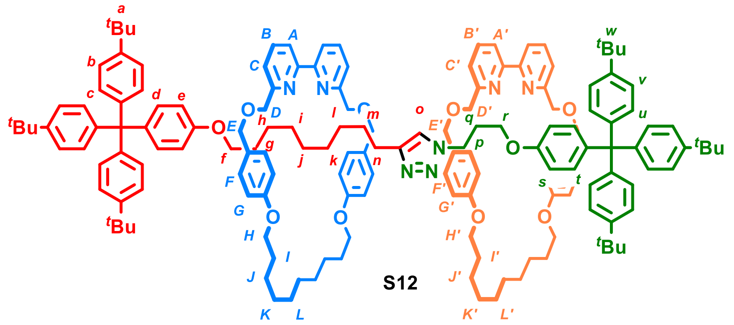

S12

[3]Rotaxane **S12** was made according to conditions B, using macrocycle **1a** (14.2 mg, 0.025 mmol), alkyne **8** (19.7 mg, 0.030 mmol), azide **3** (17.6 mg, 0.030 mmol) and $Cu(MeCN)_4.PF_6$ (8.9 mg, 0.024 mmol) in CH_2Cl_2 (2.5 mL) stirred at 100°C in a 150W microwave reactor for 2 hours. Flash column chromatography (0-30% MeCN/1:1 hexane:CH_2Cl_2 (+0.25% EtOH)) afforded 10.3 mg (34%) of the target material as a white foam: ^{1}H NMR (400 MHz, $CDCl_3$) δ ppm 8.05 (d, *J*=7.8, 2H, H_A or $H_{A'}$), 7.99 (d, *J*=7.8, 2H, H_A or $H_{A'}$), 7.52 (t, *J*=7.8, 3H, H_B or $H_{B'}$), 7.51 (t, *J*=7.8, 2H, H_B or $H_{B'}$), 7.31 (d, *J*=7.8, 4H, H_C and $H_{C'}$), 7.21 - 7.29 (m, 12H, H_b and H_v), 7.11 (d, *J*=8.4, 6H, H_c or H_u), 7.10 (d, *J*=8.4, 6H, H_c or H_u), 7.04 (d, *J*=8.7, 4H, H_F or $H_{F'}$), 7.02 (d, *J*=8.7, 4H, H_F or $H_{F'}$), 6.91 (d, *J*=8.8, 2H, H_d or H_t), 6.89 (d, *J*=8.8, 2H, H_d or H_t), 6.75 (s, 1H, H_o), 6.61 (d, *J*=8.7, 4H, H_G or $H_{G'}$), 6.58 (d, *J*=8.7, 4H, H_G or $H_{G'}$), 6.26 (d, *J*=8.8, 2H, H_e or H_s), 6.20 (d, *J*=8.8, 2H, H_e or H_s), 4.53 – 4.63 (m, 16H, H_D, $H_{D'}$, H_E and $H_{E'}$), 3.68 – 3.83 (m, 10H, H_H, $H_{H'}$ and H_p), 3.20 (t, *J*=6.7, 2H, H_f), 3.16 (t, *J*=5.6, 2H, H_r), 2.38 – 2.46 (m, 2H, H_n), 1.52 – 1.69 (m, 10H, H_I, $H_{I'}$ and H_q), 0.75 - 1.41 (m, 28H, H_J, $H_{J'}$, H_K, $H_{K'}$,H_L, $H_{L'}$, H_h, H_i, H_j, H_k, H_l and H_m), 1.33 (s, 54H, H_a and H_w); ^{13}C NMR (150 MHz, $CDCl_3$) δ ppm 158.8 (C), 158.8 (C), 158.4 (2 x C), 156.9 (C), 156.3 (C), 155.6 (C), 155.5 (C), 148.4 (C), 148.3 (C), 148.0 (C), 144.5 (C), 144.4 (C), 139.6 (C), 138.9 (C), 137.3 (CH), 137.1 (CH), 132.0 (CH), 132.0 (CH), 130.9 (CH), 130.9 (CH), 129.9 (C), 129.8 (C), 129.8 (CH), 129.8 (CH), 124.2 (CH), 124.2 (CH), 121.6 (CH), 121.3 (CH), 120.8 (CH), 120.0 (CH), 119.7 (CH), 114.5 (2 x CH), 113.0 (CH), 112.9 (CH), 72.6 (CH_2), 72.4 (2 x CH_2), 72.2 (CH_2), 67.8 (2 x CH_2), 67.4 (CH_2), 64.0 (C), 63.2 (C), 46.7 (CH_2), 34.5 (2 x C), 31.6 (2 x CH_3), 29.9 (CH_2), 29.8 (CH_2), 29.8 (CH_2), 29.7 (CH_2), 29.7 (CH_2), 29.6 (CH_2), 29.6 (CH_2), 29.5 (CH_2), 29.2 (CH_2), 29.1 (2 x CH_2), 29.0 (CH_2), 29.0 (CH_2), 26.0 (CH_2), 26.0 (CH_2), 25.9 (2 x CH_2), 25.9 (CH_2); LRMS (ESI+) m/z 1188.3 $[M+2H]^{2+}$.

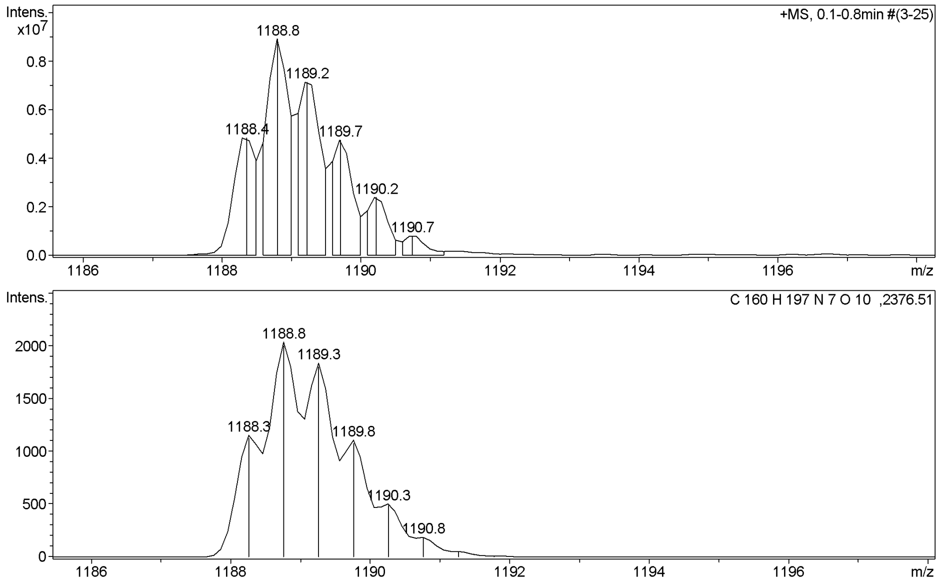

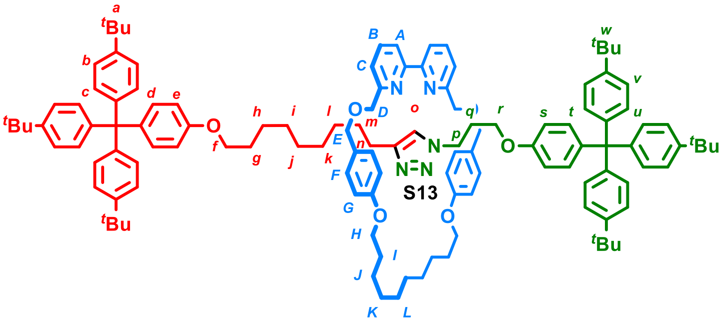

[2]Rotaxane **S13** was also isolated (21.4 mg, 47%) as a white foam: ^{1}H NMR (400 MHz, $CDCl_3$) δ ppm 8.01 (d, *J*=7.8, 2H, H_A), 7.53 (t, *J*=7.8, 2H, H_B), 7.32 (d, *J*=7.7, 2H, H_C), 7.25 (d, *J*=8.5, 6H, H_b or H_v), 7.24 (d, *J*=8.4, 6H, H_b or H_v), 7.10 (d, *J*=8.4, 6H, H_c or H_u), 7.10 (d, *J*=8.5, 6H, H_c or H_u), 6.98 - 7.07 (m, 8H, H_F, H_d and H_t), 6.92 (s, 1H, H_o), 6.61 (d, *J*=8.4, 4H, H_G), 6.60 (d, *J*=8.8, 2H, H_e), 6.46 (d, *J*=8.8, 2H, H_s), 4.58 (s, 4H, H_D or H_E), 4.58 (s, 4H, H_D or H_E), 4.03 (t, *J*=7.3, 2H, H_p), 3.78 (t, *J*=6.5, 4H, H_H), 3.67 (t, *J*=6.5, 2H, H_f), 3.49 (t, *J*=5.7, 2H, H_r), 2.50 (t, *J*=7.8, 2H, H_n), 1.84 - 1.93 (m, 2H, H_q), 1.39 – 1.66 (m, 8H, H_g, H_I, H_m), 1.04 - 1.39 (m, 20H, H_h, H_i, H_j, H_k, H_J, H_K, H_L), 1.32 (s, 54H, H_w); ^{13}C NMR (100 MHz, $CDCl_3$) δ ppm 158.8 (C), 158.4 (C), 157.1 (C), 156.4 (C), 155.6 (C), 148.5 (C), 148.4 (C), 148.2 (C), 144.4 (C), 144.3 (C), 139.9 (C), 139.3 (C), 137.2 (CH), 132.3 (CH), 132.2 (CH), 130.9 (CH), 130.9 (CH), 129.9 (C), 129.8 (CH), 124.3 (CH), 124.2 (CH), 121.5 (CH), 121.0 (CH), 119.9 (CH), 114.5 (CH), 113.1 (CH), 113.0 (CH), 72.5 (CH_2), 72.3 (CH_2), 67.8 (CH_2), 64.1 (CH_2), 63.2 (C), 63.2 (C), 46.8 (CH_2), 34.4 (2 x C), 31.6 (2 x CH_3), 30.0 (CH_2), 29.6 (2 x CH_2), 29.6 (CH_2), 29.5 (CH_2), 29.4 (2 x CH_2), 29.1 (CH_2), 29.0 (CH_2), 26.1 (CH_2), 26.0 (2 x CH_2), 25.8 (CH_2); LRMS (ESI+) 905.1 m/z $[M+2H]^{2+}$.

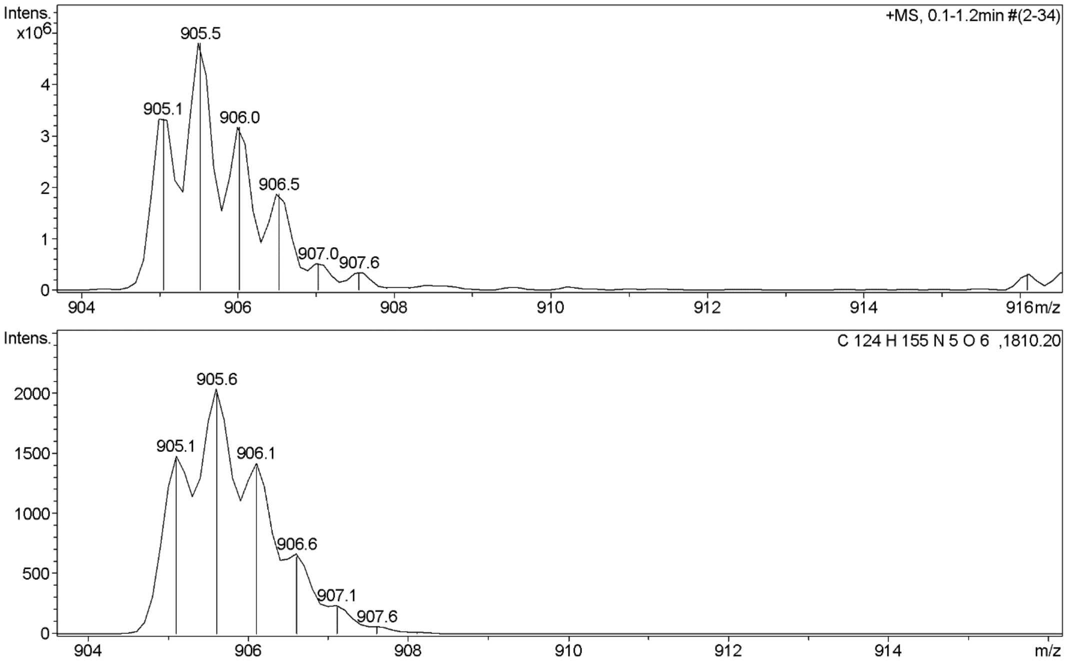

Synthesis of [2]Rotaxane S11d

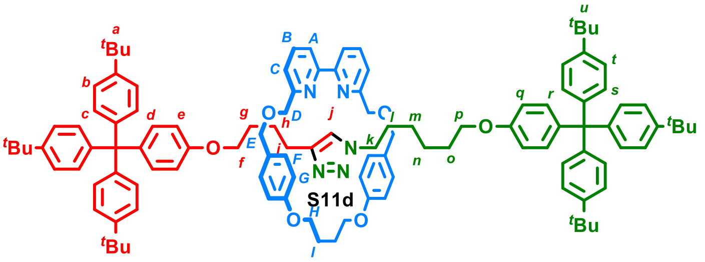

[2]Rotaxane **S11d** was made according to conditions B, using macrocycle **1d** (12.1 mg, 0.025 mmol), alkyne **7** (17.5 mg, 0.030 mmol), azide **6** (18.9 mg, 0.030 mmol) and $Cu(MeCN)_4.PF_6$ (8.9 mg, 0.024 mmol), in CH_2Cl_2 (2.5 mL) stirred at 100°C in a 150W microwave reactor for 1 hour. Flash column chromatography (0-30% MeCN/1:1 hexane:CH_2Cl_2 (+0.25% EtOH)) afforded 42.9 mg (93%) of the target material as a white foam: ^{1}H NMR (400 MHz, $CDCl_3$) δ ppm 7.62 (t, J=7.7, 2H, H_B), 7.63 (s, 1H, H_j), 7.44 (app. t, J=7.8, 4H, H_A and H_C), 7.22 - 7.26 (m, 12H, H_b and H_t), 7.03 - 7.14 (m, 16H, H_c, H_s, H_d and H_r), 6.97 (d, J=8.7, 4H, H_D), 6.63 - 6.72 (m, 8H, H_E, H_e and H_q), 4.42 - 4.60 (m, 4H, four of H_D/H_E), 4.27 - 4.35 (m, 2H, two of H_H), 4.19 - 4.27 (m, 4H, four of H_D/H_E), 4.02 - 4.09 (m, 2H, two of H_H), 3.79 (t, J=6.3, 2H, H_f), 3.62 (t, J=6.5, 2H, H_k), 3.16 - 3.23 (m, 2H, H_p), 2.56 (t, J=7.6, 2H, H_i), 1.97 - 2.11 (m, 2H, two of H_I), 1.82 - 1.97 (m, 2H, two of H_I), 1.63 - 1.71 (m, 2H, H_g), 1.52 - 1.64 (m, 2H, H_h), 1.31 (s, 27H, H_a or H_u), 1.31 (s, 27H, H_a or H_u), 0.78 - 0.86 (m, 2H, H_m), 0.65 - 0.74 (m, 2H, H_o), 0.52 - 0.59 (m, 2H, H_n); ^{13}C NMR (100 MHz, $CDCl_3$) δ ppm 159.3 (C), 159.0 (C), 157.1 (C), 157.0 (C), 156.5 (C), 148.4 (2 x C), 146.6 (C), 144.4 (C), 144.4 (C), 139.5 (C), 139.4 (C), 137.3 (CH), 132.3 (CH), 130.9 (2 x CH), 130.3 (CH), 129.1 (C), 124.2 (2 x CH), 122.1 (CH), 121.1 (CH), 121.0 (CH), 115.1 (CH), 113.1 (CH), 113.1 (CH), 72.9 (CH_2), 70.9 (CH_2), 67.5 (CH_2), 67.5 (CH_2), 66.6 (CH_2), 63.2 (C), 63.2 (C), 49.2 (CH_2), 34.4 (2 x C), 31.5 (2 x CH_3), 29.0 (CH_2), 28.8 (CH_2), 28.8 (CH_2), 25.9 (CH_2), 25.9 (CH_2), 25.5 (CH_2), 25.2 (CH_2), 24.8 (CH_2); LRMS (ESI+) m/z 848.9 $[M+2H]^{2+}$.

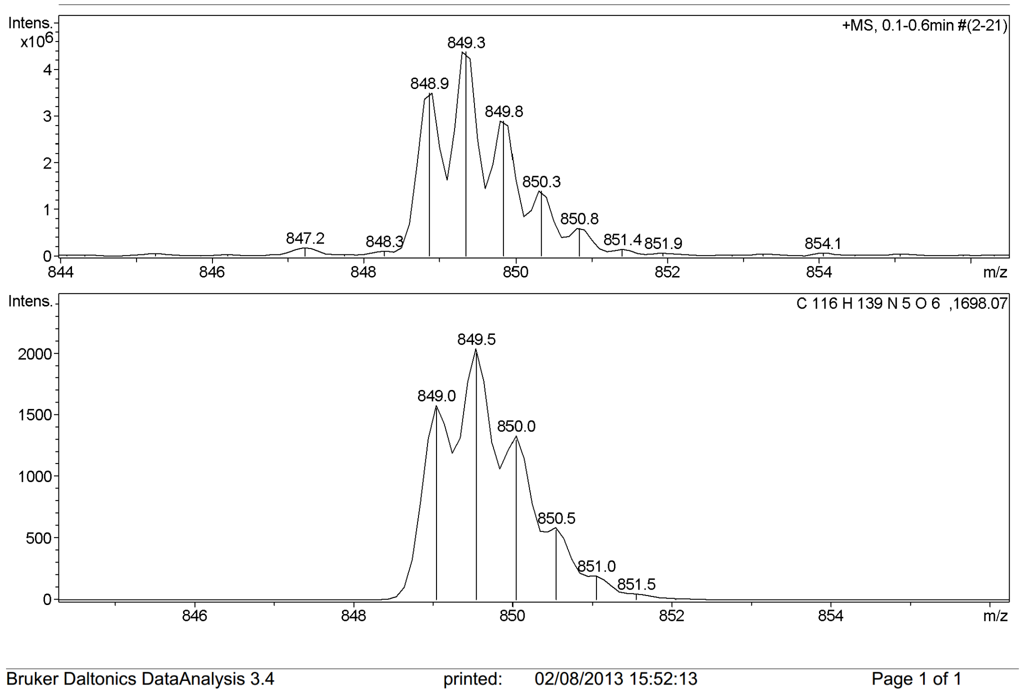

Synthesis of Thread Triazole S14

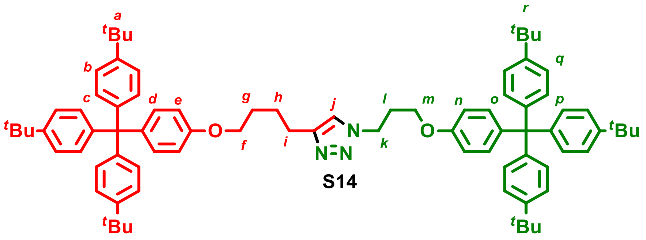

An analytical sample of triazole **S14** was isolated as a by-product of [3]rotaxane **S10** (*vide supra*). Flash column chromatography (0-30% MeCN/1:1 hexane:CH_2Cl_2 (+0.25% EtOH)) afforded triazole **S14** as a white foam: ^{1}H NMR (400 MHz, $CDCl_3$) δ ppm 7.30 (s, 1H, H_j), 7.23 (d, J=8.8, 6H, H_b or H_q), 7.23 (d, J=8.8, 6H, H_b or H_q), 7.10 (d, J=8.8, 2H, H_d or H_o), 7.08 (d, J=8.6, 6H, H_c or H_p), 7.08 (d, J=8.6, 6H, H_c or H_p), 7.06 (d, J=8.8, 2H, H_d or H_o), 6.74 (d, J=8.8, 2H, H_e or H_p), 6.72 (d, J=8.8, 2H, H_e or H_p), 4.54 (t, J=6.9, 2H, H_k), 3.94 (t, J=5.6, 2H, H_m), 3.94 (t, J=5.8, 2H, H_f), 2.79 (t, J=7.0, 2H, H_i), 2.36 (tt, J=6.9, 5.6, 2H, H_l), 1.78 - 1.90 (m, 4H, H_g and H_h), 1.30 (s, 27H, H_a or H_r), 1.30 (s, 27H, H_a or H_r); ^{13}C NMR (150 MHz, $CDCl_3$) δ ppm 156.9 (C), 156.4 (C), 148.5 (C), 148.4 (C), 148.0 (C), 144.3 (C), 144.2 (C), 140.3 (C), 139.6 (C), 132.5 (CH), 132.4 (CH), 130.9 (CH), 130.9 (CH), 124.2 (CH), 124.2 (CH), 121.4 (CH), 113.1 (CH), 113.1 (CH), 67.5 (CH_2), 64.1 (CH_2), 63.2 (2 x C), 47.1 (CH_2), 34.4 (2 x C), 31.5 (2 x CH_3), 30.2 (CH_2), 29.0 (CH_2), 26.2 (CH_2), 25.5 (CH_2); LRMS (ESI+) m/z 1172.8 $[M+H]^+$.

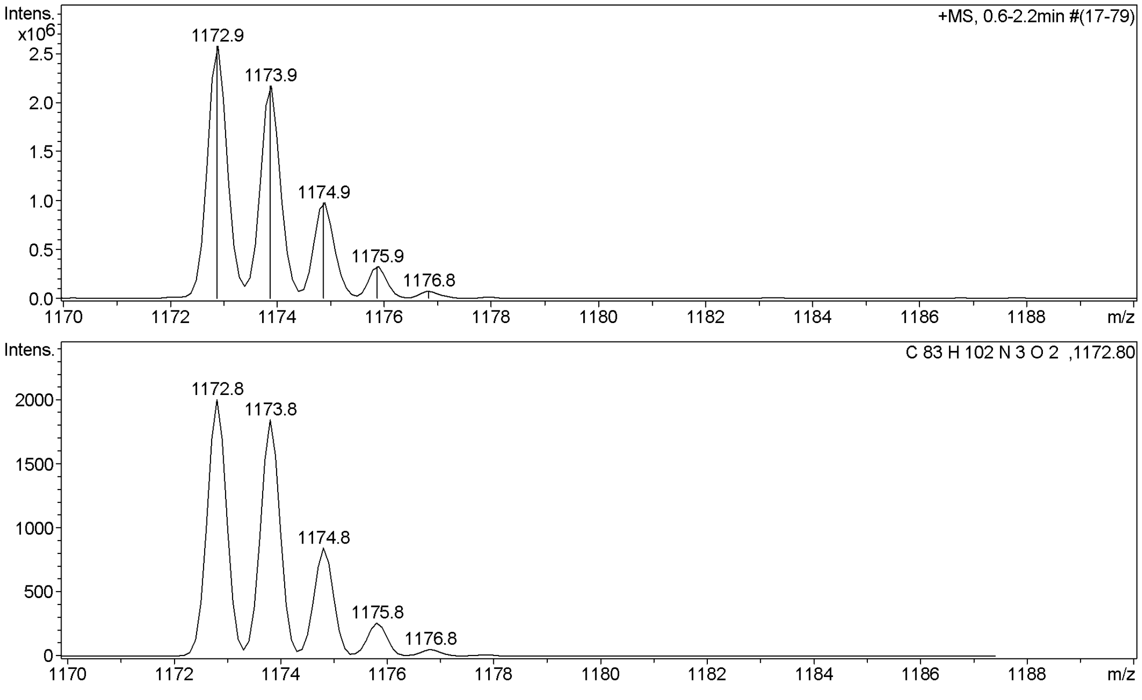

Synthesis of Thread Triazole S15

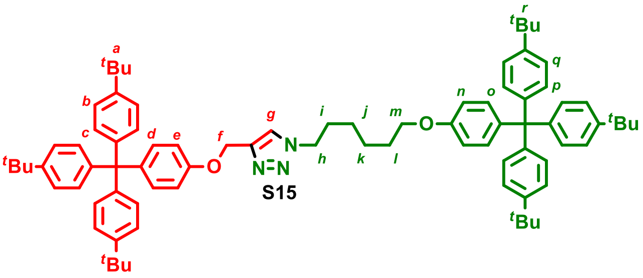

An analytical sample of triazole **S15** was isolated as a by-product of [3]rotaxane **S11** (*vide supra*). Flash column chromatography (0-30% MeCN/1:1 hexane:CH_2Cl_2 (+0.25% EtOH)) afforded triazole **S15** as a white foam: ^{1}H NMR (400 MHz, $CDCl_3$) δ ppm 7.59 (s, 1H, H_g), 7.24 (d, *J*=8.7, 6H, H_b or H_q), 7.23 (d, *J*=8.7, 6H, H_b or H_q), 7.08 (d, *J*=8.7, 6H, H_c or H_p), 7.08 (d, *J*=8.8, 6H, H_c or H_p), 7.03 - 7.14 (m, 4H, H_d and H_o), 6.85 (d, *J*=9.0, 2H, H_e or H_n), 6.74 (d, *J*=9.0, 2H, H_e or H_n), 5.18 (s, 2H, H_f), 4.38 (t, *J*=7.2, 2H, H_h), 3.92 (t, *J*=6.3, 2H, H_m), 1.96 (tt, *J*=7.2, 7.6, 2H, H_i), 1.72 - 1.81 (m, 2H, H_l), 1.48 – 1.59 (m, 2H, H_j), 1.37 - 1.47 (m, 2H, H_k), 1.31 (s, 27H, H_a or H_r), 1.28 (s, 27H, H_a or H_r); ^{13}C NMR (150 MHz, $CDCl_3$) δ ppm 157.0 (C), 156.3 (C), 148.5 (C), 148.5 (C), 144.6 (C), 144.3 (C), 144.2 (C), 140.3 (C), 139.7 (C), 132.5 (CH), 132.4 (CH), 130.9 (2 x CH), 124.2 (CH), 124.2 (CH), 122.5 (CH), 113.4 (CH), 113.1 (CH), 67.5 (CH_2), 63.2 (2 x C), 62.3 (CH_2), 50.5 (CH_2), 34.4 (2 x C), 31.5 (2 x CH_3), 30.4 (CH_2), 29.2 (CH_2), 26.4 (CH_2), 25.8 (CH_2); LRMS (ESI+) m/z 1194.8 $[M+Na]^+$.

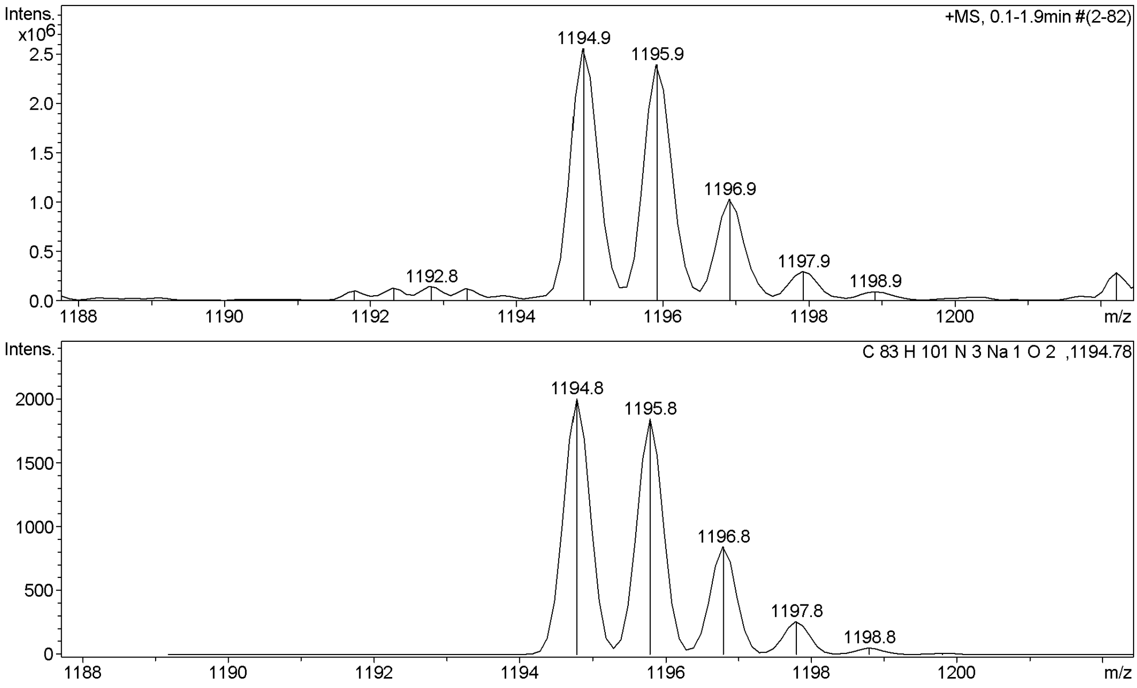

Synthesis of Thread Triazole S16

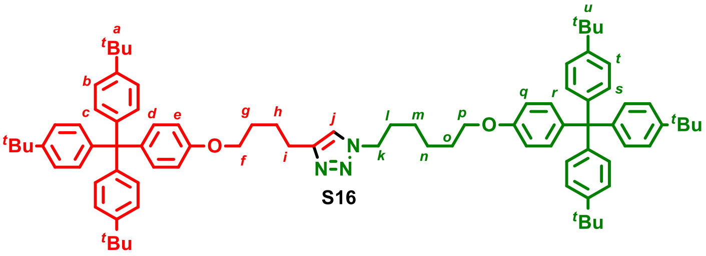

A solution of alkyne **7** (14.6 mg, 0.025 mmol), azide **6** (15.7 mg, 0.025 mmol) and $Cu(MeCN)_4.PF_6$ (8.4 mg, 0.0225 mmol), in CH_2Cl_2 (2.5 mL) was stirred at 25 °C for 72 hours in the absence of macrocycle. The solution was diluted with further CH_2Cl_2 (50 mL) and washed with 16% aqueous EDTA tetrasodium-saturated ammonia solution (50 mL). The organic layer was retained and the aqueous layer extracted twice further with CH_2Cl_2 (50 mL portions). The organic extracts were combined, dried over $MgSO_4$ and dried *in vacuo*. Flash column chromatography (0-50% CH_2Cl_2/petrol) afforded 23.0 mg (76%) of triazole **S16** as a white foam: ^{1}H NMR (600 MHz, $CDCl_3$) δ ppm 7.28 (s, 1H, H_j), 7.21 - 7.25 (d, J=8.7, 12H, H_b and H_t), 7.06 - 7.11 (m, 16H, H_c, H_s, H_d and H_r), 6.75 (d, J=9.1, 2H, H_e or H_q), 6.74 (d, J=9.1, 2H, H_e or H_q), 4.32 (t, J=7.2, 2H, H_k), 3.96 (t, J=5.9, 2H, H_f), 3.92 (t, J=6.3, 2H, H_p), 2.80 (t, J=7.1, 2H, H_i), 1.93 (tt, J=7.8, 7.2, 2H, H_l), 1.81 - 1.89 (m, 4H, H_g and H_h), 1.73 - 1.81 (m, 2H, H_o), 1.48 - 1.56 (m, 2H, H_n), 1.34 - 1.43 (m, 2H, H_m), 1.31 (s, 54H, H_a and H_u); ^{13}C NMR (150 MHz, $CDCl_3$) δ ppm 157.0 (C), 148.4 (C), 148.4 (C), 148.1 (C), 144.3 (2 x C), 139.7 (C), 139.6 (C), 132.4 (2 x CH), 130.9 (2 x CH), 124.2 (2 x CH), 120.6 (CH), 113.1 (2 x CH), 67.6 (CH_2), 67.5 (CH_2), 63.2 (2 x C), 50.2 (CH_2), 34.4 (2 x C), 31.5 (2 x CH_3), 30.4 (CH_2), 29.3 (CH_2), 29.0 (CH_2), 26.5 (CH_2), 26.2 (CH_2), 25.8 (CH_2), 25.6 (CH_2); LRMS (ESI+) m/z 1236.8 $[M+Na]^+$.

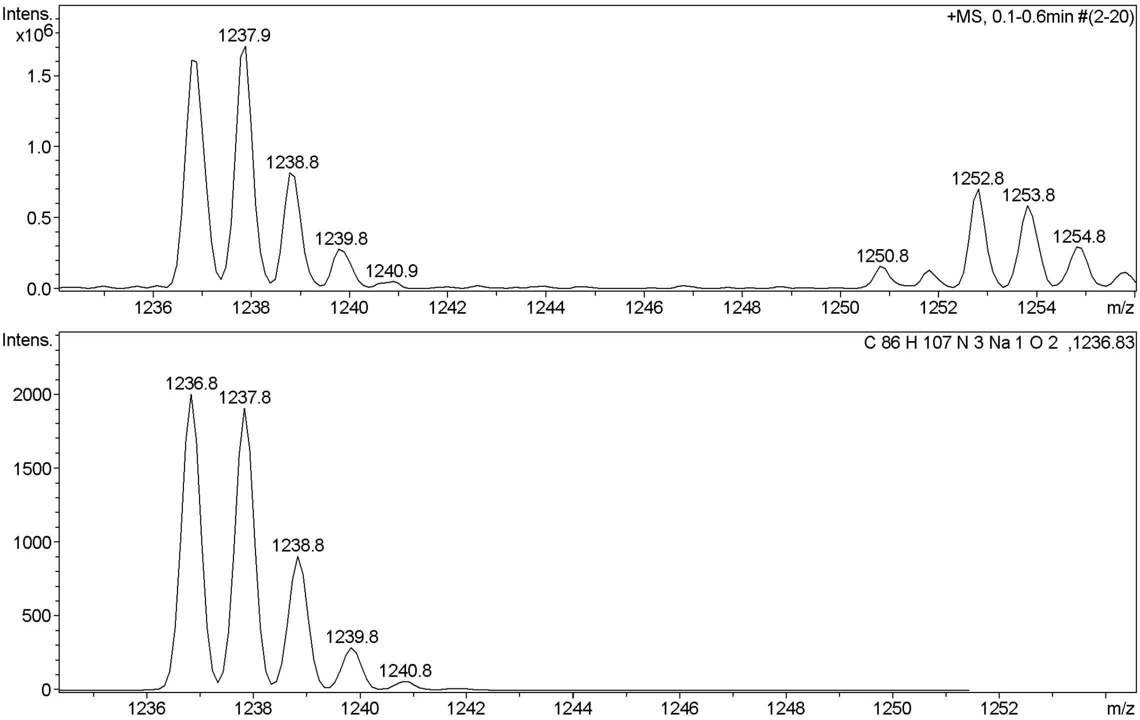

Synthesis of Thread Triazole S17

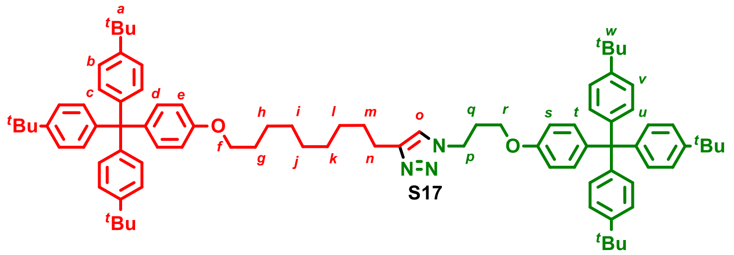

An analytical sample of triazole **S17** was isolated as a by-product of [3]rotaxane **S13** (*vide supra*). Flash column chromatography (0-30% MeCN/1:1 hexane:CH_2Cl_2 (+0.25% EtOH)) afforded triazole **S17** as a white foam: ^{1}H NMR (400 MHz, $CDCl_3$) δ ppm 7.27 (s, 1H, H_o), 7.24 (d, *J*=8.5, 6H, H_b or H_v), 7.24 (d, *J*=8.7, 6H, H_b or H_v), 7.09 (m, 16H, H_c, H_v, H_d and H_t), 6.75 (d, *J*=8.8, 4H, H_e and H_s), 4.54 (t, *J*=6.8, 2H, H_p), 3.95 (t, *J*=5.4, 2H, H_r), 3.92 (t, *J*=6.4, 2H, H_f), 2.71 (t, *J*=7.7, 2H, H_n), 2.37 (tt, *J*=6.8, 5.4, 2H, H_q), 1.76 (tt, *J*=7.7, 6.7, 2H, H_m), 1.66 (m, 2H, H_g), 1.13 - 1.49 (m, 10H, H_h, H_i, H_j, H_k and H_l), 1.31 (s, 54H, H_a and H_w); ^{13}C NMR (100 MHz, $CDCl_3$) δ ppm 157.1 (C), 156.5 (C), 148.6 (C), 148.5 (C), 148.4 (C), 144.4 (C), 144.2 (C), 140.3 (C), 139.5 (C), 132.5 (CH), 132.4 (2 x CH), 130.9 (CH), 130.9 (CH), 124.2 (CH), 124.2 (CH), 121.2 (CH), 113.1 (2 x CH), 68.0 (CH_2), 64.1 (CH_2), 63.2 (C), 63.2 (C), 47.1 (CH_2), 34.4 (6 x C), 31.5 (18 x CH_3), 30.2 (CH_2), 29.6 (CH_2), 29.6 (CH_2), 29.5 (CH_2), 29.5 (CH_2), 29.5 (CH_2), 29.4 (CH_2), 26.2 (CH_2), 25.8 (CH_2); LRMS (ESI+) m/z 1242.8 $[M+H]^+$.

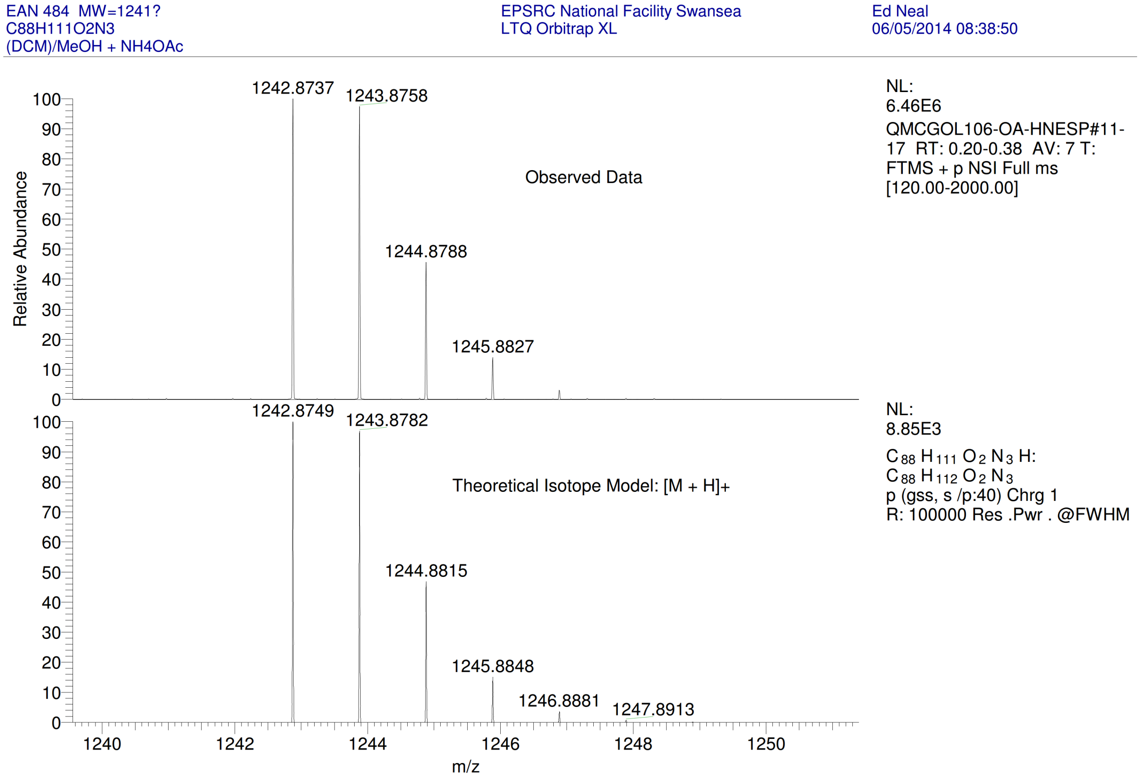

Synthesis of Macrocycle 9a

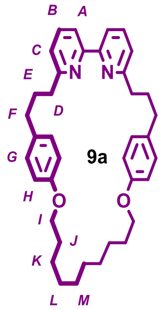

To a solution of bisphenol **S18** (1 g, 2.36 mmol) in DMF (1 L) were added 1,10-dibromodecane (0.707 g, 2.36 mmol) and K_2CO_3 (1.628 g, 11.8 mmol, 5 eq). The solution was stirred at 60 °C for 72 hours and dried *in vacuo*. The residue was twice extracted with Et_2O in 250 mL portions, with the extracts dried over $MgSO_4$ and dried *in vacuo*. Flash column chromatography (0-10% MeCN/1:1 petrol:CH_2Cl_2; 0-20% Et_2O/petrol) afforded 164 mg (12%) of macrocycle **9a** as a white solid: m.p. 78-80 °C; ^1H NMR (400 MHz, $CDCl_3$) δ ppm 8.02 (d, J = 7.7, 2H, H_A), 7.64 (t, J = 7.7, 2H, H_B), 7.08 (d, J = 7.7, 2H, H_C), 6.99 (d, J = 7.9, 4H, H_G), 6.71 (d, J = 7.9, 4H, H_H), 3.91 (t, J = 5.9, 4H, H_I), 2.91 (t, J = 6.8, 4H, H_D), 2.62 (t, J = 7.5, 4H, H_F), 2.09 (tt, J = 7.5, 6.8, 4H, H_E), 1.68 (tt, J = 7.1, 5.9, 4H, H_J), 1.37 (br. s., 4H, H_K), 1.24 (br. s., 8H, H_L and H_M); ^{13}C NMR δ ppm (100 MHz, $CDCl_3$) δ 161.7 (C), 157.1 (C), 156.7 (C), 136.8 (CH), 134.6 (C), 129.4 (CH), 122.6 (CH), 118.8 (CH), 114.8 (CH), 68.0 (CH_2), 38.1 (CH_2), 34.6 (CH_2), 32.1 (CH_2), 29.4 (CH_2), 28.9 (CH_2), 28.9 (CH_2), 25.9 (CH_2); HRMS (ESI+) m/z 563.3624 $[M+H]^+$ (calc. for $C_{38}H_{47}N_2O_2$ 563.3632).

Synthesis of Macrocycle 9b

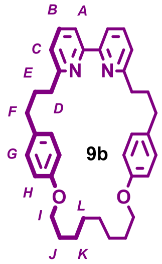

Macrocycle **9b** was made using the same method as macrocycle **9a** except using 1,8-dibromooctane (0.641 g, 0.45 mL, 2.36 mmol) to afford 187 mg (15%) of macrocycle **9b** as a white solid: m.p. 120-123 °C; ^1H NMR (400 MHz, $CDCl_3$) δ ppm 7.91 (d, J = 7.7, 2H, H_A), 7.62 (t, J = 7.7, 2H, H_B), 7.07 (d, J = 7.7, 2H, H_C), 6.98 (d, J = 8.6, 4H, H_G), 6.69 (d, J = 8.6, 4H, H_H), 3.91 (t, J = 6.3, 4H, H_I), 2.91 (t, J = 7.2, 4H, H_D), 2.62 (dd, J = 8.2, 7.4, 4H, H_F), 2.08 (ddt, J = 8.2, 7.4, 7.2, 4H, H_E), 1.66 (tt, J = 7.3, 6.3, 4H, H_J), 1.37 (tt, J = 7.3, 6, 4H, H_K), 1.20 - 1.32 (m, 4H, H_L); ^{13}C NMR (100 MHz, $CDCl_3$) δ ppm 161.8 (C), 157.0 (C), 157.0 (C), 136.8 (CH), 134.6 (C), 129.4 (CH), 122.5 (CH), 119.0 (CH), 114.8 (CH), 67.9 (CH_2), 38.1 (CH_2), 34.5 (CH_2), 32.1 (CH_2), 28.9 (CH_2), 28.8 (CH_2), 25.7 (CH_2); HRMS (ESI+) m/z 535.3312 $[M+H]^+$ (calc. for $C_{36}H_{43}N_2O_2$ 535.3319).

Synthesis of Macrocycle 9c

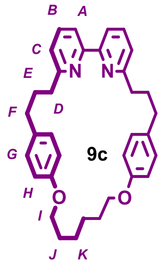

Macrocycle **9c** was made using the same method as macrocycle **9a** except using 1,6-dibromohexane (0.575 g, 0.36 mL, 2.36 mmol) to afford 120 mg (10%) of macrocycle **9c** as a white solid: m.p. 121-125 °C; ^{1}H NMR (400 MHz, $CDCl_3$) δ ppm 7.75 (d, *J* = 7.8, 2H, H_A), 7.61 (t, *J* = 7.8, 2H, H_B), 7.07 (d, *J* = 7.8, 2H, H_C), 6.98 (d, *J* = 8.4, 4H, H_G), 6.68 (d, *J* = 8.4, 4H, H_H), 3.94 (t, *J* = 6.1, 4H, H_I), 2.91 (t, *J* = 7.5, 4H, H_D), 2.64 (t, *J* = 7.5, 4H, H_F), 2.12 (app. quin, *J* = 7.5, 4H, H_E), 1.72 (app. quin, *J* = 6.1, 4H, H_J), 1.48 (m, 4H, H_K); ^{13}C NMR (100 MHz, $CDCl_3$) δ ppm 162.1 (C), 157.2 (C), 157.2 (C), 136.7 (CH), 134.5 (C), 129.5 (CH), 122.4 (CH), 119.2 (CH), 114.9 (CH), 67.9 (CH_2), 37.9 (CH_2), 34.6 (CH_2), 31.6 (CH_2), 28.5 (CH_2), 25.2 (CH_2); HRMS (ESI+) m/z 507.3000 $[M+H]^+$ (calc. for $C_{34}H_{39}N_2O_2$ 507.3006).

Synthesis of [2]Rotaxane 10a

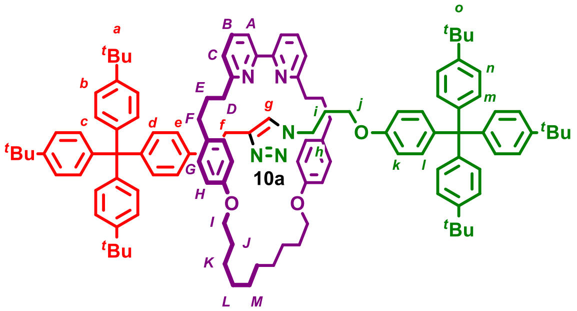

[2]Rotaxane **10a** was made according to condition B (except at 0.05 M), using macrocycle **9a** (14.1 mg, 0.025 mmol), azide **3** (17.6 mg, 0.030 mmol), alkyne **2** (16.3 mg, 0.030 mmol) and $Cu(MeCN)_4.PF_6$ (8.9 mg, 0.024 mmol), in CH_2Cl_2 (0.5 mL) stirred at 100°C in a 150W microwave reactor for 15 minutes. Flash column chromatography (0-30% MeCN/1:1 hexane:CH_2Cl_2 (+0.25% EtOH)) afforded 40.5 mg (96%) of [2]rotaxane **10a** as a white solid: m.p. 114-116 °C; ^{1}H NMR (400 MHz, $CDCl_3$) δ ppm 7.94 (d, J = 7.8, 2H, H_A), 7.40 (t, J = 7.8, 2H, H_B), 7.26 (d, J = 8.7, 6H, H_b or H_n), 7.24 (d, J = 8.7, 6H, H_b or H_n), 7.10 (d, J = 8.7, 6H, H_c or H_m), 7.08 (d, J = 8.7, 6H, H_c or H_m), 7.04 (d, J = 8.9, 2H, H_d), 7.00 (s, 1H, H_g), 6.92 (d, J = 8.8, 2H, H_l), 6.92 (d, J = 7.8, 2H, H_C), 6.72 (d, J = 8.6, 4H, H_G), 6.70 (d, J = 8.9, 2H, H_e), 6.50 (d, J = 8.6, 4H, H_H), 6.26 (d, J = 8.8, 2H, H_k), 4.88 (s, 2H, H_f), 3.79 (t, J = 7.3, 2H, H_h), 3.76 (t, J = 6.7, 4H, H_I), 3.24 (t, J = 5.7, 2H, H_j), 2.80 (t, J = 7.6, 4H, H_D), 2.48 (t, J = 7.6, 4H, H_F), 1.87 (quin., J = 7.6, 4H, H_E), 1.65 (tt, J = 7.3, 5.7, 2H, H_i), 1.57 (tt, J = 7.1, 6.7, 4H, H_J), 1.33 (s, 27H, H_a or H_o), 1.31 (s, 27H, H_a or H_o), 1.22 - 1.28 (m, 4H, H_K), 1.08 - 1.19 (m, 8H, H_L and H_M); ^{13}C NMR (100 MHz, $CDCl_3$) δ ppm 161.6 (C), 157.0 (C), 156.7 (C), 156.4 (C), 156.2 (C), 148.4 (2 x C), 144.4 (C), 144.3 (C), 143.8 (C), 140.0 (C), 139.7 (C), 137.0 (CH), 134.2 (C), 132.3 (CH), 132.1 (CH), 130.9 (2 x CH), 129.2 (CH), 124.2 (CH), 124.2 (CH), 123.1 (CH), 122.7 (CH), 118.9 (CH), 114.6 (CH), 113.3 (CH), 112.9 (CH), 67.8 (CH_2), 63.6 (CH_2), 63.2 (C), 63.2 (C), 61.8 (CH_2), 46.9 (CH_2), 38.0 (CH_2), 34.5 (C), 34.4 (C), 34.4 (CH_2), 32.3 (CH_2), 31.6 (CH_3), 31.5 (CH_3), 29.7 (CH_2), 29.6 (CH_2), 29.0 (CH_2), 28.8 (CH_2), 25.7 (CH_2); LRMS (ESI+) 1693 m/z $[M+H]^+$.

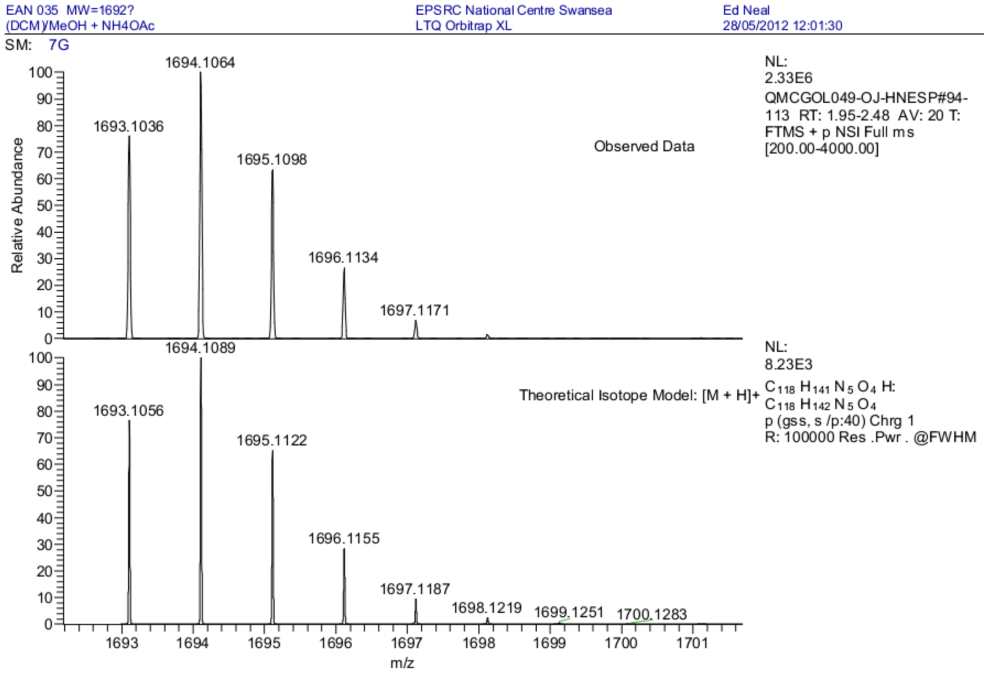

Synthesis of [2]Rotaxane 10b

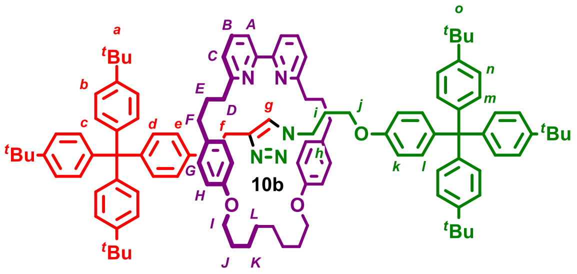

[2]Rotaxane **10b** was made according to condition B (except at 0.05 M), using macrocycle **9b** (13.4 mg, 0.025 mmol), azide **3** (17.6 mg, 0.030 mmol), alkyne **2** (16.3 mg, 0.030 mmol) and $Cu(MeCN)_4.PF_6$ (8.9 mg, 0.024 mmol), in CH_2Cl_2 (0.5 mL) stirred at 100°C in a 150W microwave reactor for 15 minutes. Flash column chromatography (0-30% MeCN/1:1 hexane:CH_2Cl_2 (+0.25% EtOH)) afforded 39.2 mg (95%) of the target material as a white solid: m.p. 128-130 °C; ^{1}H NMR (400 MHz, $CDCl_3$) δ ppm 7.80 (d, J = 7.8, 2H, H_A), 7.40 (t, J = 7.8, 2H, H_B), 7.27 (d, J = 8.3, 6H, H_b or H_n), 7.23 (d, J = 8.6, 6H, H_b or H_n), 7.11 (d, J = 8.8, 6H, H_m or H_c), 7.09 (d, J = 8.8, 6H, H_m or H_c), 7.08 (s, 1H, H_g), 7.03 (d, J = 8.8, 2H, H_d), 6.94 (d, J = 7.8, 2H, H_C), 6.89 (d, J = 8.8, 2H, H_l), 6.70 (d, J = 8.8, 2H, H_e), 6.69 (d, J = 8.6, 4H, H_G), 6.48 (d, J = 8.6, 4H, H_H), 6.18 (d, J = 8.8, 2H, H_k), 4.89 (s, 2H, H_f), 3.77 (t, J = 6.8, 4H, H_I), 3.64 (t, J = 7.2, 2H, H_h), 3.10 (t, J = 5.7, 2H, H_j), 2.78 (t, J = 7.5, 4H, H_D), 2.45 (t, J = 7.8, 4H, H_F), 1.79 (ttd, J = 7.8, 7.5, 3.7, 4H, H_E), 1.55 (m, 4H, H_J), 1.33 (s, 27H, H_a or H_o), 1.31 (s, 27H, H_a or H_o), 1.20 - 1.26 (m, 4H, H_K), 1.46 - 1.53 (m, 4H, H_i), 1.11 - 1.19 (m, 4H, H_L); ^{13}C NMR (100 MHz, $CDCl_3$) δ ppm 161.8 (C), 157.2 (C), 156.9 (C), 156.4 (C), 156.1 (C), 148.4 (C), 148.4 (C), 144.4 (C), 144.3 (2 x C), 143.7 (CH), 140.0 (C), 139.5 (C), 136.9 (CH), 134.1 (C), 132.3 (CH), 132.0 (CH), 130.9 (2 x CH), 129.1 (CH), 124.0 (CH), 124.0 (CH), 123.2 (CH), 122.4 (CH), 119.3 (CH), 114.7 (CH), 113.3 (CH), 112.9 (CH), 67.7 (CH_2), 63.5 (CH_2), 63.2 (C) 63.2 (C), 61.8 (CH_2), 46.8 (CH_2), 38.0 (CH_2), 34.5 (C), 34.4 (C), 34.4 (CH_2), 32.6 (CH_2), 31.6 (CH_3), 31.5 (CH_3), 29.4 (CH_2), 29.3 (CH_2), 28.9 (CH_2), 26.0 (CH_2); LRMS (ESI+) 1665 m/z $[M+H]^+$.

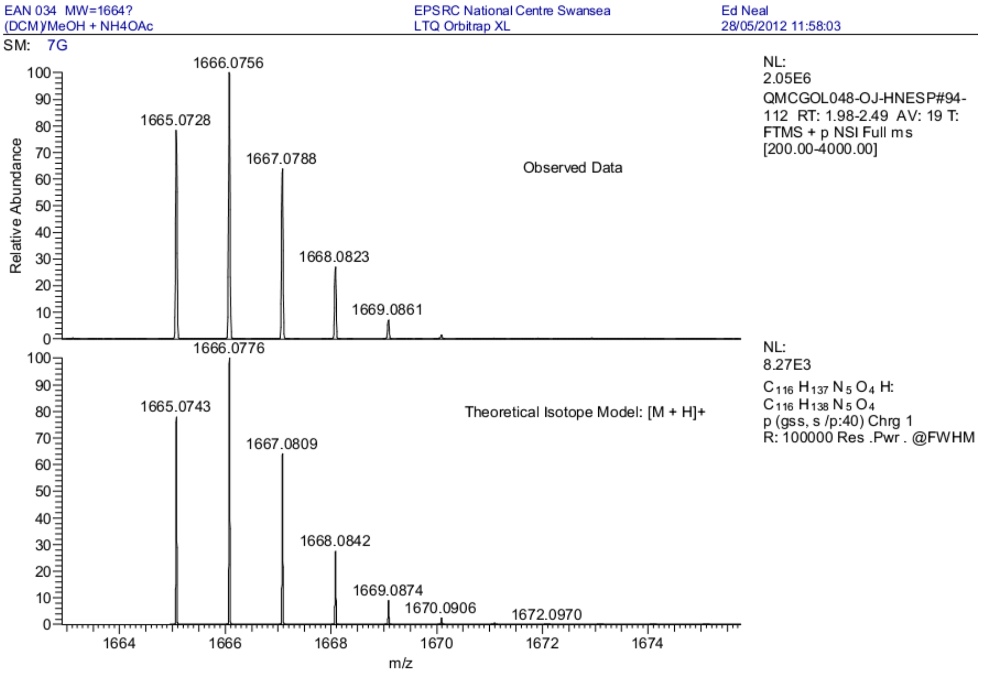

Synthesis of [2]Rotaxane 10c

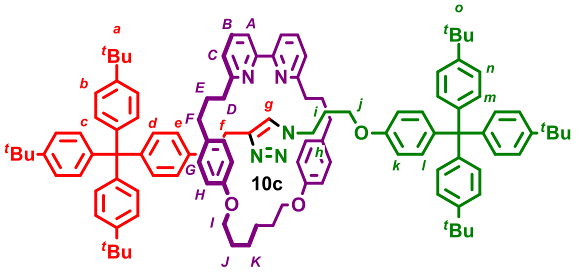
10c

[2]Rotaxane **10c** was made according to conditions B (except at 0.05 M), using macrocycle **9c** (12.7 mg, 0.025 mmol), azide **3** (17.6 mg, 0.030 mmol), alkyne **2** (16.3 mg, 0.030 mmol) and Cu(MeCN)$_4$.PF$_6$ (8.9 mg, 0.024 mmol), in CH_2Cl_2 (0.5 mL) stirred at 100°C in a 150W microwave reactor for 15 minutes. Flash column chromatography (0-30% MeCN/1:1 hexane:CH_2Cl_2 (+0.25% EtOH)) afforded 40.2 mg (95%) of the target material as a white solid: m.p. 135-139 °C; ^{1}H NMR (400 MHz, $CDCl_3$) δ ppm 7.52 (s, 1H, H$_g$), 7.52 (d, J = 7.8, 2H, H$_A$), 7.43 (t, J = 7.8, 2H, H$_B$), 7.28 (d, J = 8.6, 6H, H$_b$ or H$_n$), 7.23 (d, J = 8.6, 6H, H$_b$ or H$_n$), 7.13 (d, J = 8.6, 6H, H$_c$ or H$_m$), 7.09 (d, J = 8.6, 6H, H$_c$ or H$_m$), 7.04 (d, J = 8.9, 2H, H$_d$), 6.97 (d, J = 7.8, 2H, H$_C$), 6.90 (d, J = 8.9, 2H, H$_l$), 6.72 (d, J = 8.9, 2H, H$_e$), 6.66 (d, J = 8.5, 4H, H$_G$), 6.49 (d, J = 8.5, 4H, H$_H$), 6.21 (d, J = 8.9, 2H, H$_k$), 4.89 (s, 2H, H$_f$), 3.86 (dtt, J = 11.3, 10.2, 6.4, 4H, H$_I$), 3.44 (t, J = 7.5, 2H, H$_h$), 3.03 (t, J = 5.8, 2H, H$_j$), 2.69 (t, J = 7.8, 4H, H$_D$), 2.46 (t, J = 7.5, 4H, H$_F$), 1.68 (tt, J = 7.8, 7.5, 4H, H$_E$), 1.63 (m, 4H, H$_J$), 1.33 (s, 27H, H$_a$ or H$_o$), 1.31 (s, 27H, H$_a$ or H$_o$), 1.25 - 1.29 (m, 6H, H$_K$ and H$_i$); ^{13}C NMR (100 MHz, $CDCl_3$) δ ppm 162.2 (C), 158.0 (C), 157.1 (C), 156.4 (C), 156.2 (C), 148.4 (C), 148.4 (C), 144.4 (C), 144.3 (C), 143.5 (C), 139.9 (C), 139.3 (C), 136.9 (CH), 133.9 (C), 132.3 (CH), 131.9 (CH), 130.9 (2 x CH), 129.2 (CH), 124.2 (CH), 124.2 (CH), 123.7 (CH), 122.0 (CH), 119.9 (CH), 114.9 (CH), 113.3 (CH), 112.9 (CH), 67.8 (CH_2), 63.7 (CH_2), 63.2 (C), 63.2 (C), 61.8 (CH_2), 46.5 (CH_2), 37.8 (CH_2), 34.5 (C), 34.5 (CH_2), 34.4 (C), 32.7 (CH_2), 31.6 (CH_3), 31.5 (CH_3), 29.0 (CH_2), 29.0 (CH_2), 25.5 (CH_2); LRMS (ESI+) 1637 m/z $[M+H]^+$.

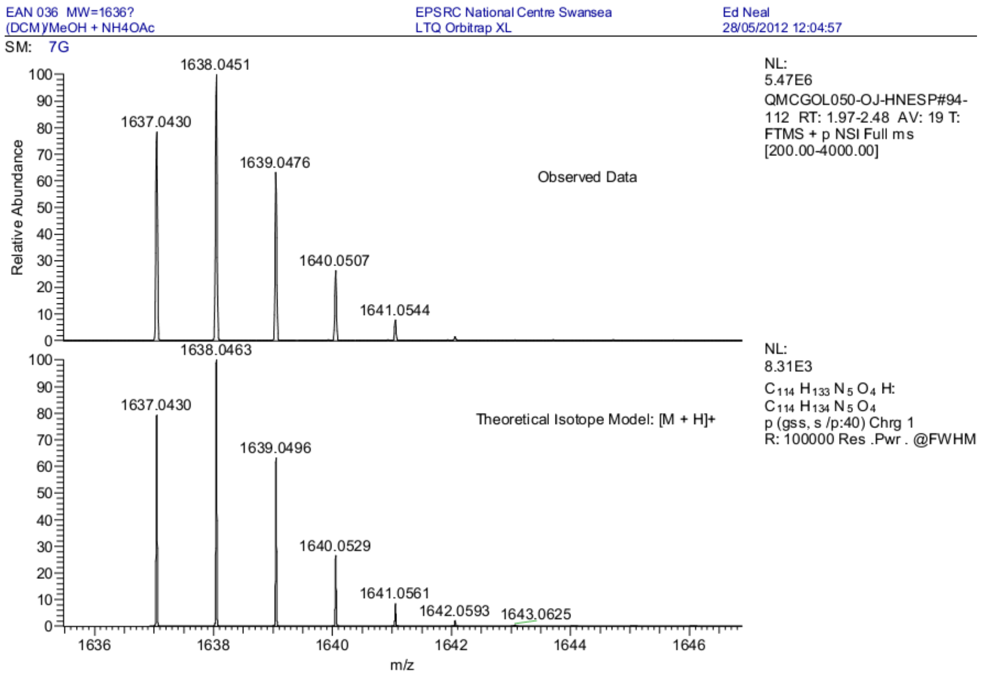

Synthesis of [2]Rotaxane 10d

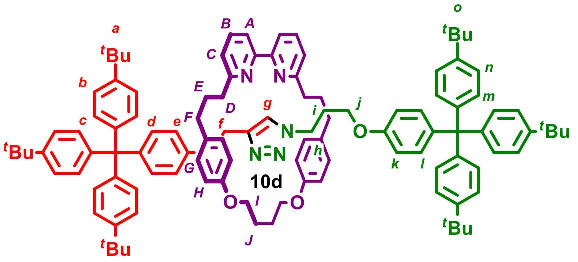

[2]Rotaxane **10d** was made according to conditions B (except at 0.05 M), using macrocycle **9d** (12.0 mg, 0.025 mmol), azide **3** (17.6 mg, 0.030 mmol), alkyne **2** (16.3 mg, 0.030 mmol) and Cu(MeCN)$_4$.PF$_6$ (8.9 mg, 0.024 mmol), in CH_2Cl_2 (0.5 mL) stirred at 100°C in a 150W microwave reactor for 15 minutes. Flash column chromatography (0-30% MeCN/1:1 hexane:CH_2Cl_2 (+0.25% EtOH)) afforded 42.4 mg (>99%) of the target material as a white solid; m.p. 151-154 °C; ^{1}H NMR (400 MHz, $CDCl_3$) δ ppm 8.42 (s, 1H, H_g), 7.53 (t, J = 7.7, 2H, H_B), 7.32 (d, J = 7.7, 2H, H_A), 7.28 (d, J = 8.7, 6H, H_b` and $H_n$`), 7.23 (d, J = 8.7, 6H, H_b and H_n), 7.13 (d, J = 8.7, 6H, H_c` and $H_m$`), 7.09 (d, J = 8.7, 6H, H_c and H_m), 7.03 (d, J = 9.1, 2H, H_d), 7.03 (d, J = 7.7, 2H, H_C), 6.97 (d, J = 8.9, 2H, H_l), 6.76 (d, J = 9.1, 2H, H_e), 6.70 (d, J = 8.7, 4H, H_G), 6.63 (d, J = 8.7, 4H, H_H), 6.45 (d, J = 8.9, 2H, H_k), 4.90 (s, 2H, H_f), 4.31 (dd, J = 16.3, 6.9, 2H, H_I`), 3.98 - 4.11 (m, 2H, $H_I$), 3.38 - 3.45 (m, 2H, $H_h$), 3.37 (t, $J$ = 6.1, 2H, $H_j$), 2.51 (td, $J$ = 12.5, 5.3, 2H, $H_D$`), 2.43 (td, J = 12.5, 5.3, 2H, H_D), 2.37 - 2.62 (m, 4H, H_F and H_F`), 2.09 - 2.21 (m, 2H, $H_J$`), 1.88 - 2.01 (m, 2H, H_J), 1.62 - 1.83 (m, 1H, H_E and H_E`), 1.28 - 1.39 (m, 54H, H_a and H_o), 1.16 - 1.24 (m, 2H, H_i); ^{13}C NMR (100 MHz, $CDCl_3$) δ ppm 162.7 (C), 157.8 (C), 157.5 (C), 156.6 (C), 156.4 (C), 148.4 (2 x C), 144.4 (C), 144.3 (C), 142.6 (C), 139.8 (C), 139.1 (C), 136.8 (CH), 133.2 (C), 132.2 (CH), 132.0 (CH), 130.9 (CH), 130.8 (CH), 129.5 (CH), 125.1 (CH), 124.2 (CH), 124.1 (CH), 121.6 (CH), 120.3 (CH), 115.0 (CH), 113.3 (CH), 113.0 (CH), 66.6 (CH_2), 64.6 (CH_2), 63.2 (C), 63.2 (C), 61.7 (CH_2), 46.7 (CH_2), 37.2 (CH_2), 35.0 (CH_2), 34.4 (C), 34.4 (C), 32.1 (CH_2), 31.6 (CH_3), 31.5 (CH_3), 28.6 (CH_2), 24.9 (CH_2); LRMS (ESI+) 1609 m/z $[M+H]^+$.

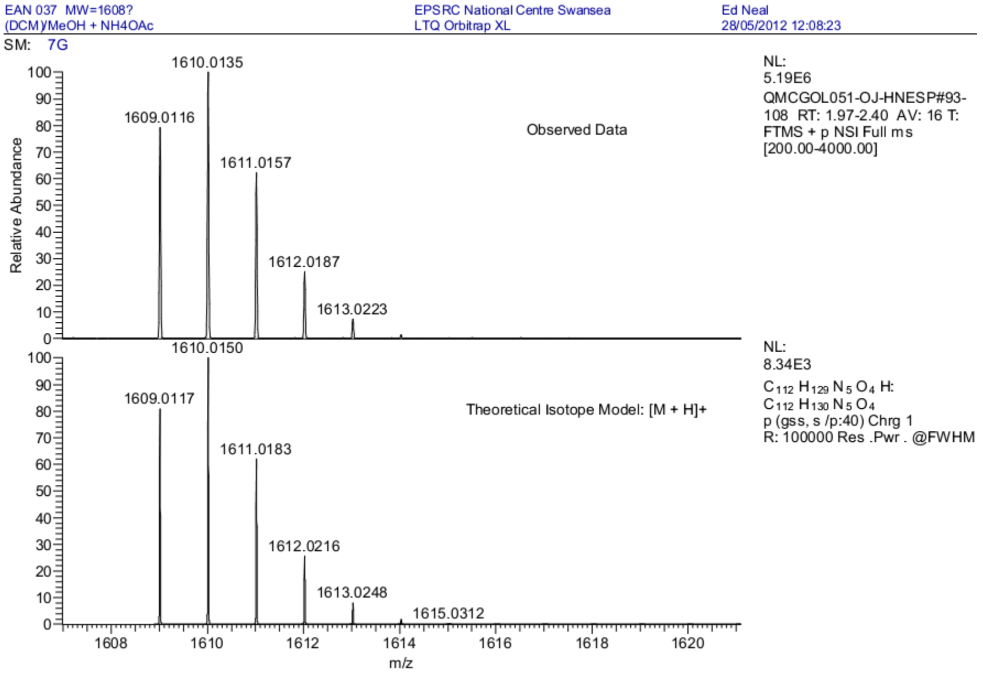

Lithiation Experiments (Scheme 4)

Step 1

A solution of macrocycle **1b** (26.9 mg, 0.05 mmol) and $Cu(MeCN)_4.PF_6$ (0.96 eq., 17.9 mg, 0.048 mmol) in THF (2.5 mL) was stirred at rt for 15 min, to form a solution of [**1b**.Cu].PF_6 (0.02 M). Separately, a solution of alkyne **1** (21.7 mg, 0.04 mmol) in THF (2.0 mL) was cooled to -78 °C and ^{n}BuLi (2.5 M in hexanes, 0.16 µL, 0.04 mmol) was added. After 15 min the solution of the lithiated alkyne was transferred to an ice bath, a portion of the solution of [**1b**.Cu].PF_6 (2.0 mL) was added and the resulting mixture allowed to warm to rt to give a solution of macrocycle bound Cu-acetylide (0.01M).

Step 2 (no extra [**1b**.Cu].PF_6)

A portion of the solution of macrocycle bound Cu-acetylide (0.50 mL, 0.005 mmol) was added to azide **3** (2.9 mg, 0.005 mmol) in THF (0.5 mL) and the mixture heated at 80 °C for 16 h. The solution was allowed to return to rt before dilution with further CH_2Cl_2 (20 mL) and washing with 16% aqueous EDTA tetrasodium-saturated ammonia solution (20 mL). The aqueous layer was extracted with CH_2Cl_2 (2×20 mL). The organic extracts were combined, dried over $MgSO_4$ and dried *in vacuo*. The residue was analysed by ^{1}H NMR. All macrocycle **1b** was consumed in the formation of rotaxane **4b**, the only interlocked product formed (ratio **4b** : **5b** = >99% : <1%)

Step 2 (additional [**1b**.Cu].PF_6)

A portion of the solution of [**1b**.Cu].PF_6 (0.25 mL, 0.005 mmol) was added to azide **3** (2.9 mg, 0.005 mmol) in THF (0.25 mL) followed by a portion of the solution of macrocycle bound Cu-acetylide (0.50 mL, 0.005 mmol) and the resulting mixture heated at 80 °C for 16 h. The solution was allowed to return to rt before dilution with further CH_2Cl_2 (20 mL) and washing with 16% aqueous EDTA tetrasodium-saturated ammonia solution (20 mL). The aqueous layer was extracted with CH_2Cl_2 (2 × 20 mL). The organic extracts were combined, dried over $MgSO_4$ and dried *in vacuo*. The residue was analysed by ^{1}H NMR. Rotaxanes **4b** and **5b** formed in a 90 : 10 ratio.

[3]Rotaxane 5a ^{1}H NMR ($CDCl_3$, 400 MHz, 300 K)

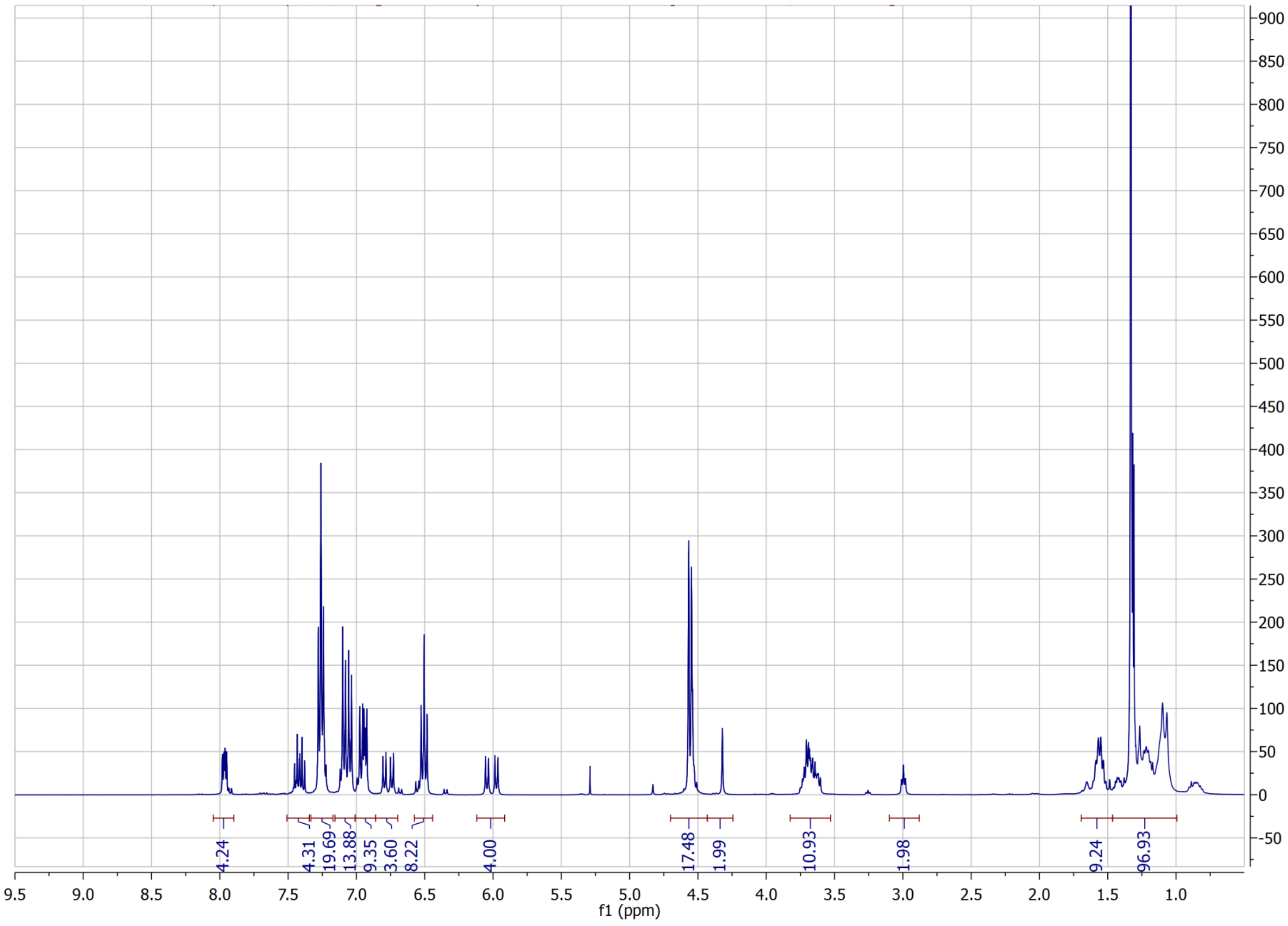

[3]Rotaxane 5a DEPTQ135 NMR ($CDCl_3$, 100 MHz, 300 K)

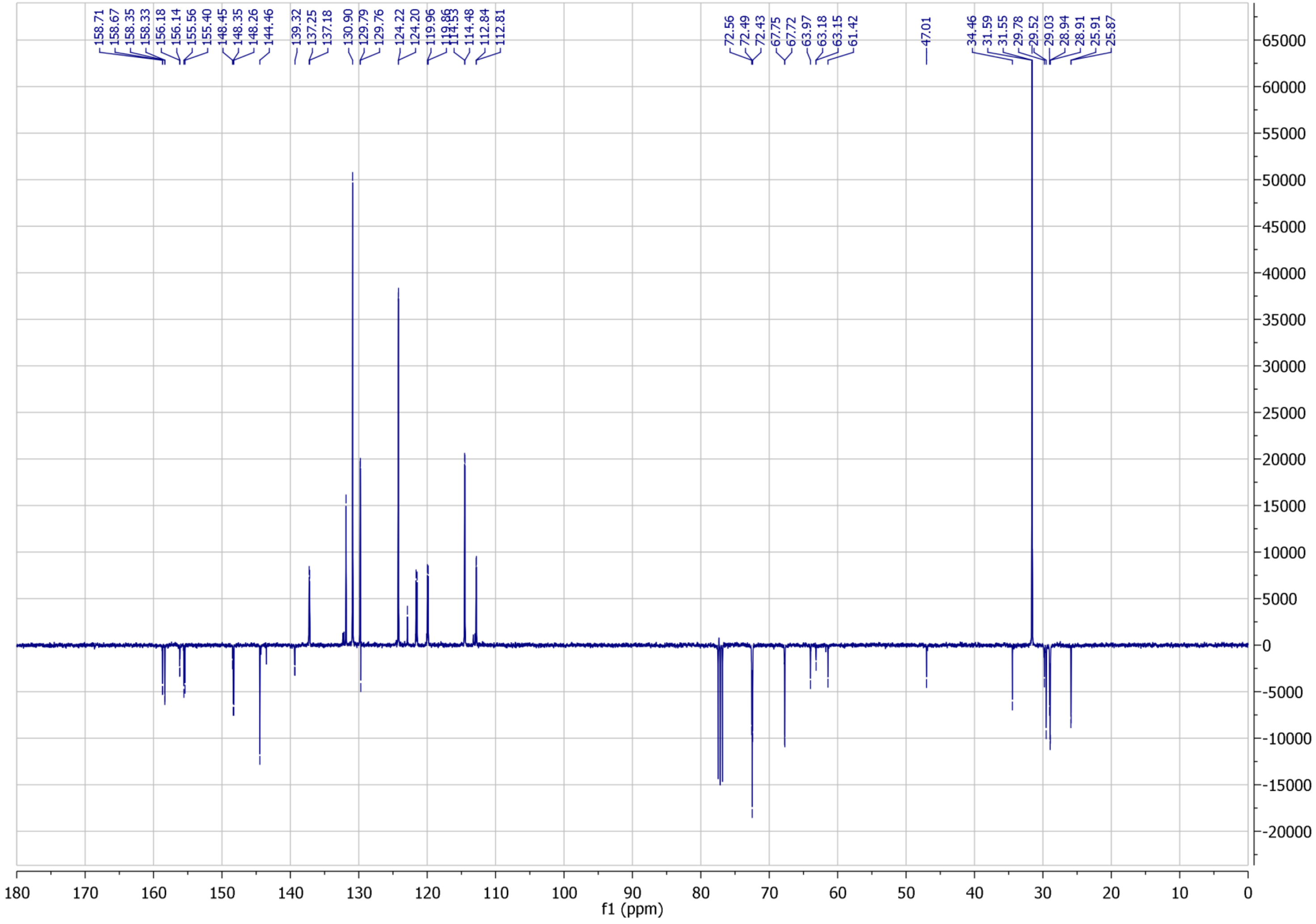

[3]Rotaxane 5a COSY ($CDCl_3$, 400 MHz, 300 K)

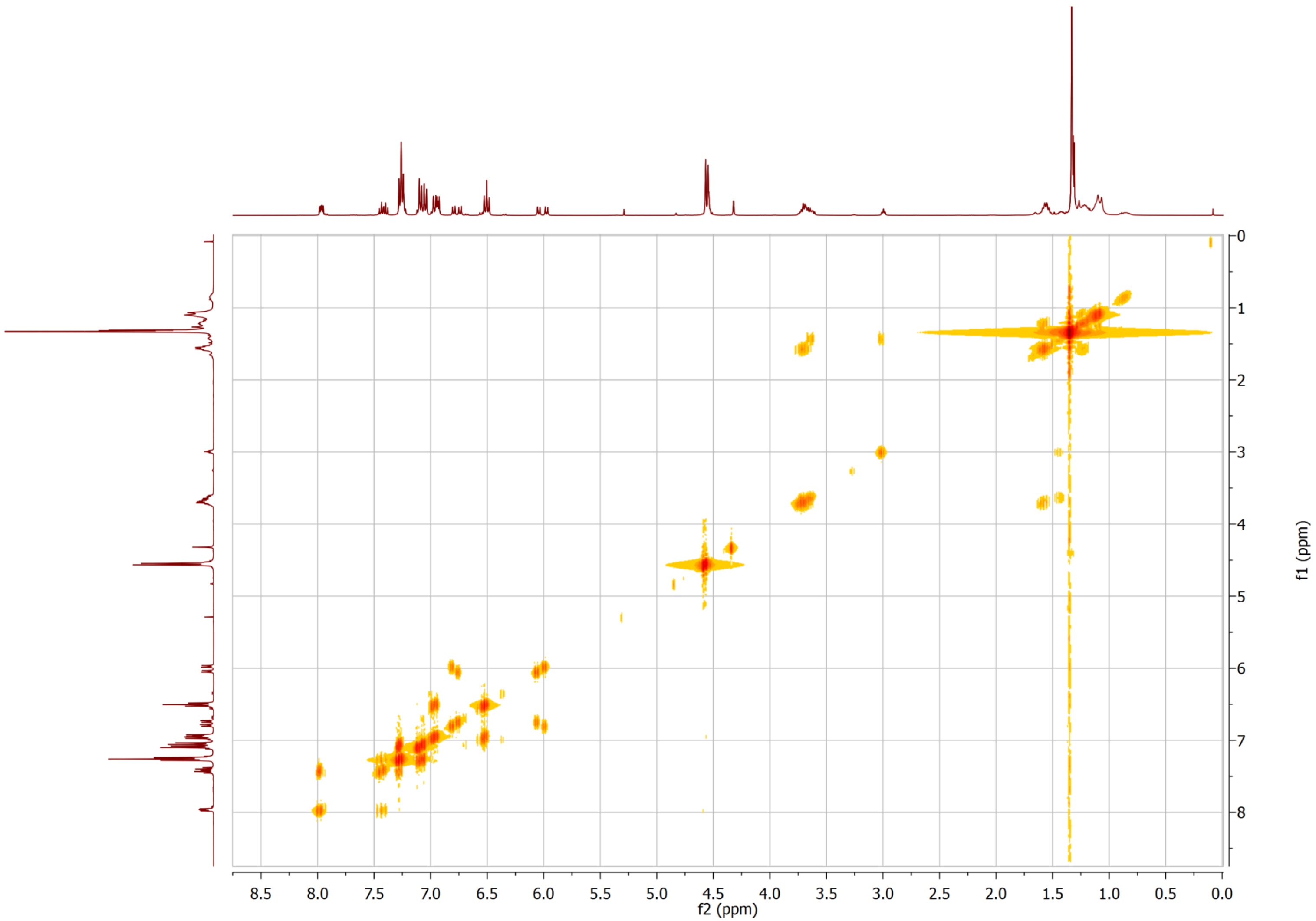

[3]Rotaxane 5a HSQC NMR ($CDCl_3$, 400 MHz, 300 K)

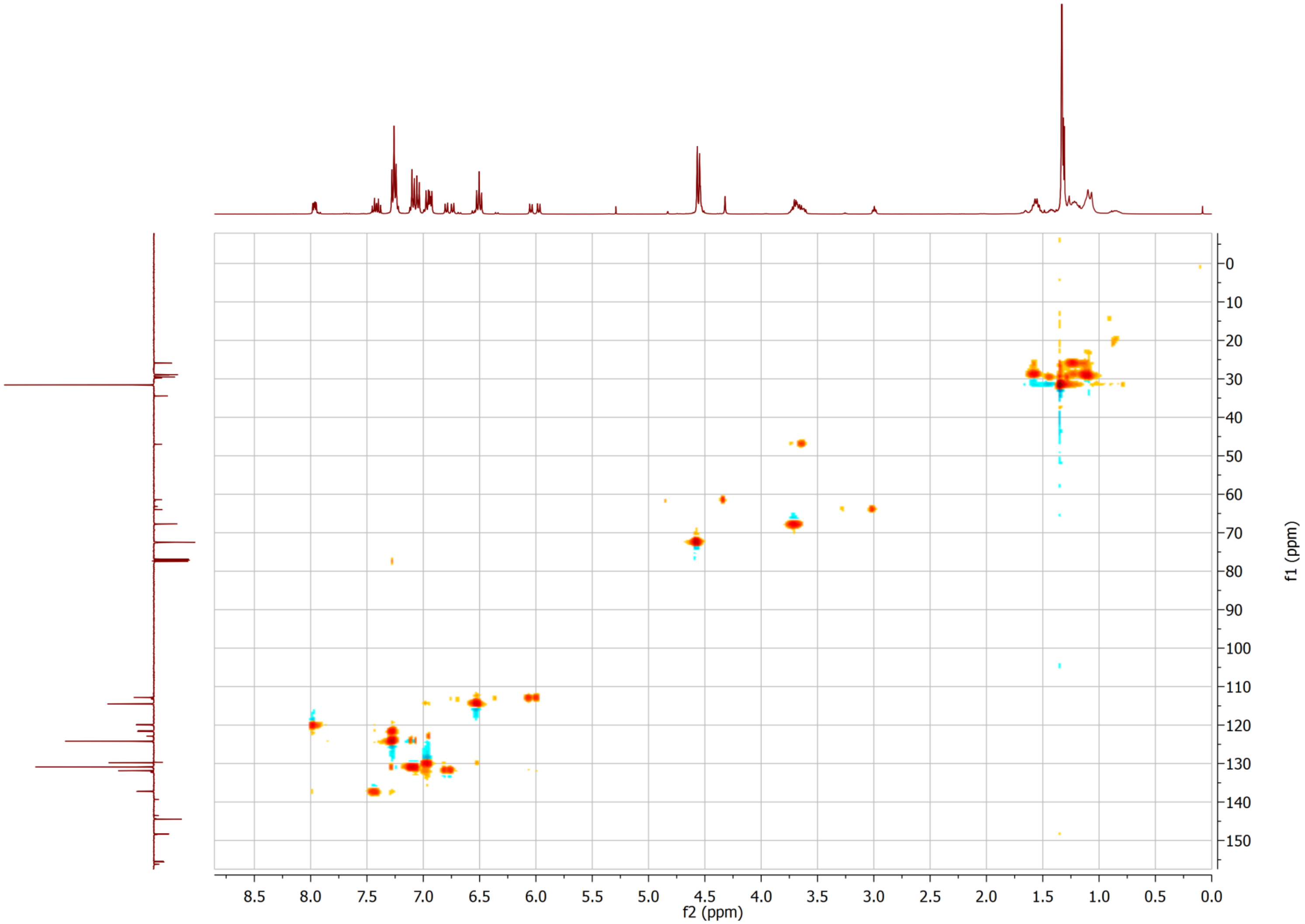

[3]Rotaxane 5a HMBC NMR ($CDCl_3$, 400 MHz, 300 K)

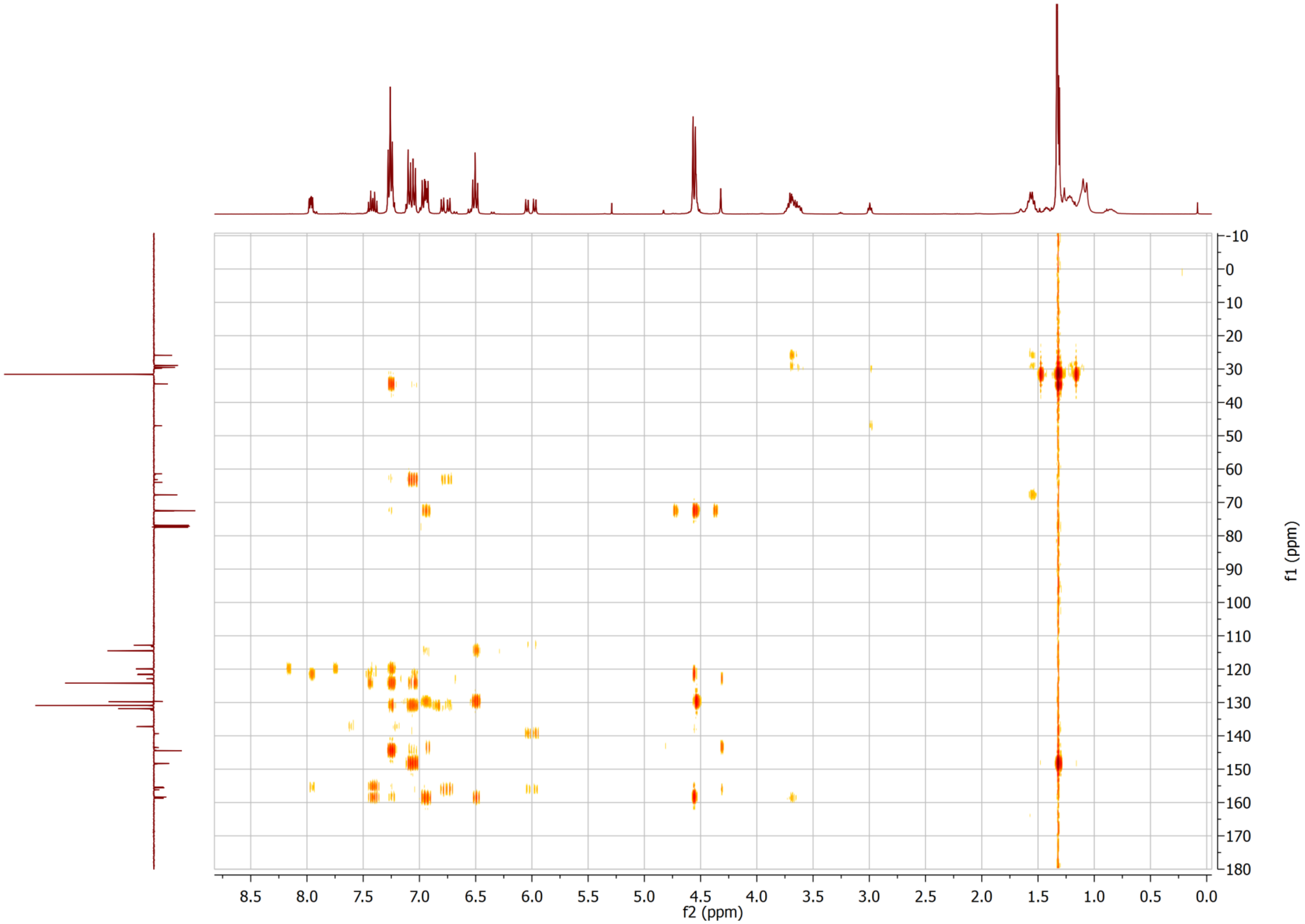

[3]Rotaxane 5b ^{1}H NMR ($CDCl_3$, 400 MHz, 300 K)

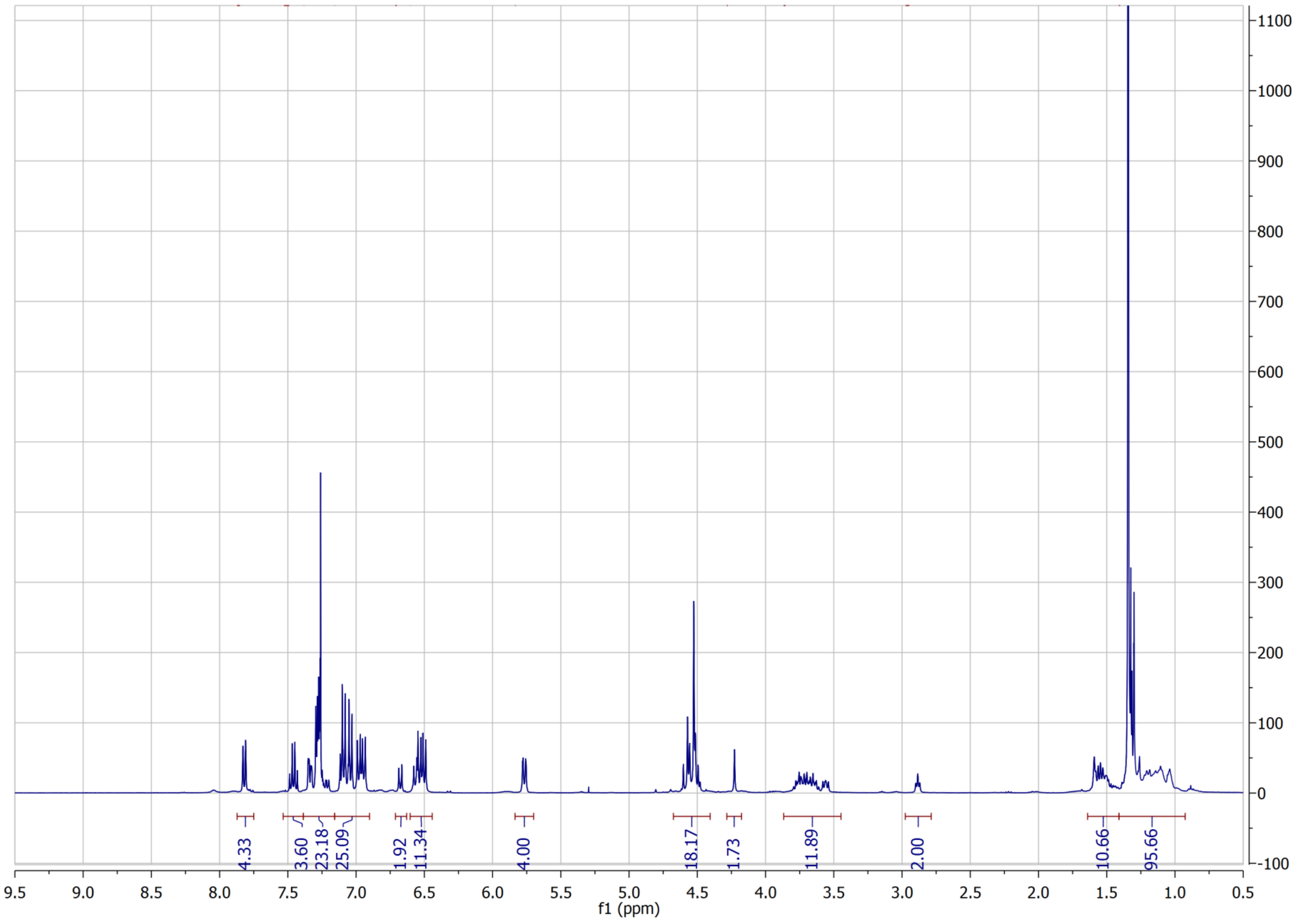

[3]Rotaxane 5b DEPTQ135 NMR ($CDCl_3$, 100 MHz, 300 K)

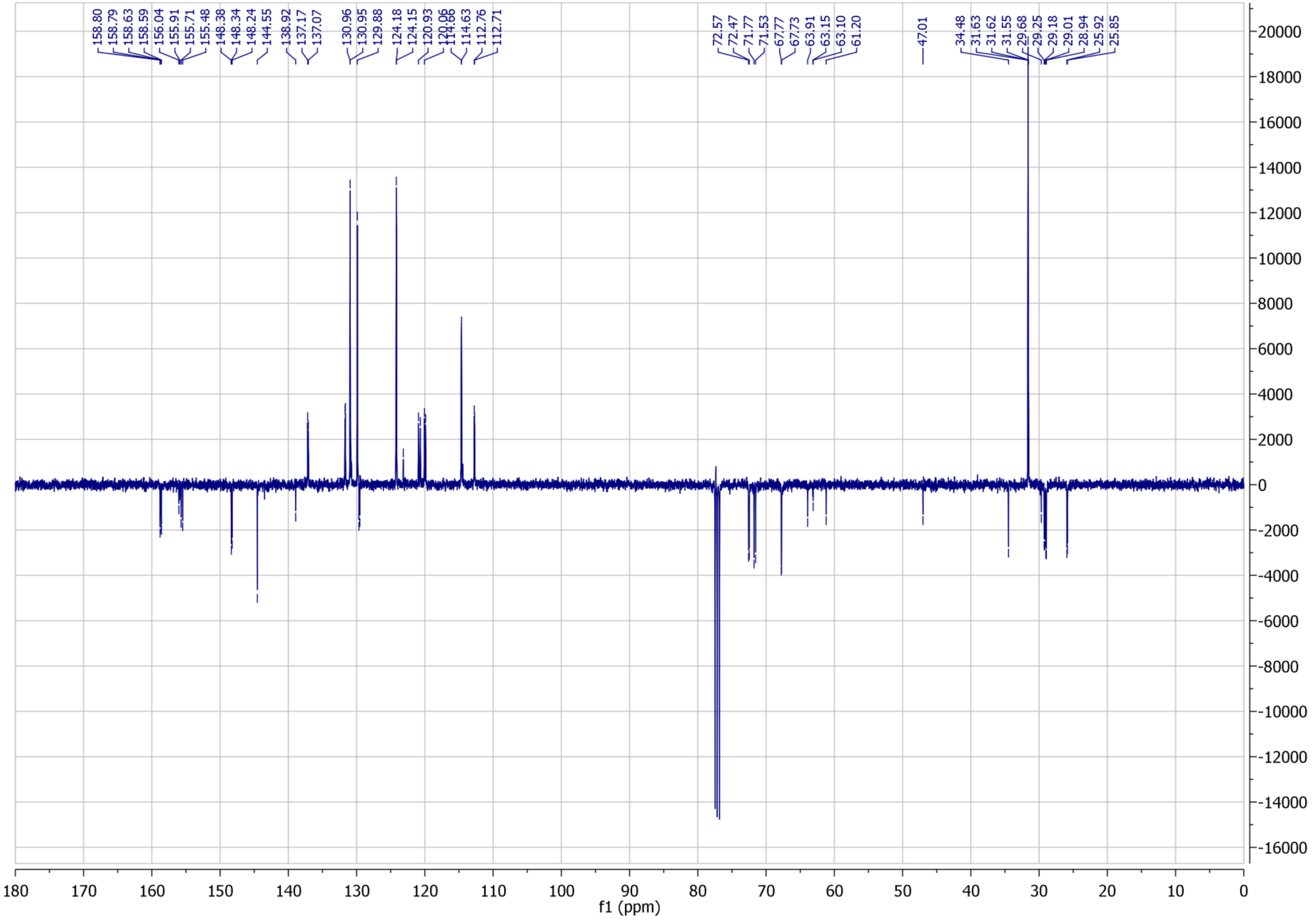

[3]Rotaxane 5b COSY ($CDCl_3$, 400 MHz, 300 K)

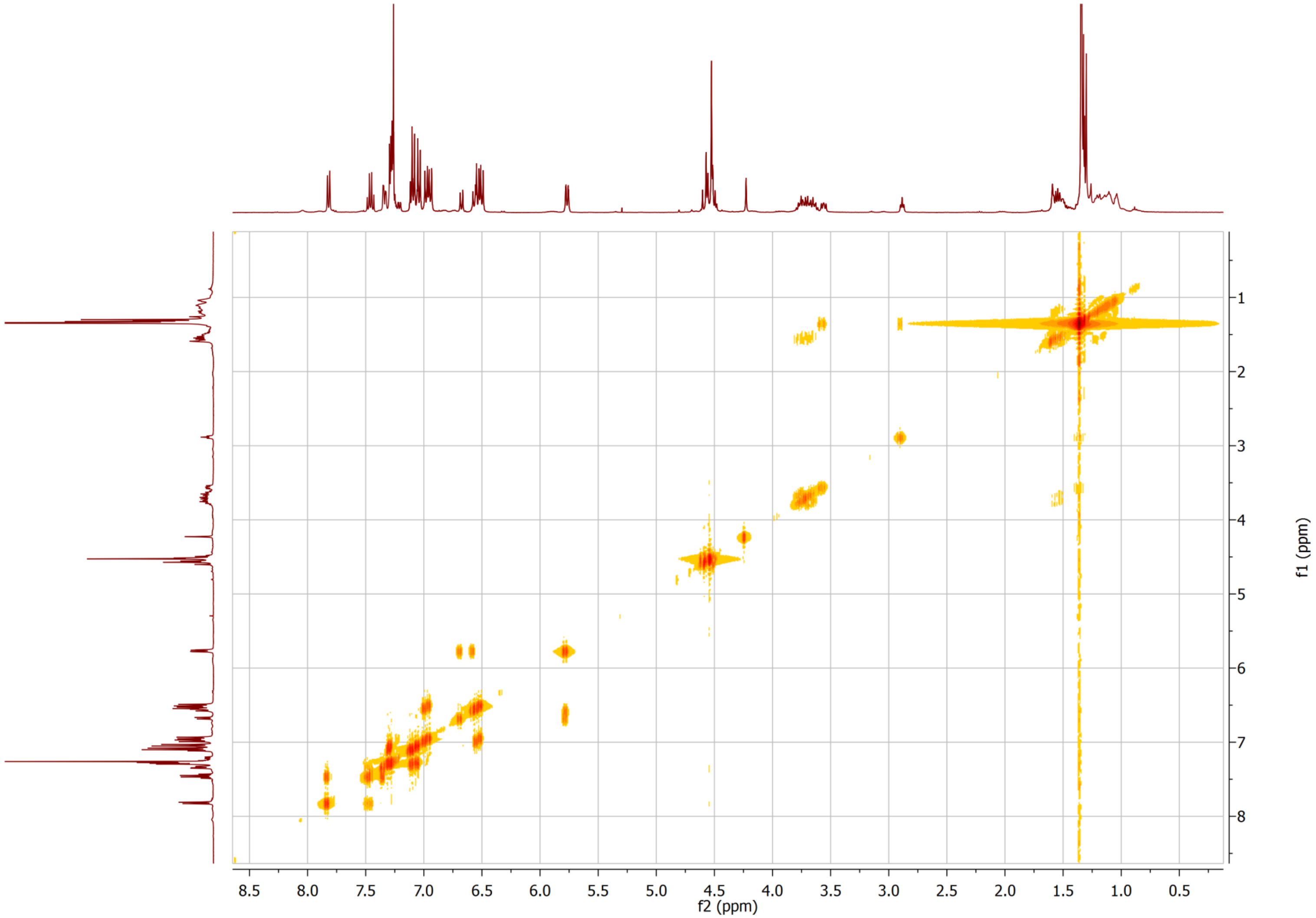

[3]Rotaxane 5b HSQC NMR ($CDCl_3$, 400 MHz, 300 K)

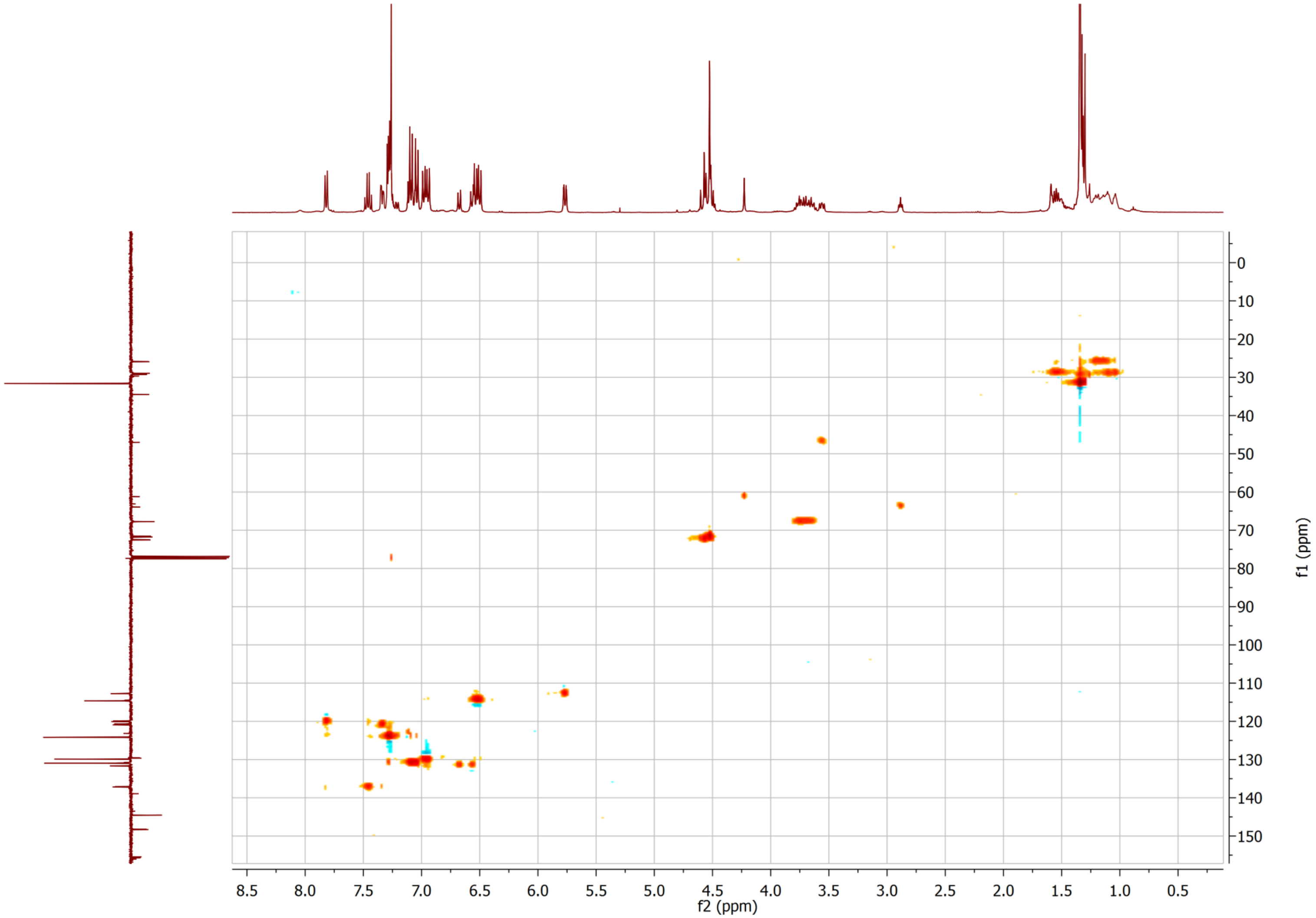

[3]Rotaxane 5b HMBC NMR ($CDCl_3$, 400 MHz, 300 K)

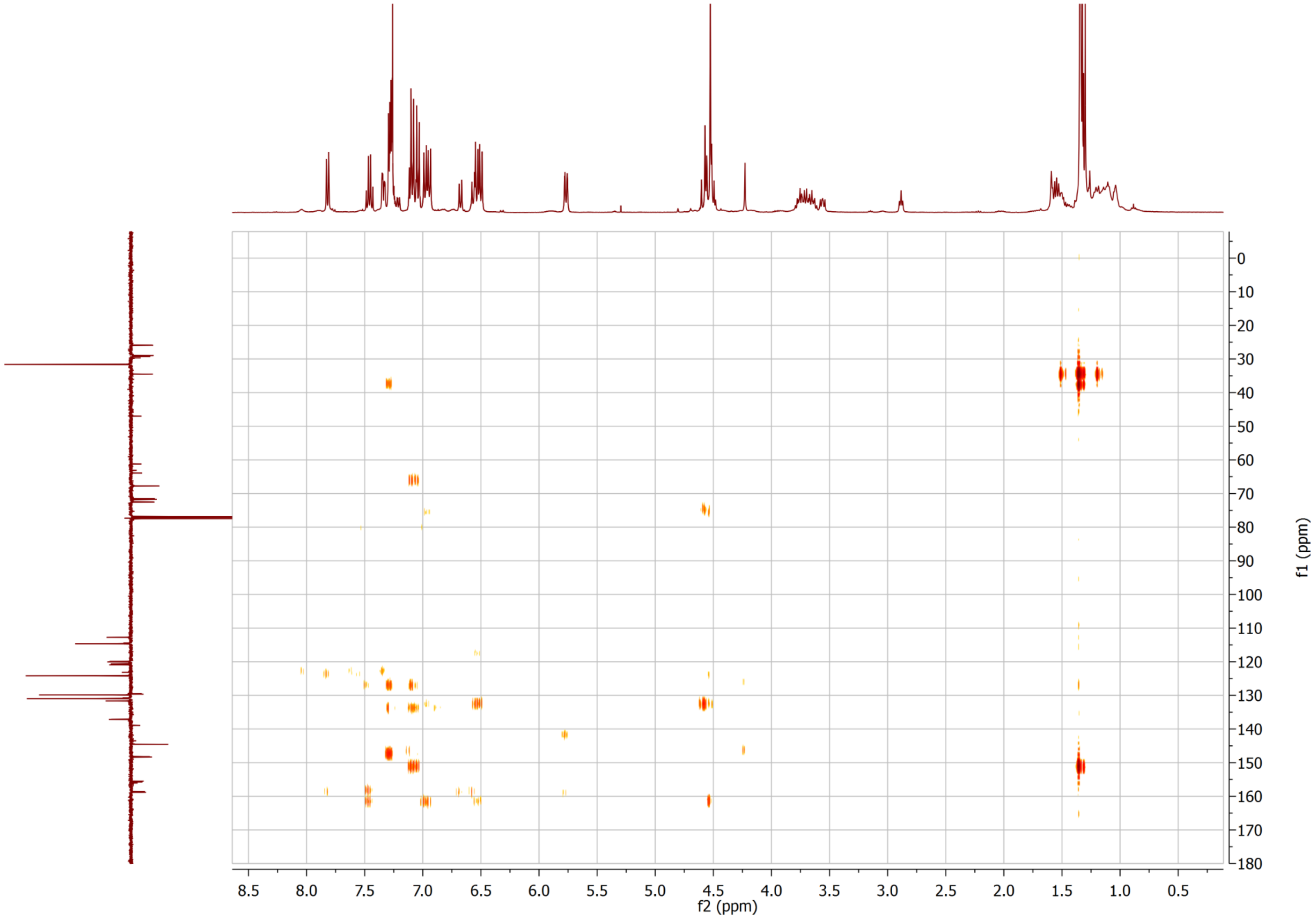

[3]Rotaxane 5c ^{1}H NMR ($CDCl_3$, 400 MHz, 300 K)

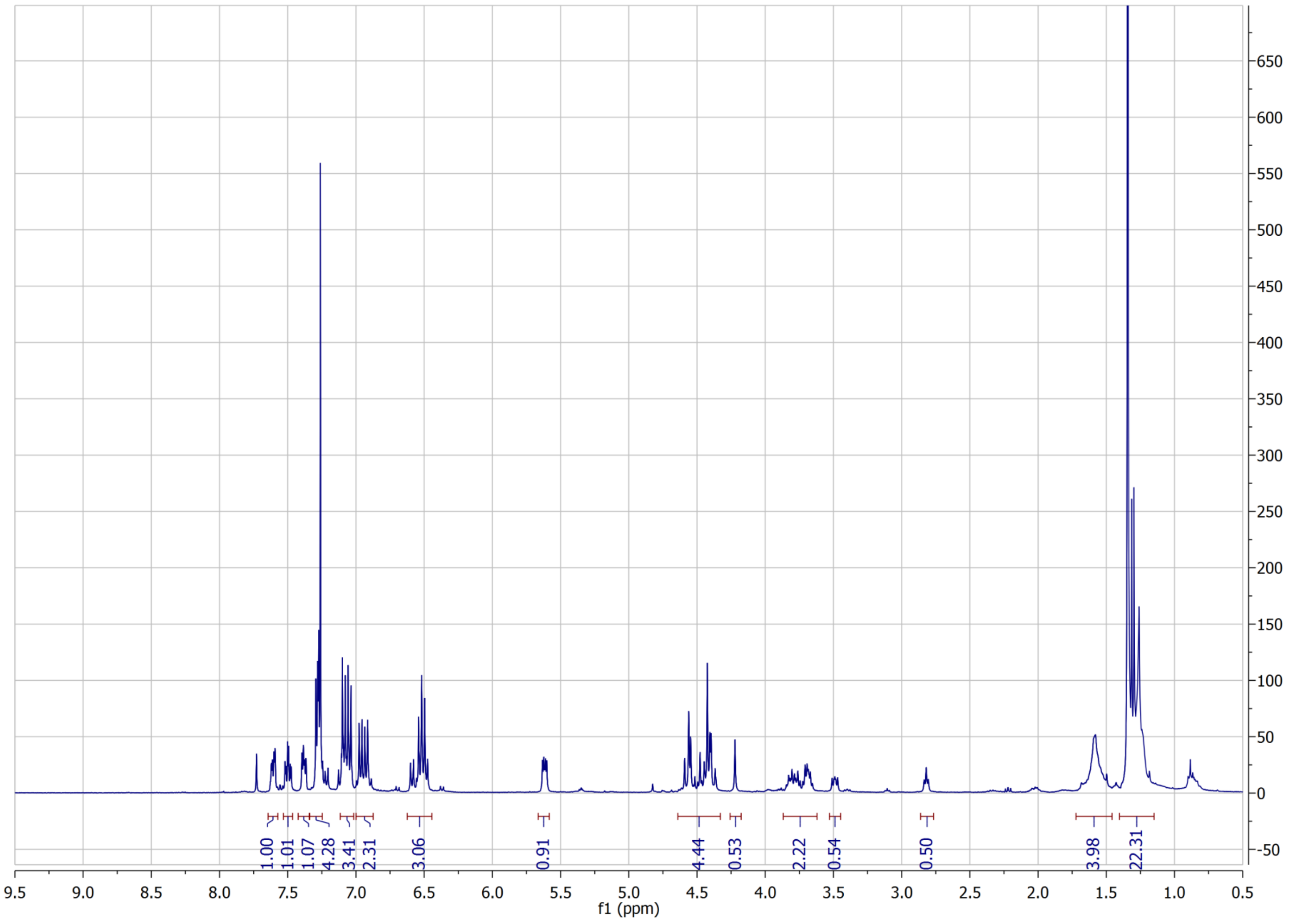

[3]Rotaxane 5c DEPTQ135 NMR ($CDCl_3$, 100 MHz, 300 K)

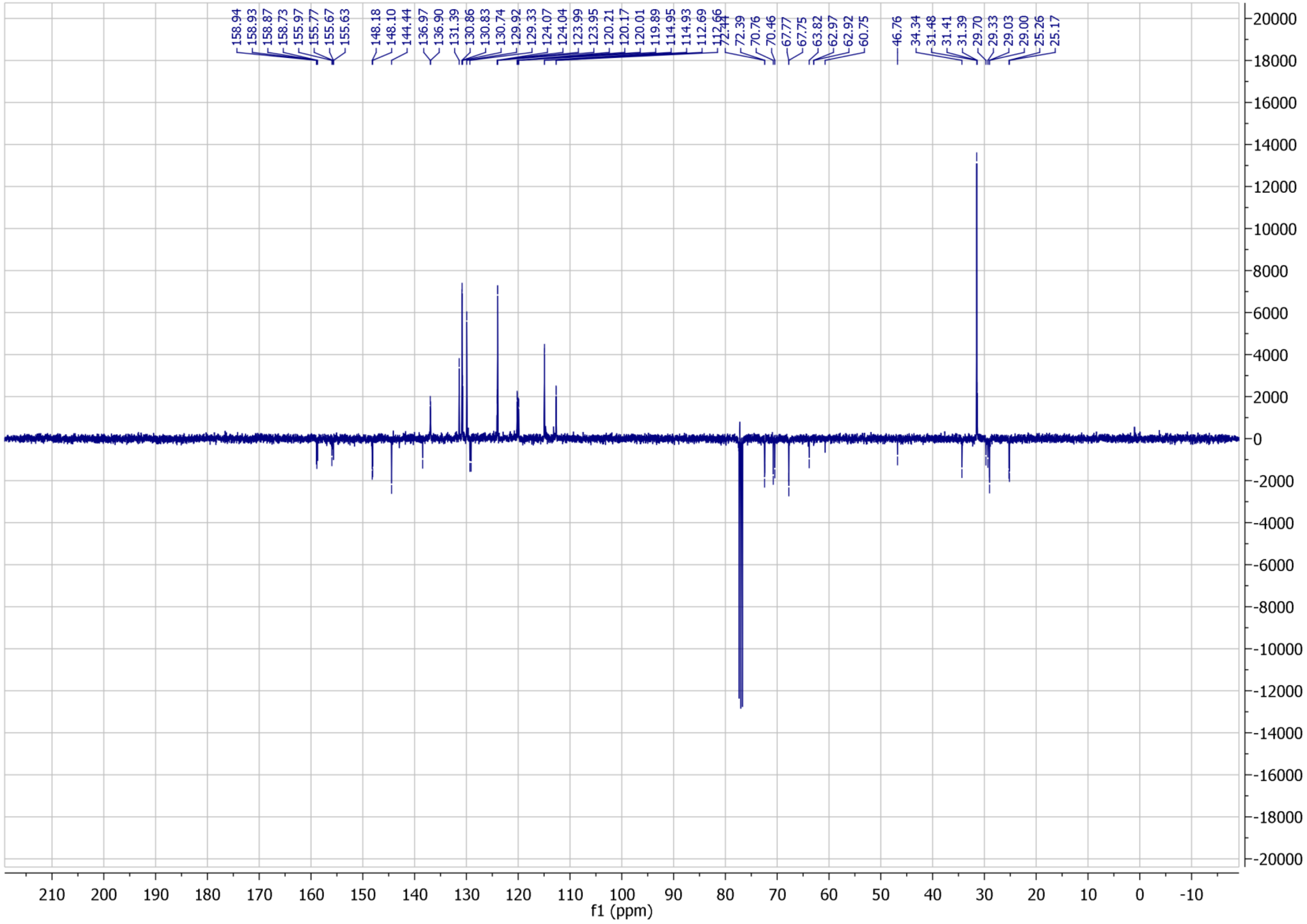

[3]Rotaxane 5c COSY ($CDCl_3$, 400 MHz, 300 K)

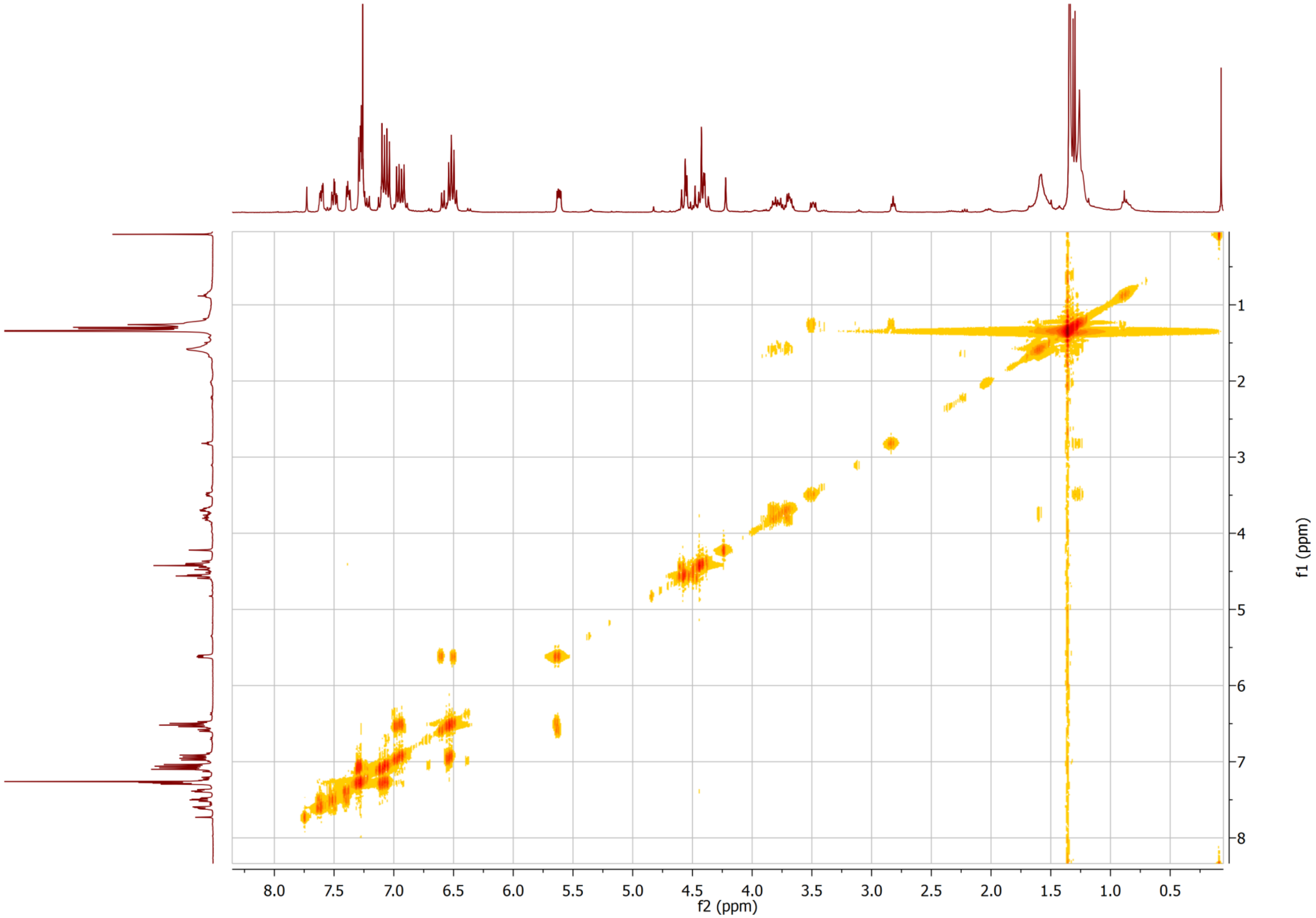

[3]Rotaxane 5c HSQC NMR ($CDCl_3$, 400 MHz, 300 K)

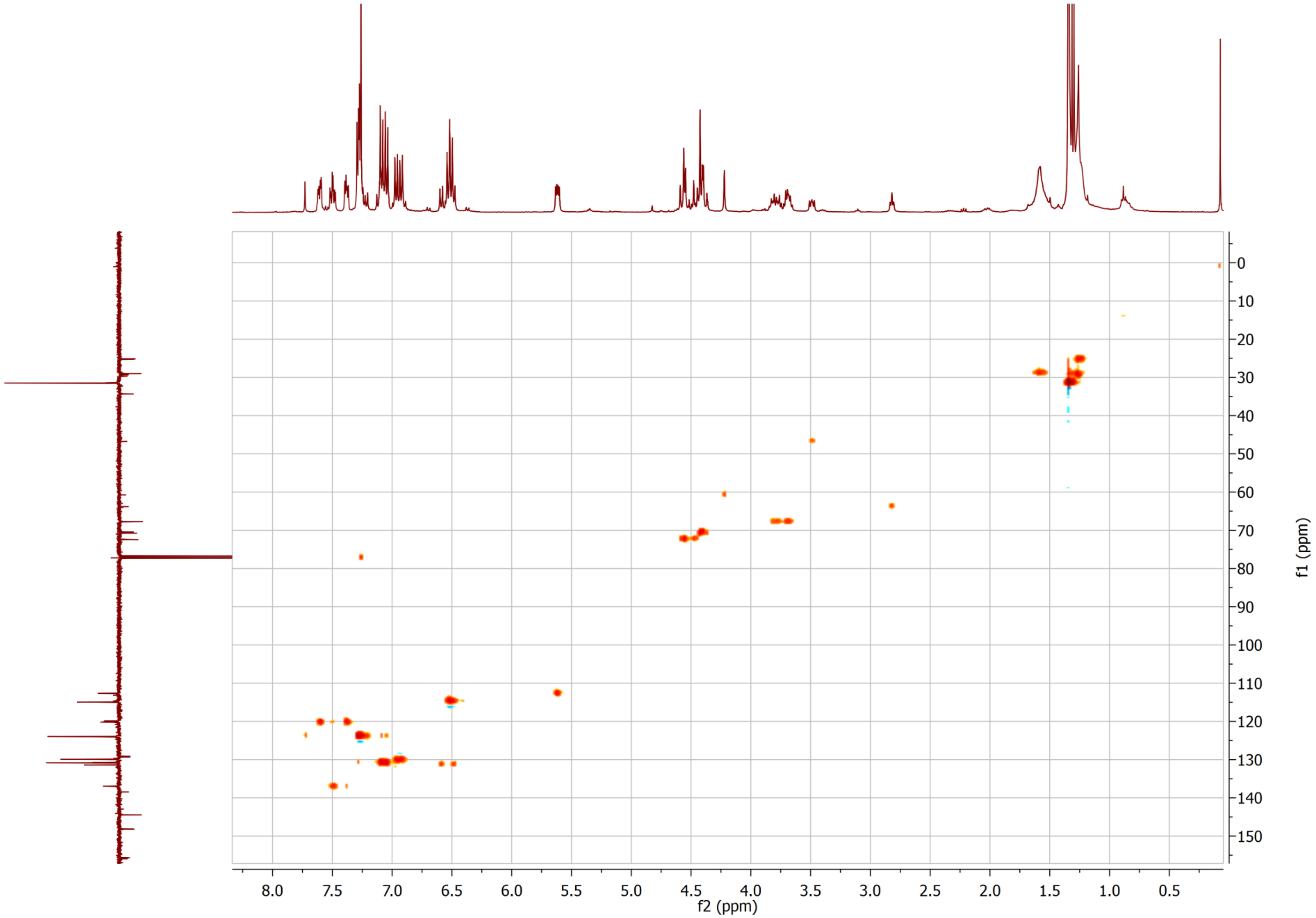

[3]Rotaxane 5c HMBC NMR ($CDCl_3$, 400 MHz, 300 K)

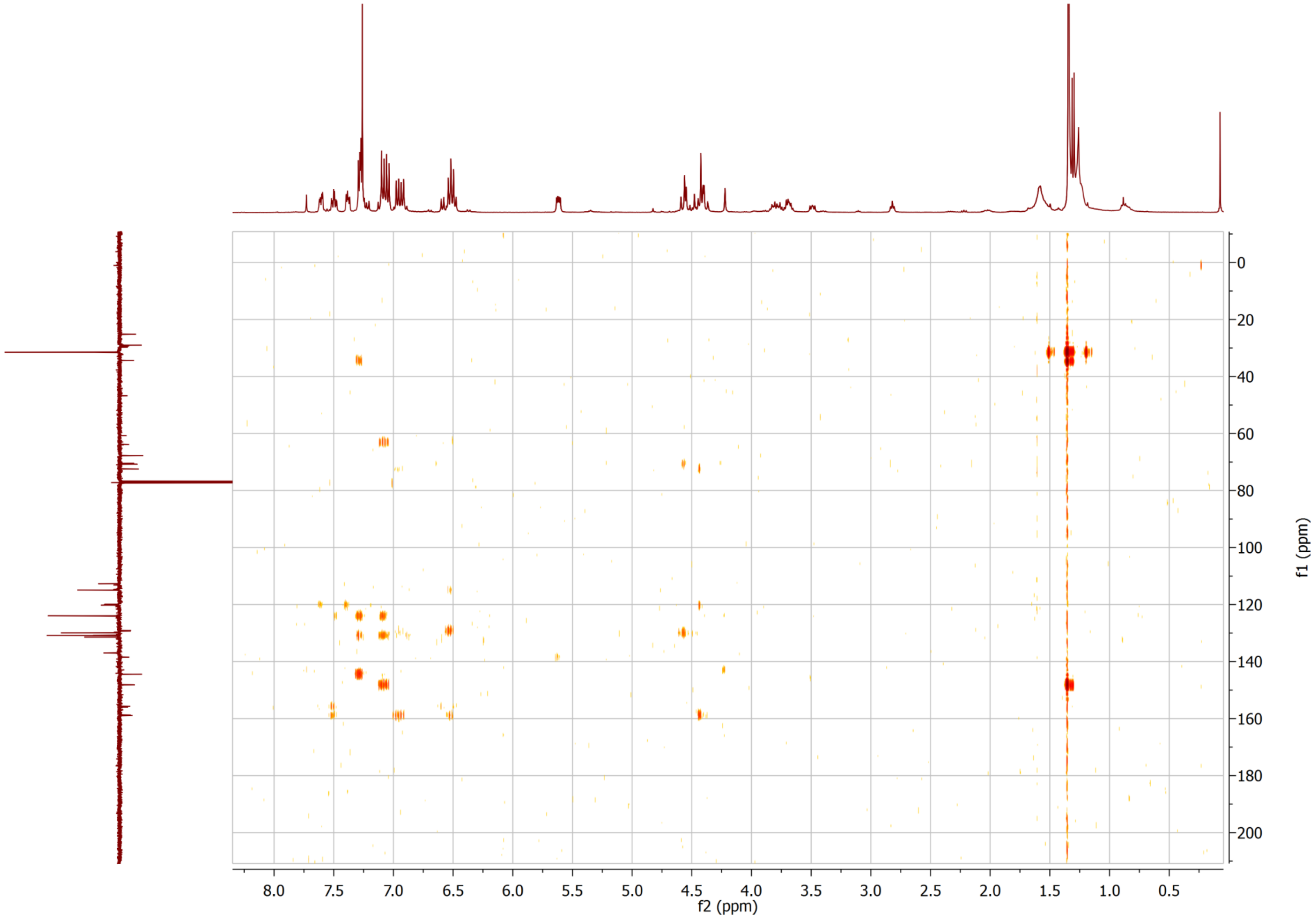

Azide 6 ^{1}H NMR ($CDCl_3$, 600 MHz, 300 K)

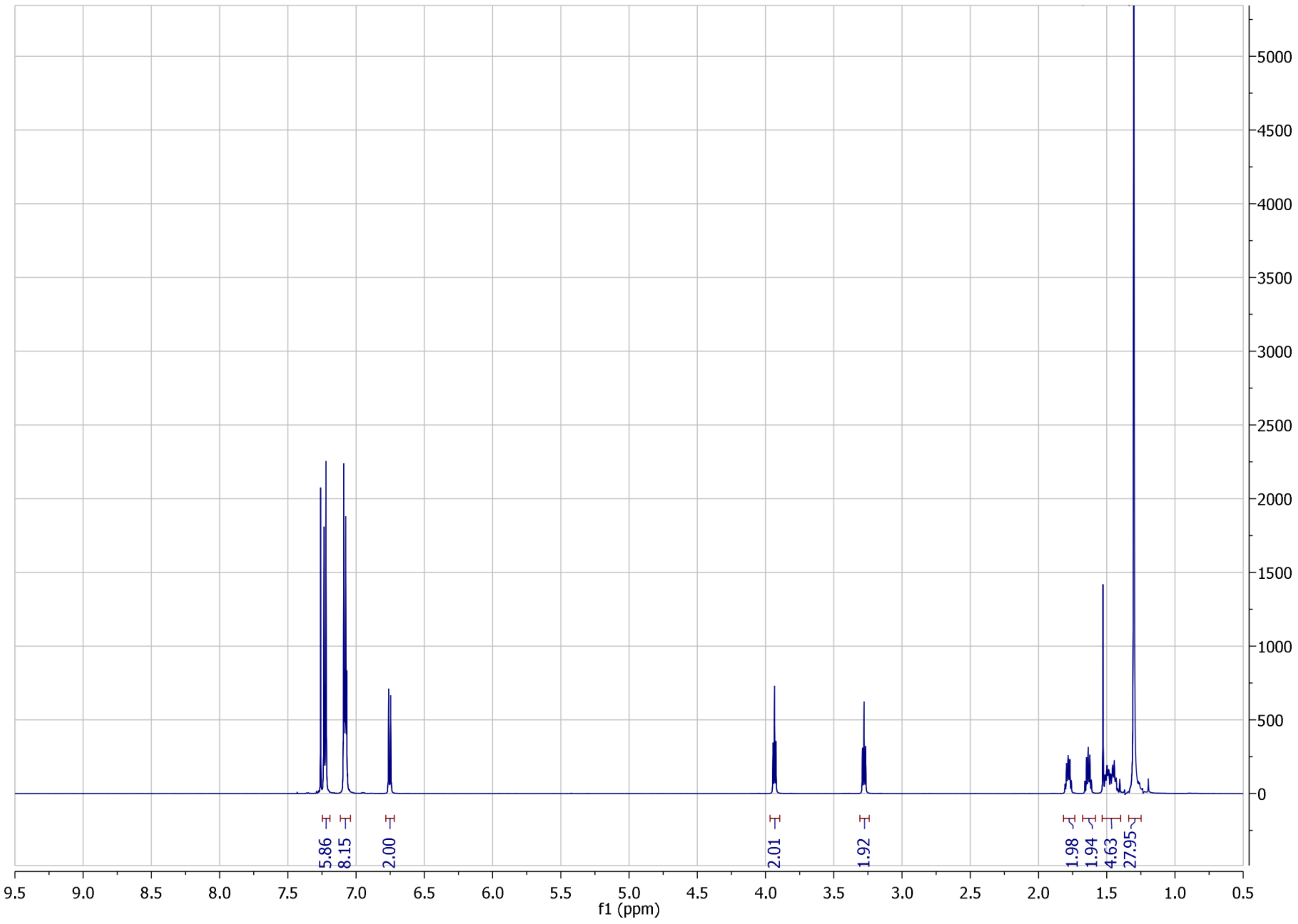

Azide 6 DEPTQ135 NMR ($CDCl_3$, 150 MHz, 300 K)

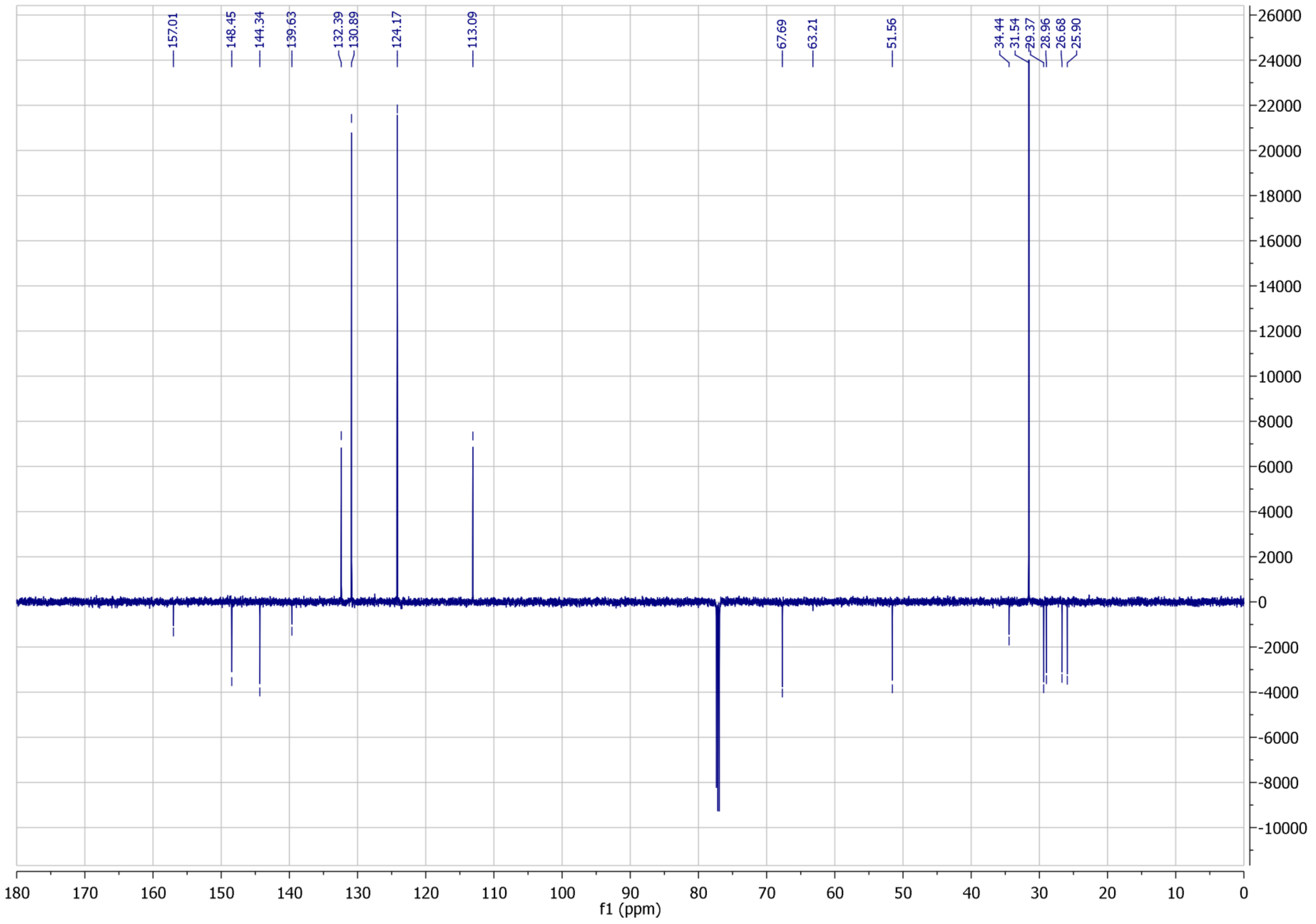

Alkyne 7 ^{1}H NMR ($CDCl_3$, 600 MHz, 300 K)

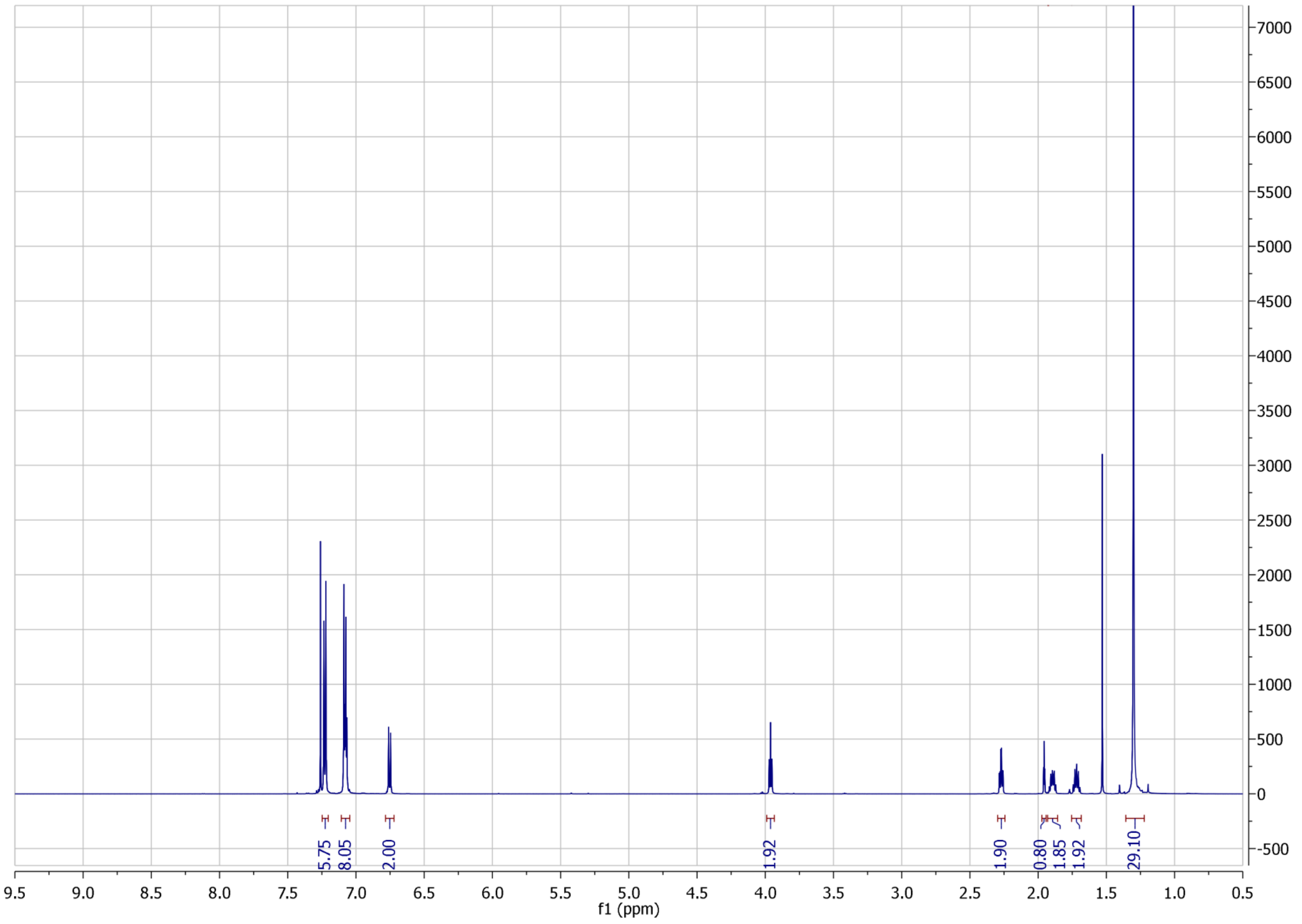

Alkyne 7 DEPTQ135 NMR ($CDCl_3$, 150 MHz, 300 K)

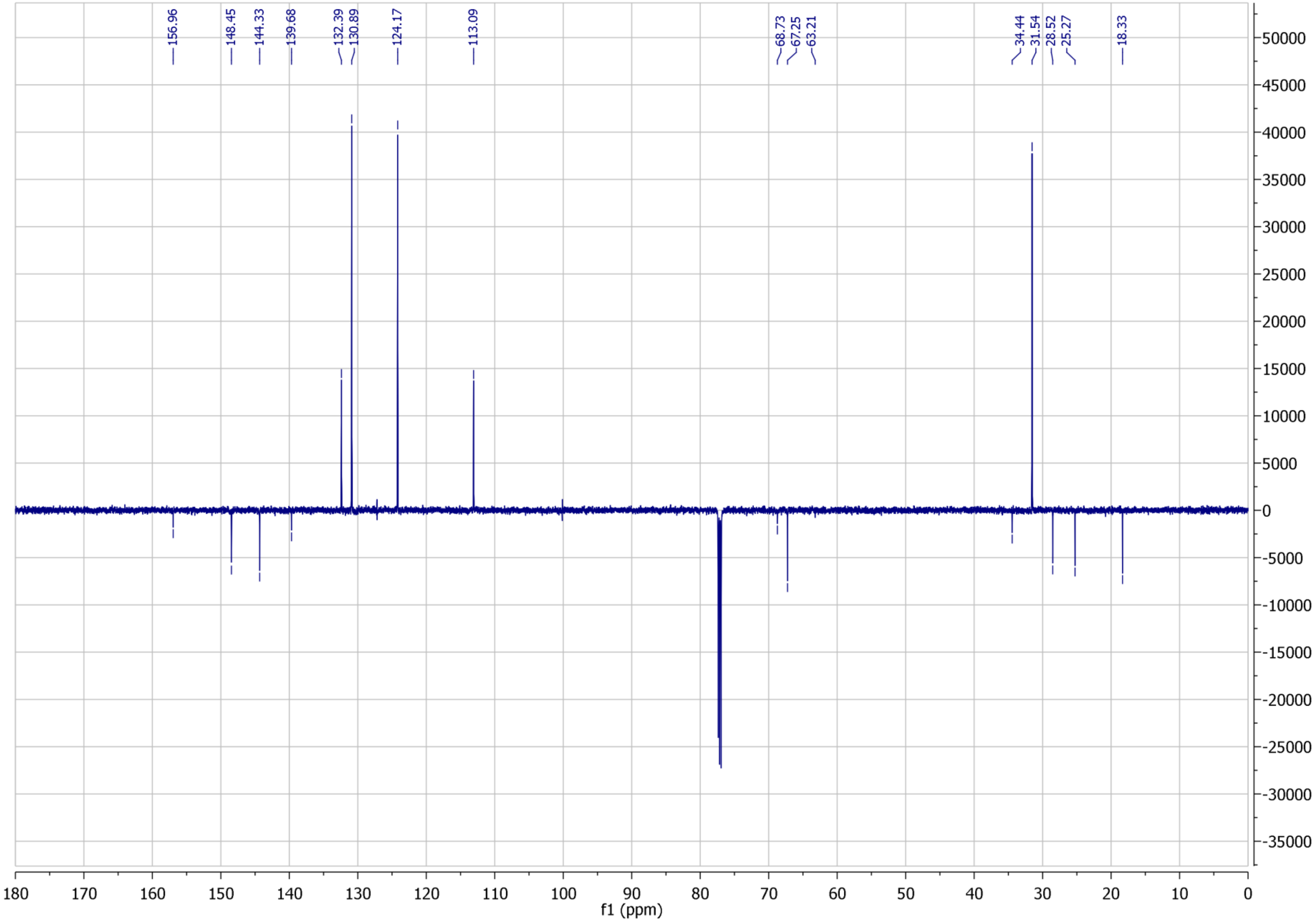

Alkyne 8 ^{1}H NMR ($CDCl_3$, 400 MHz, 300 K)

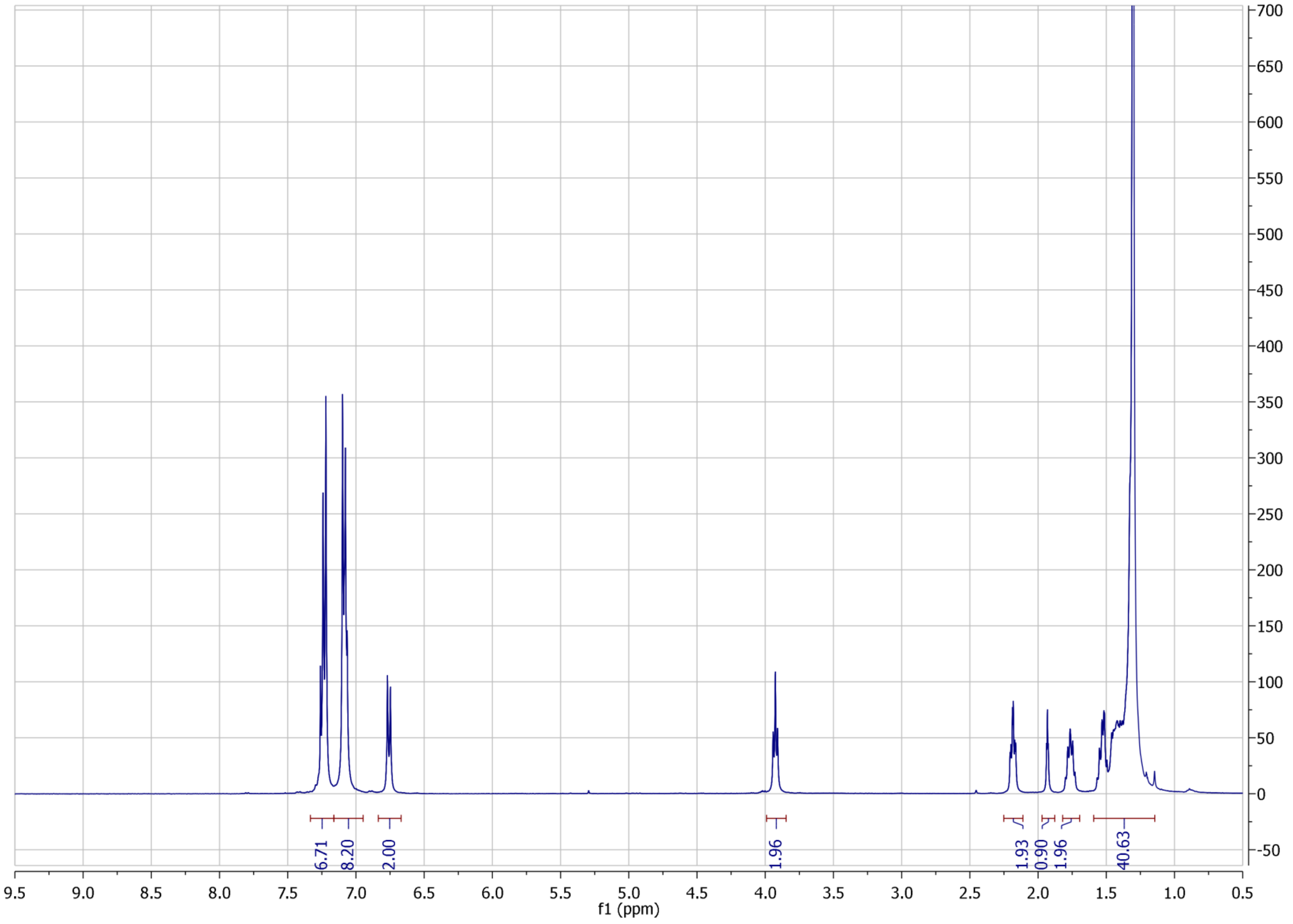

Alkyne 8 DEPTQ135 NMR ($CDCl_3$, 100 MHz, 300 K)

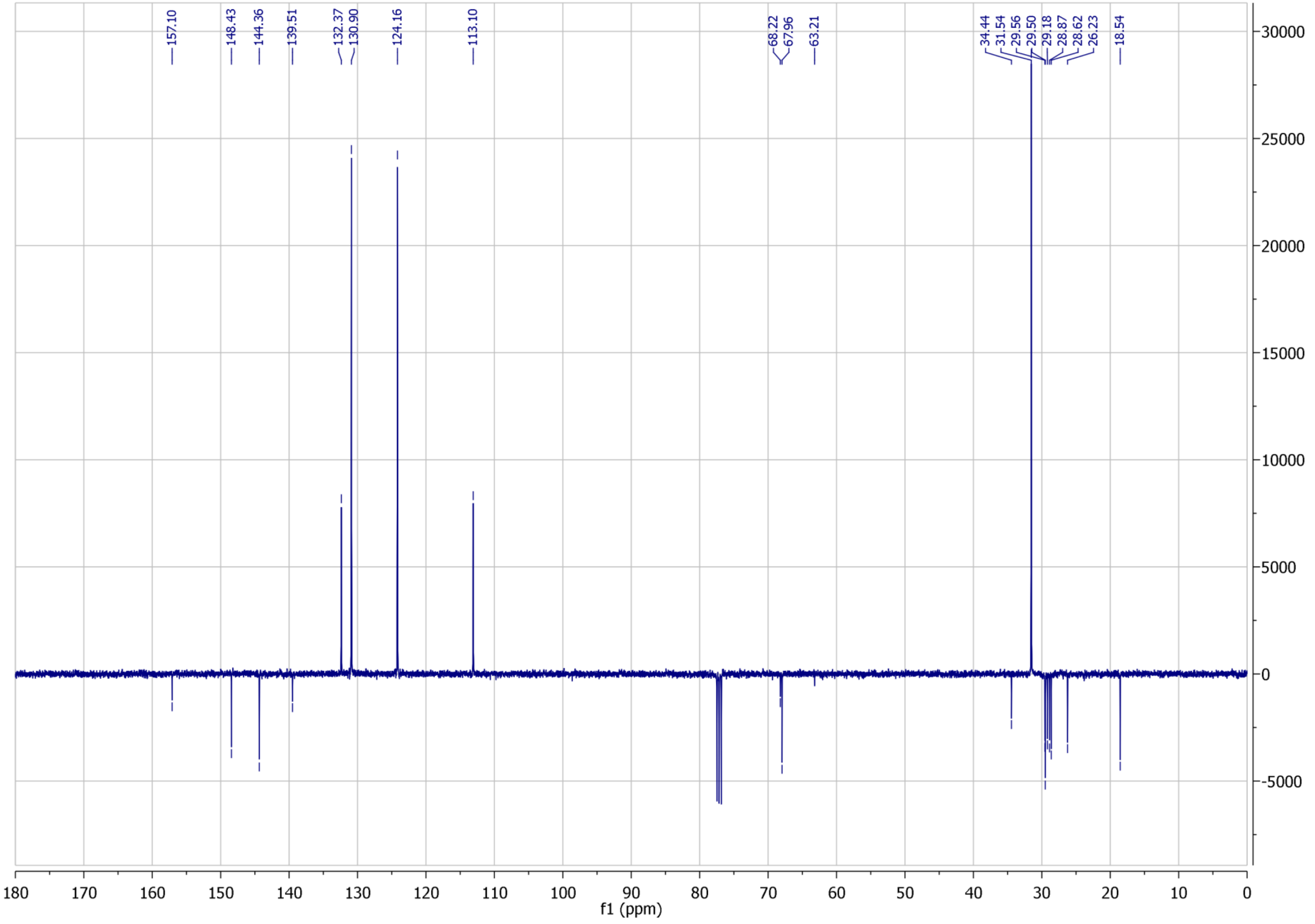

[3]Rotaxane S6 ^{1}H NMR ($CDCl_3$, 400 MHz, 300 K)

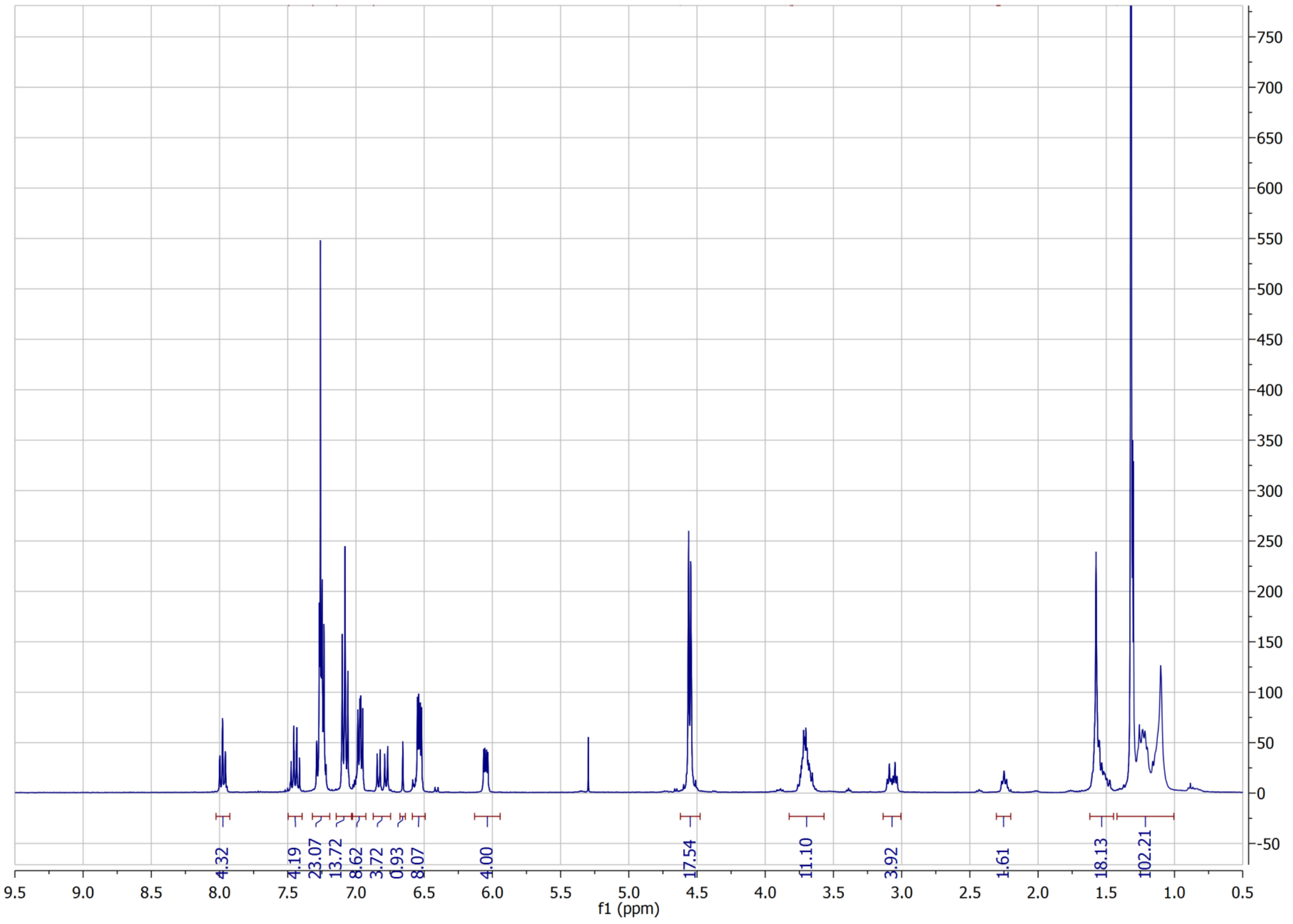

[3]Rotaxane S6 DEPTQ135 NMR ($CDCl_3$, 150 MHz, 300 K)

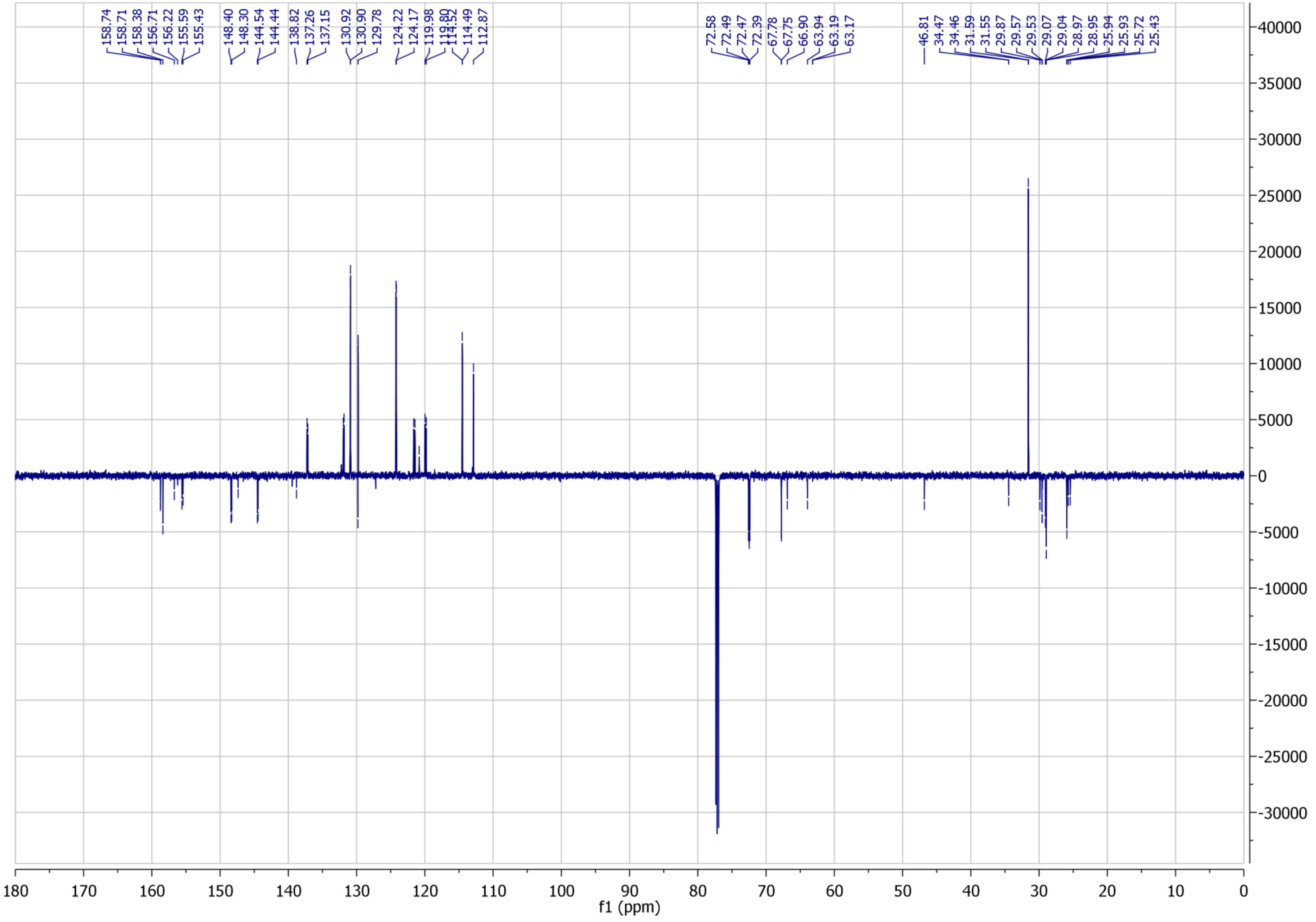

[3]Rotaxane S6 COSY ($CDCl_3$, 400 MHz, 300 K)

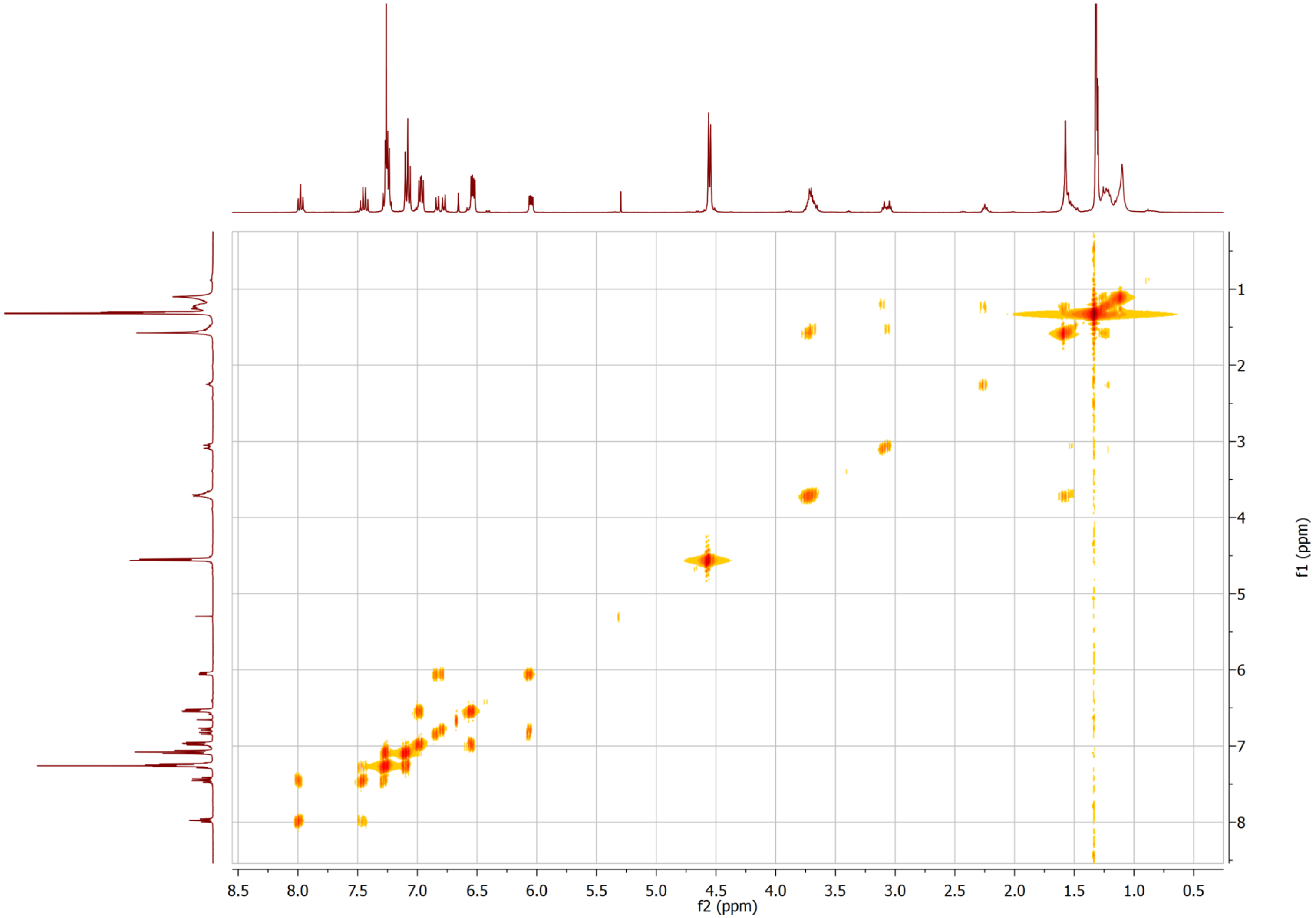

[3]Rotaxane S6 HSQC NMR ($CDCl_3$, 400 MHz, 300 K)

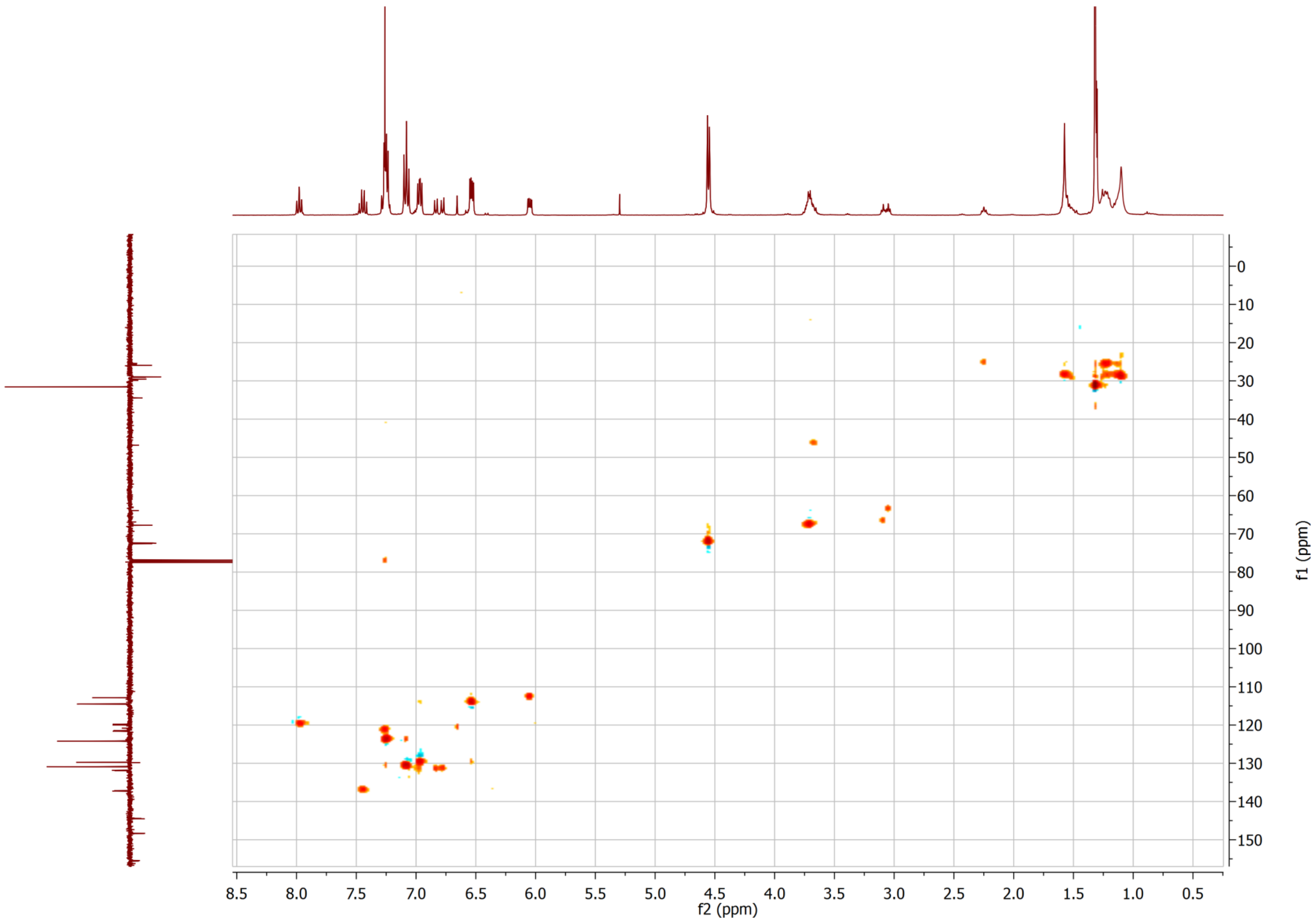

[3]Rotaxane S6 HMBC NMR ($CDCl_3$, 400 MHz, 300 K)

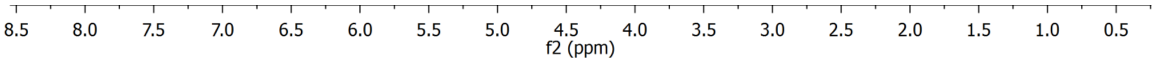

[2]Rotaxane S7 ^{1}H NMR ($CDCl_3$, 400 MHz, 300 K)

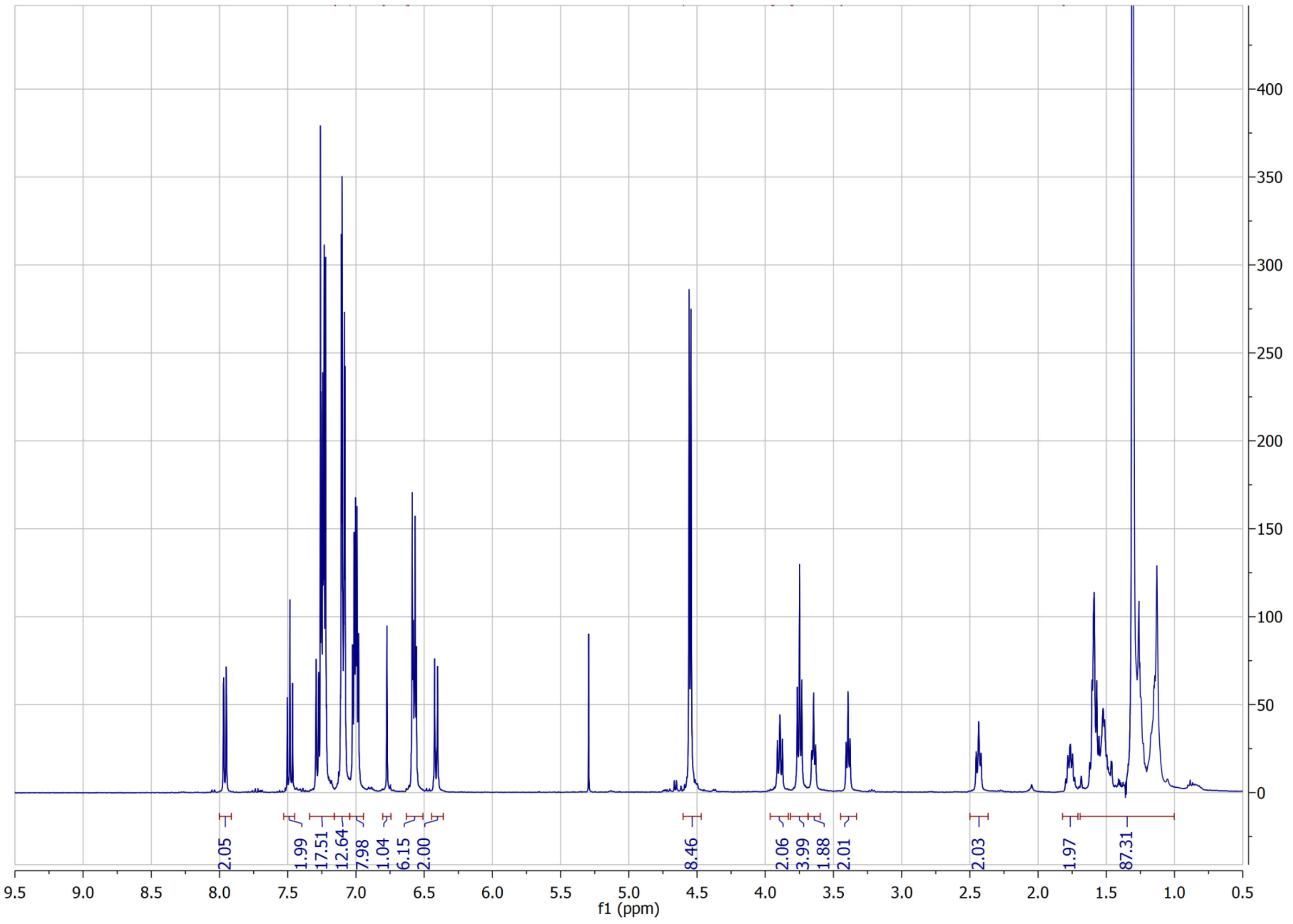

[2]Rotaxane S7 DEPTQ135 NMR ($CDCl_3$, 100 MHz, 300 K)

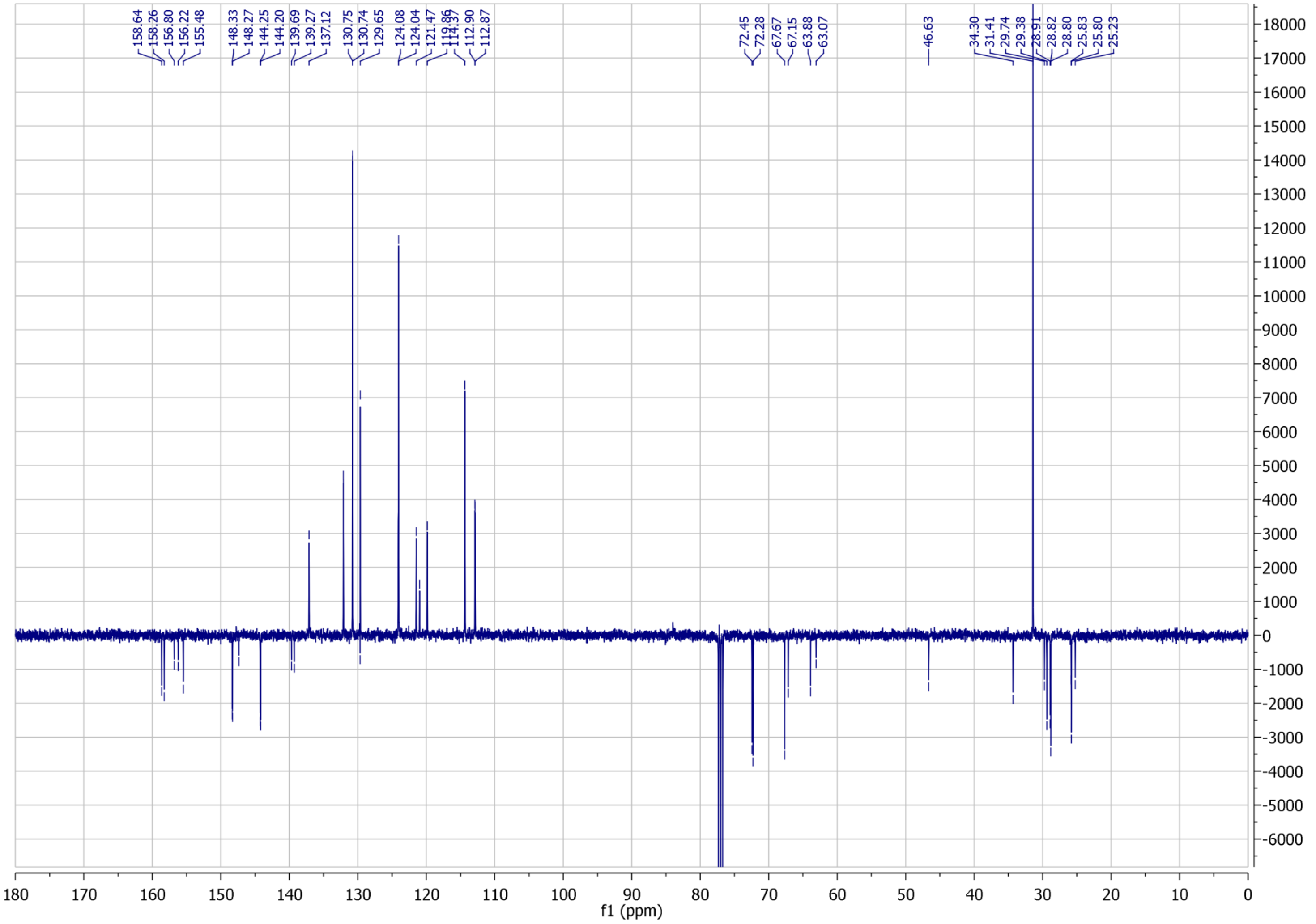

[2]Rotaxane S7 COSY ($CDCl_3$, 400 MHz, 300 K)

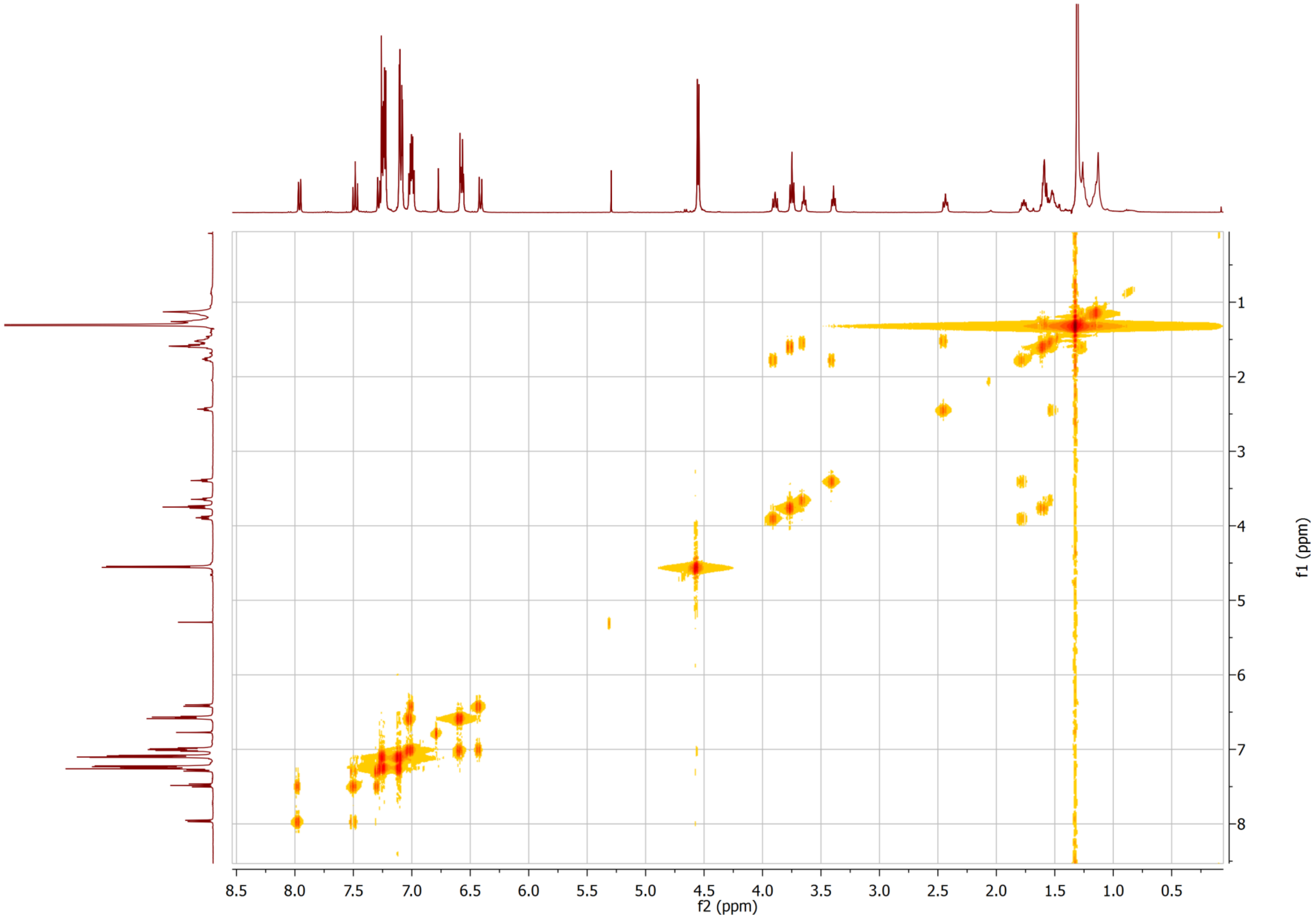

[2]Rotaxane S7 HSQC NMR ($CDCl_3$, 400 MHz, 300 K)

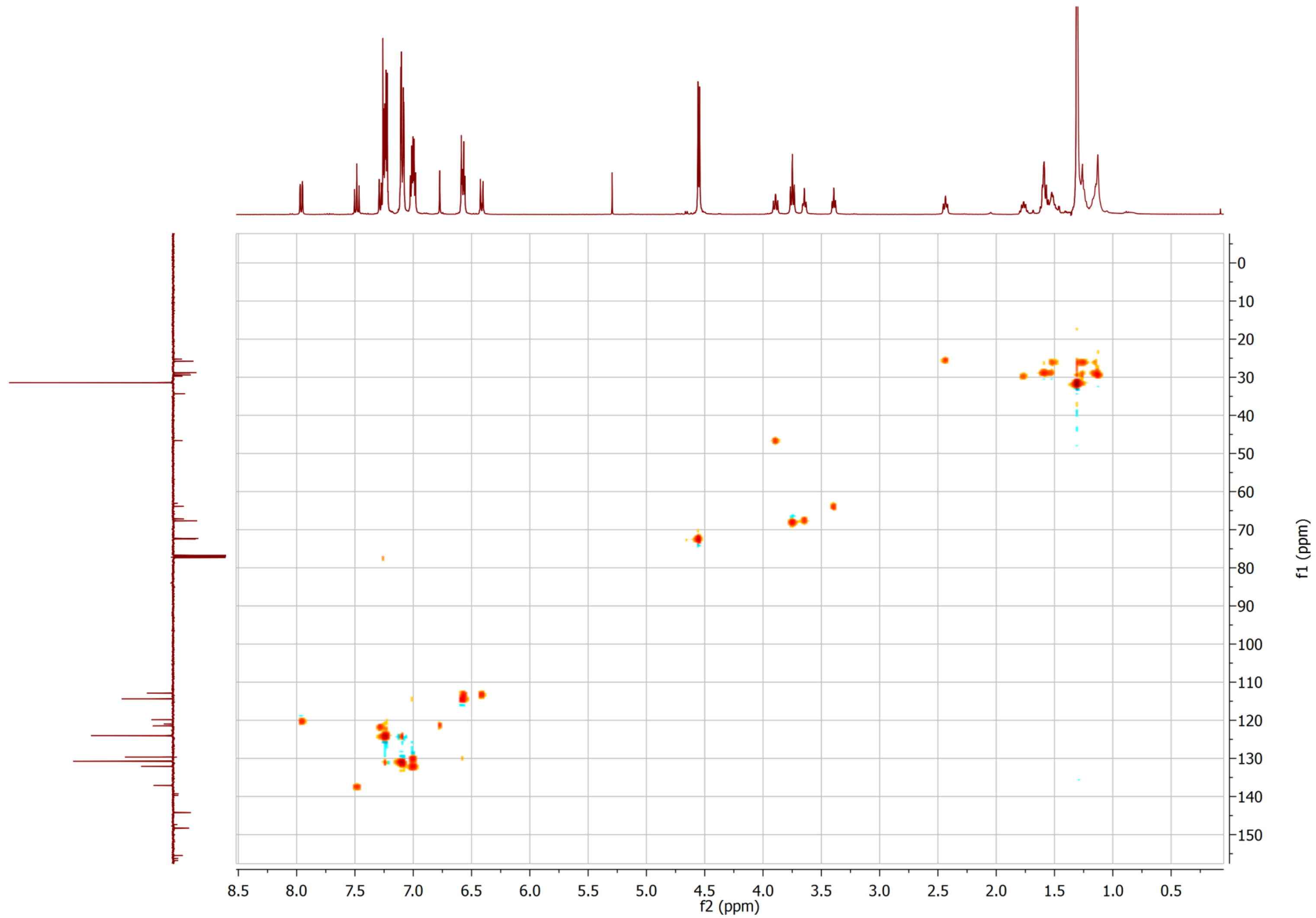

[2]Rotaxane S7 HMBC NMR ($CDCl_3$, 400 MHz, 300 K)

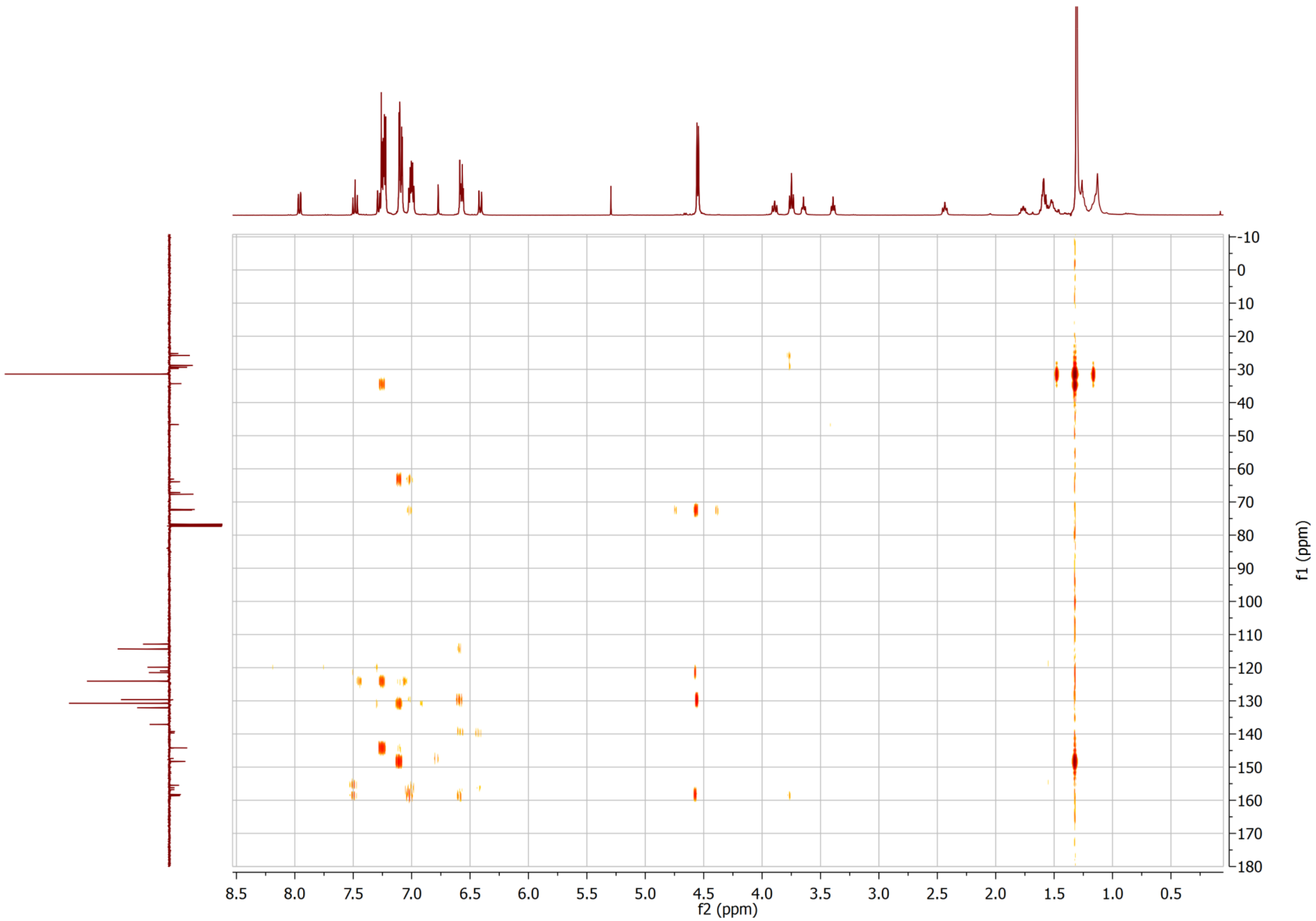

[3]Rotaxane S8 ^{1}H NMR ($CDCl_3$, 400 MHz, 300 K)

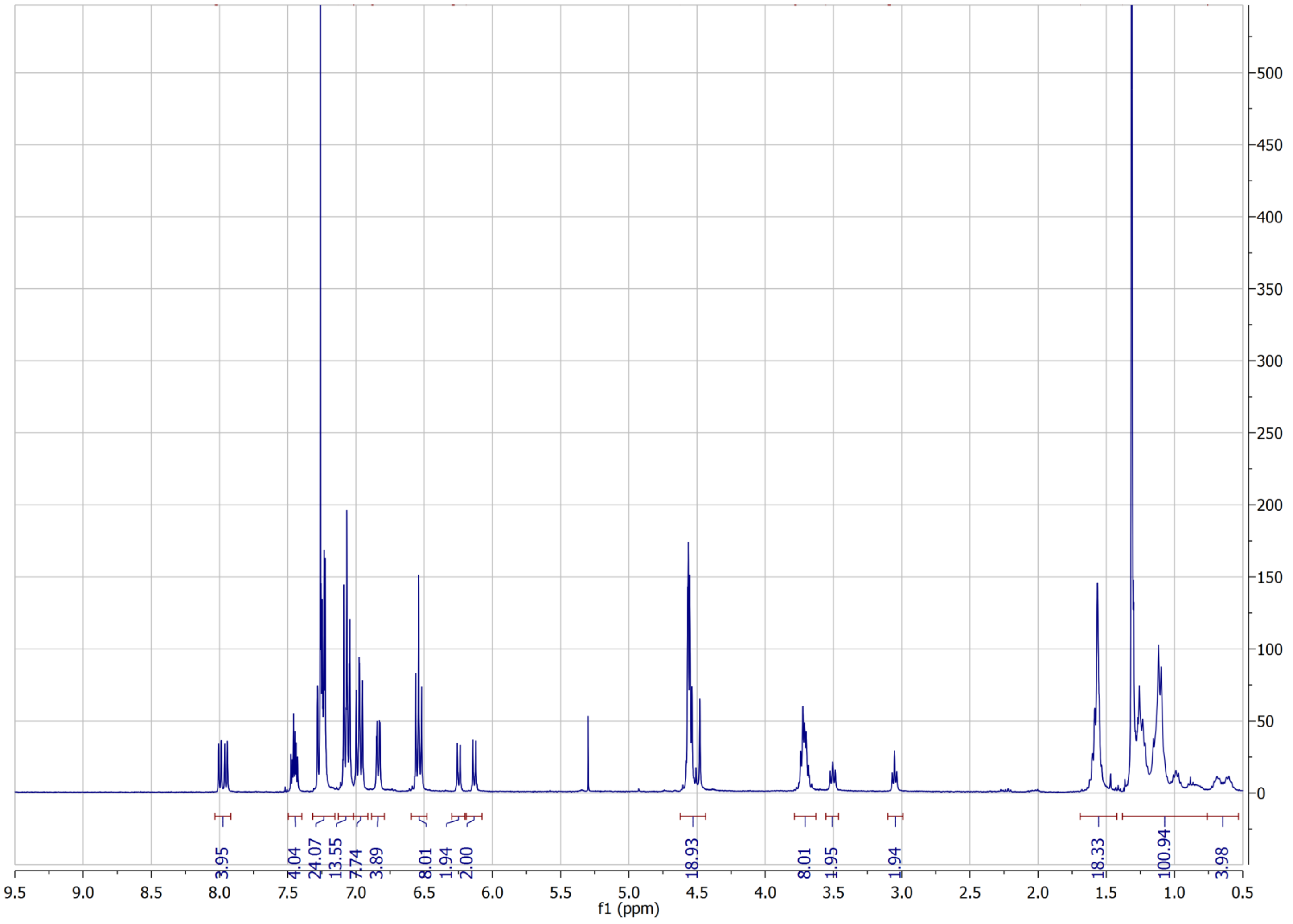

[3]Rotaxane S8 DEPTQ135 NMR ($CDCl_3$, 150 MHz, 300 K)

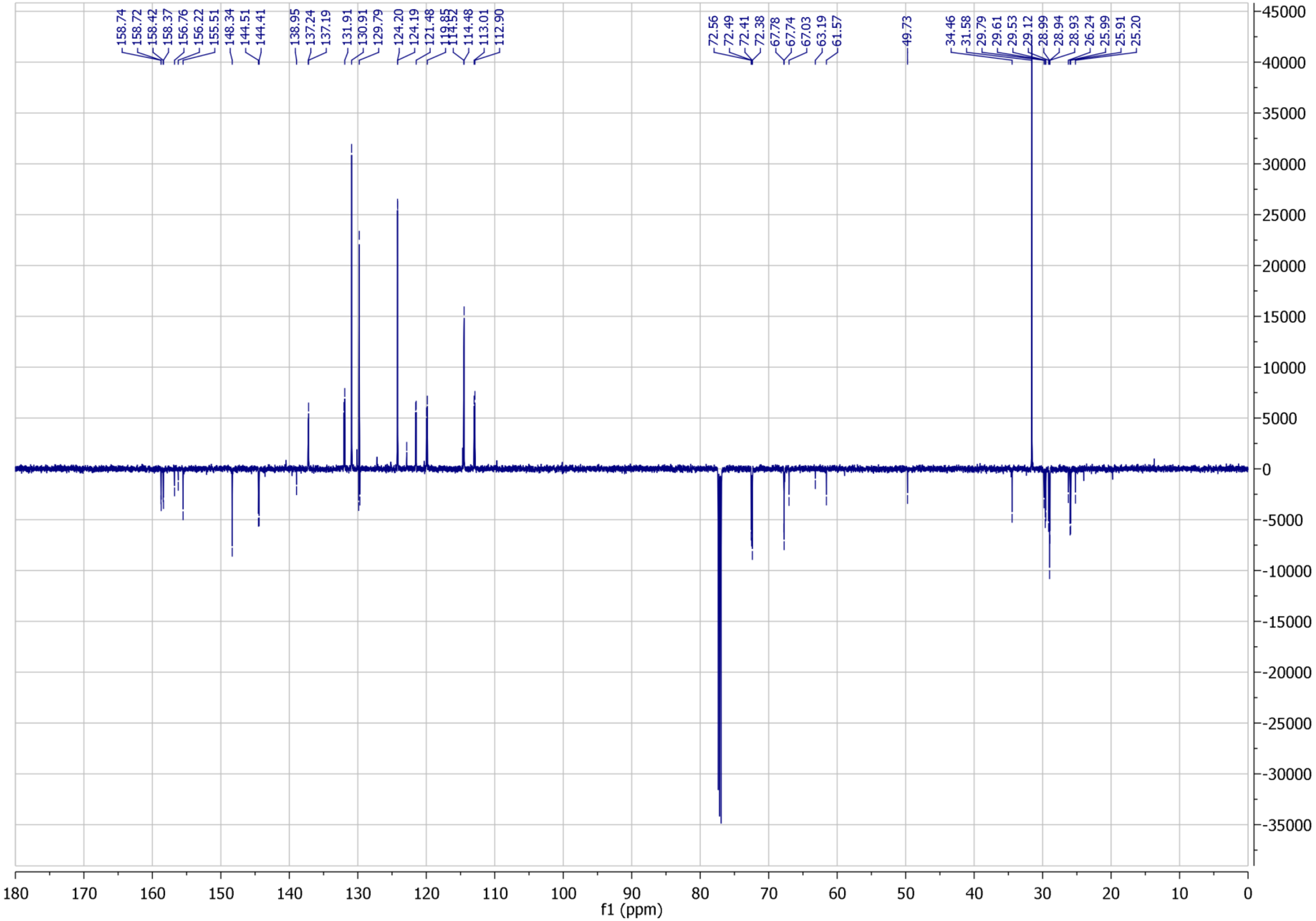

[3]Rotaxane S8 COSY ($CDCl_3$, 400 MHz, 300 K)

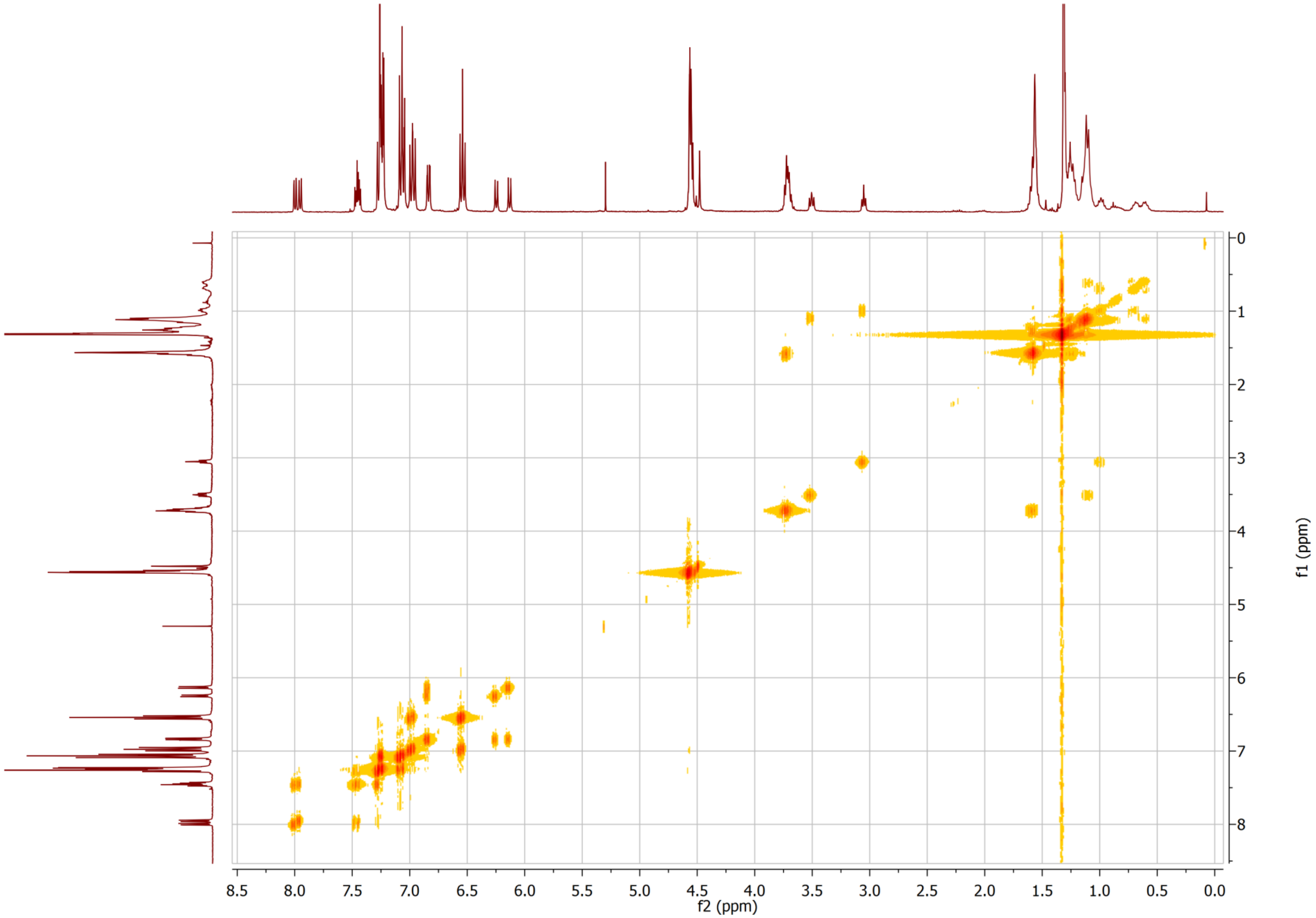

[3]Rotaxane S8 HSQC NMR ($CDCl_3$, 400 MHz, 300 K)

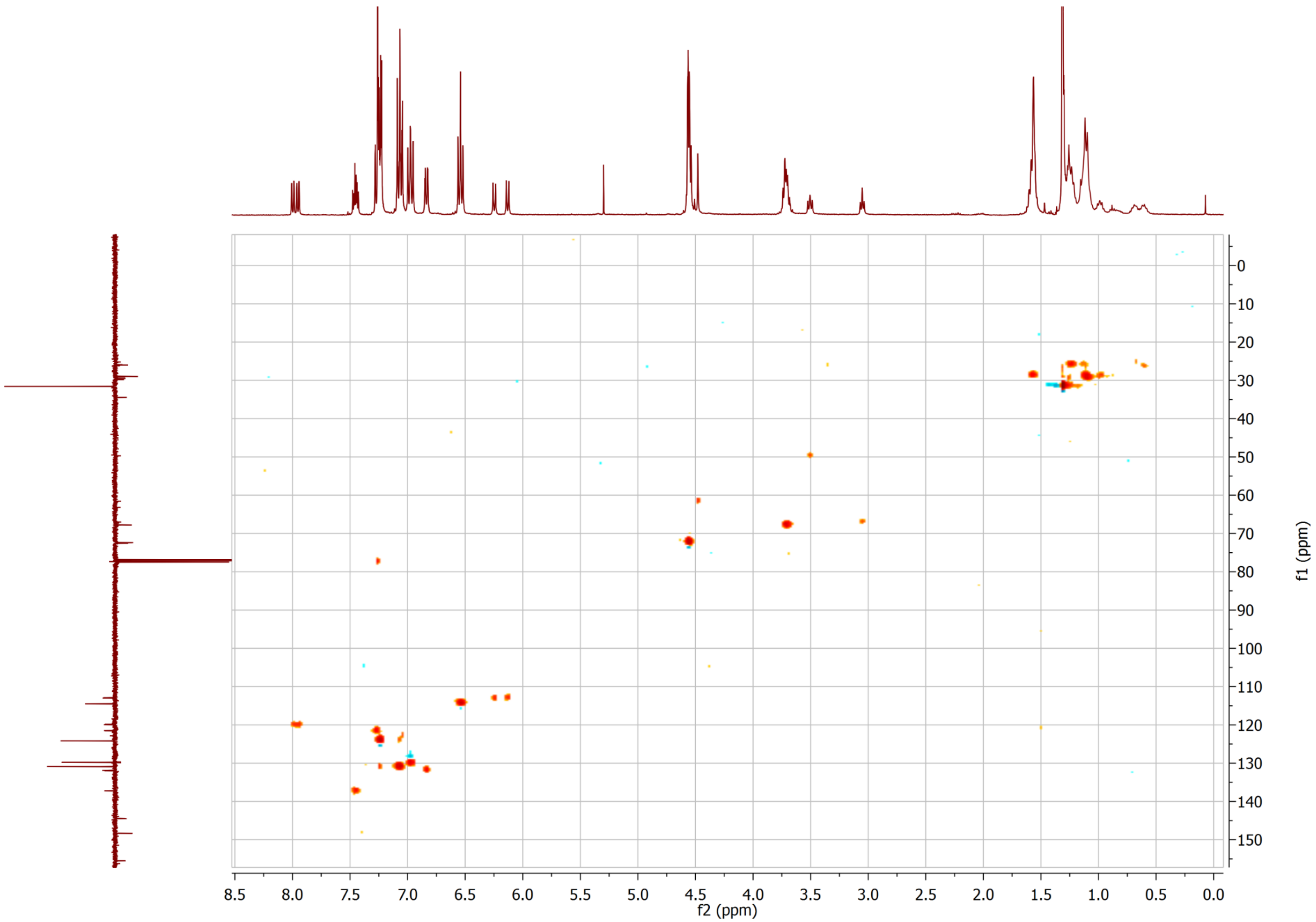

[3]Rotaxane S8 HMBC NMR ($CDCl_3$, 400 MHz, 300 K)

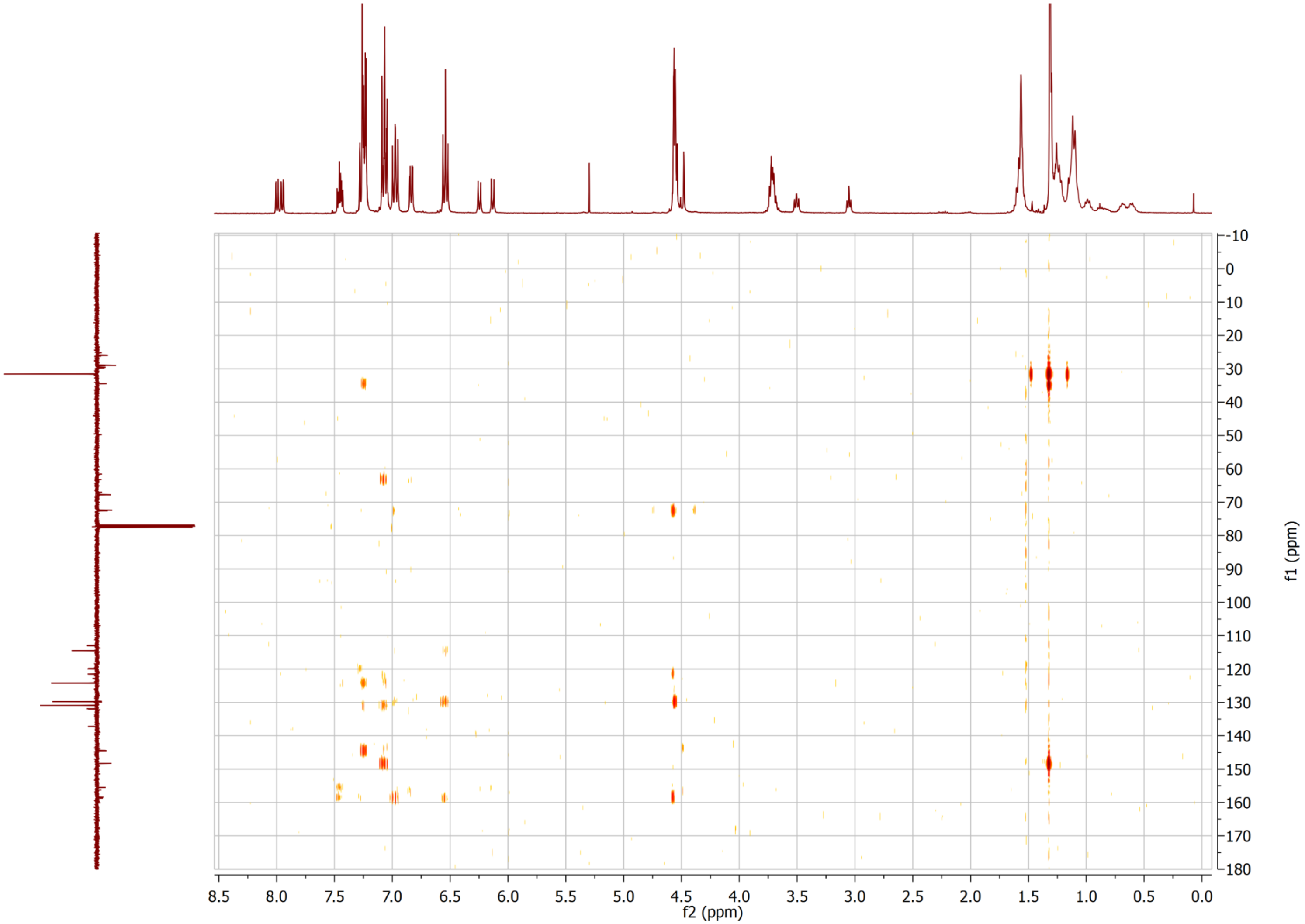

[2]Rotaxane S9 ^{1}H NMR ($CDCl_3$, 400 MHz, 300 K)

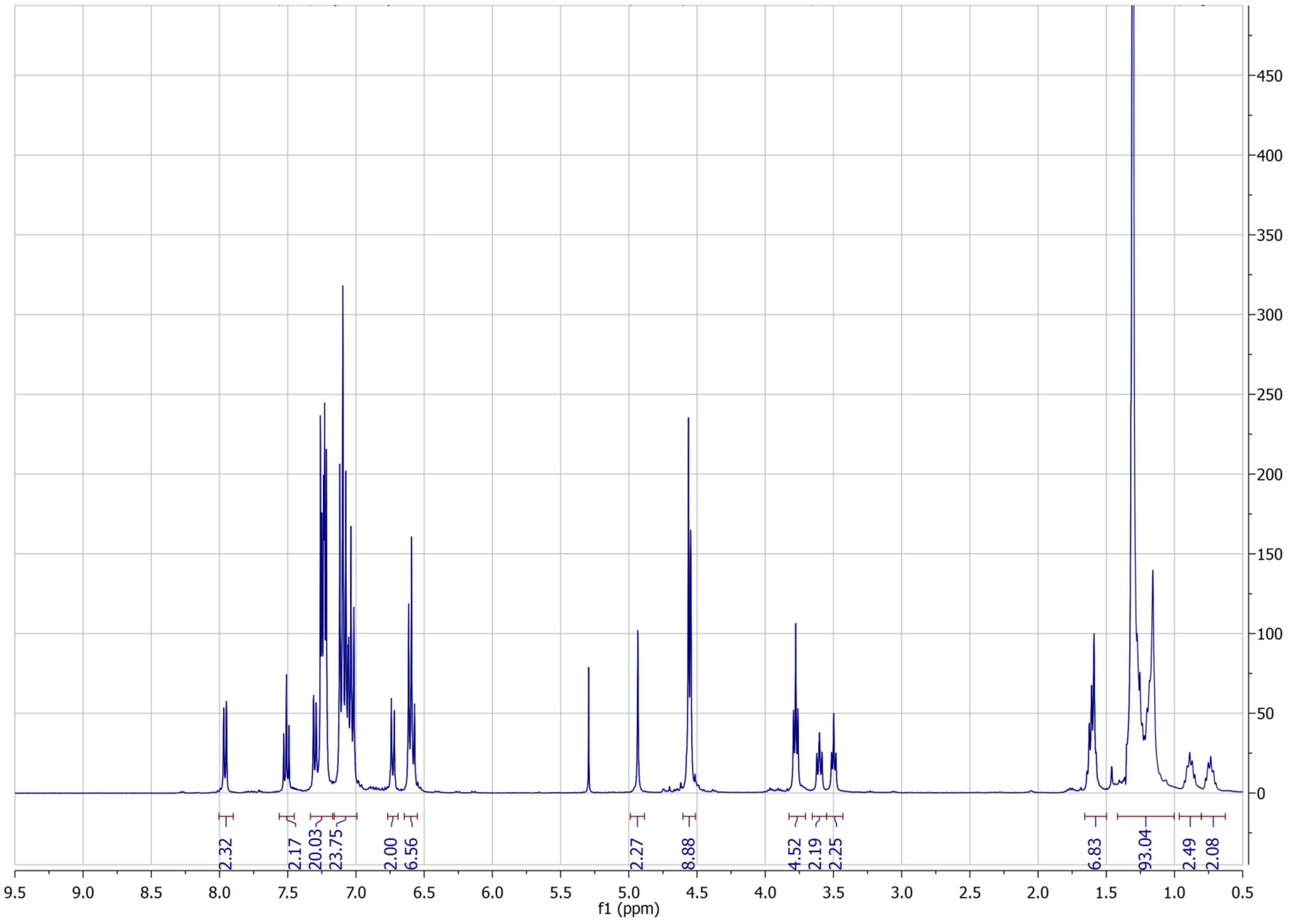

[2]Rotaxane S9 DEPTQ135 NMR ($CDCl_3$, 100 MHz, 300 K)

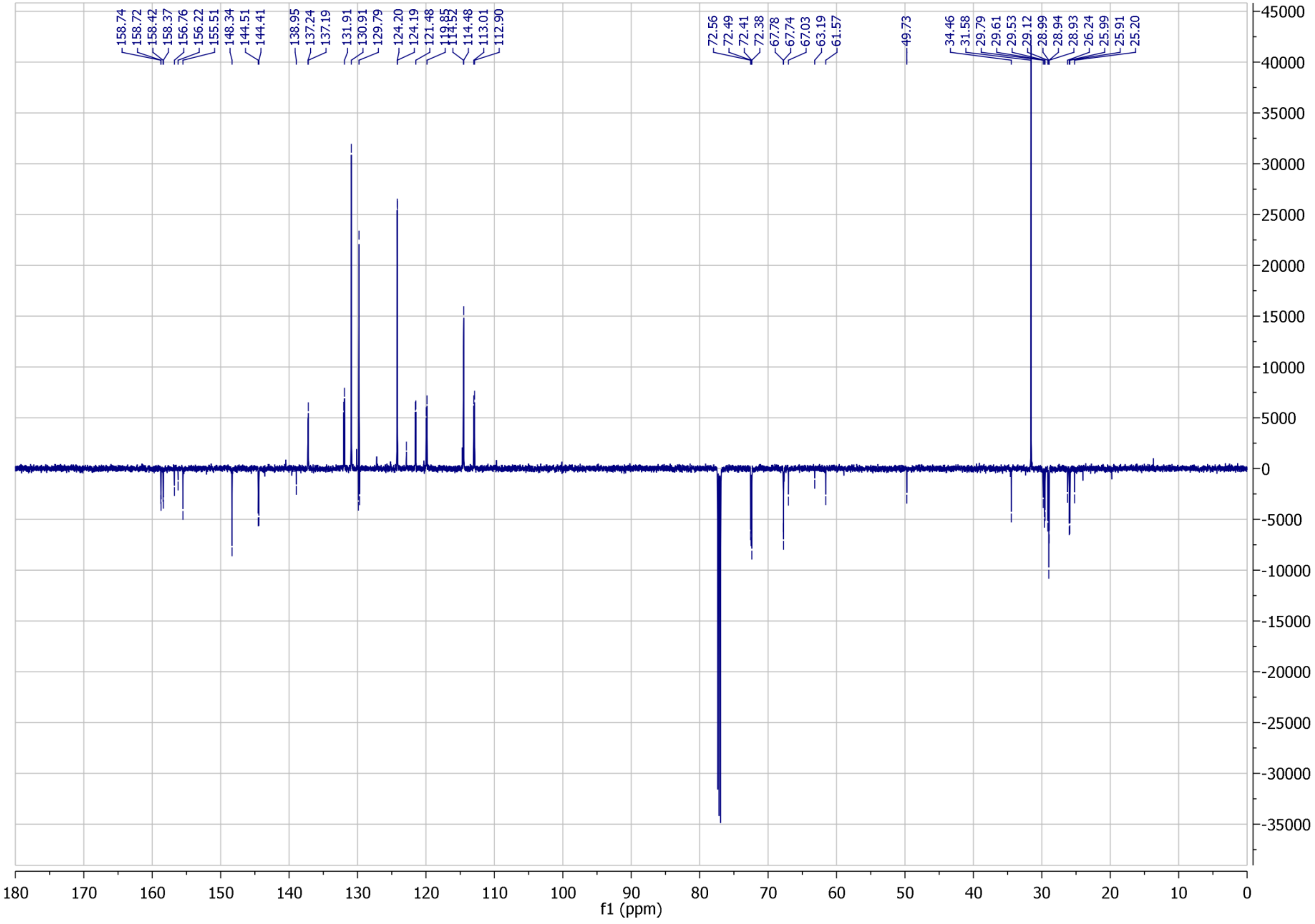

[2]Rotaxane S9 COSY ($CDCl_3$, 400 MHz, 300 K)

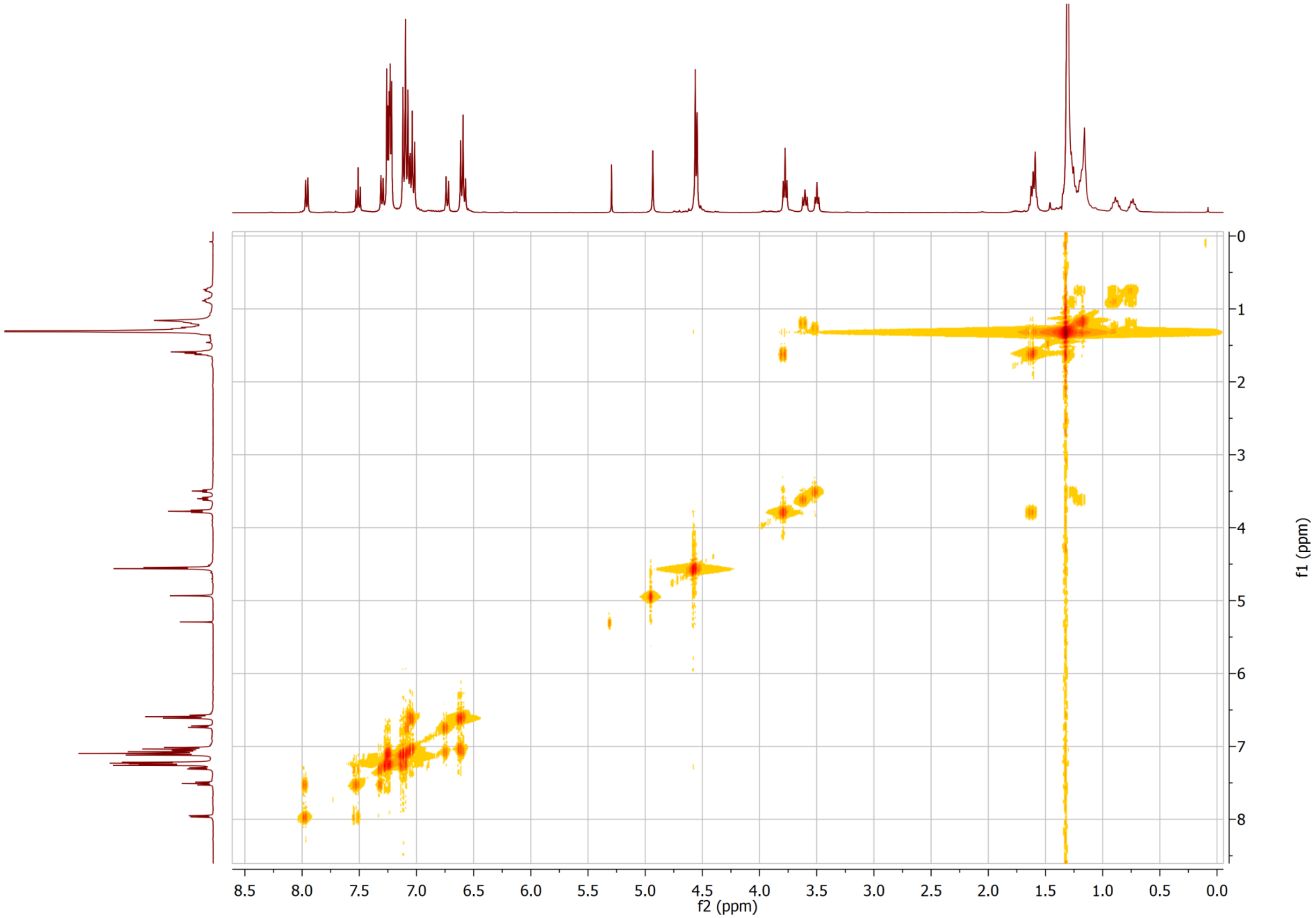

[2]Rotaxane S9 HSQC NMR ($CDCl_3$, 400 MHz, 300 K)

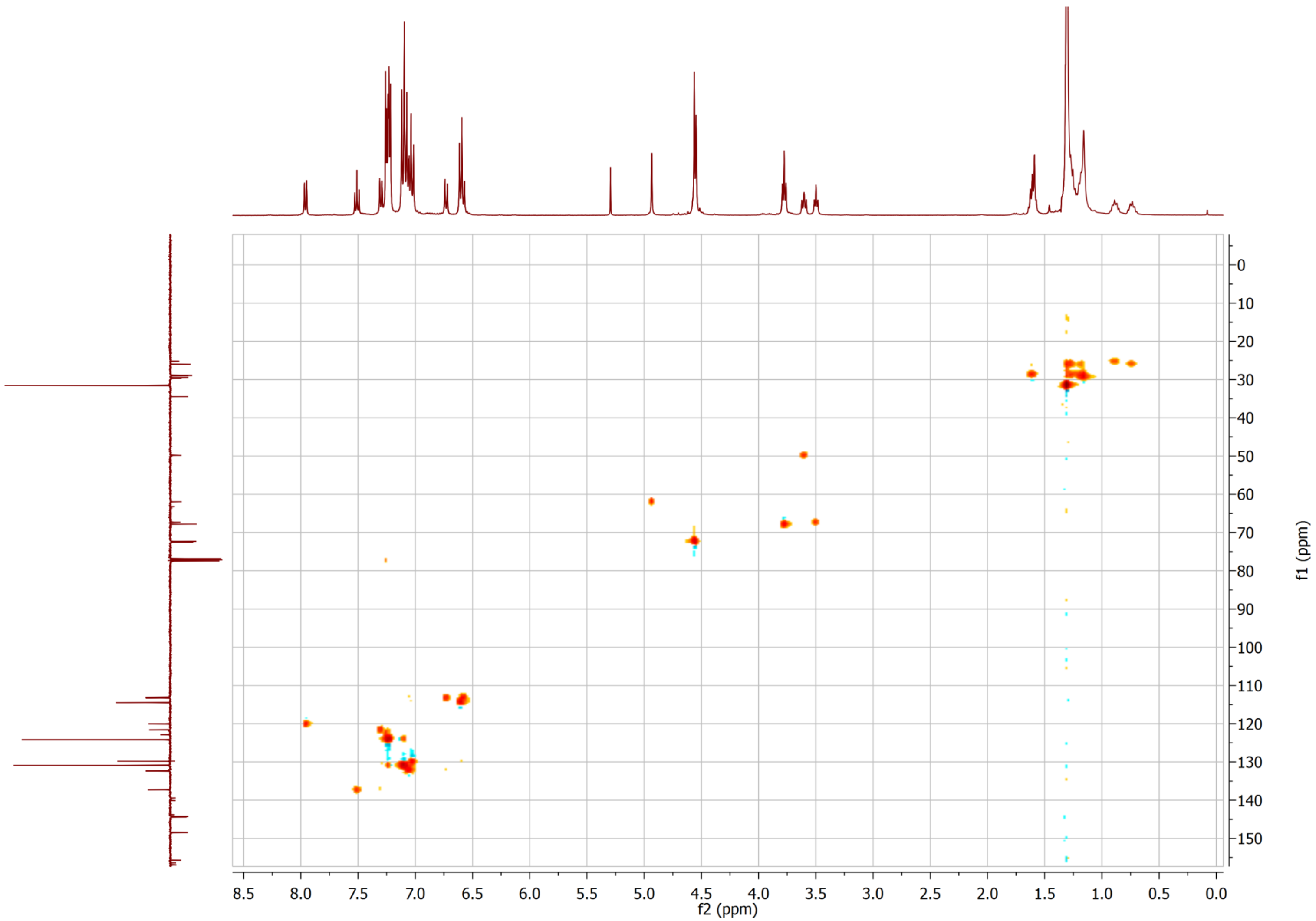

[2]Rotaxane S9 HMBC NMR ($CDCl_3$, 400 MHz, 300 K)

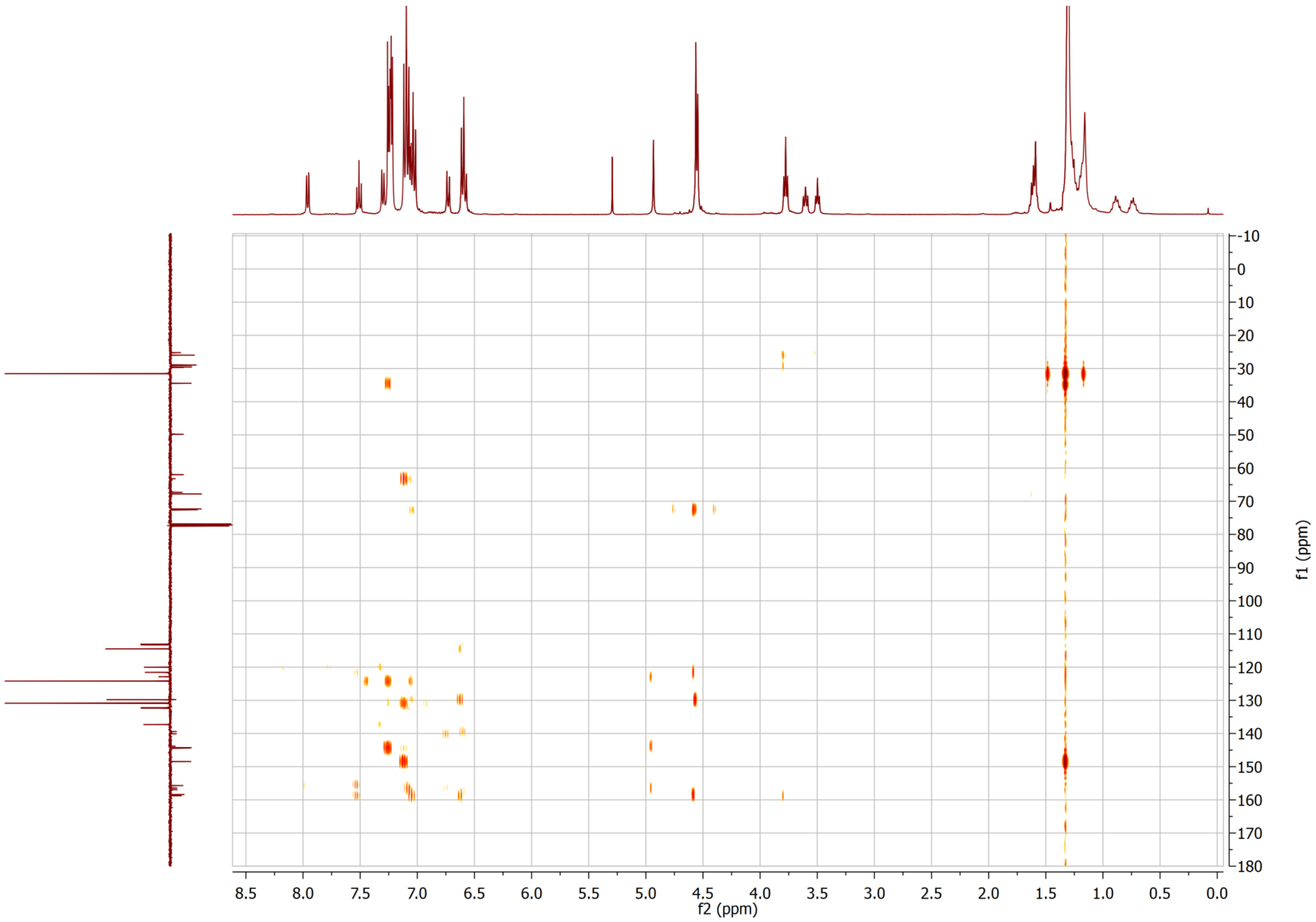

[3]Rotaxane S10a ^{1}H NMR (CDCl$_3$, 400 MHz, 300 K)

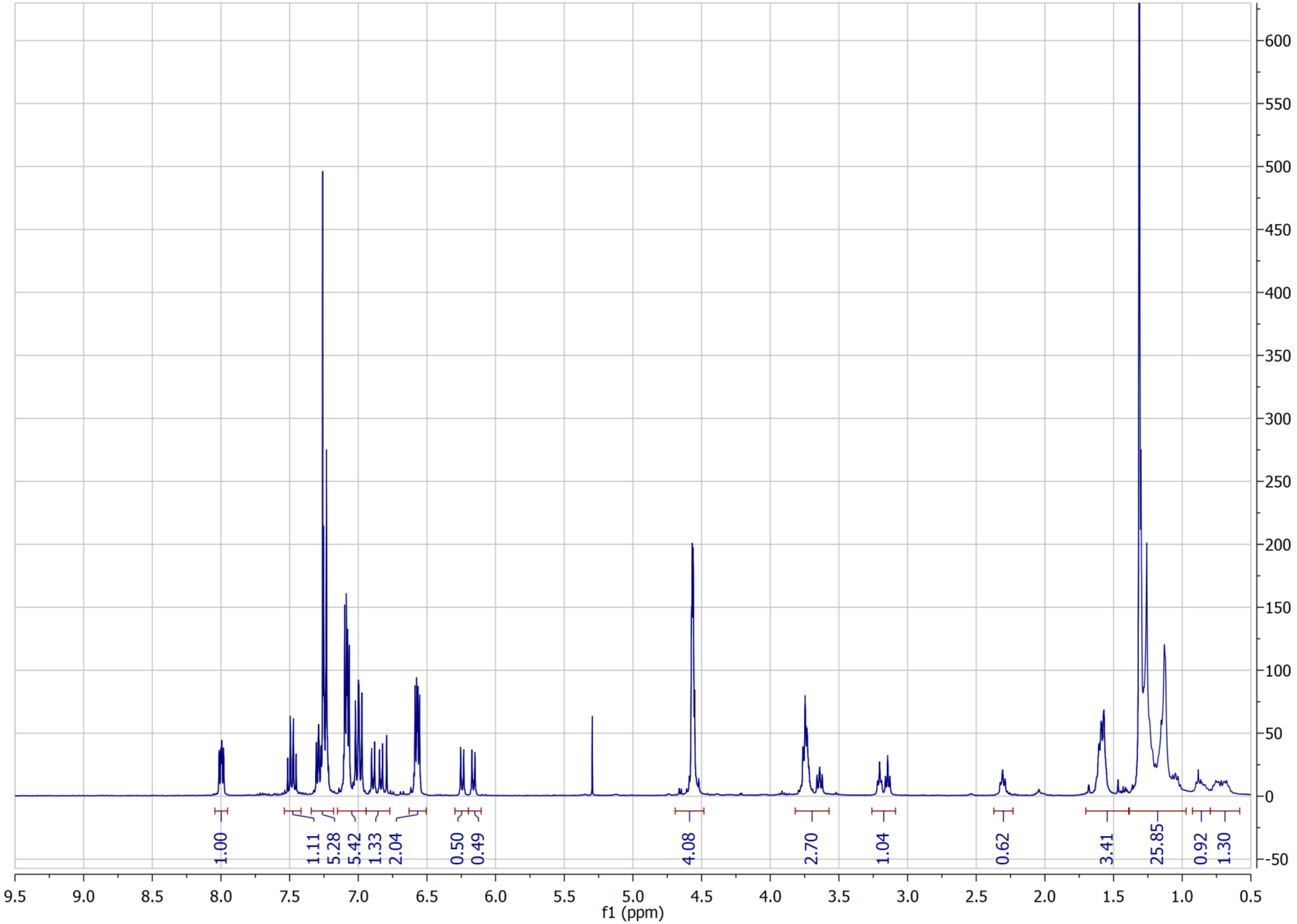

[3]Rotaxane S10a DEPTQ135 NMR ($CDCl_3$, 150 MHz, 300 K)

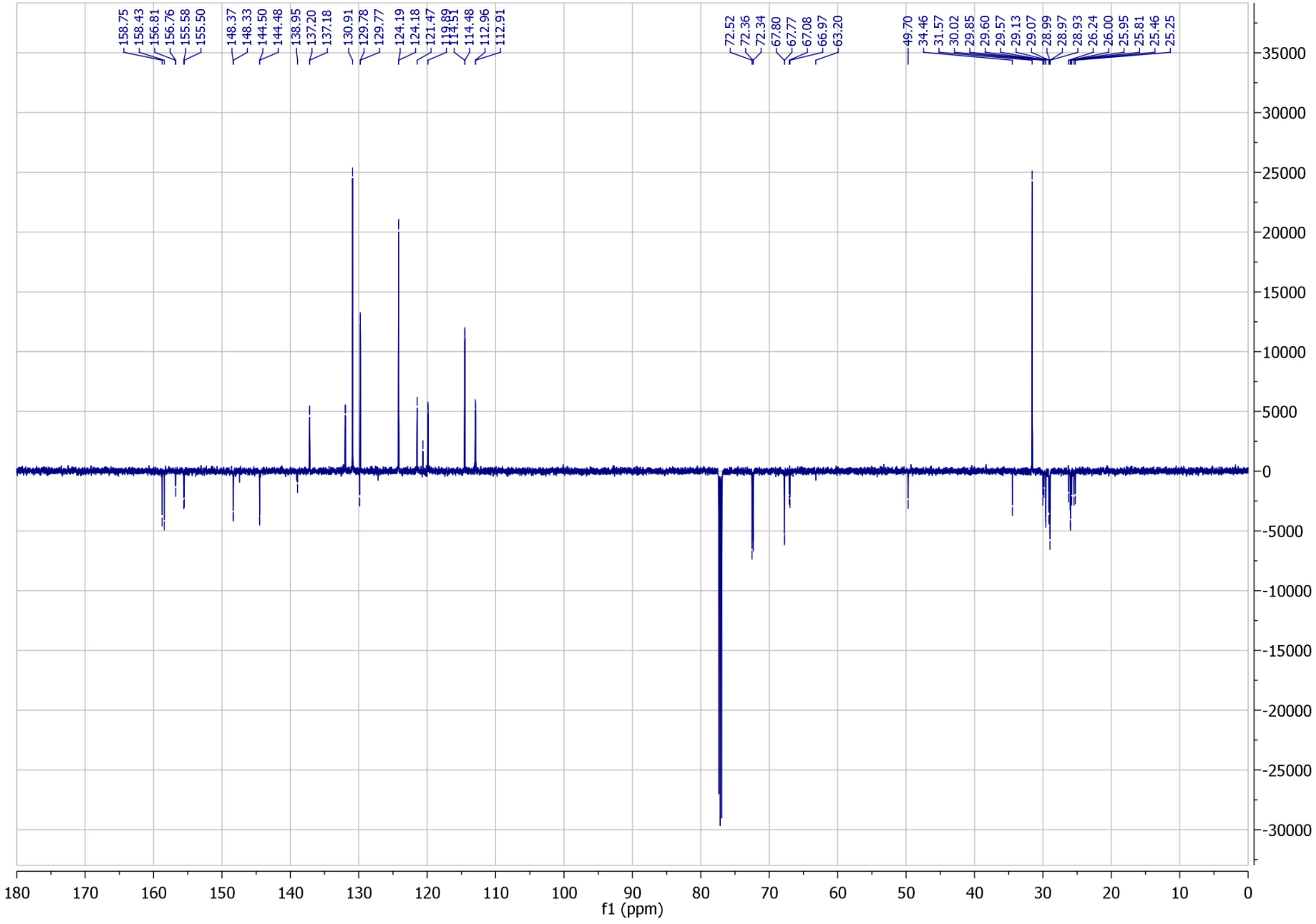

[3]Rotaxane S10a COSY ($CDCl_3$, 600 MHz, 300 K)

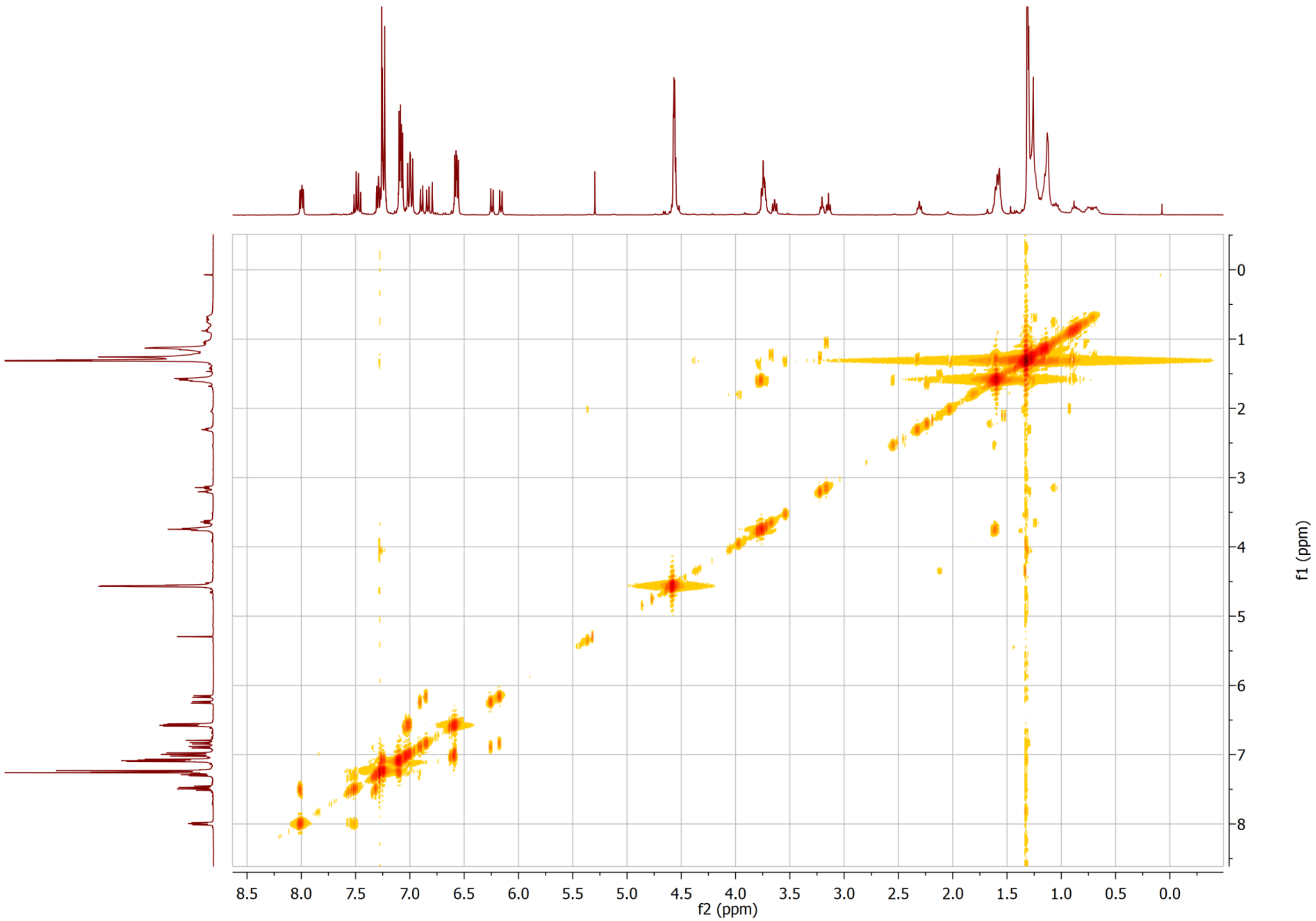

[3]Rotaxane S10a HSQC NMR ($CDCl_3$, 600 MHz, 300 K)

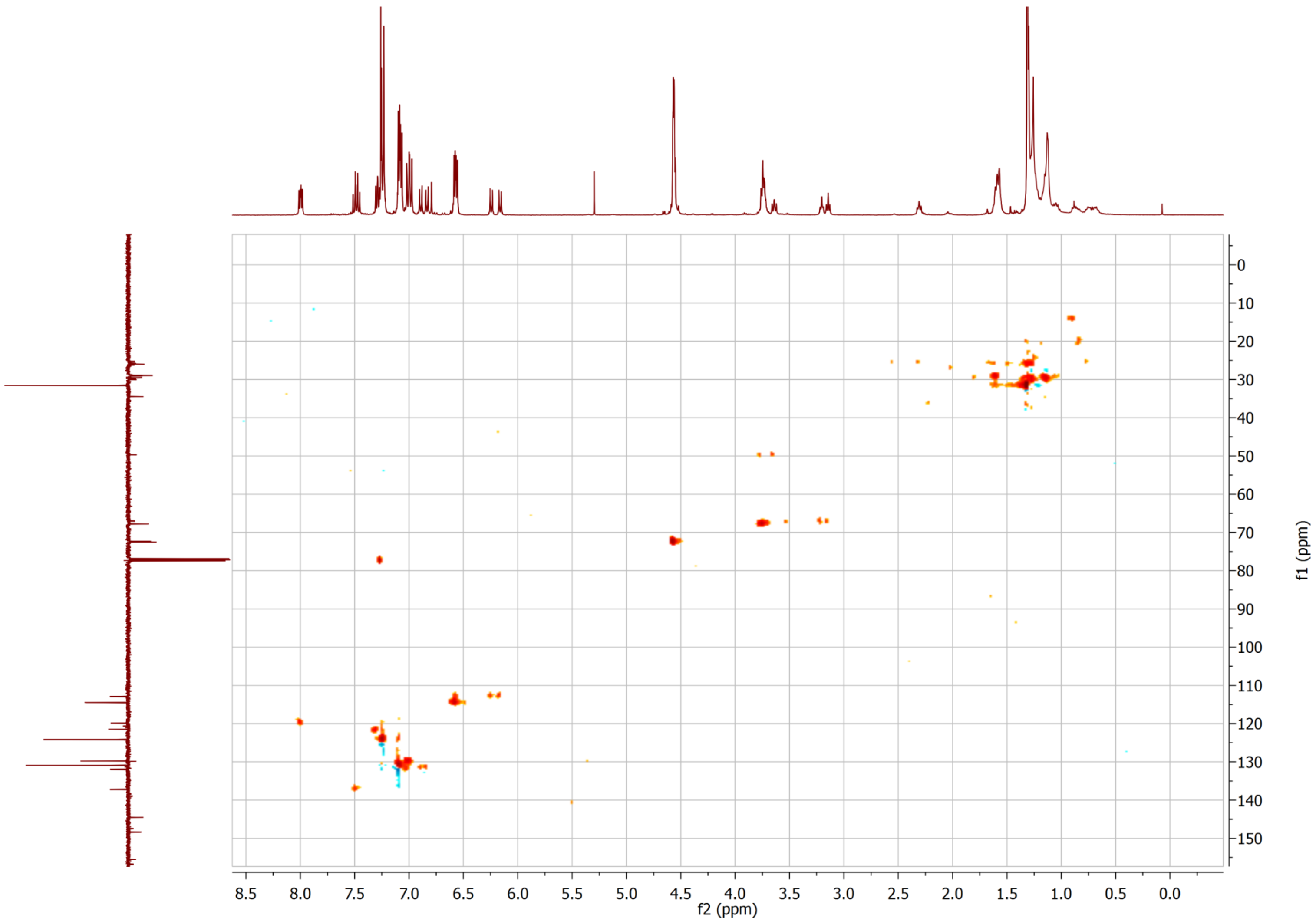

[3]Rotaxane S10a HMBC NMR ($CDCl_3$, 600 MHz, 300 K)

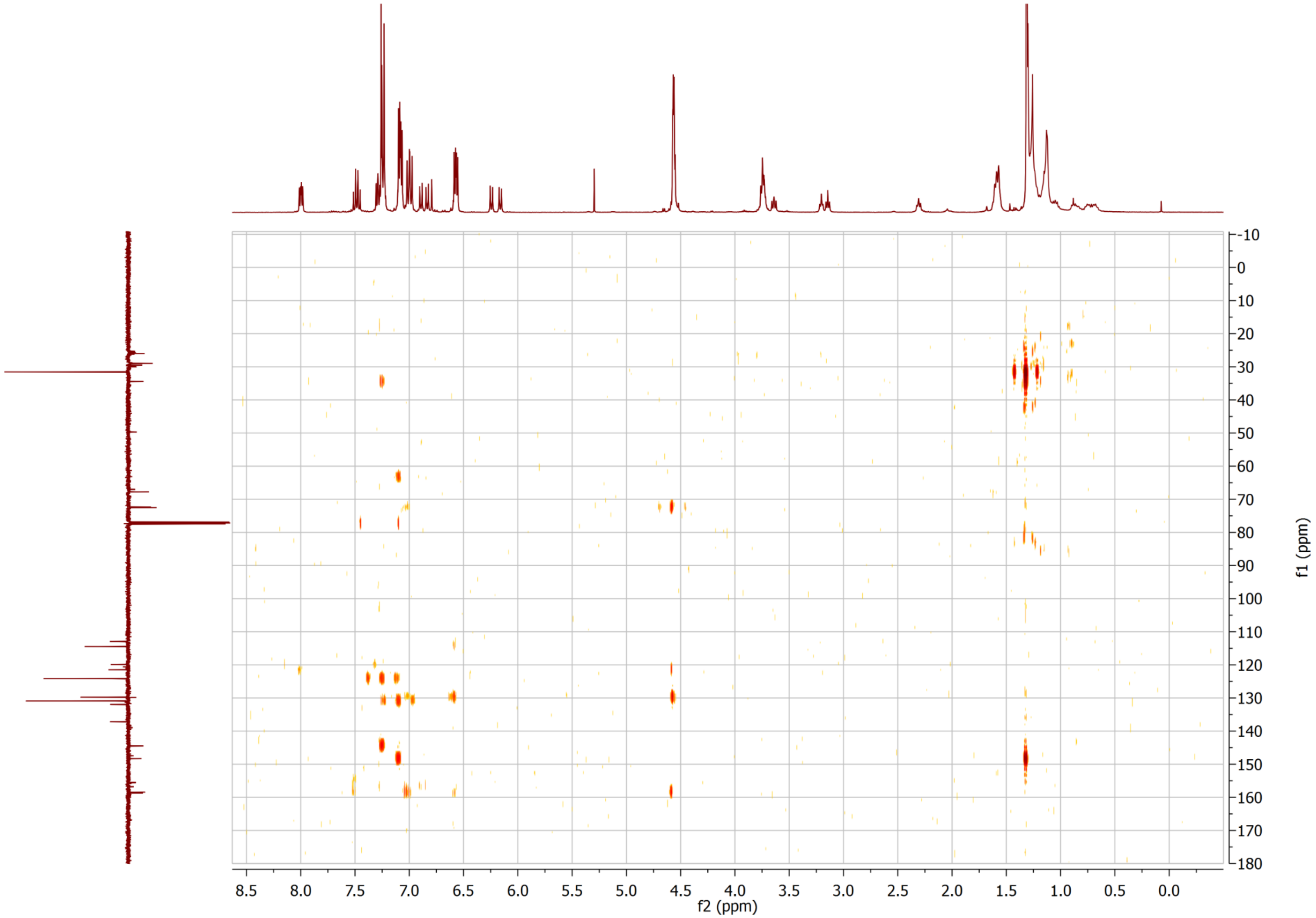

[2]Rotaxane S11a ^1H NMR ($CDCl_3$, 400 MHz, 300 K)

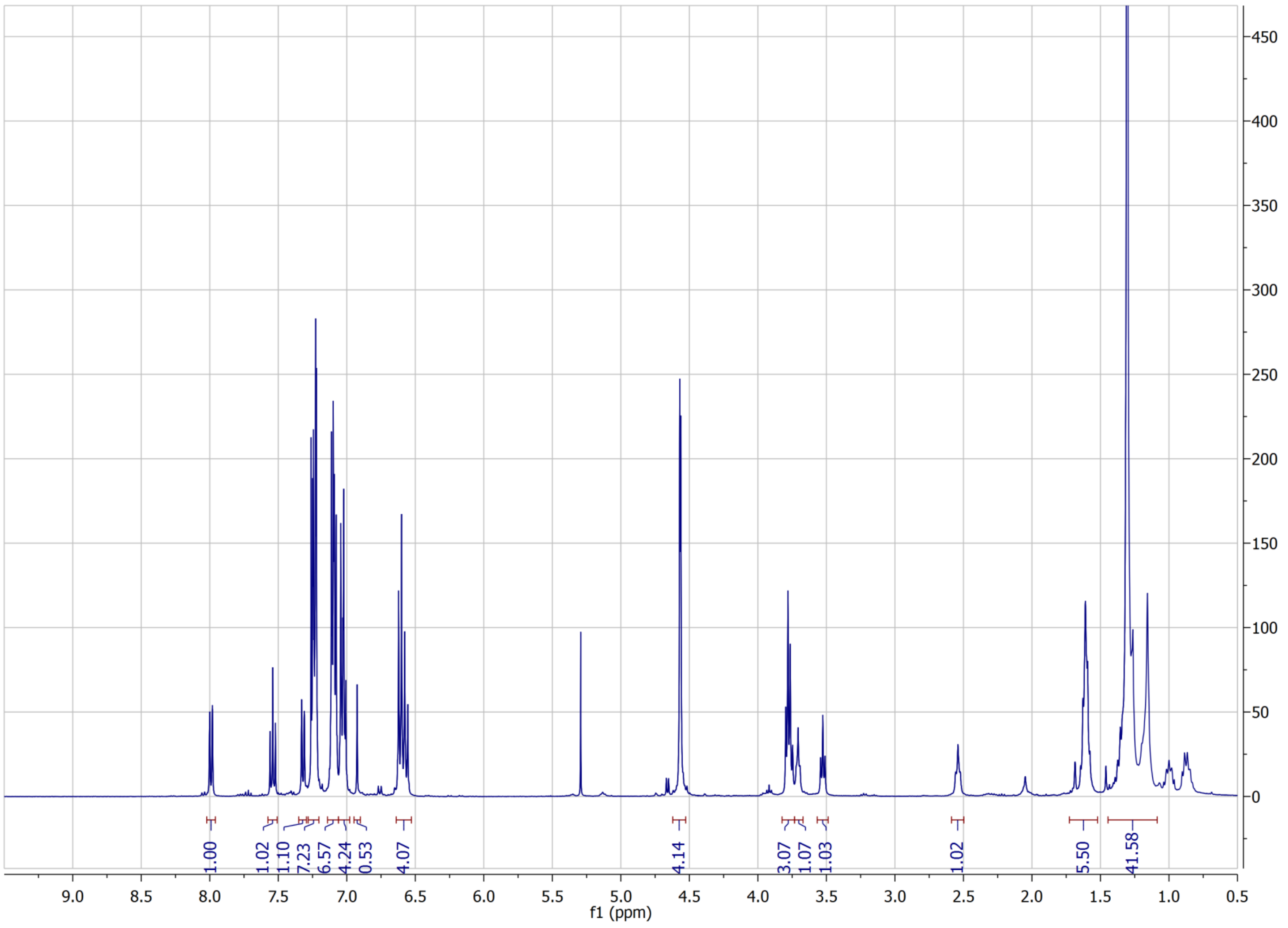

[2]Rotaxane S11a DEPTQ135 NMR ($CDCl_3$, 100 MHz, 300 K)

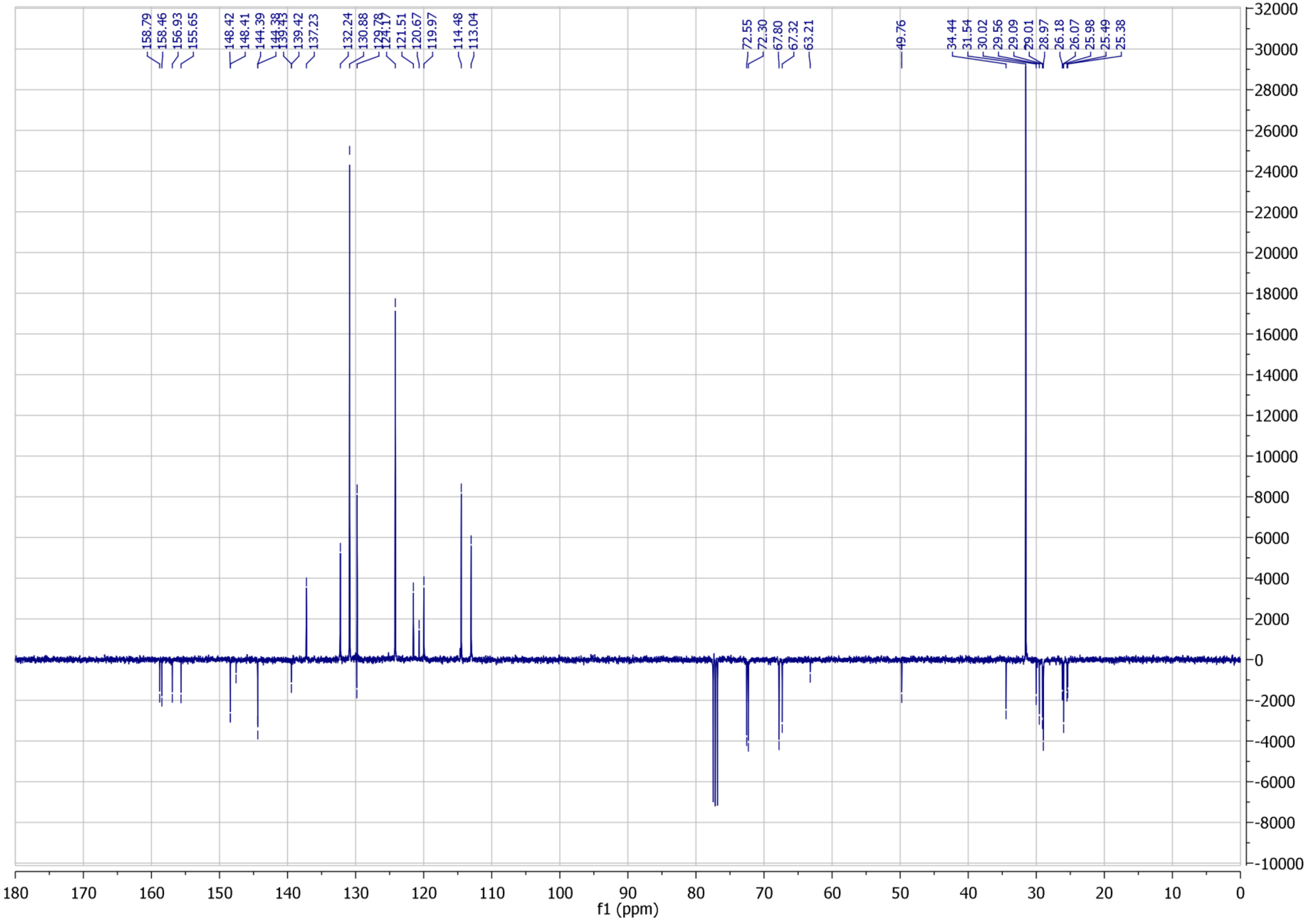

[2]Rotaxane S11a COSY ($CDCl_3$, 400 MHz, 300 K)

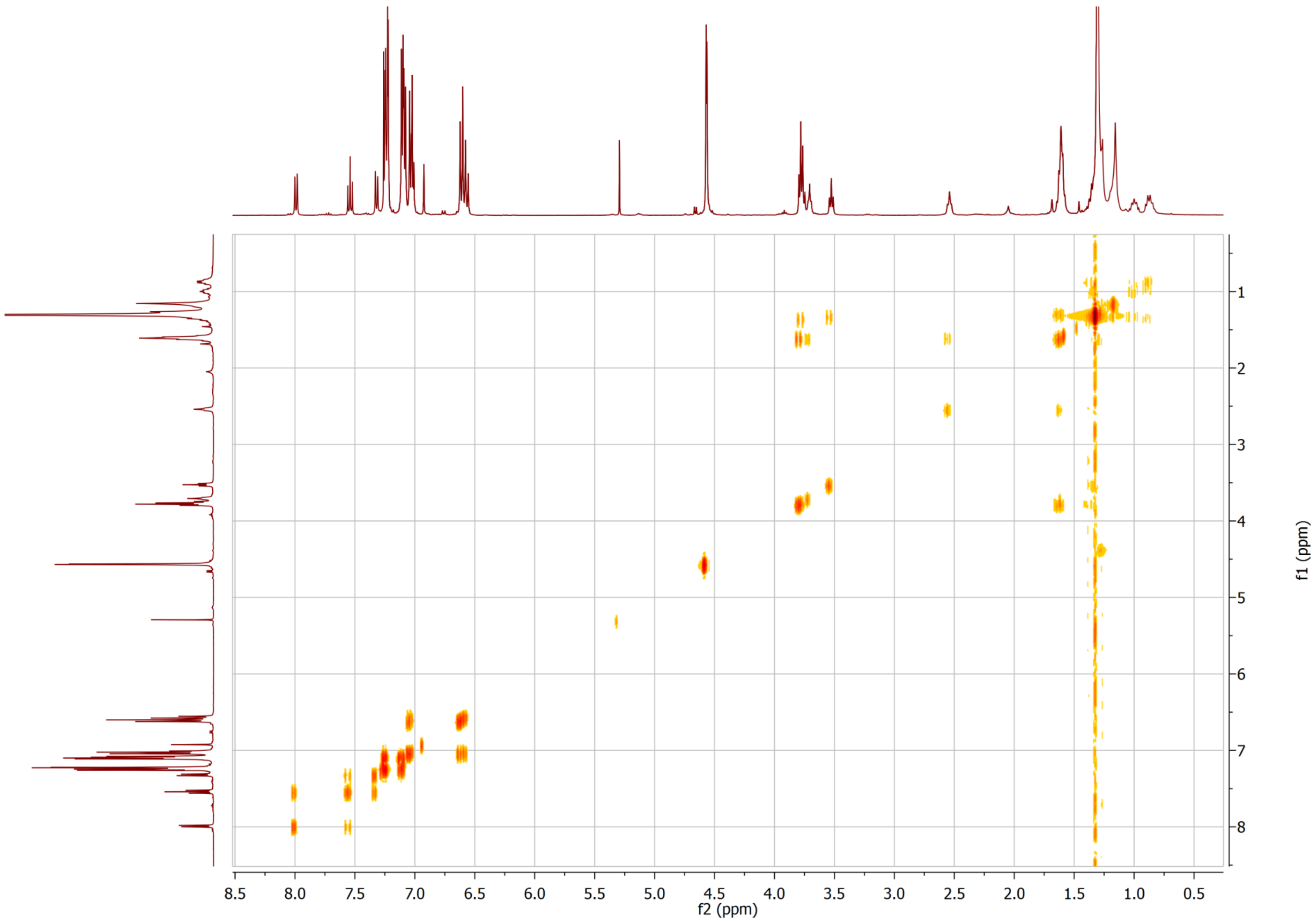

[2]Rotaxane S11a HSQC NMR ($CDCl_3$, 400 MHz, 300 K)

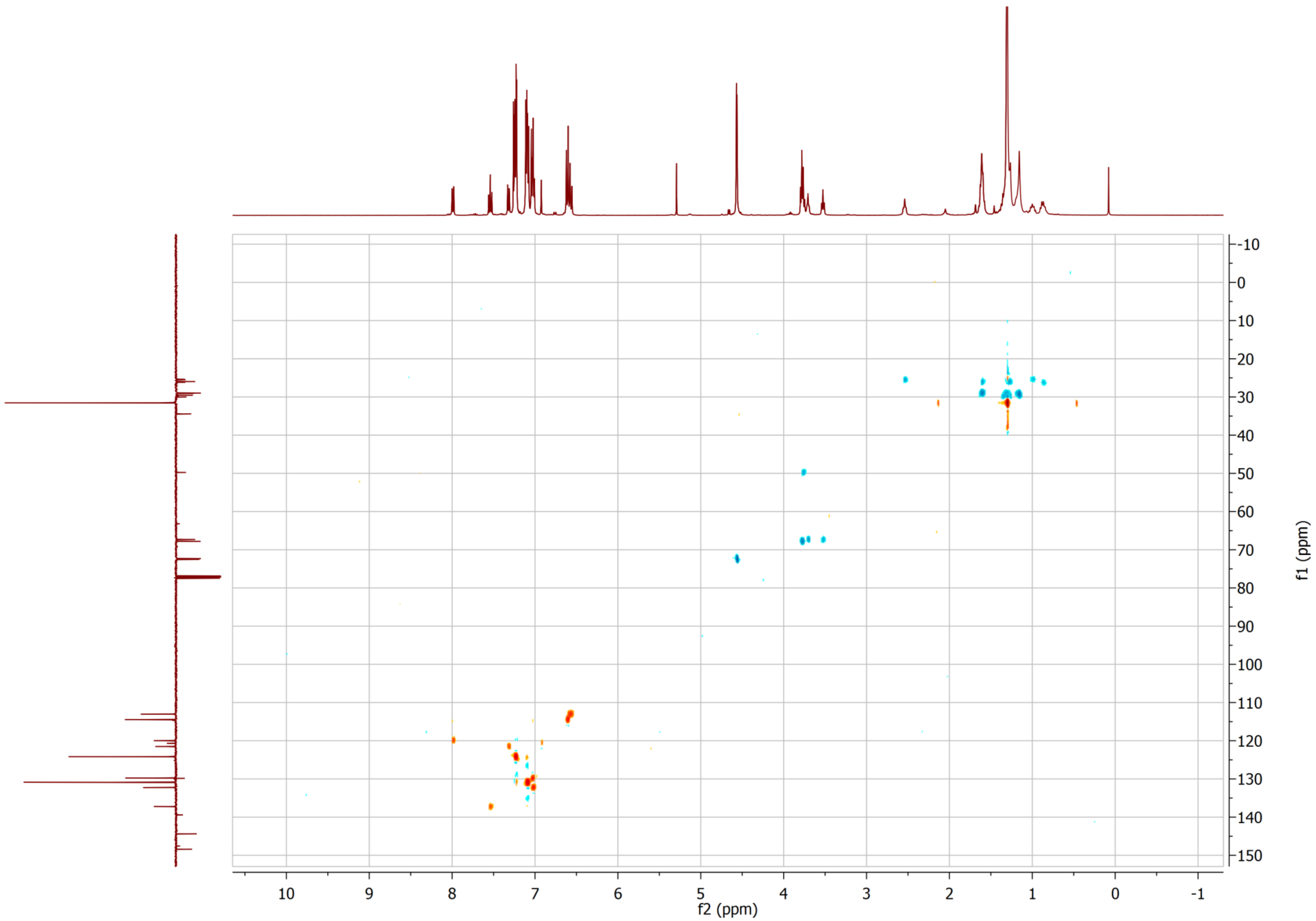

[2]Rotaxane S11a HMBC NMR ($CDCl_3$, 400 MHz, 300 K)

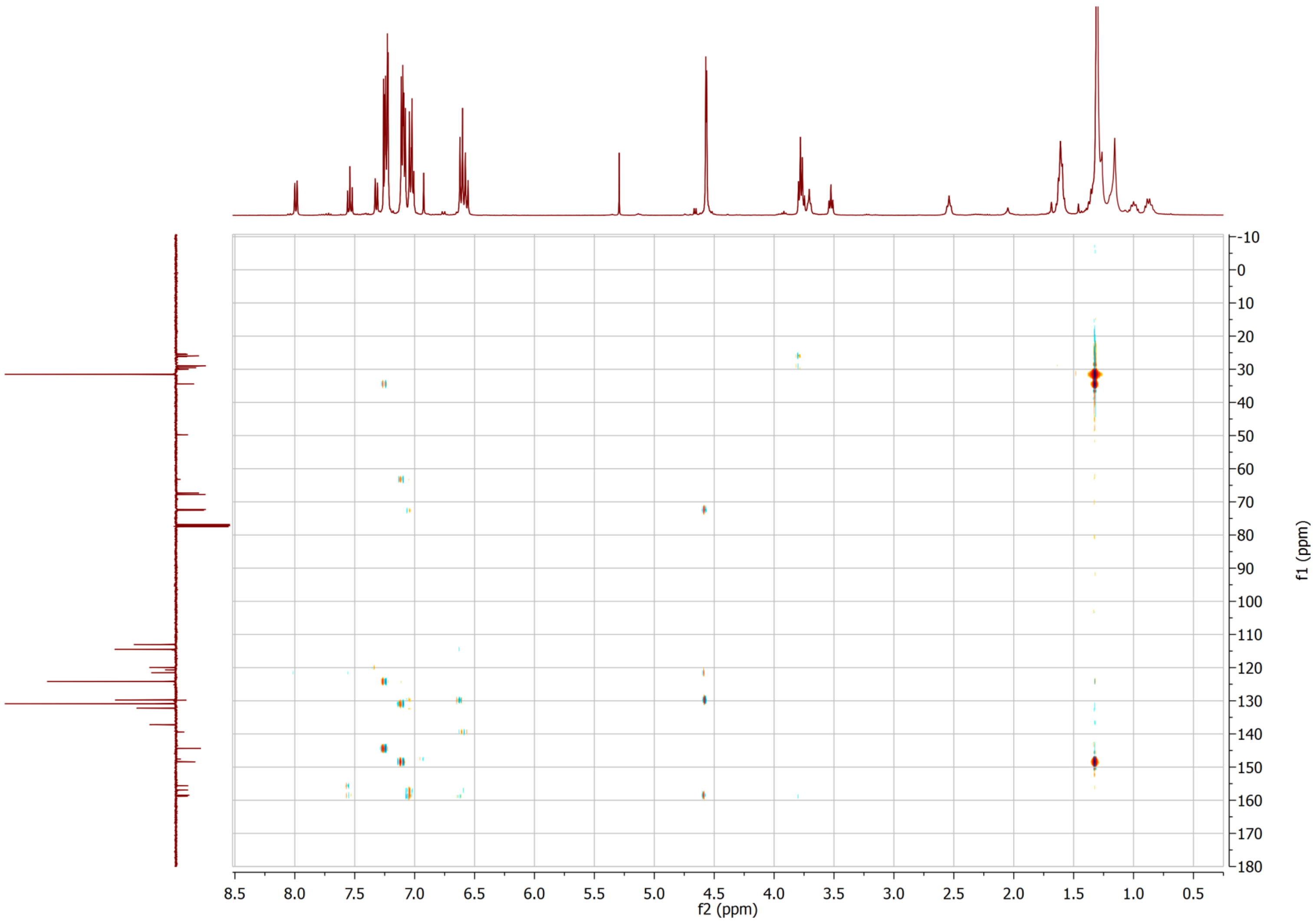

[2]Rotaxane S11d ^{1}H NMR ($CDCl_3$, 400 MHz, 300 K)

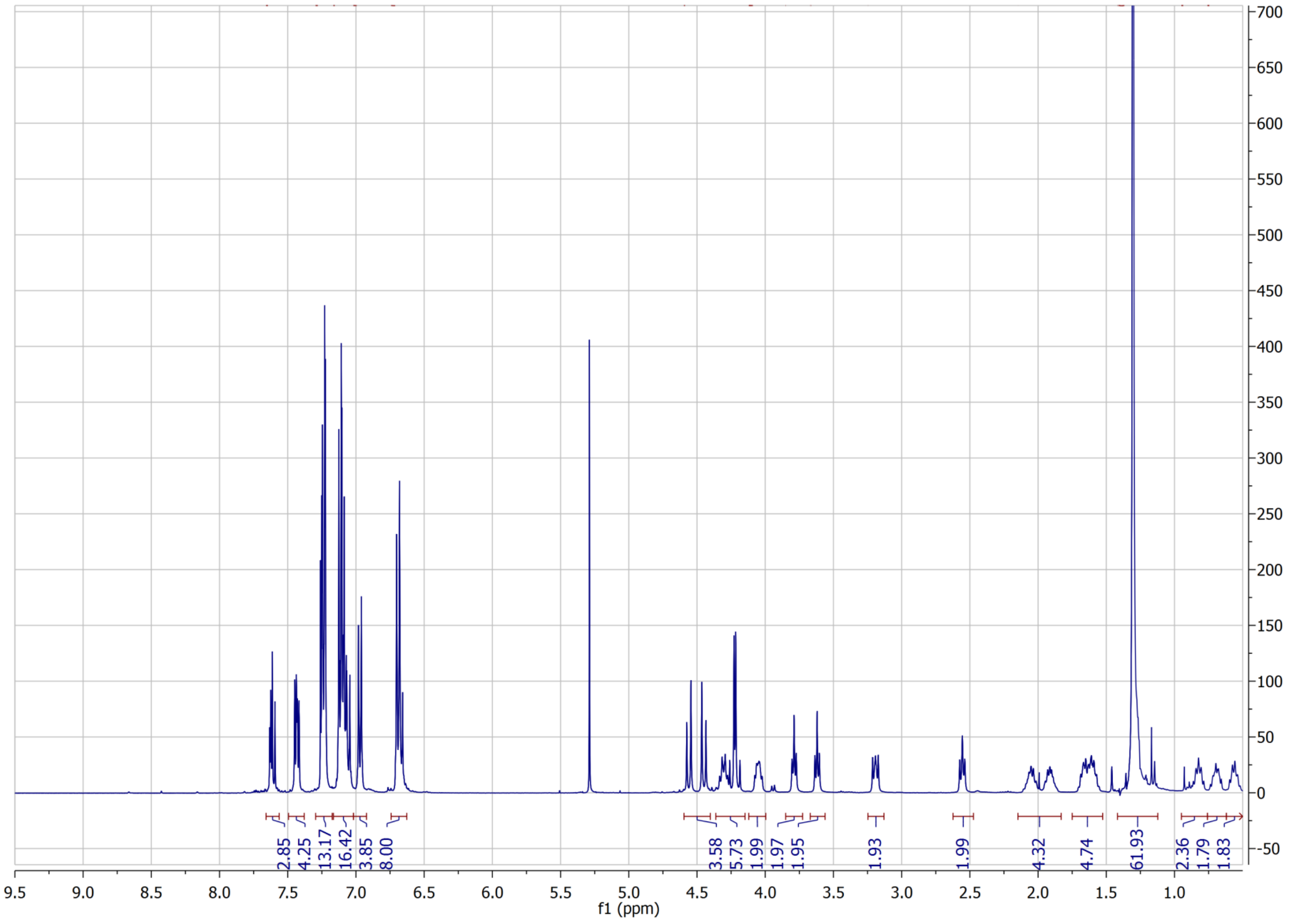

[2]Rotaxane S11d DEPTQ135 NMR ($CDCl_3$, 100 MHz, 300 K)

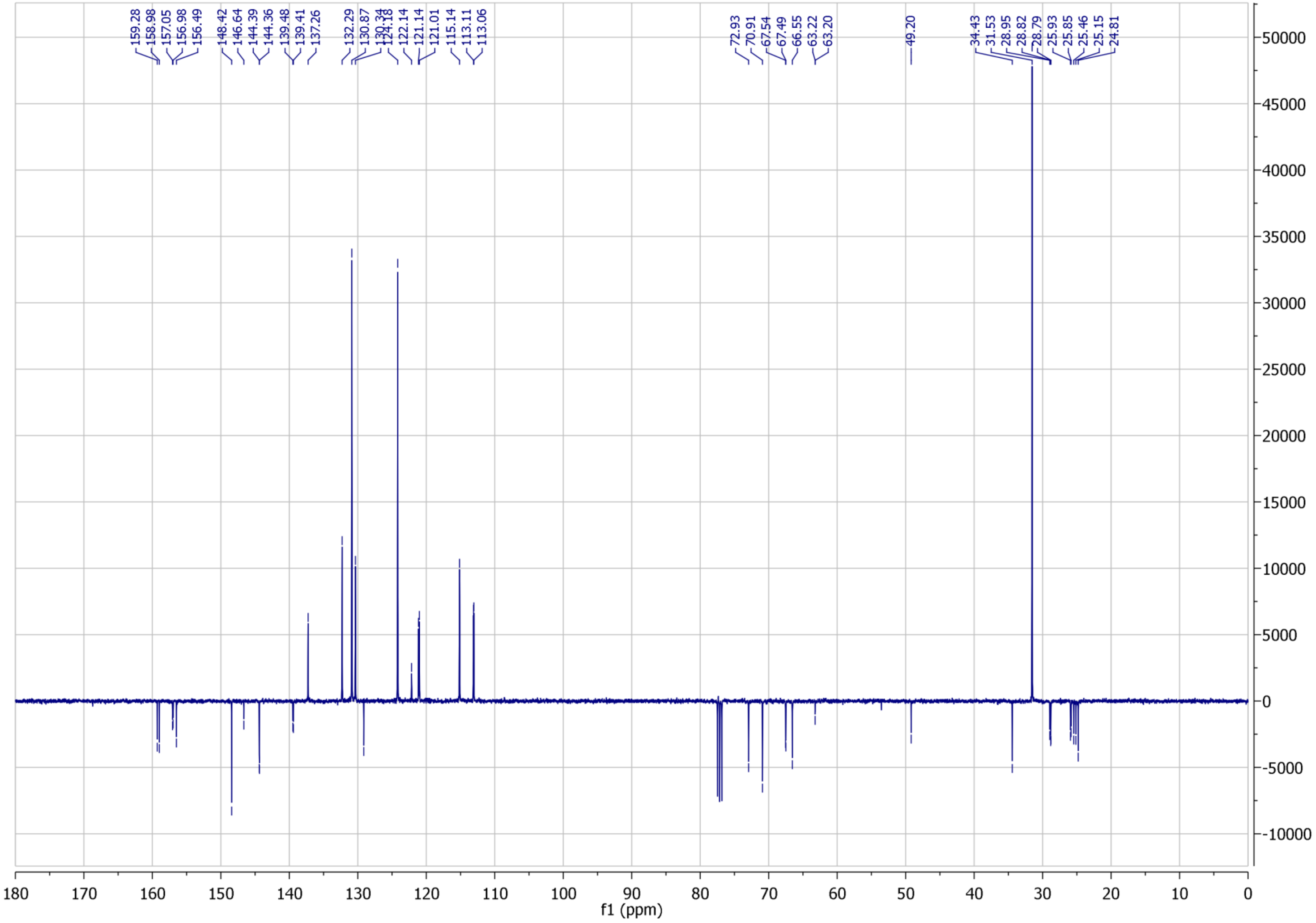

[2]Rotaxane S11d COSY ($CDCl_3$, 600 MHz, 300 K)

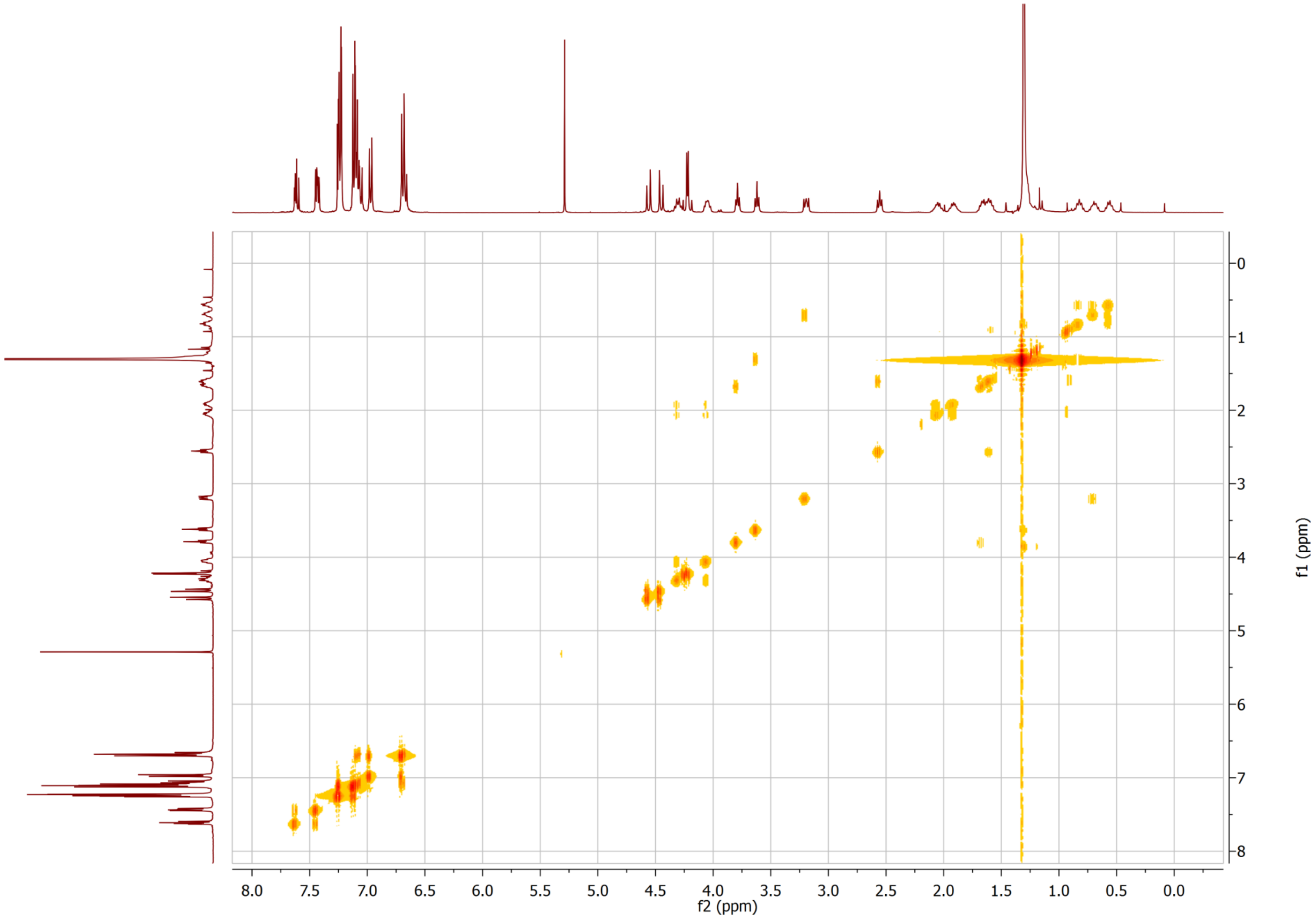

[2]Rotaxane S11d HSQC NMR ($CDCl_3$, 600 MHz, 300 K)

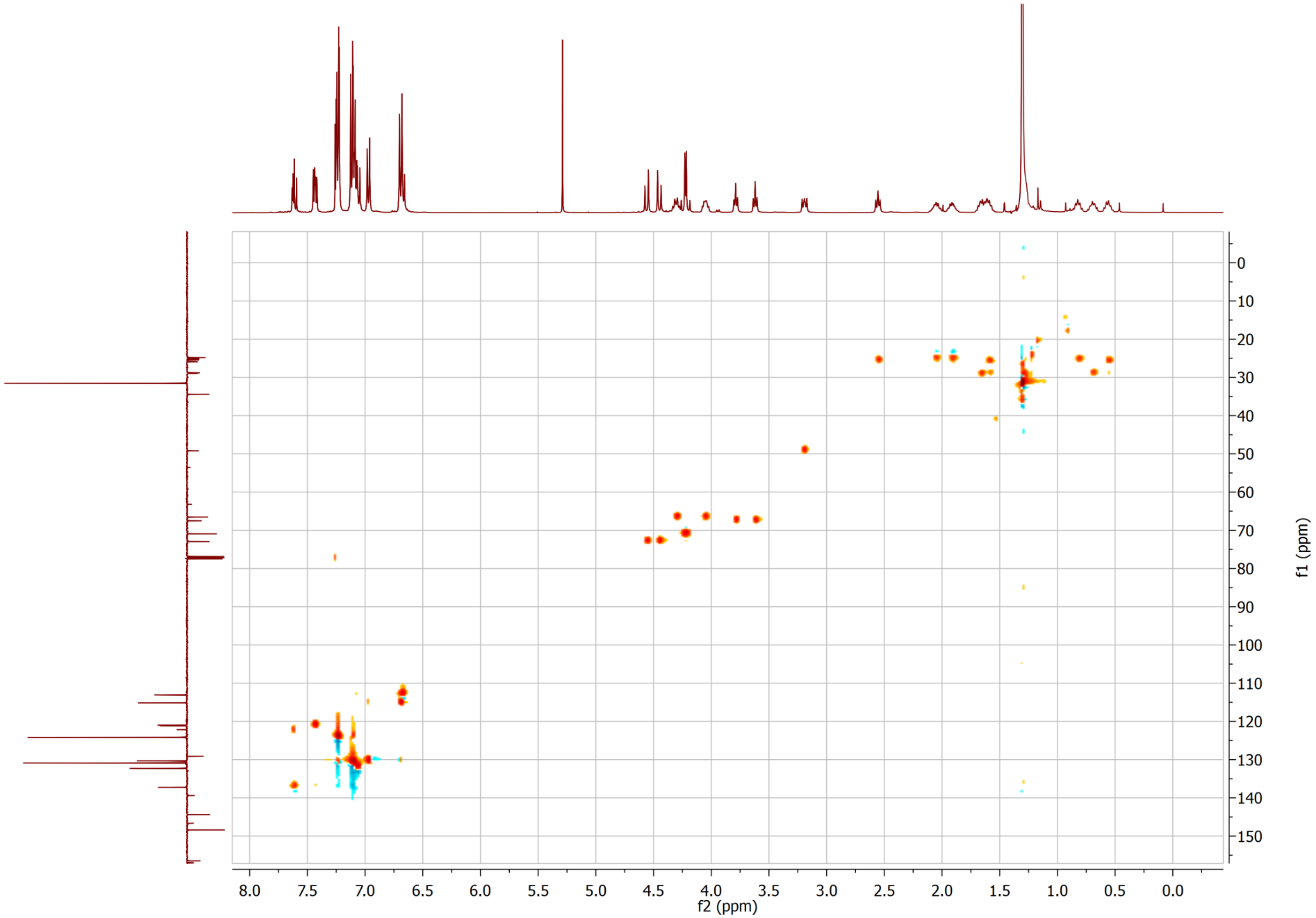

[2]Rotaxane S11d HMBC NMR ($CDCl_3$, 600 MHz, 300 K)

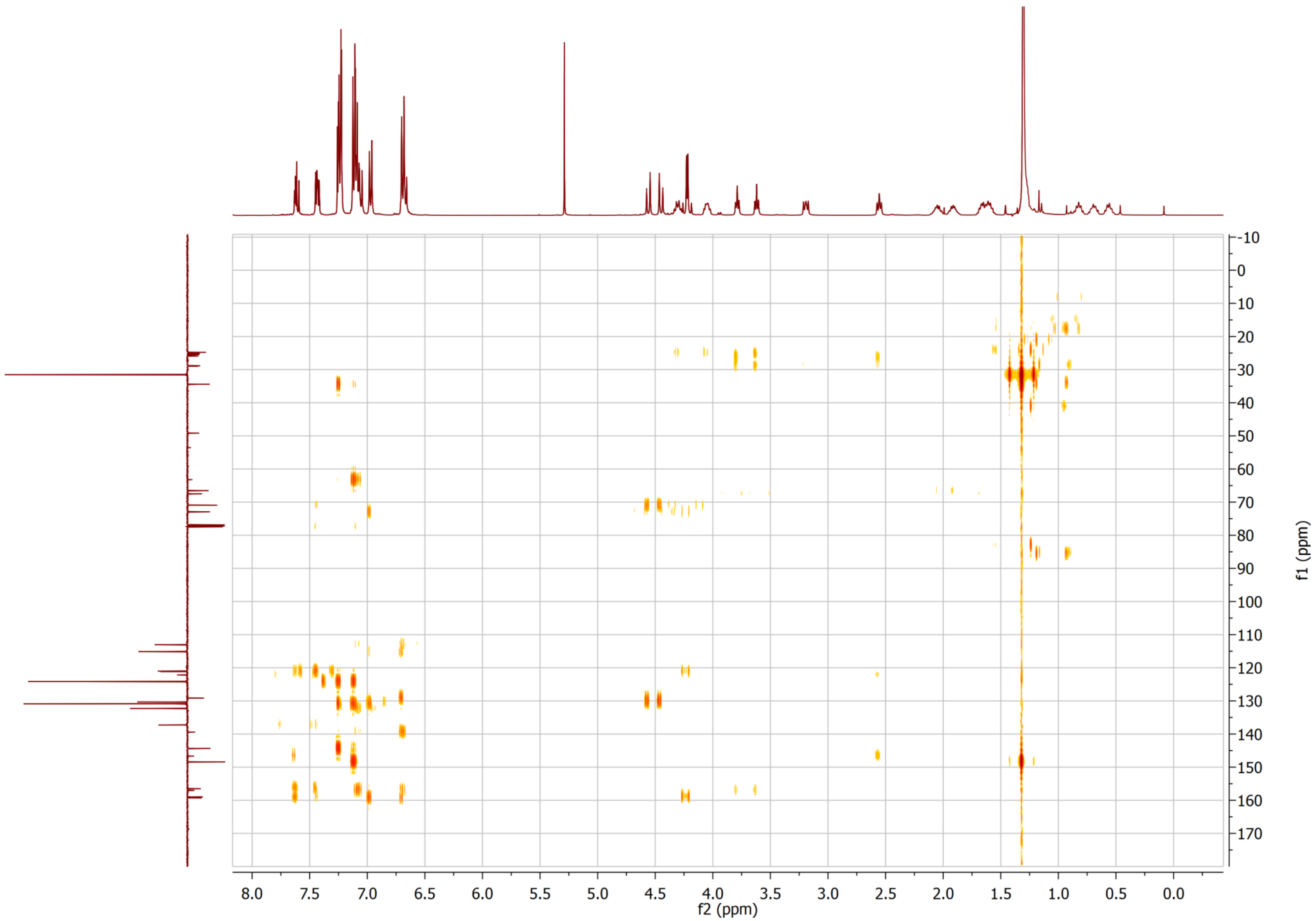

[3]Rotaxane S12 ^{1}H NMR ($CDCl_3$, 400 MHz, 300 K)

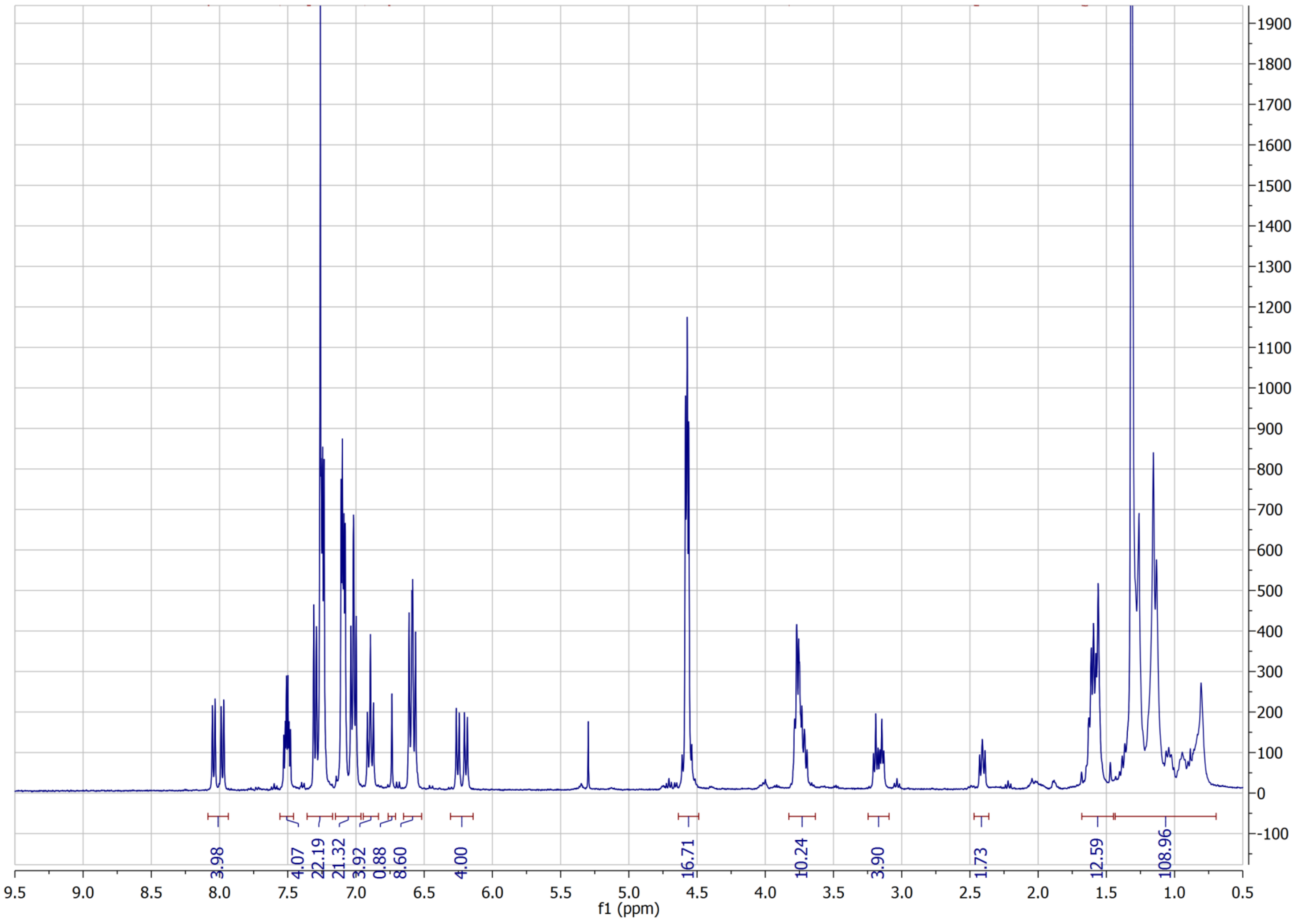

[3]Rotaxane S12 DEPTQ135 NMR ($CDCl_3$, 150 MHz, 300 K)

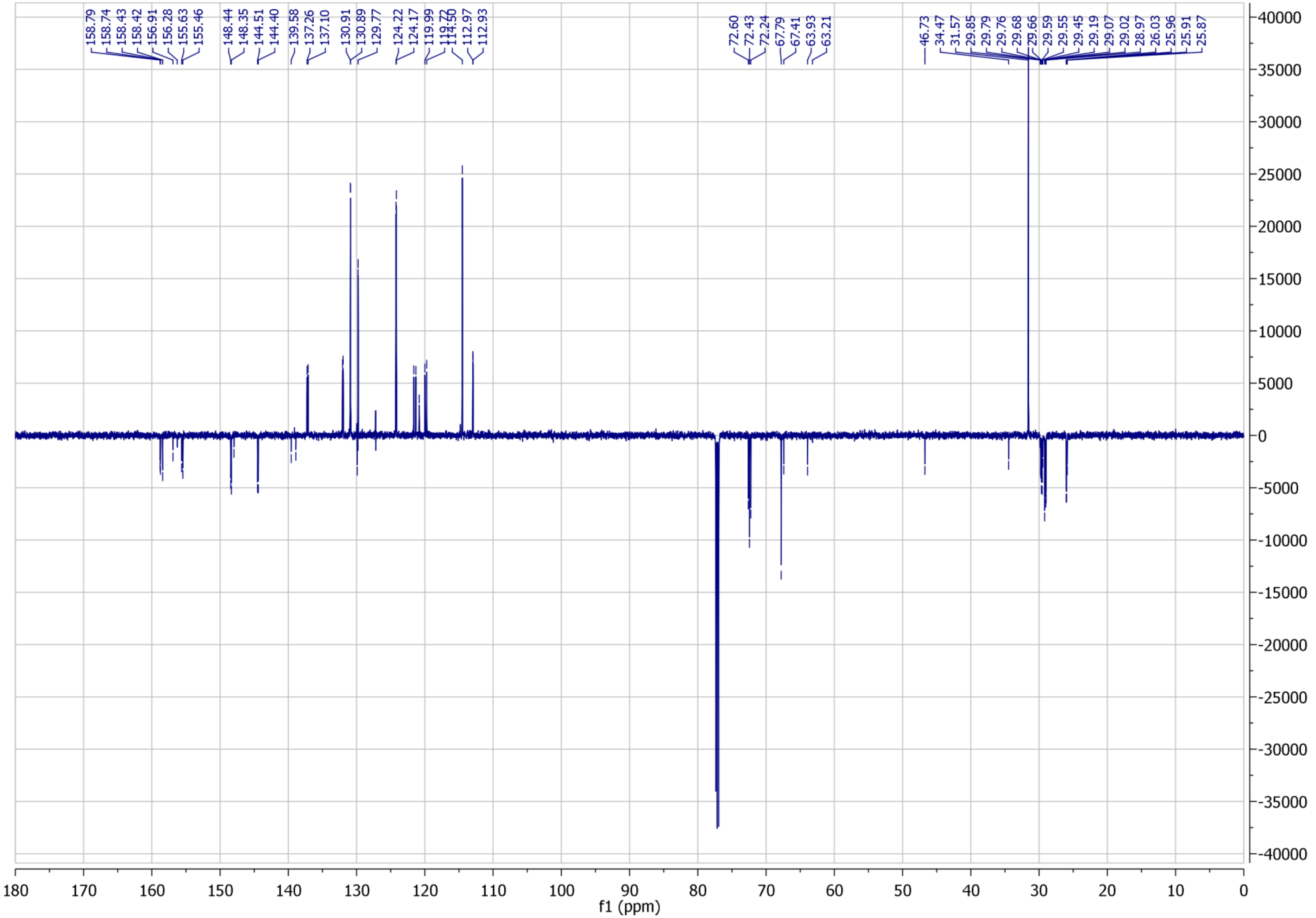

[3]Rotaxane S12 COSY ($CDCl_3$, 400 MHz, 300 K)

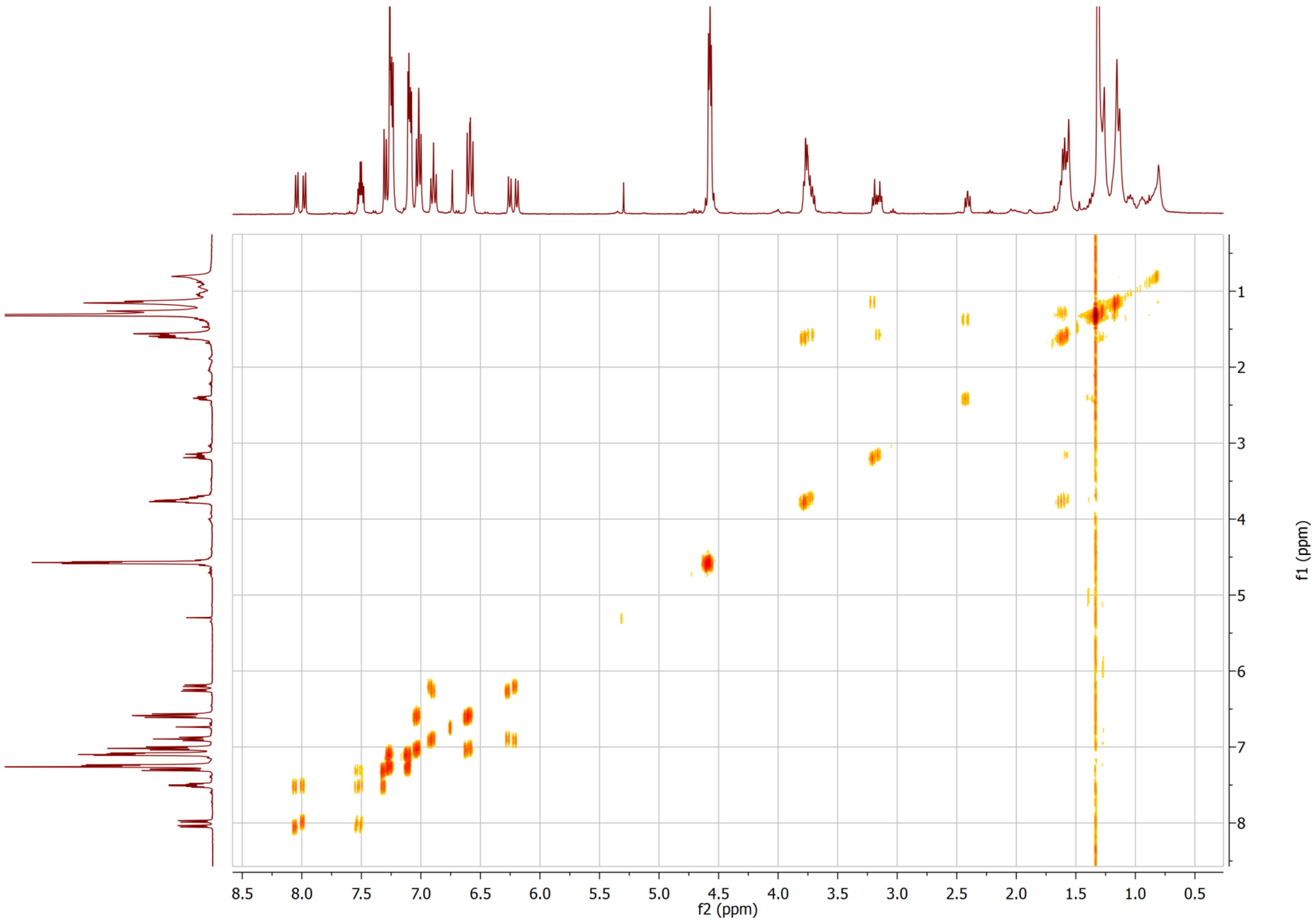

[3]Rotaxane S12 HSQC NMR ($CDCl_3$, 400 MHz, 300 K)

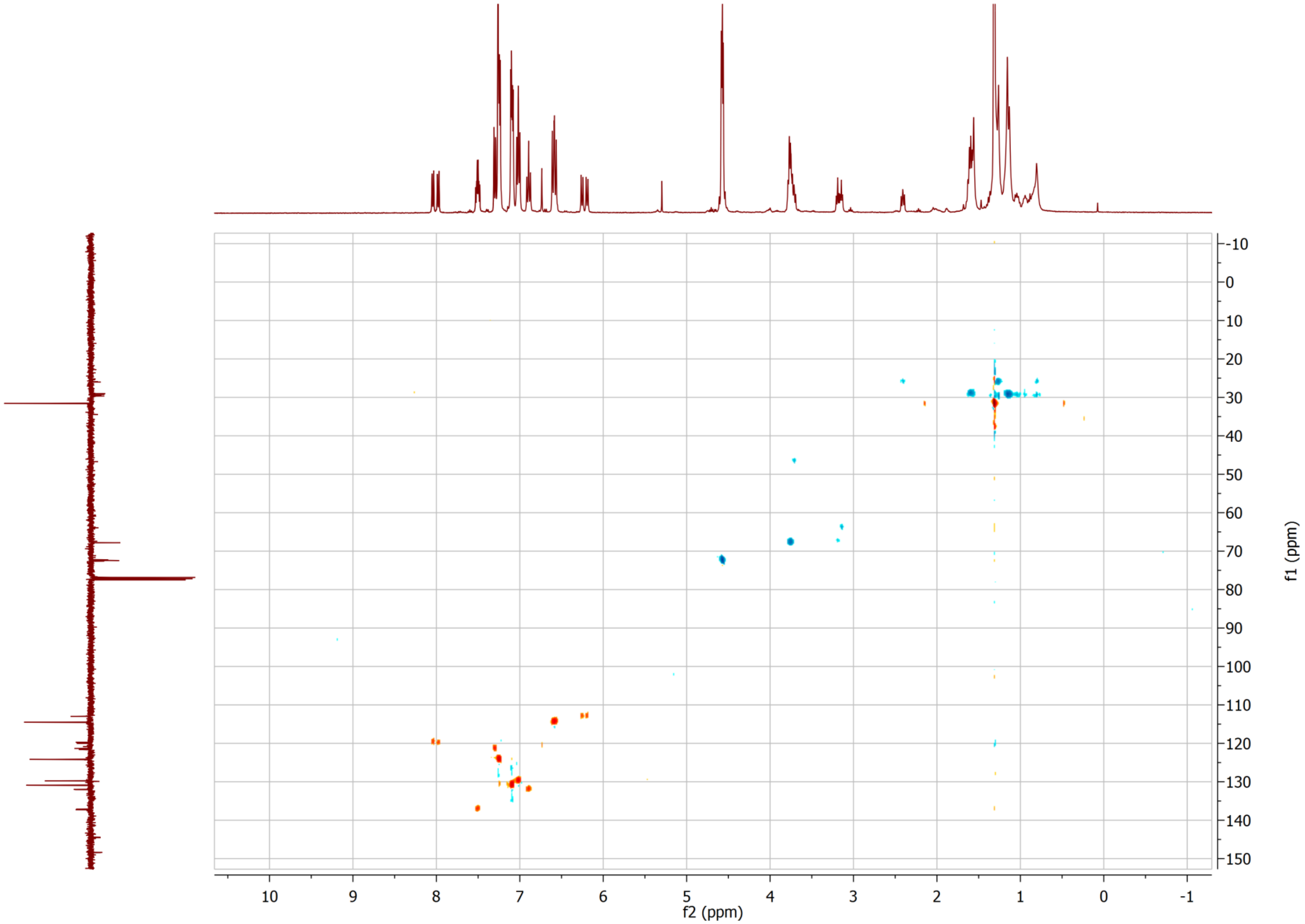

[3]Rotaxane S12 HMBC NMR ($CDCl_3$, 400 MHz, 300 K)

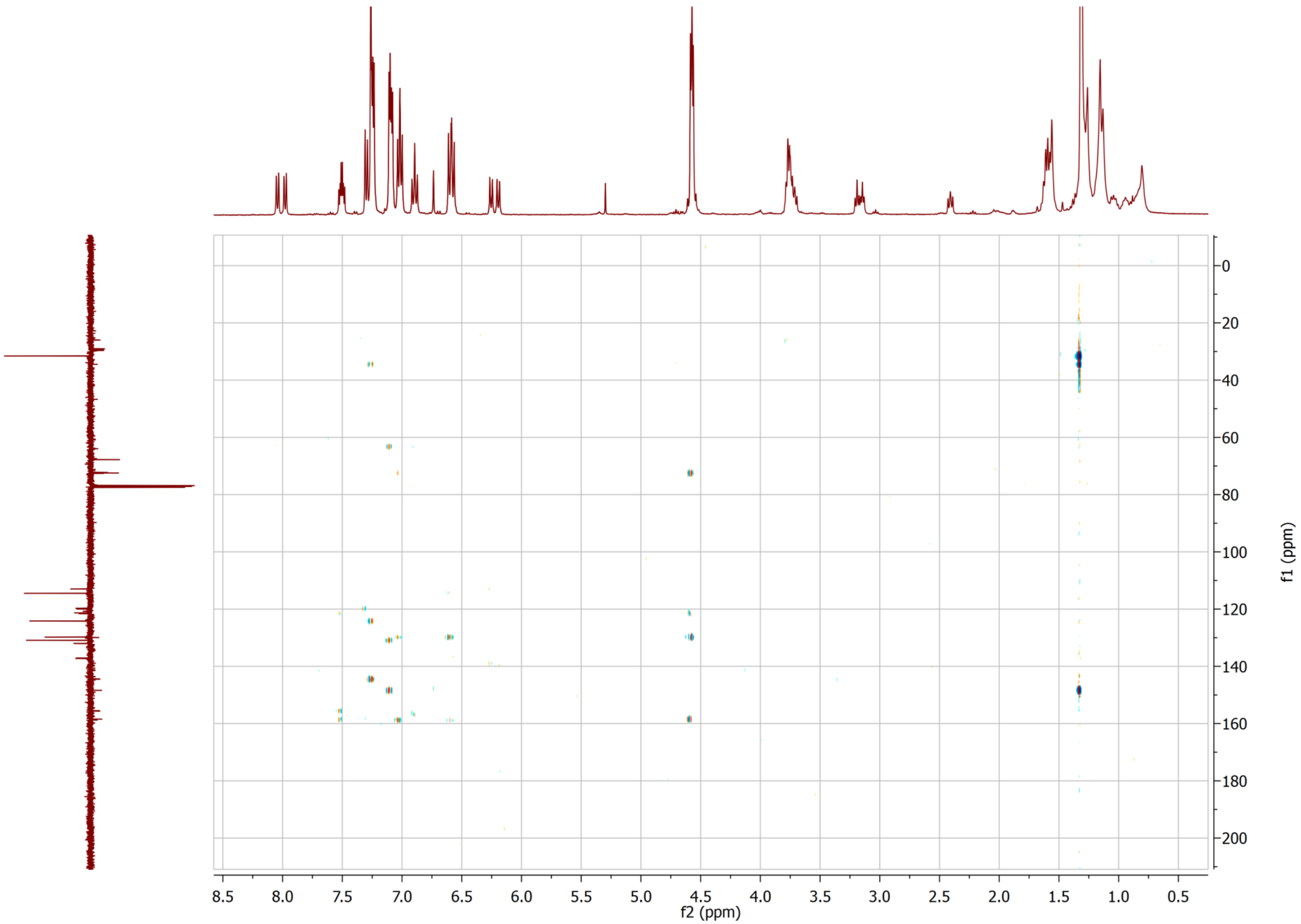

[2]Rotaxane S13 ^{1}H NMR (CDCl$_3$, 400 MHz, 300 K)

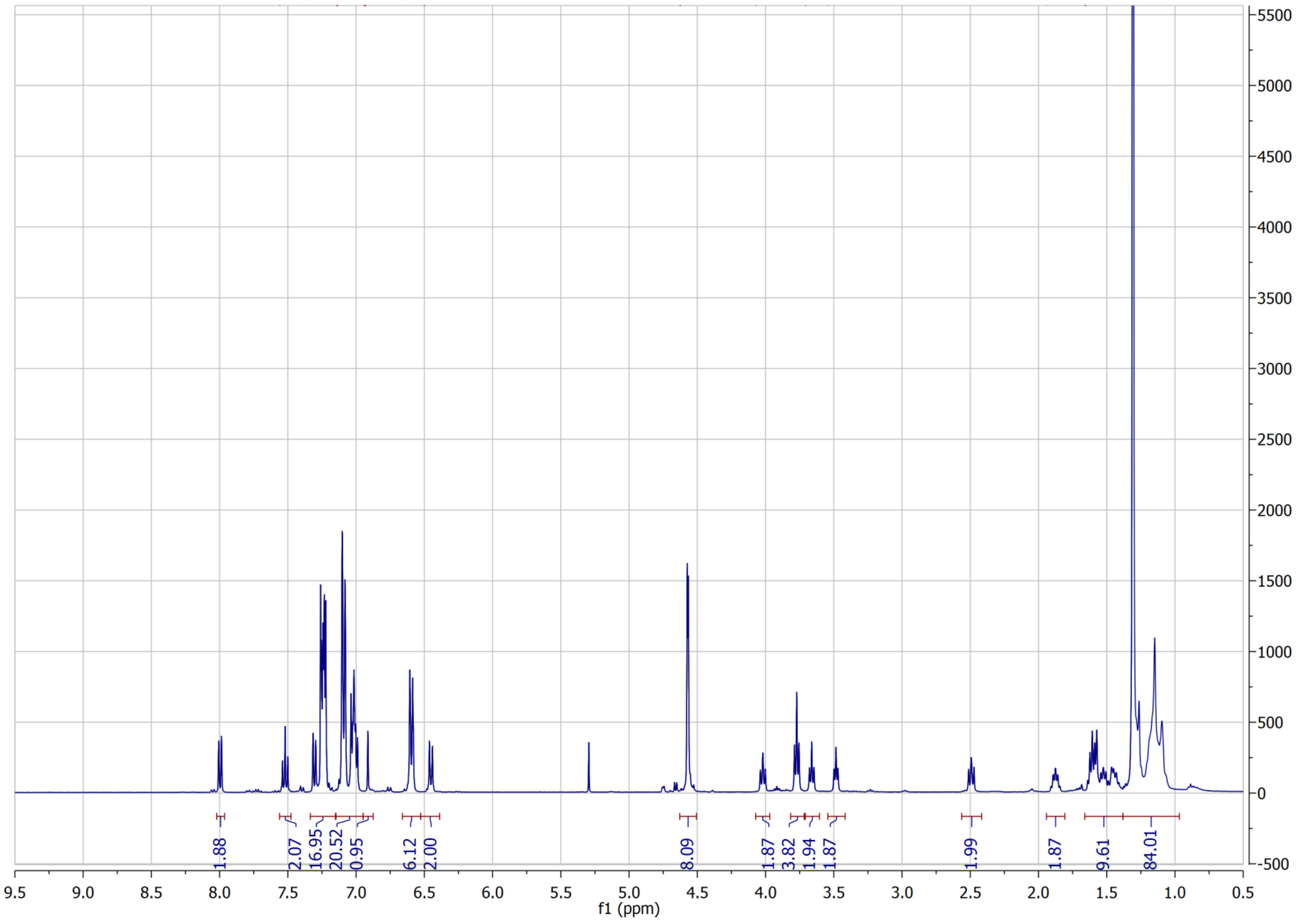

[2]Rotaxane S13 DEPTQ135 NMR ($CDCl_3$, 100 MHz, 300 K)

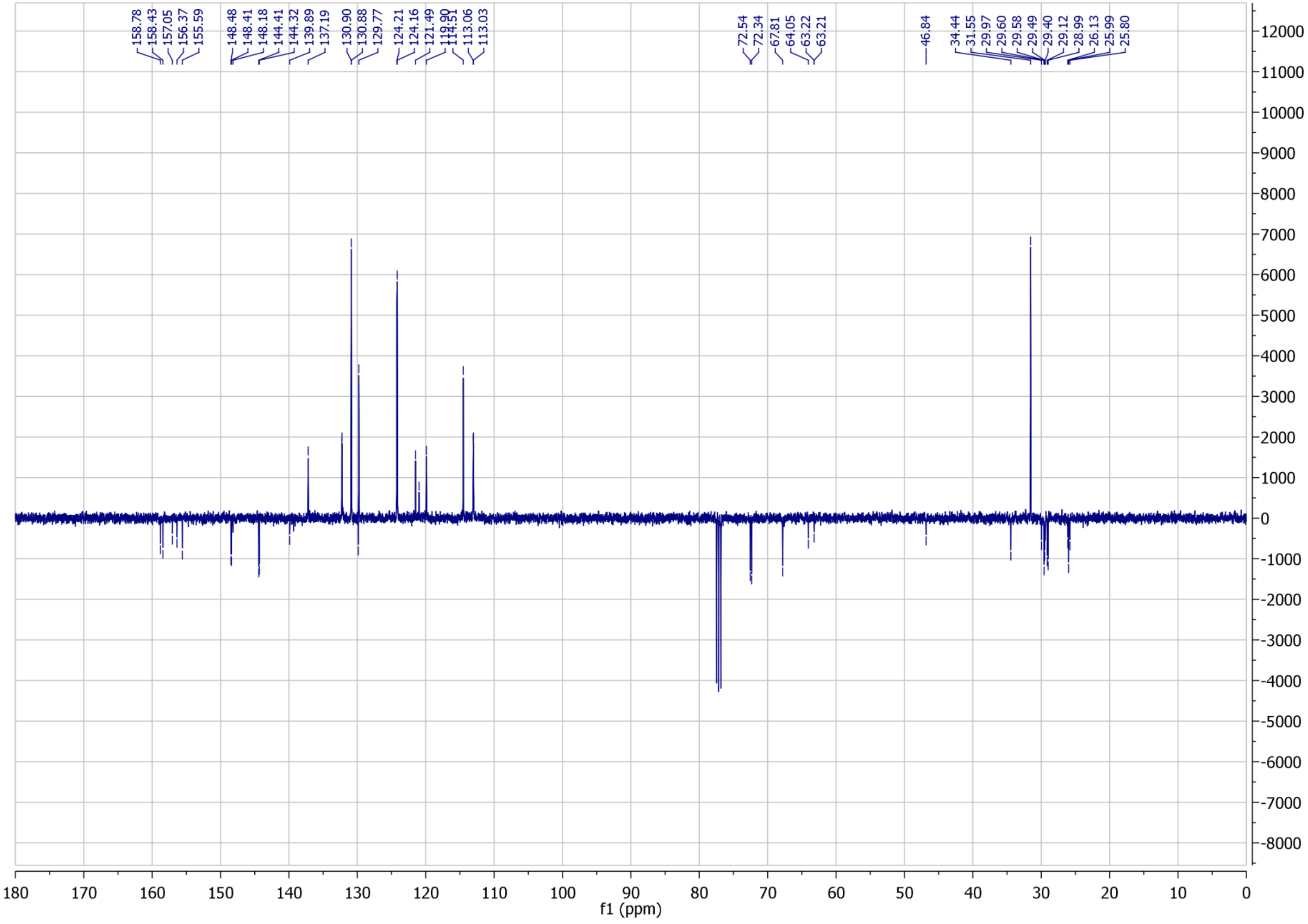

[2]Rotaxane S13 COSY ($CDCl_3$, 400 MHz, 300 K)

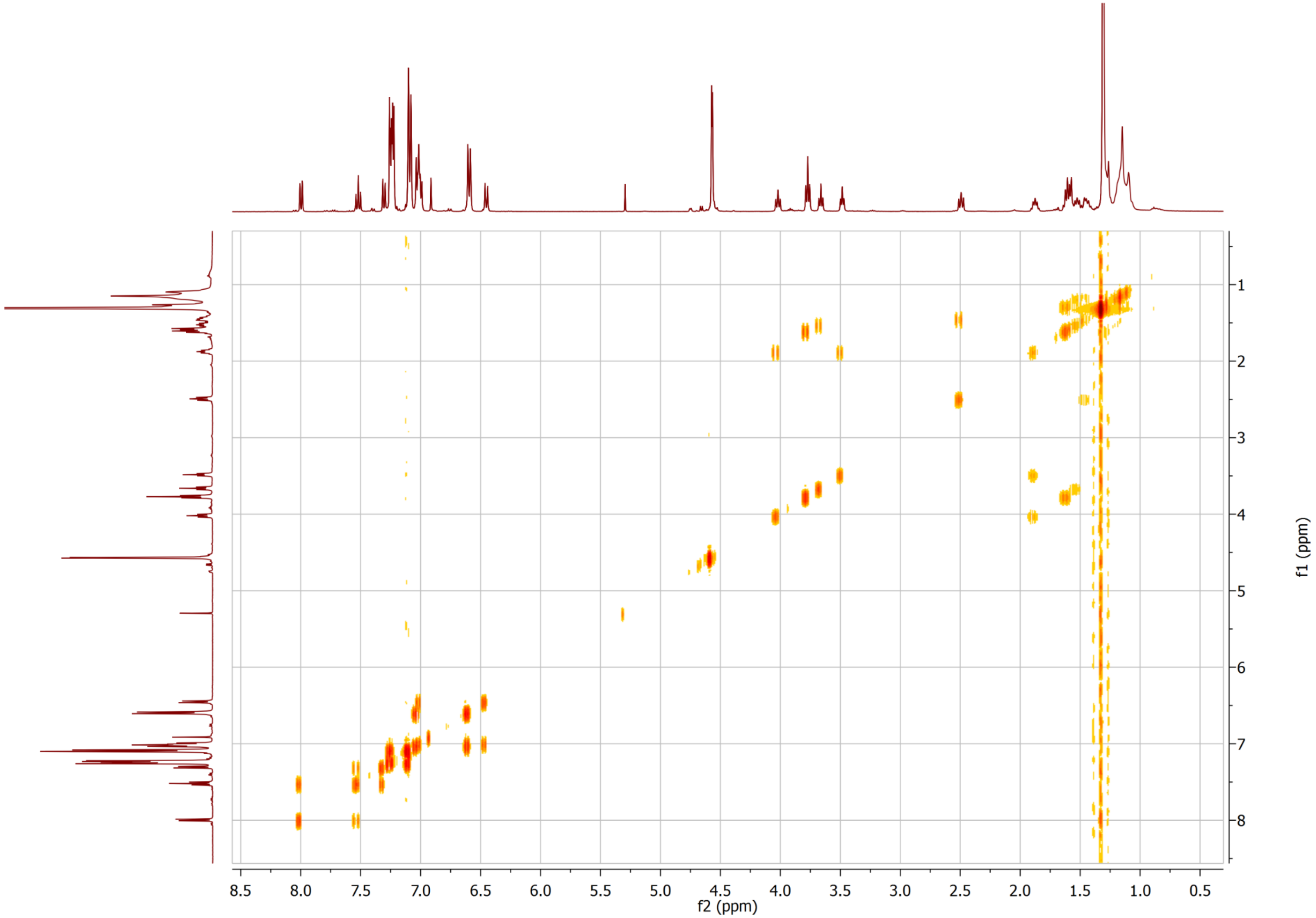

[2]Rotaxane S13 HSQC NMR ($CDCl_3$, 400 MHz, 300 K)

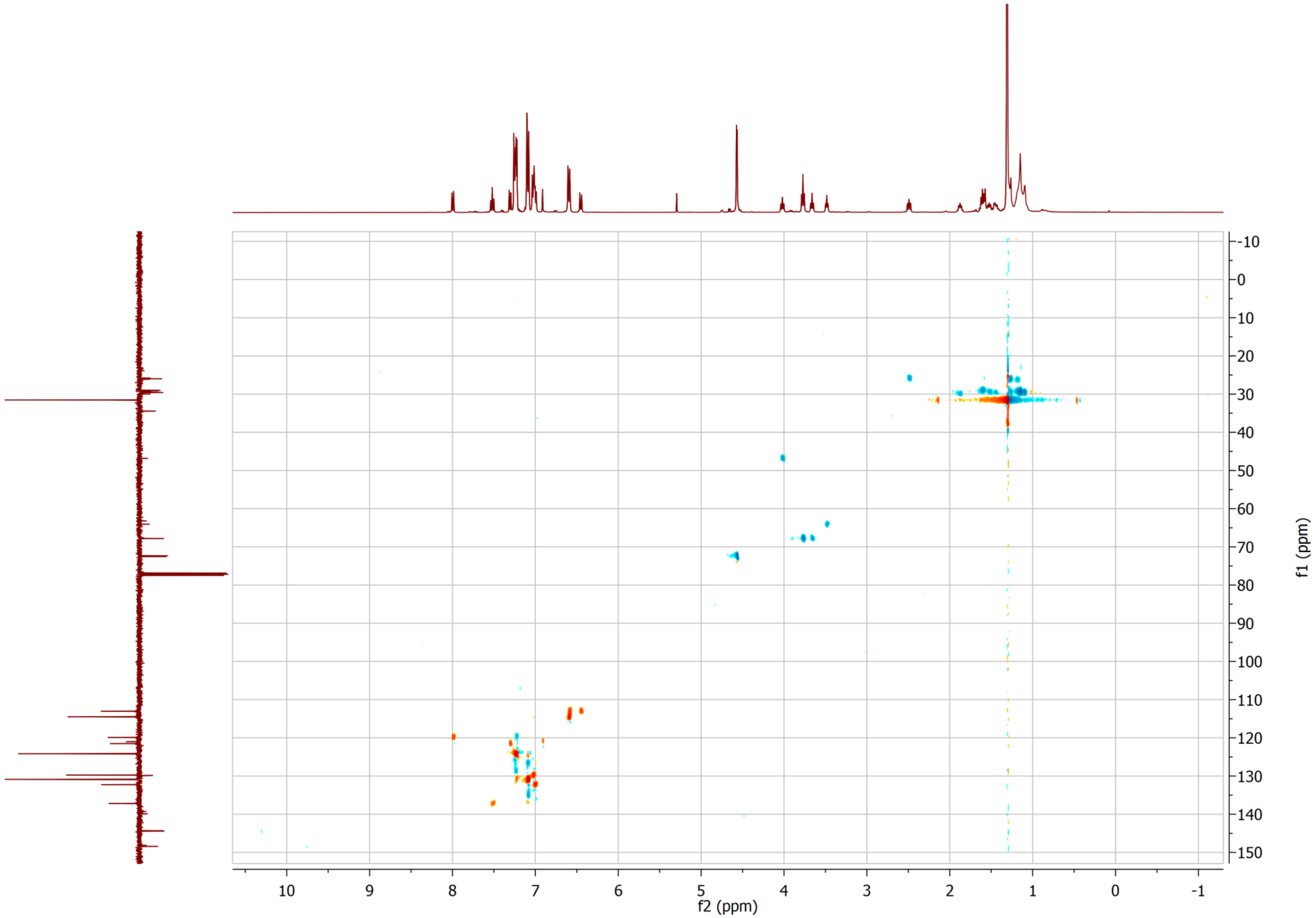

[2]Rotaxane S13 HMBC NMR ($CDCl_3$, 400 MHz, 300 K)

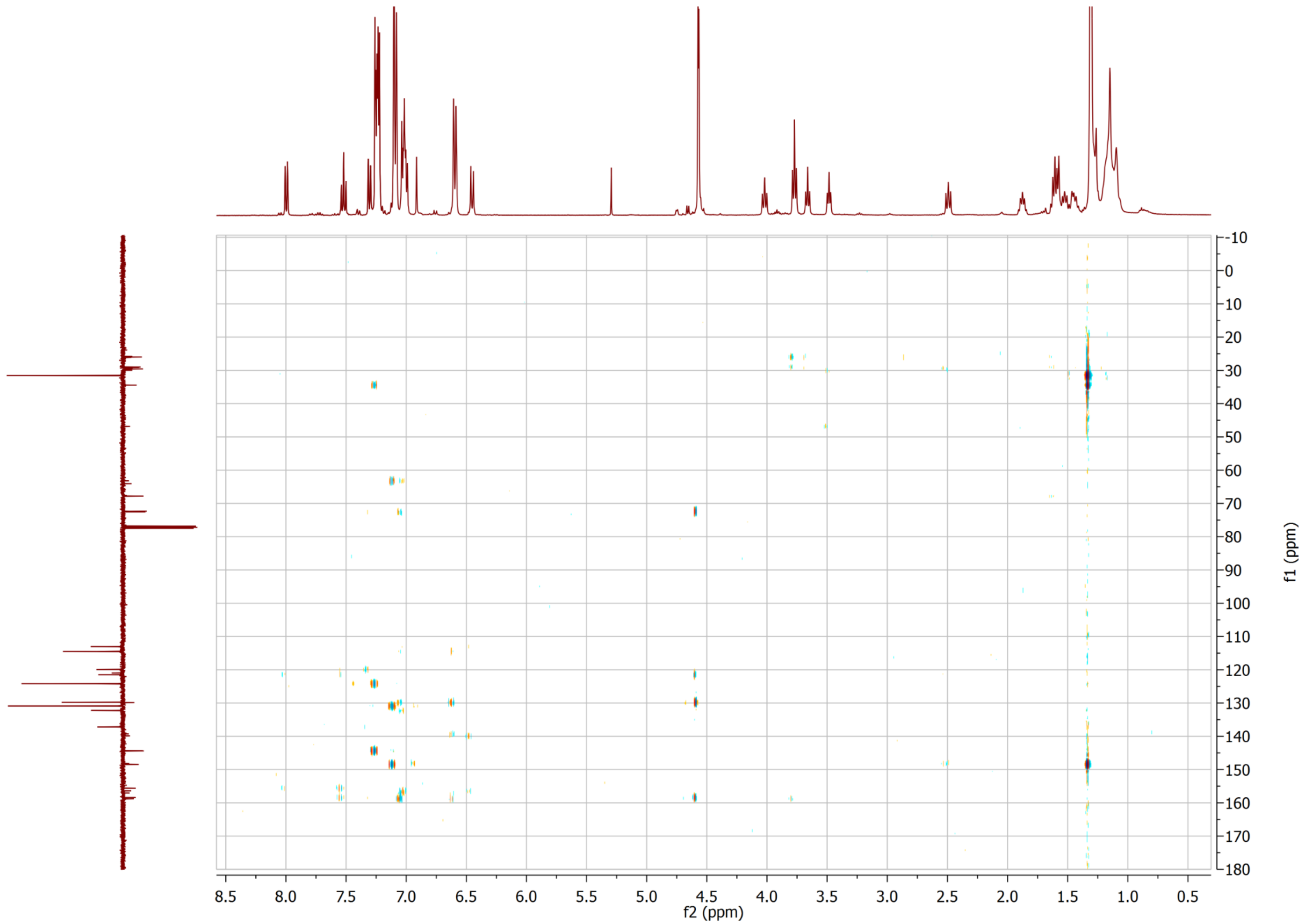

Thread S14 ^{1}H NMR ($CDCl_3$, 400 MHz, 300 K)

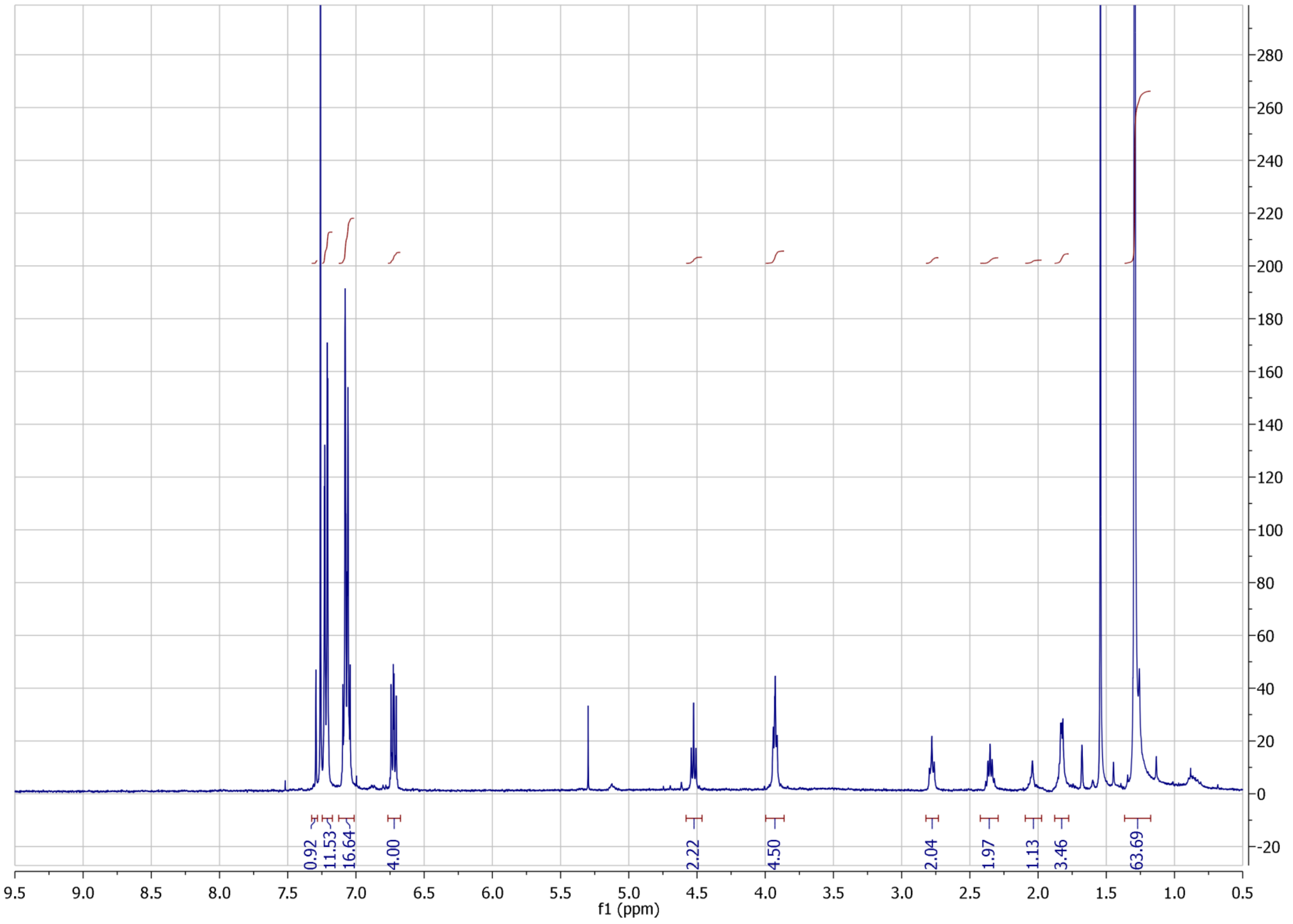

Thread S14 DEPTQ135 NMR ($CDCl_3$, 150 MHz, 300 K)

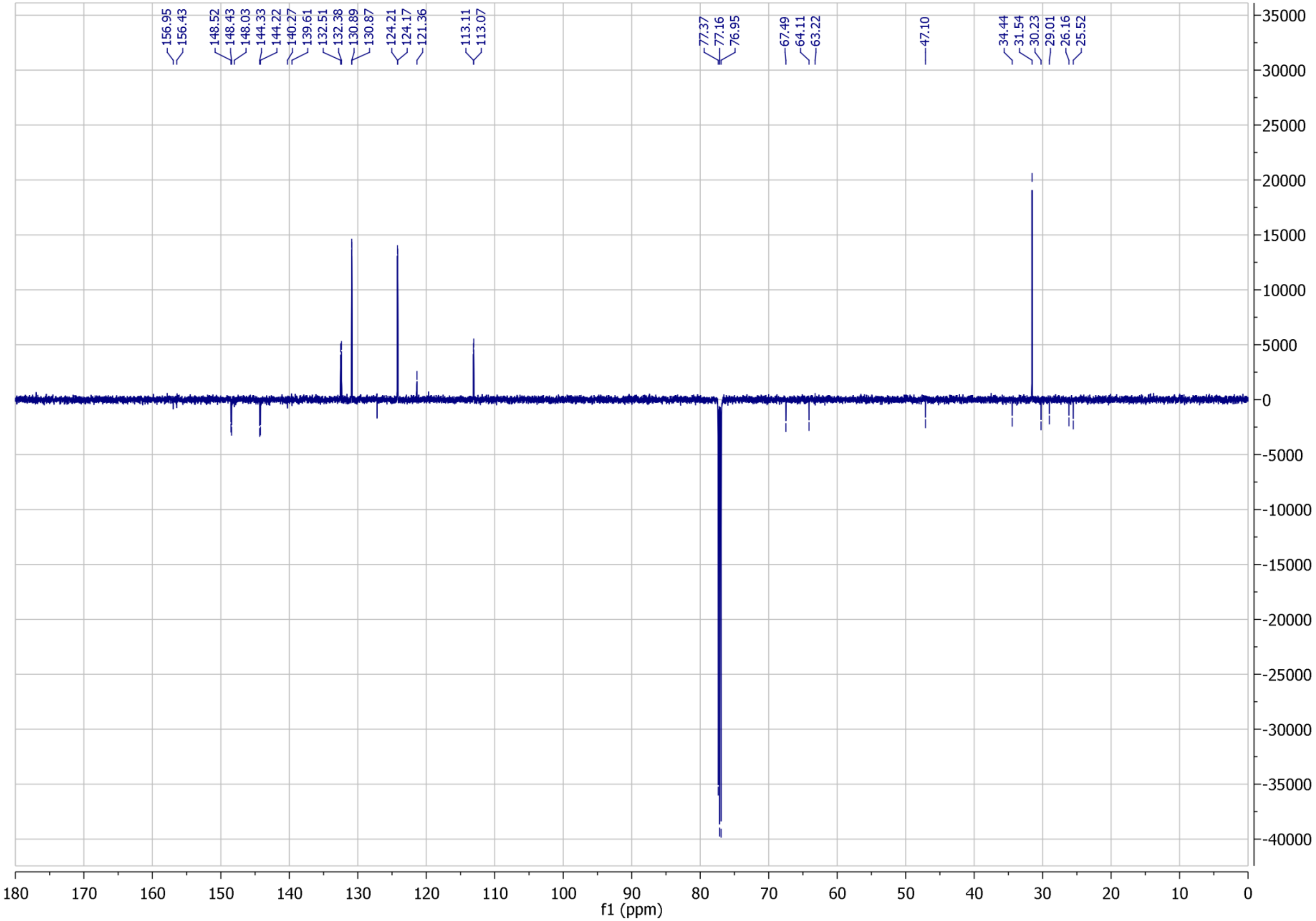

Thread S15 ^{1}H NMR ($CDCl_3$, 400 MHz, 300 K)

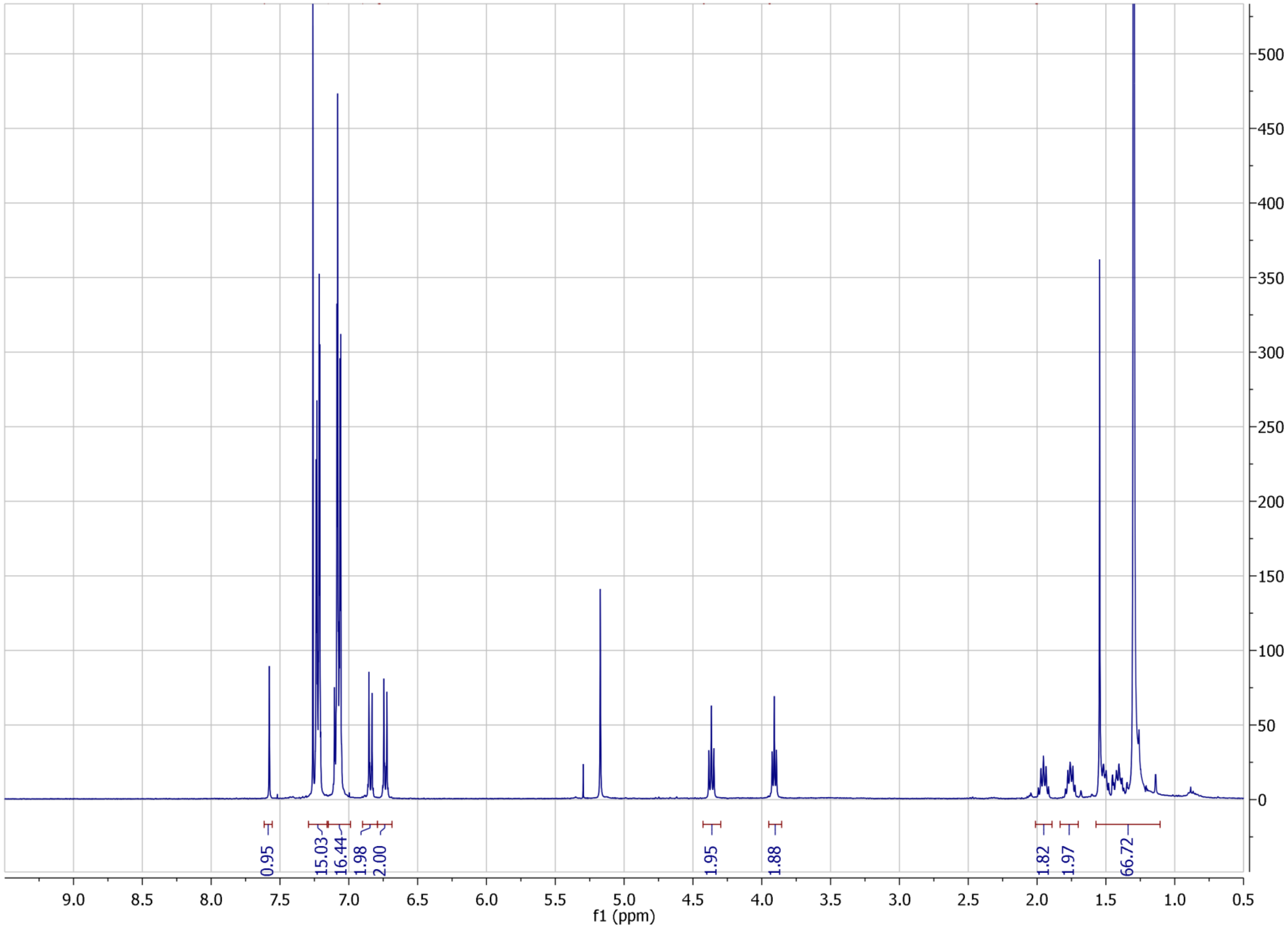

Thread S15 DEPTQ135 NMR ($CDCl_3$, 150 MHz, 300 K)

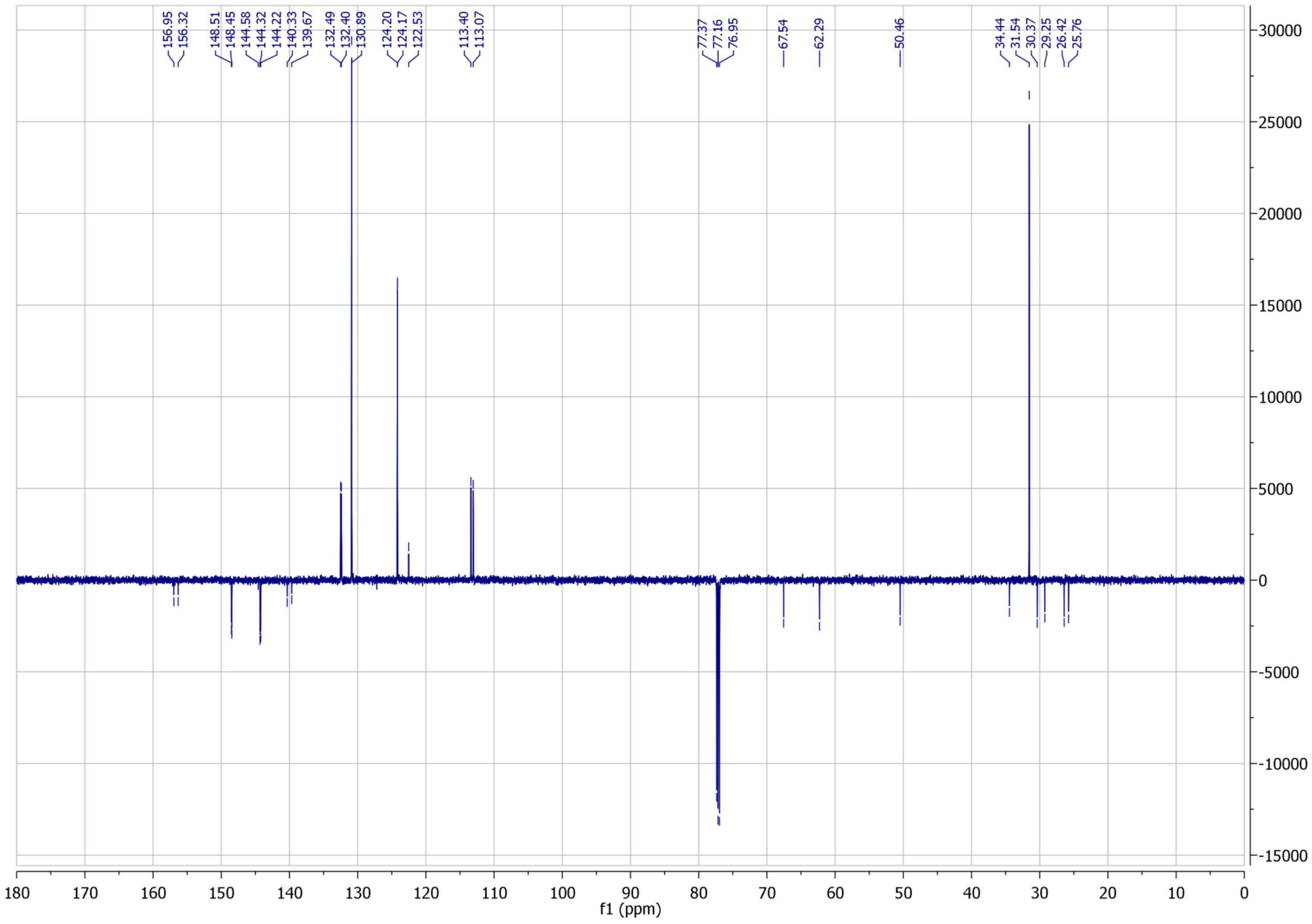

Thread S16 ^{1}H NMR ($CDCl_3$, 400 MHz, 300 K)

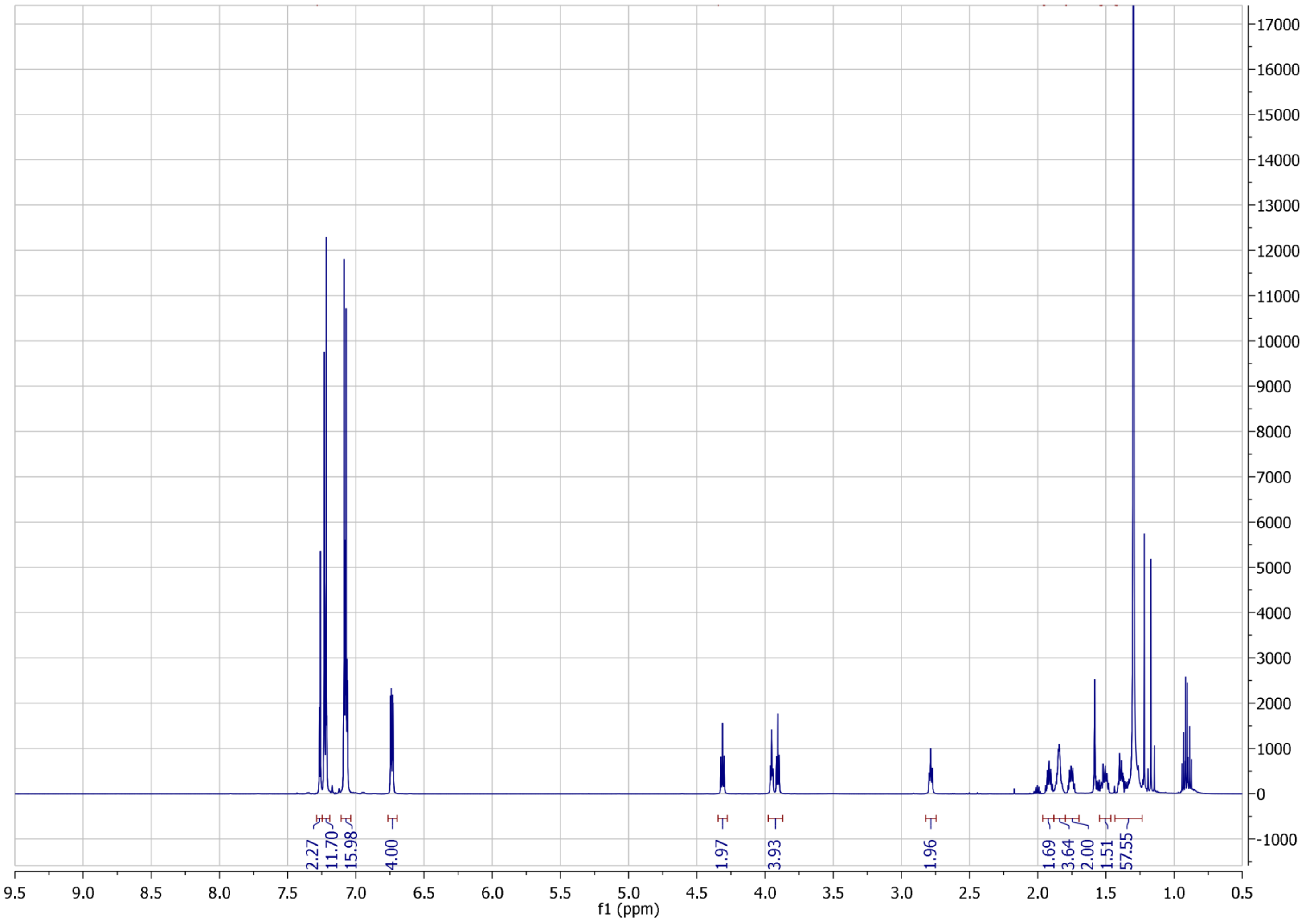

Thread S16 DEPTQ135 NMR ($CDCl_3$, 100 MHz, 300 K)

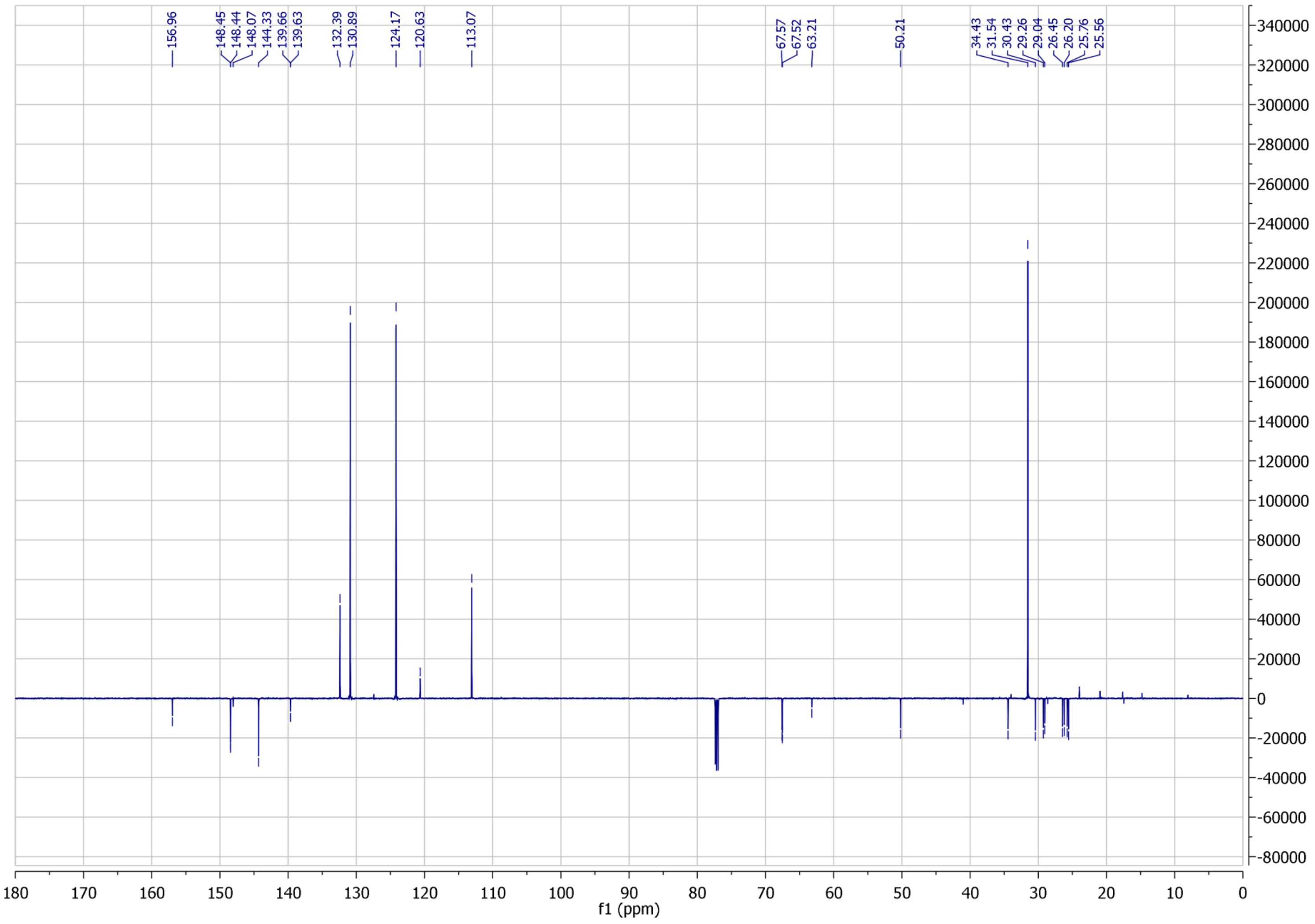

Thread S17 ^{1}H NMR ($CDCl_3$, 400 MHz, 300 K)

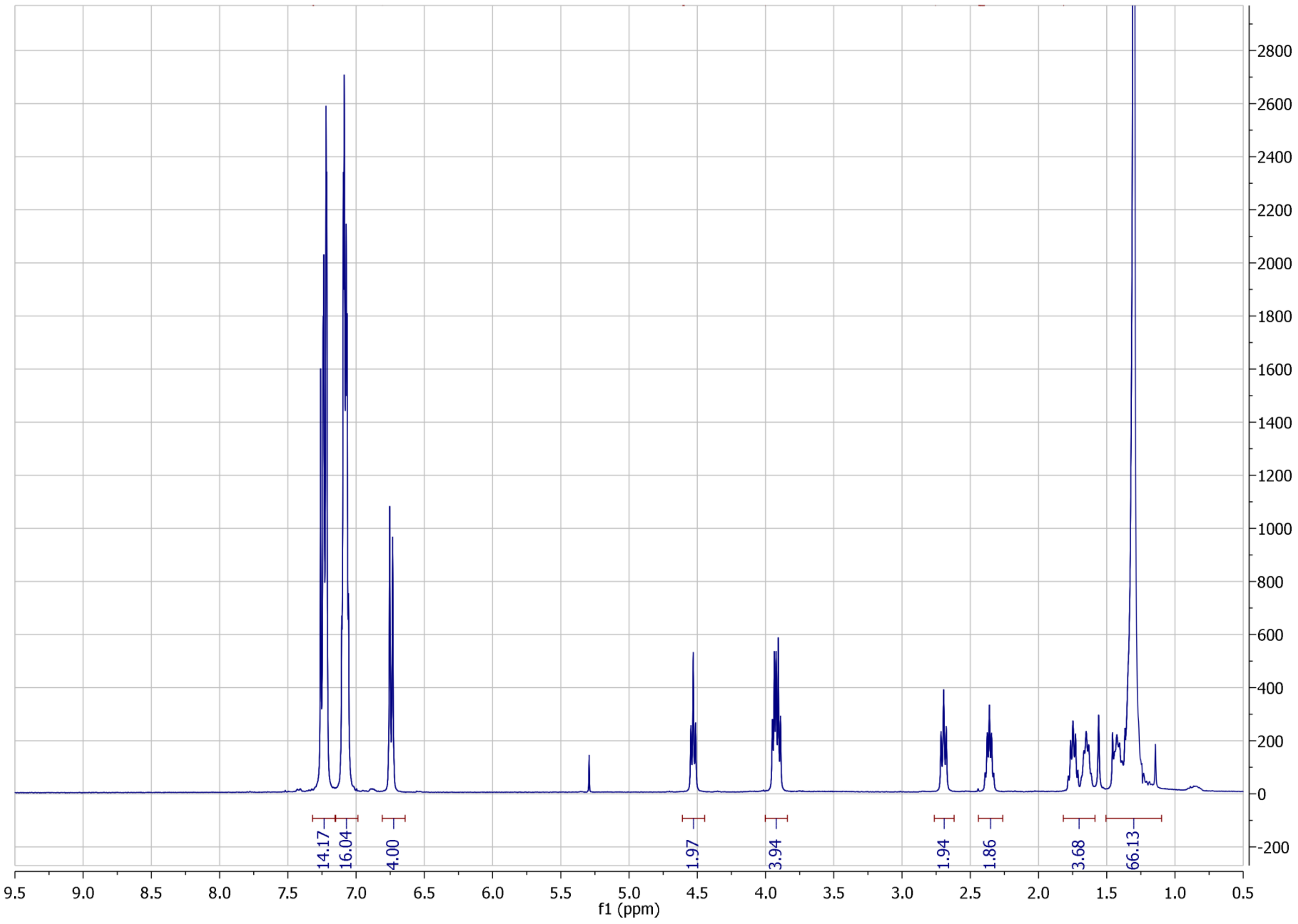

Thread S17 DEPTQ135 NMR ($CDCl_3$, 100 MHz, 300 K)

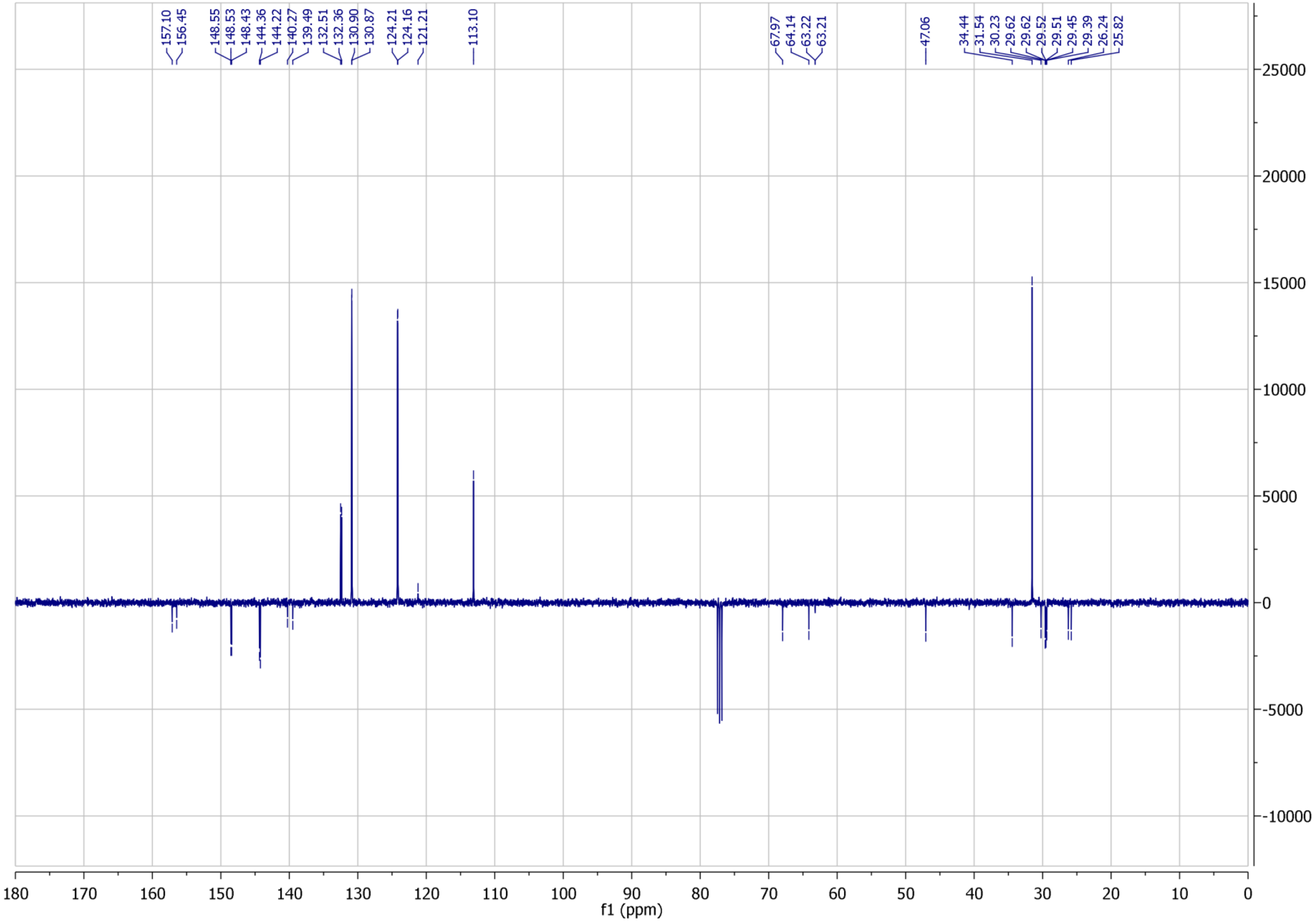

Macrocycle 9a ^1H NMR ($CDCl_3$, 400 MHz, 300 K)

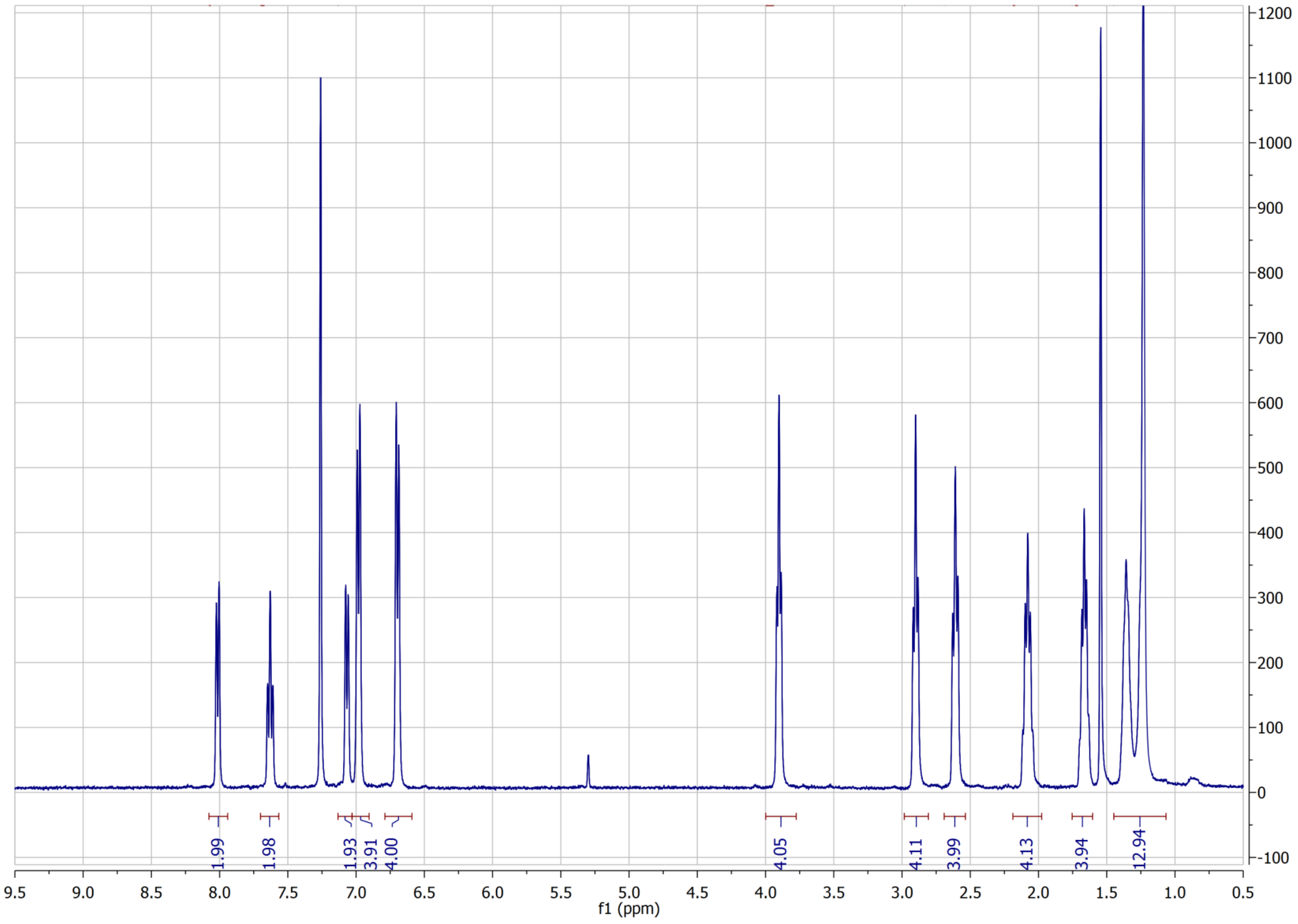

Macrocycle 9a ^{13}C NMR ($CDCl_3$, 100 MHz, 300 K)

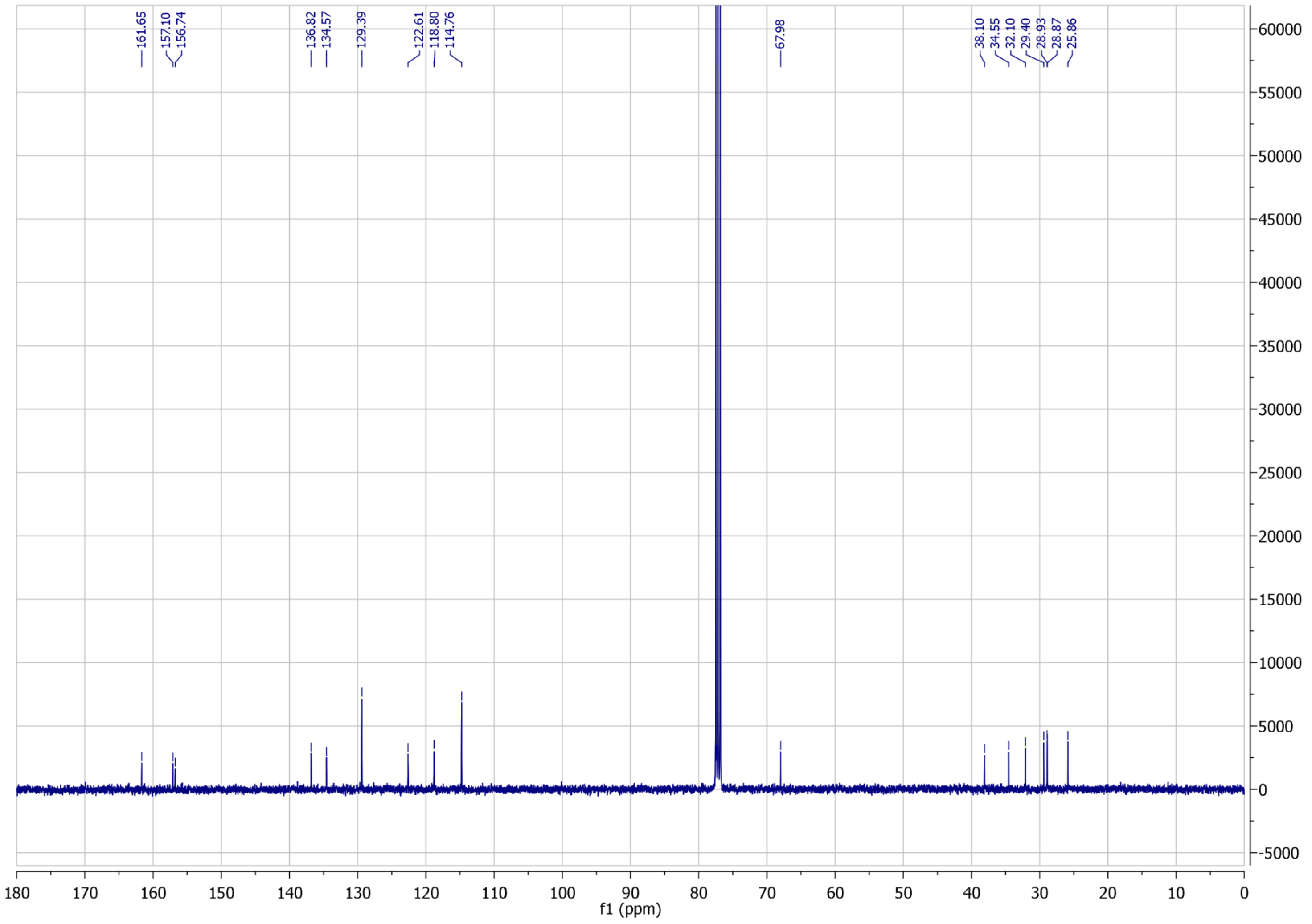

Macrocycle 9a DEPT135 NMR ($CDCl_3$, 100 MHz, 300 K)

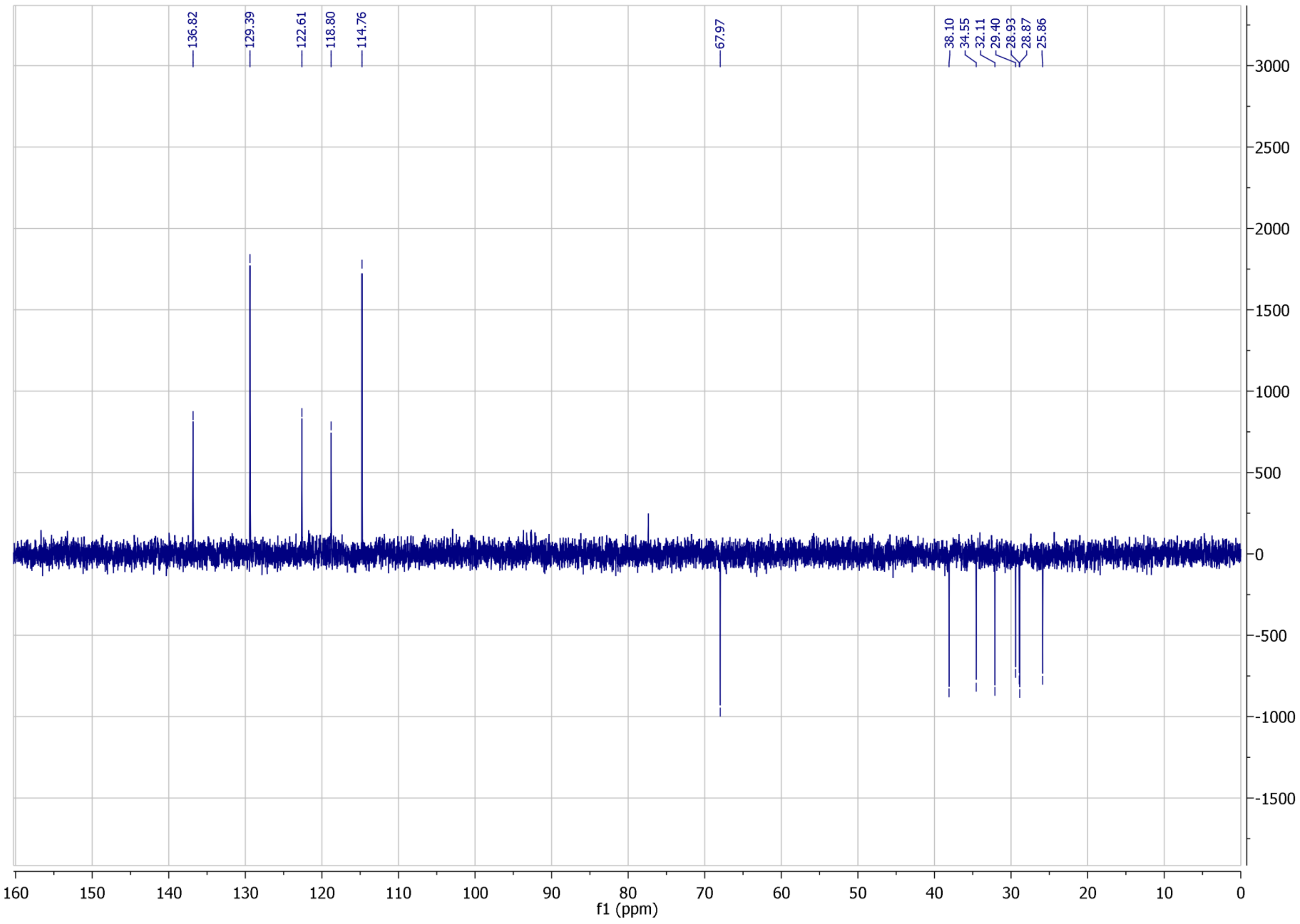

Macrocycle 9a COSY ($CDCl_3$, 400 MHz, 300 K)

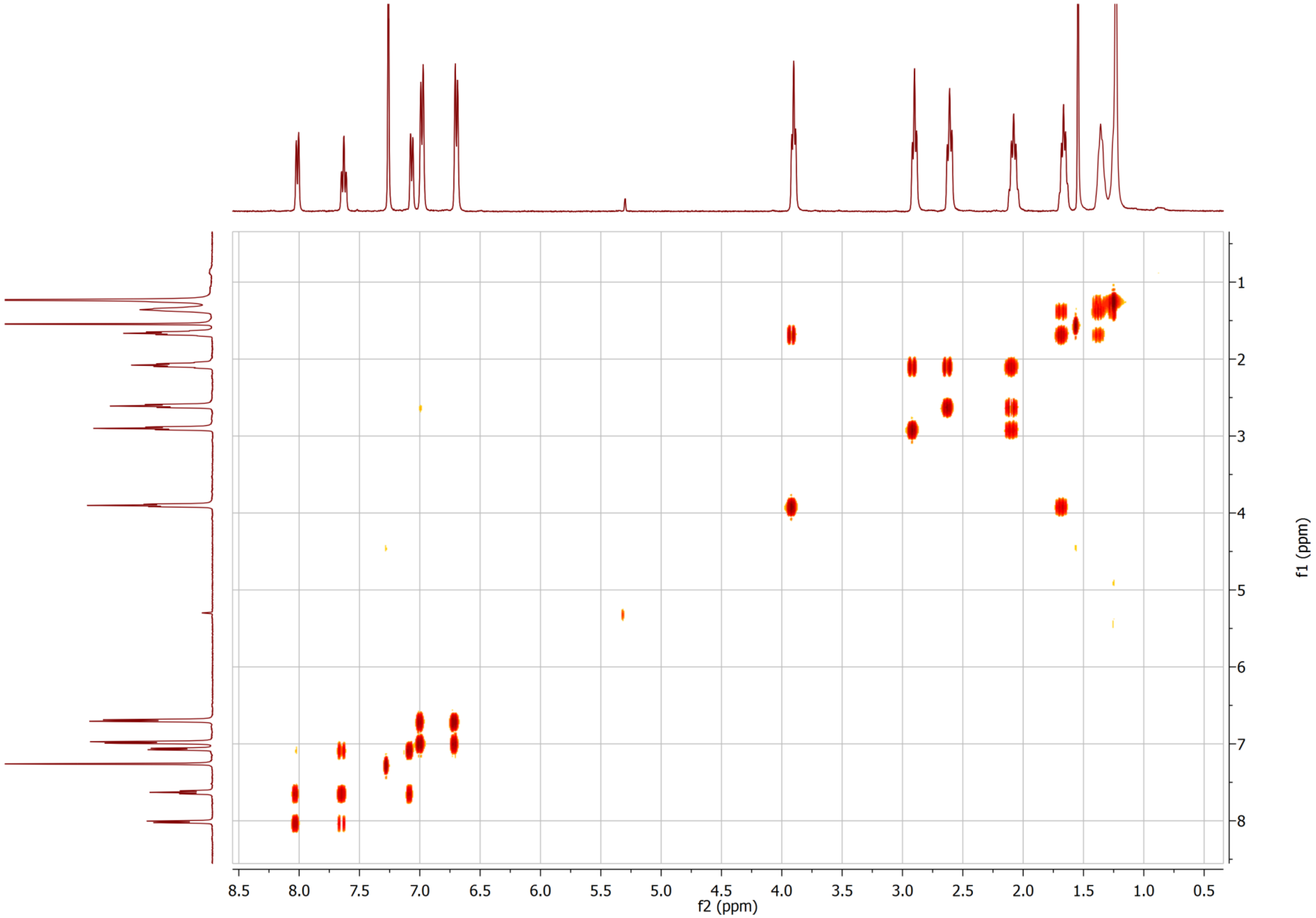

Macrocycle 9a HSQC NMR ($CDCl_3$, 400 MHz, 300 K)

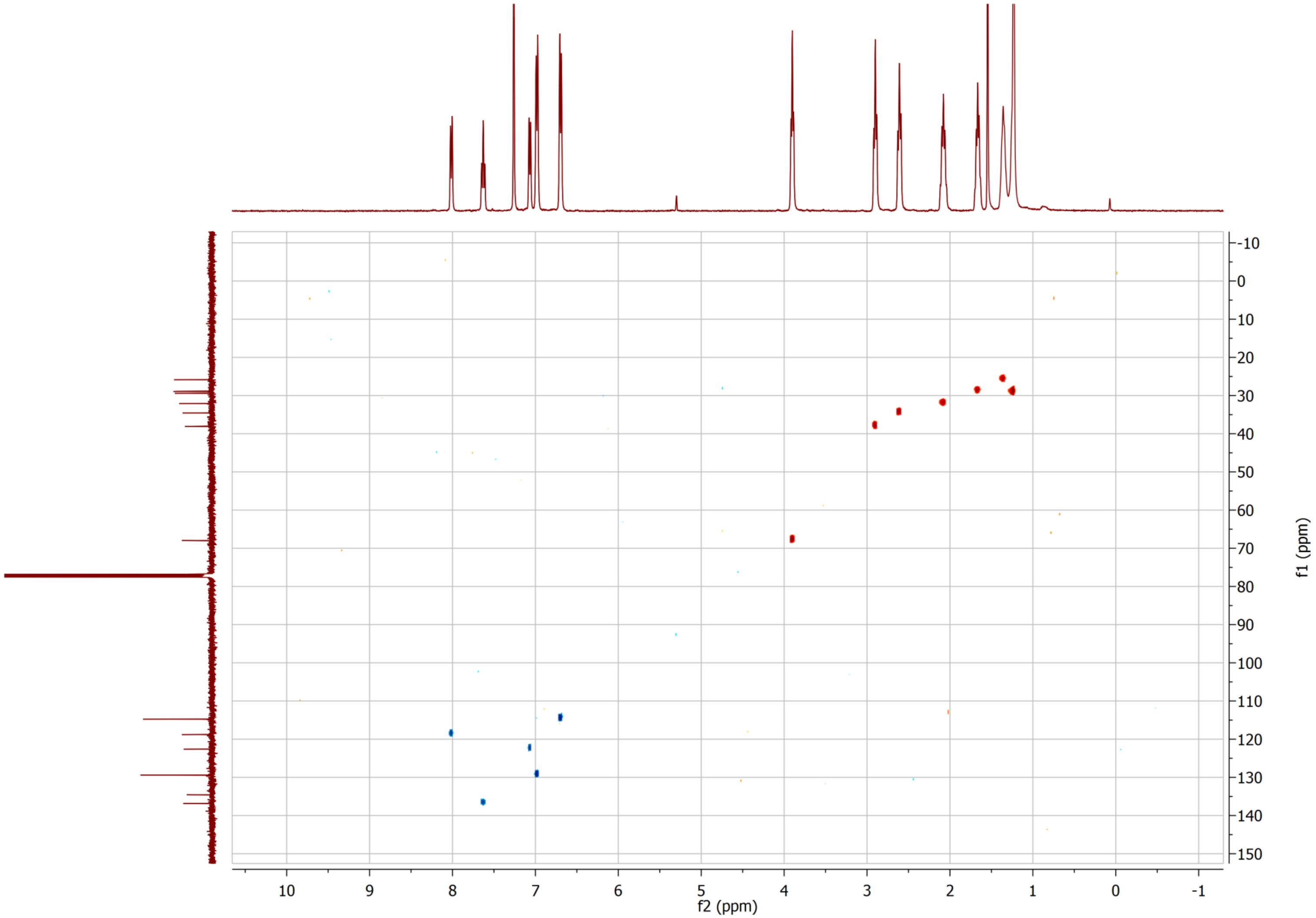

Macrocycle 9a HMBC NMR ($CDCl_3$, 400 MHz, 300 K)

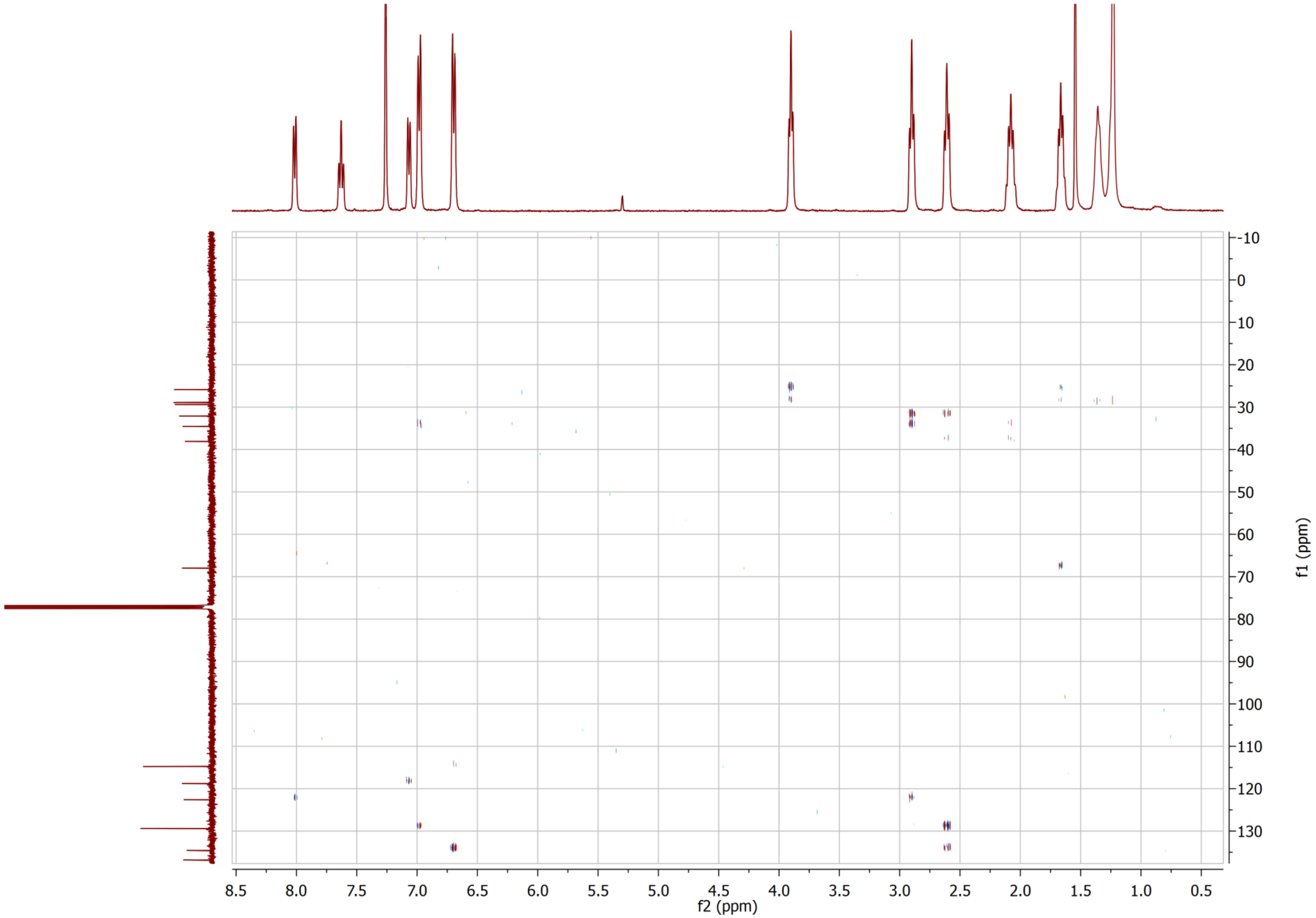

Macrocycle 9b ^{1}H NMR ($CDCl_3$, 400 MHz, 300 K)

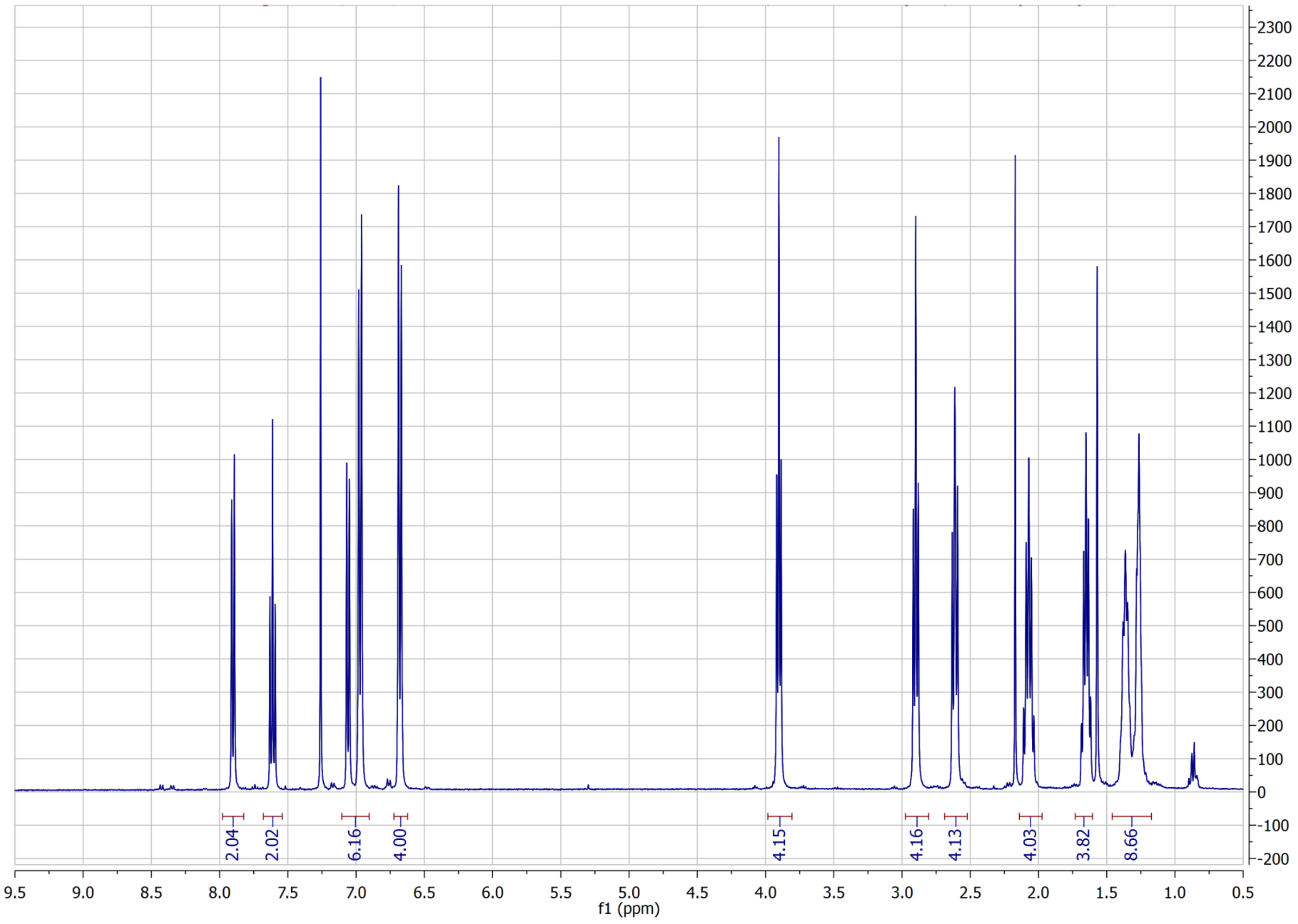

Macrocycle 9b DEPTQ135 NMR ($CDCl_3$, 100 MHz, 300 K)

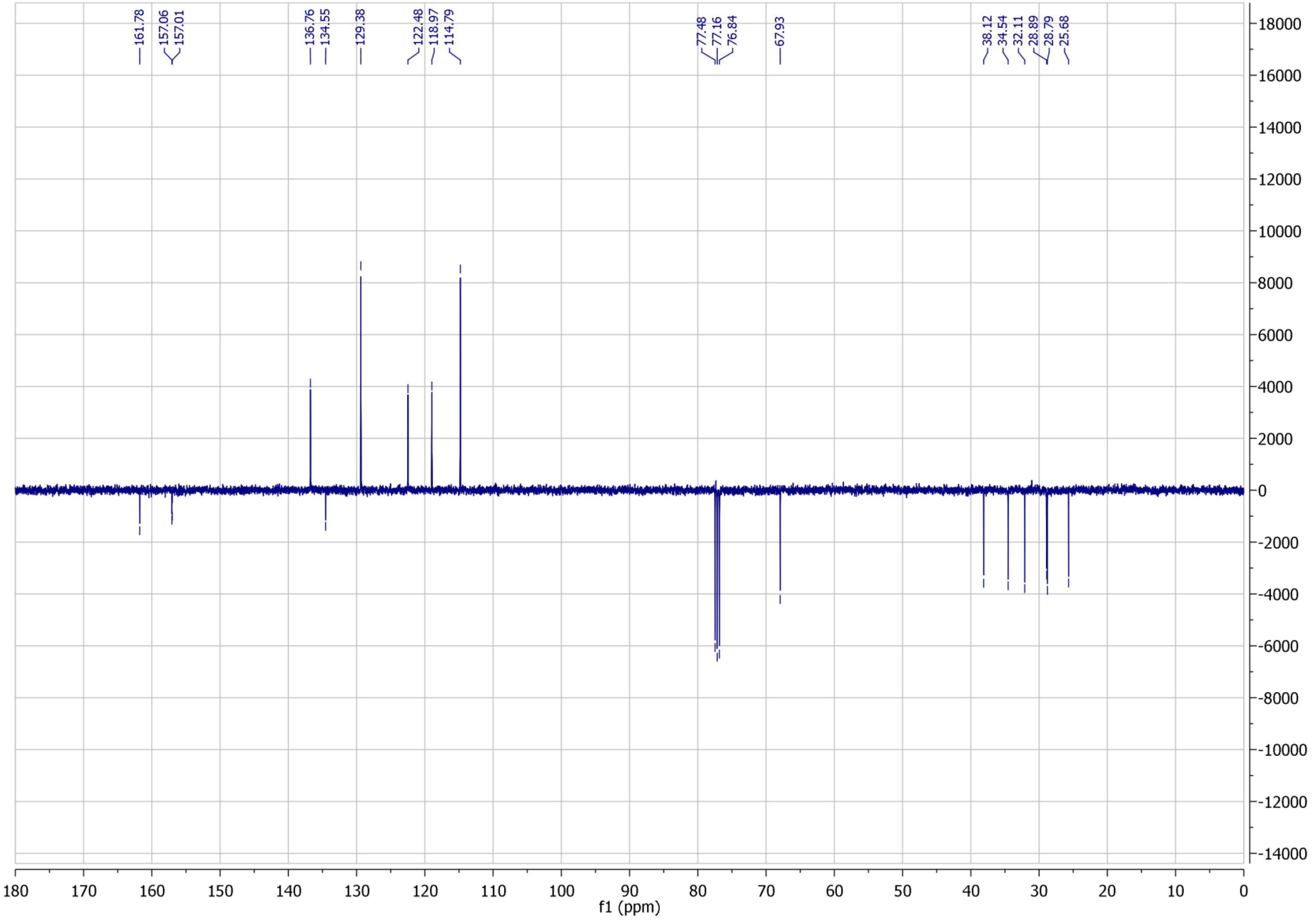

Macrocycle 9b COSY ($CDCl_3$, 400 MHz, 300 K)

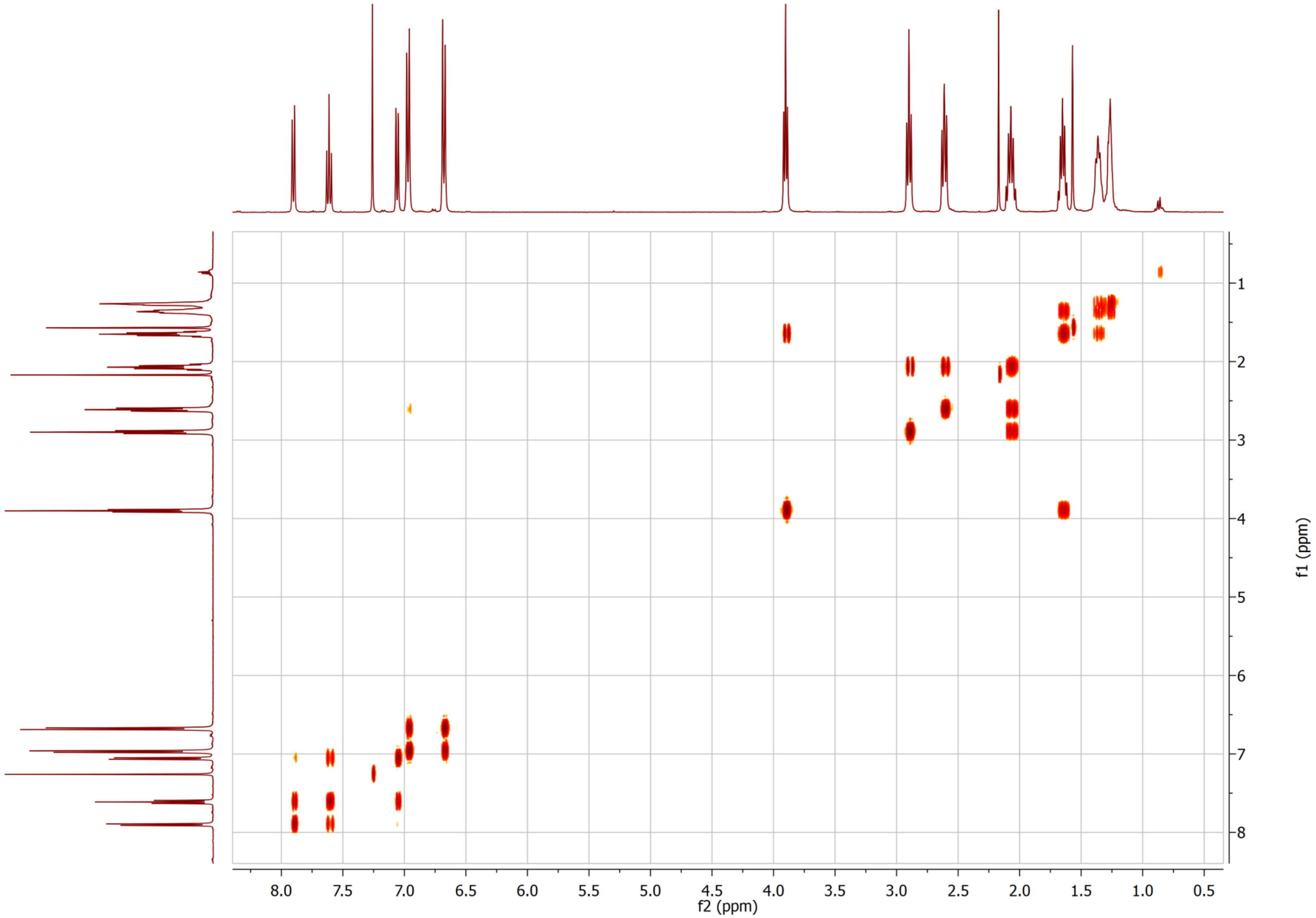

Macrocycle 9b HSQC NMR ($CDCl_3$, 400 MHz, 300 K)

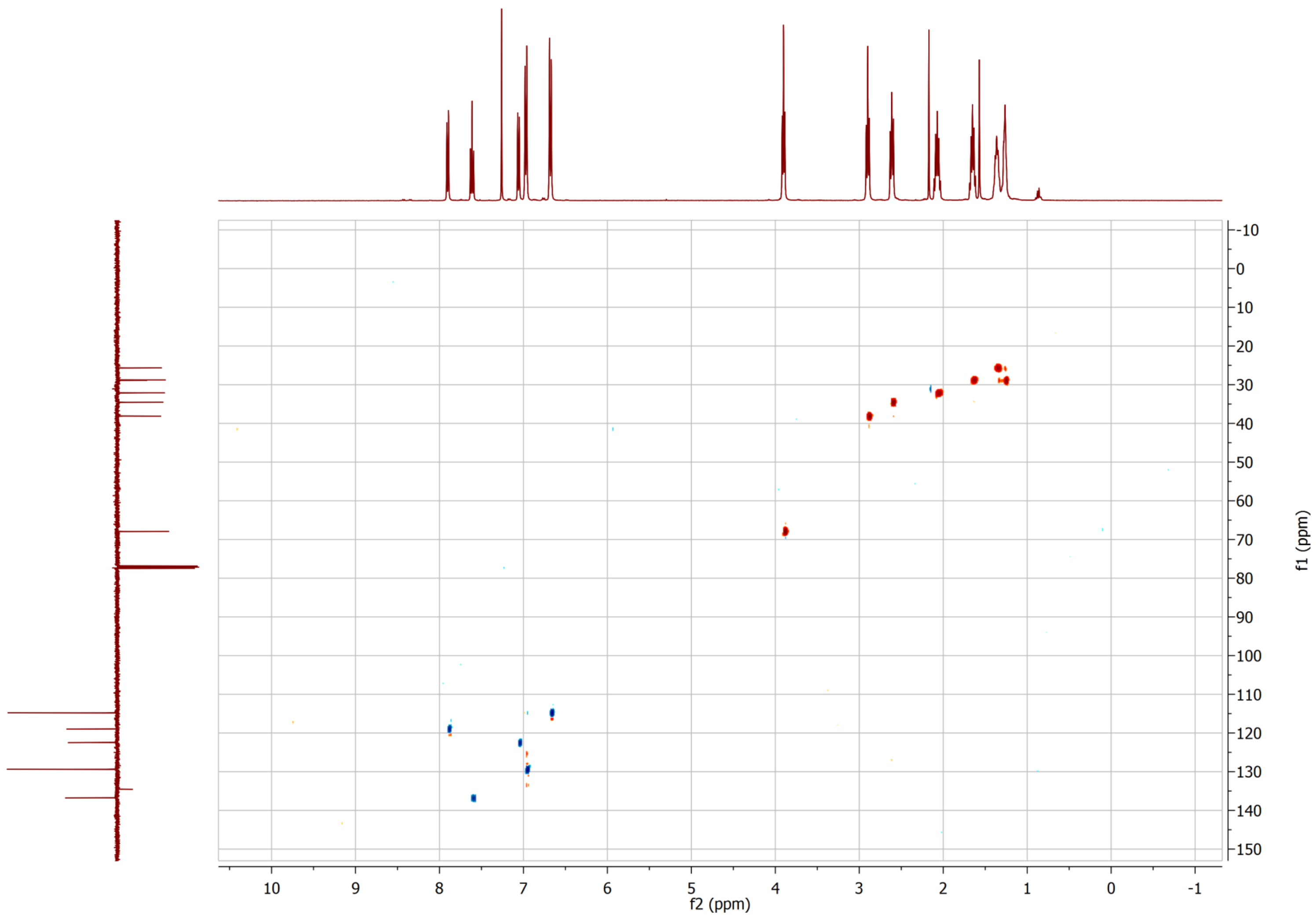

Macrocycle 9b HMBC NMR ($CDCl_3$, 400 MHz, 300 K)

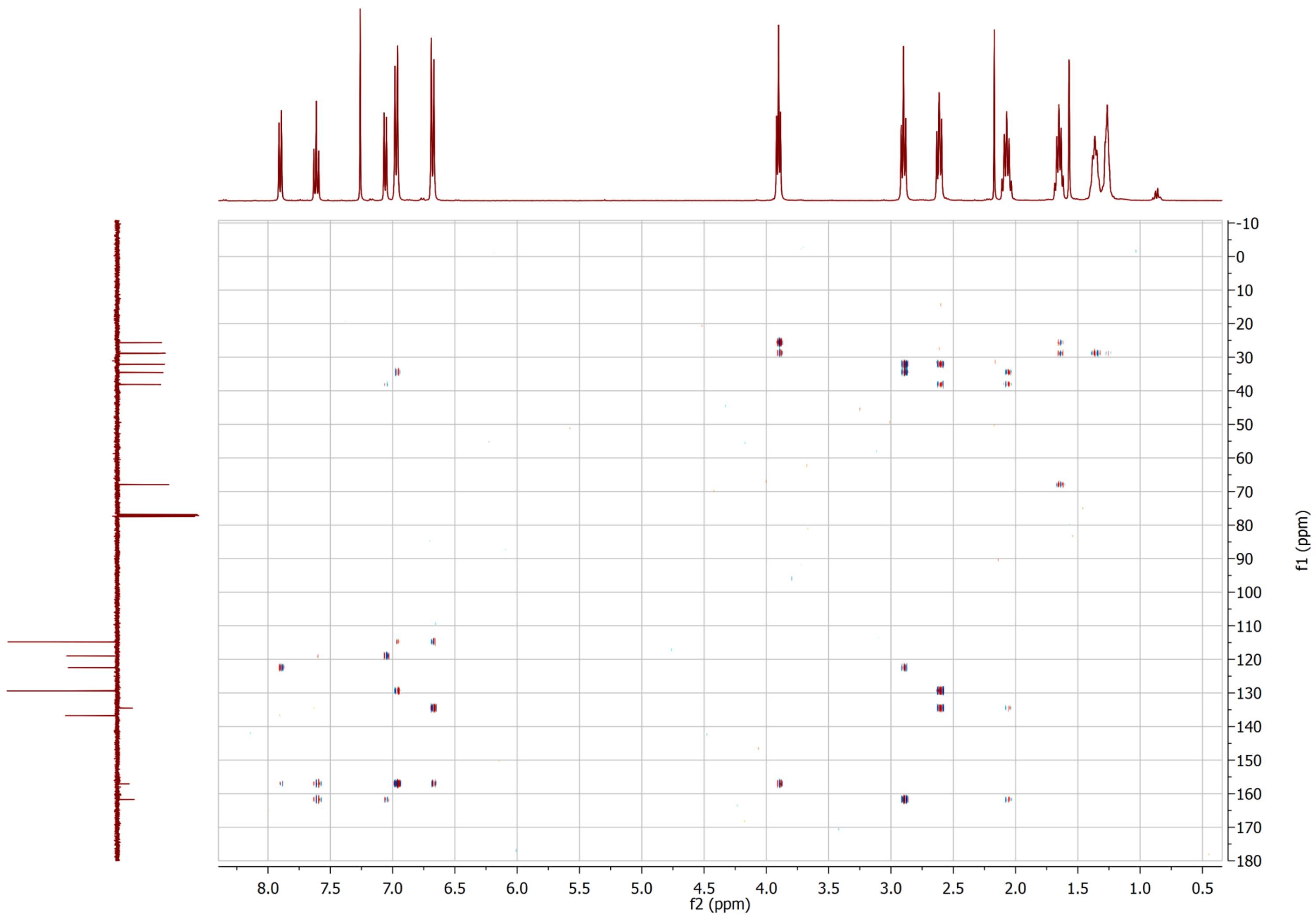

Macrocycle 9c ^{1}H NMR ($CDCl_3$, 400 MHz, 300 K)

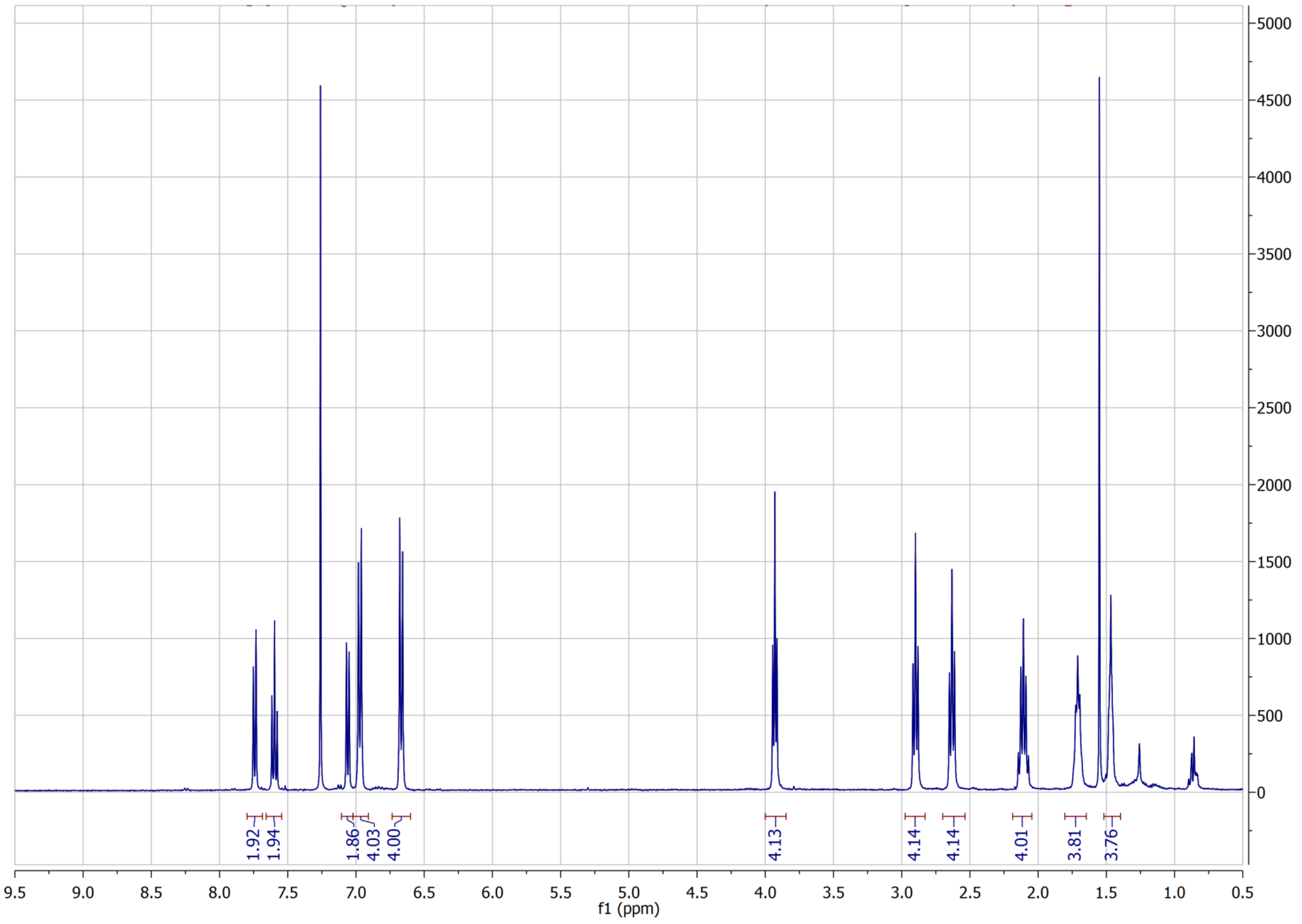

Macrocycle 9c DEPTQ135 NMR ($CDCl_3$, 100 MHz, 300 K)

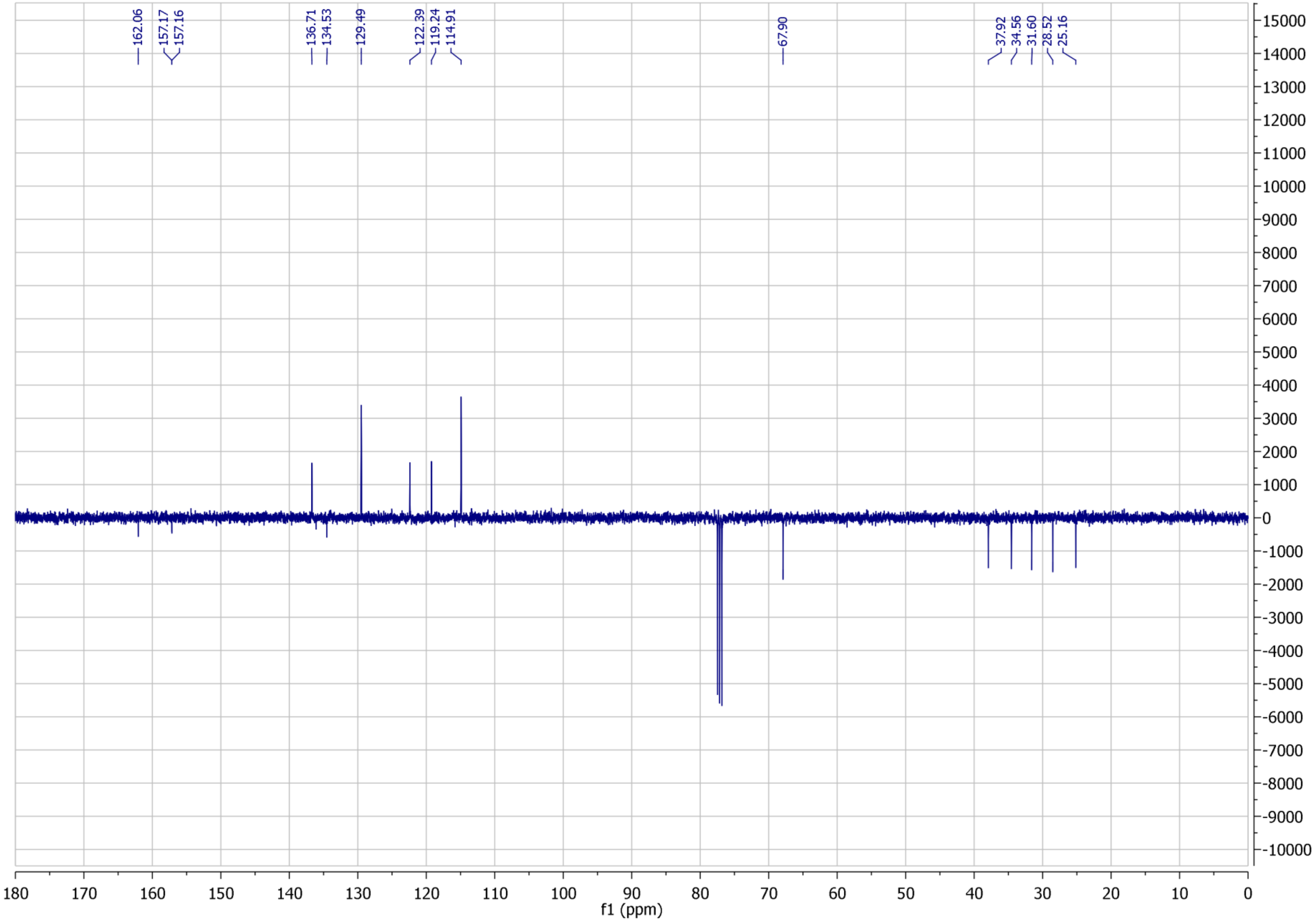

Macrocycle 9c COSY ($CDCl_3$, 400 MHz, 300 K)

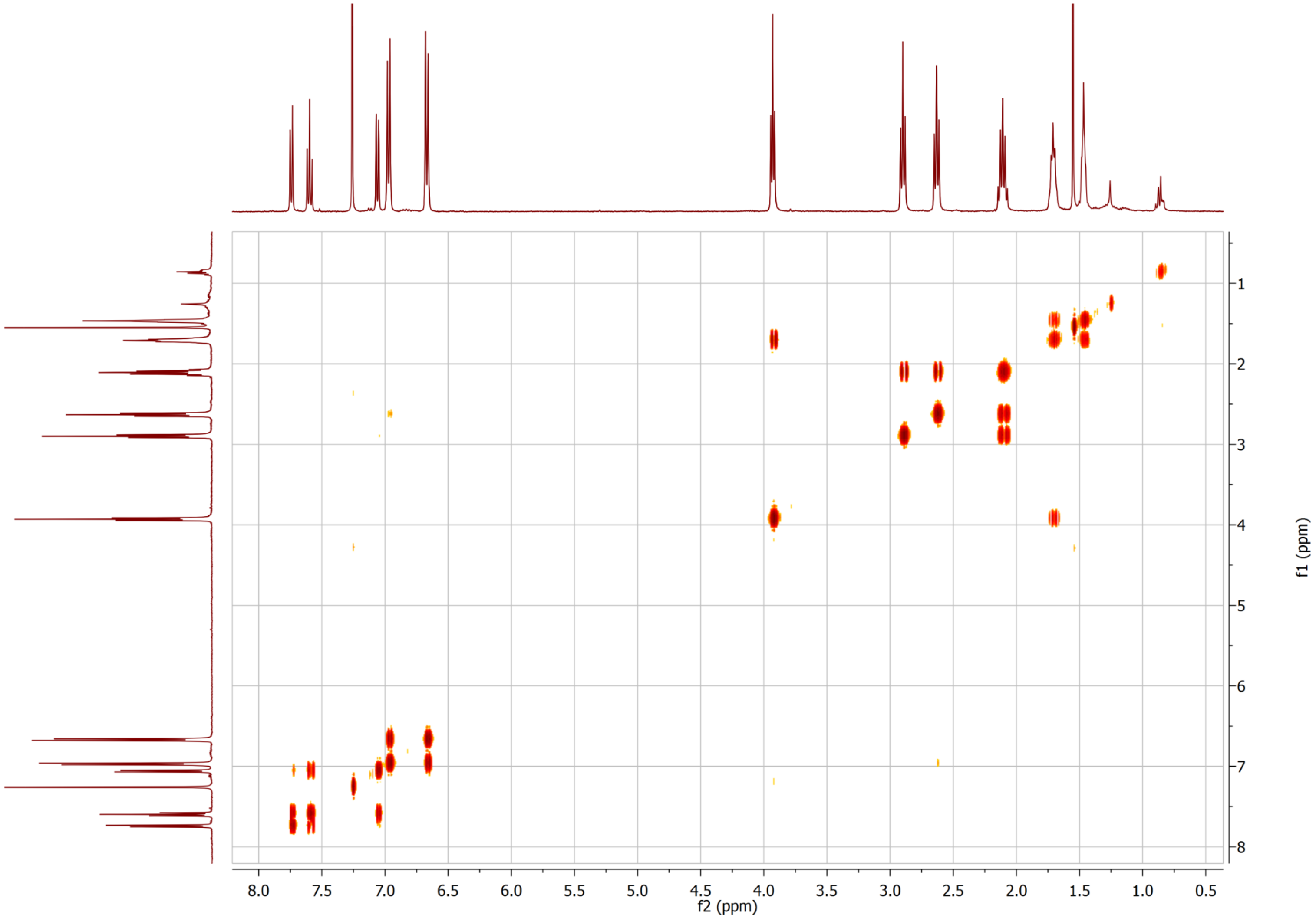

Macrocycle 9c HSQC NMR ($CDCl_3$, 400 MHz, 300 K)

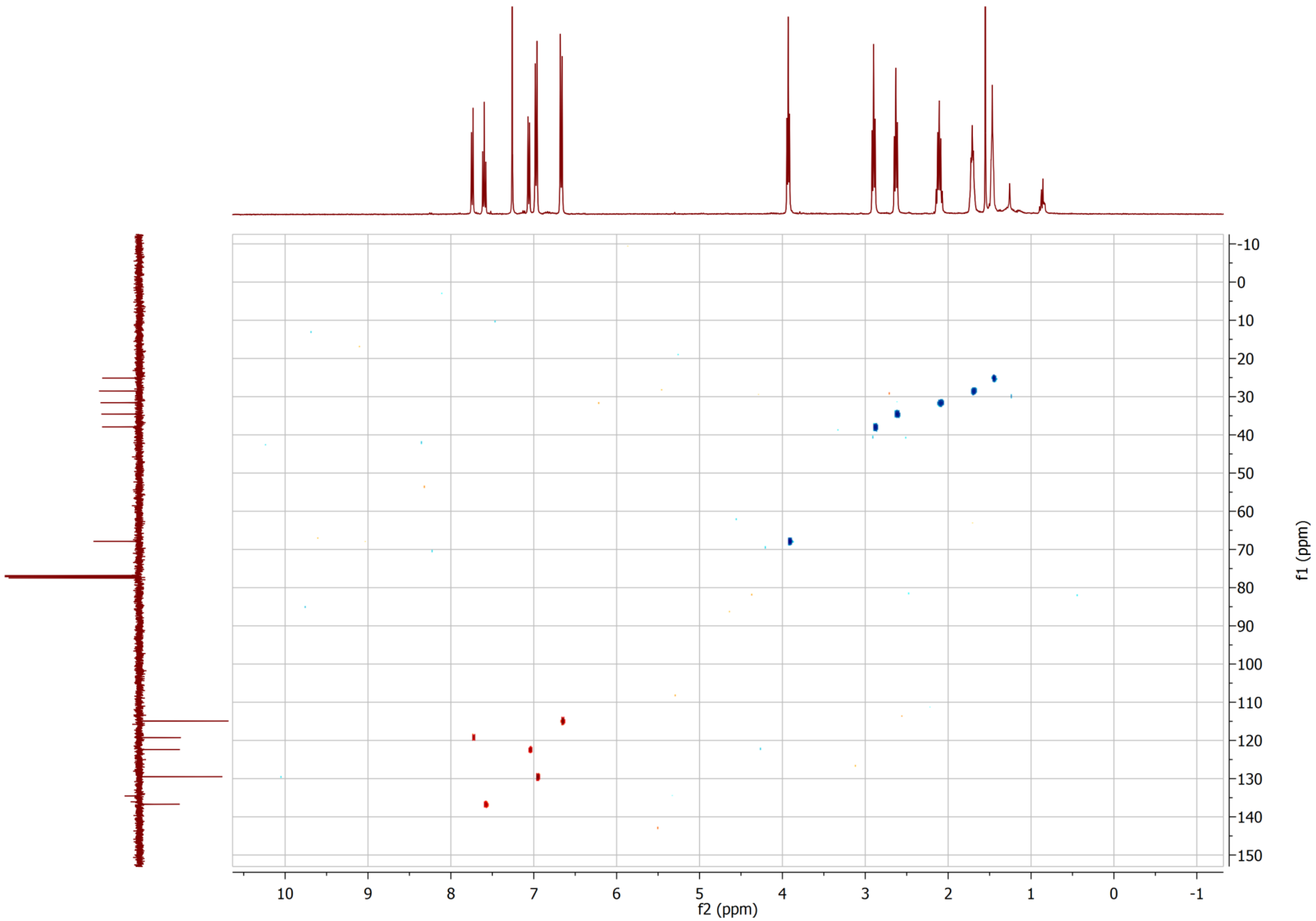

Macrocycle 9c HMBC NMR ($CDCl_3$, 400 MHz, 300 K)

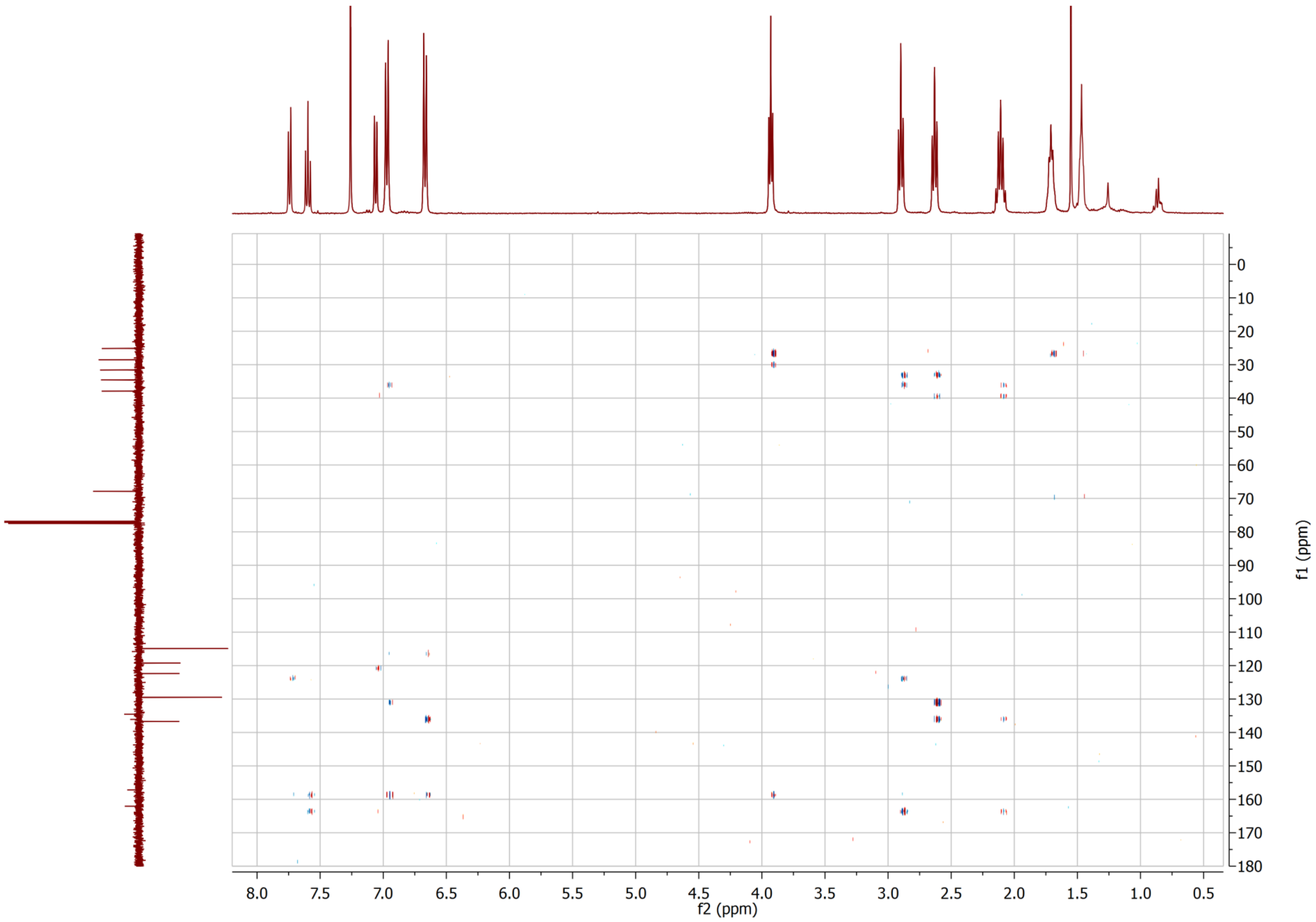

[2]Rotaxane 10a ^{1}H NMR ($CDCl_3$, 400 MHz, 300 K)

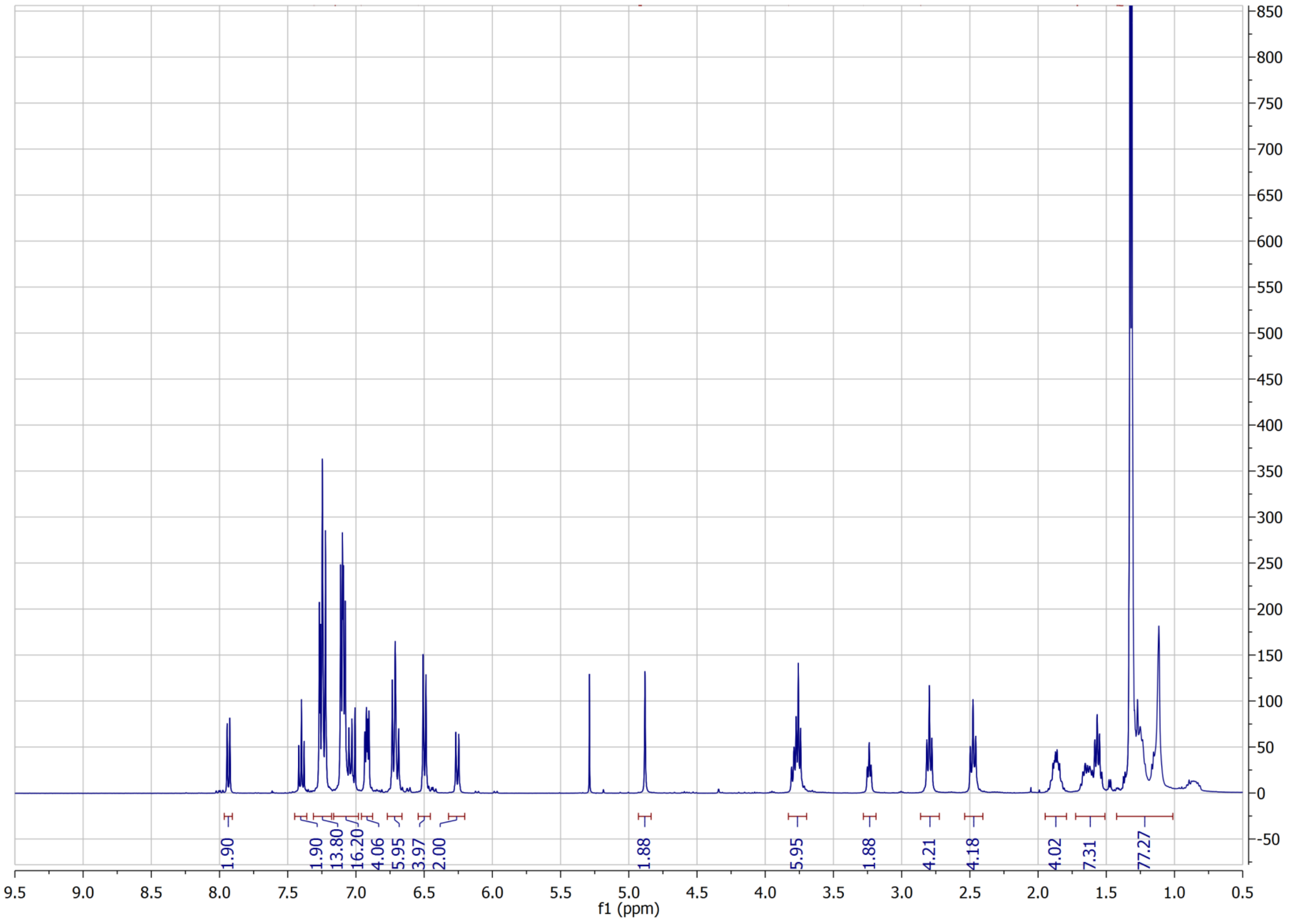

[2]Rotaxane 10a DEPTQ135 NMR ($CDCl_3$, 100 MHz, 300 K)

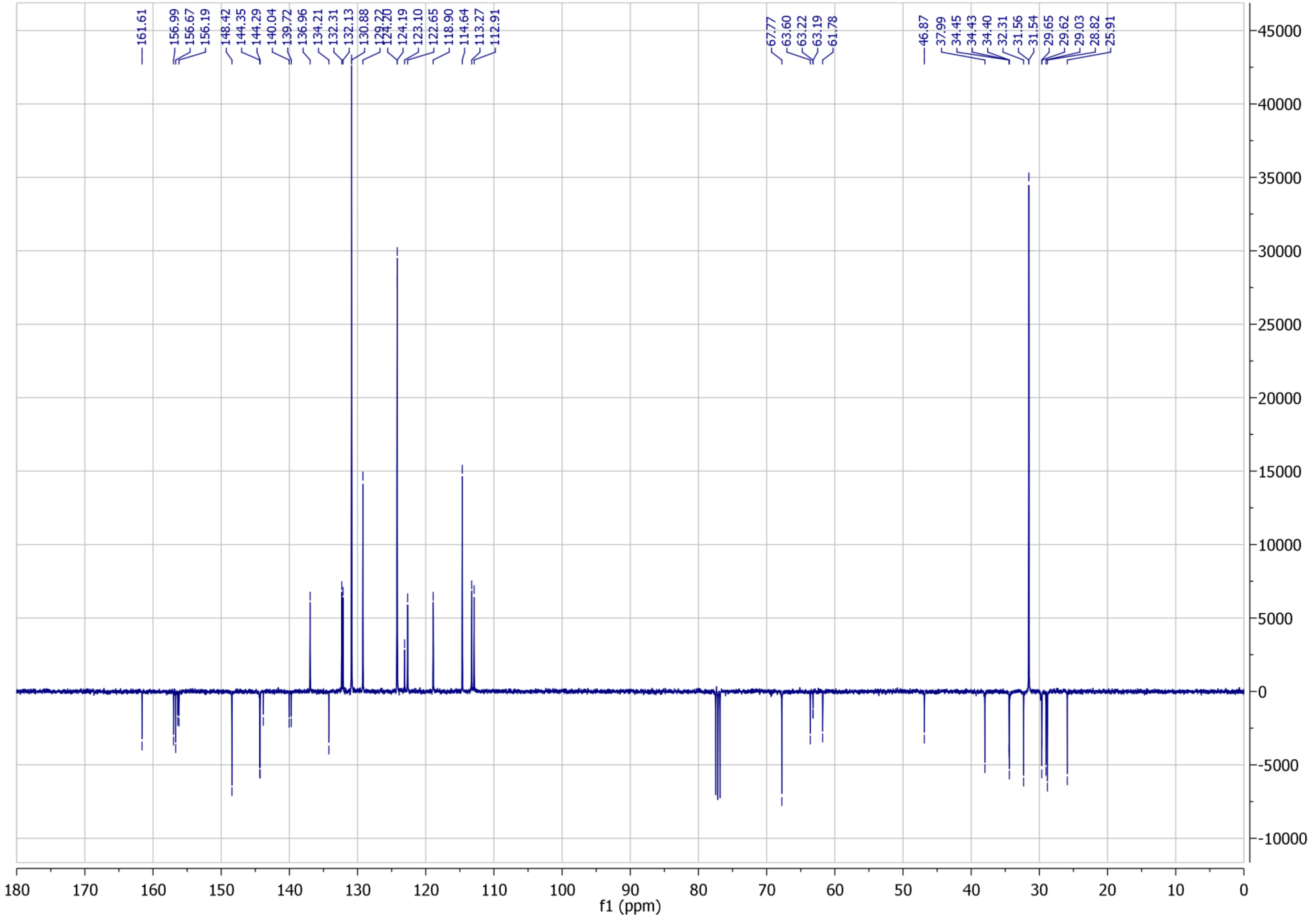

[2]Rotaxane 10a COSY ($CDCl_3$, 600 MHz, 300 K)

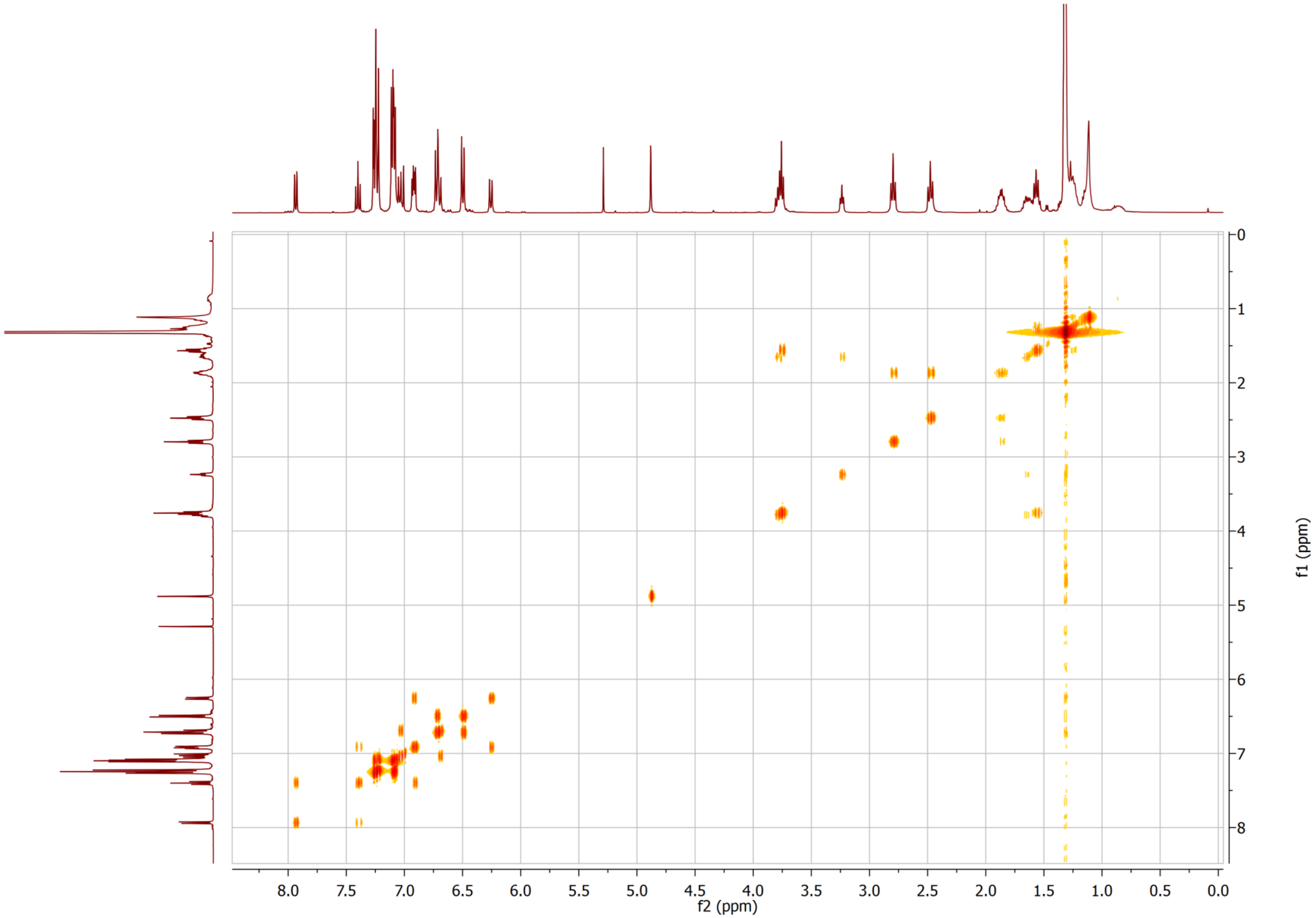

[2]Rotaxane 10a HSQC NMR ($CDCl_3$, 600 MHz, 300 K)

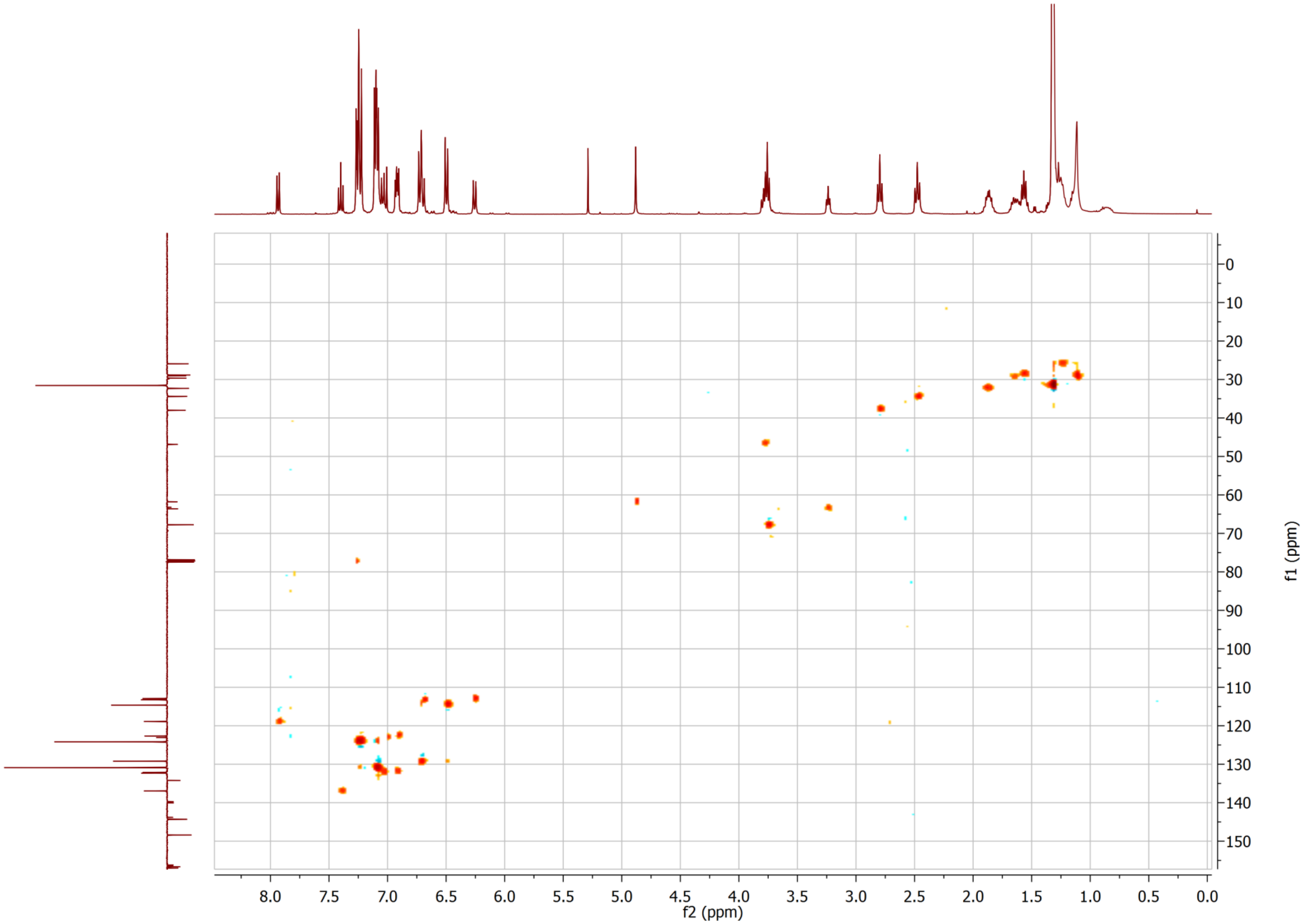

[2]Rotaxane 10a HMBC NMR ($CDCl_3$, 600 MHz, 300 K)

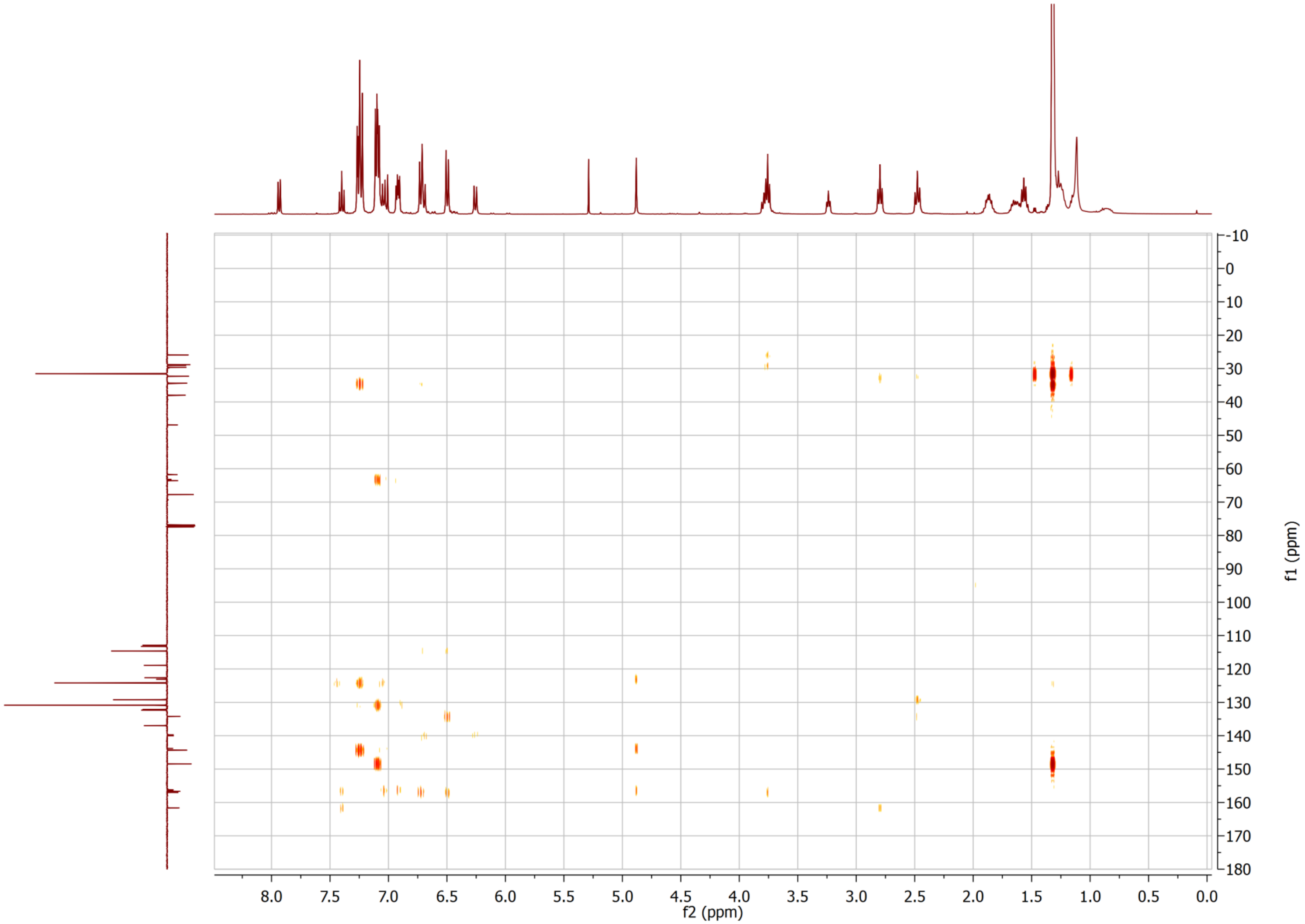

[2]Rotaxane 10b ^{1}H NMR ($CDCl_3$, 400 MHz, 300 K)

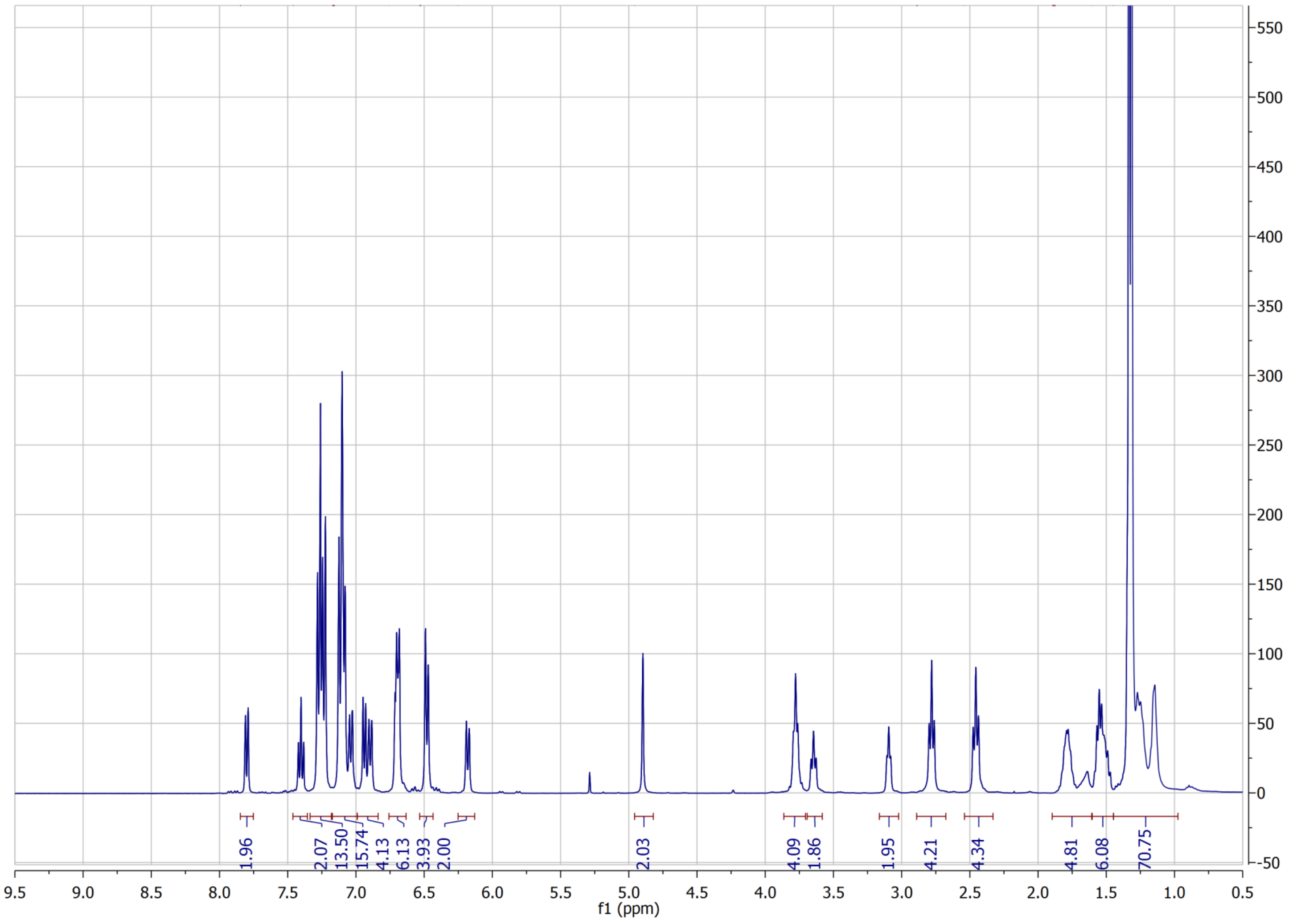

[2]Rotaxane 10b DEPTQ135 NMR ($CDCl_3$, 100 MHz, 300 K)

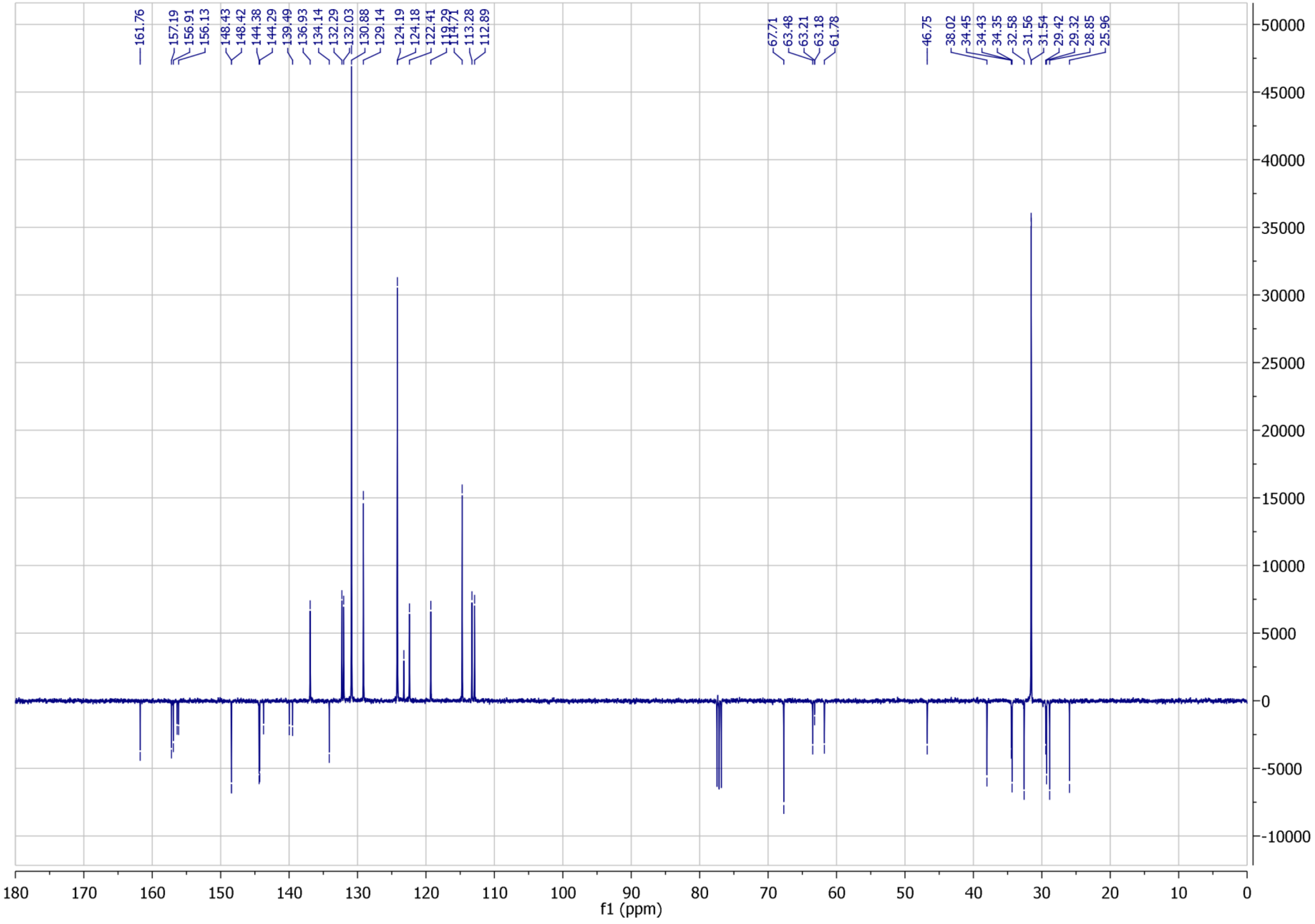

[2]Rotaxane 10b COSY ($CDCl_3$, 600 MHz, 300 K)

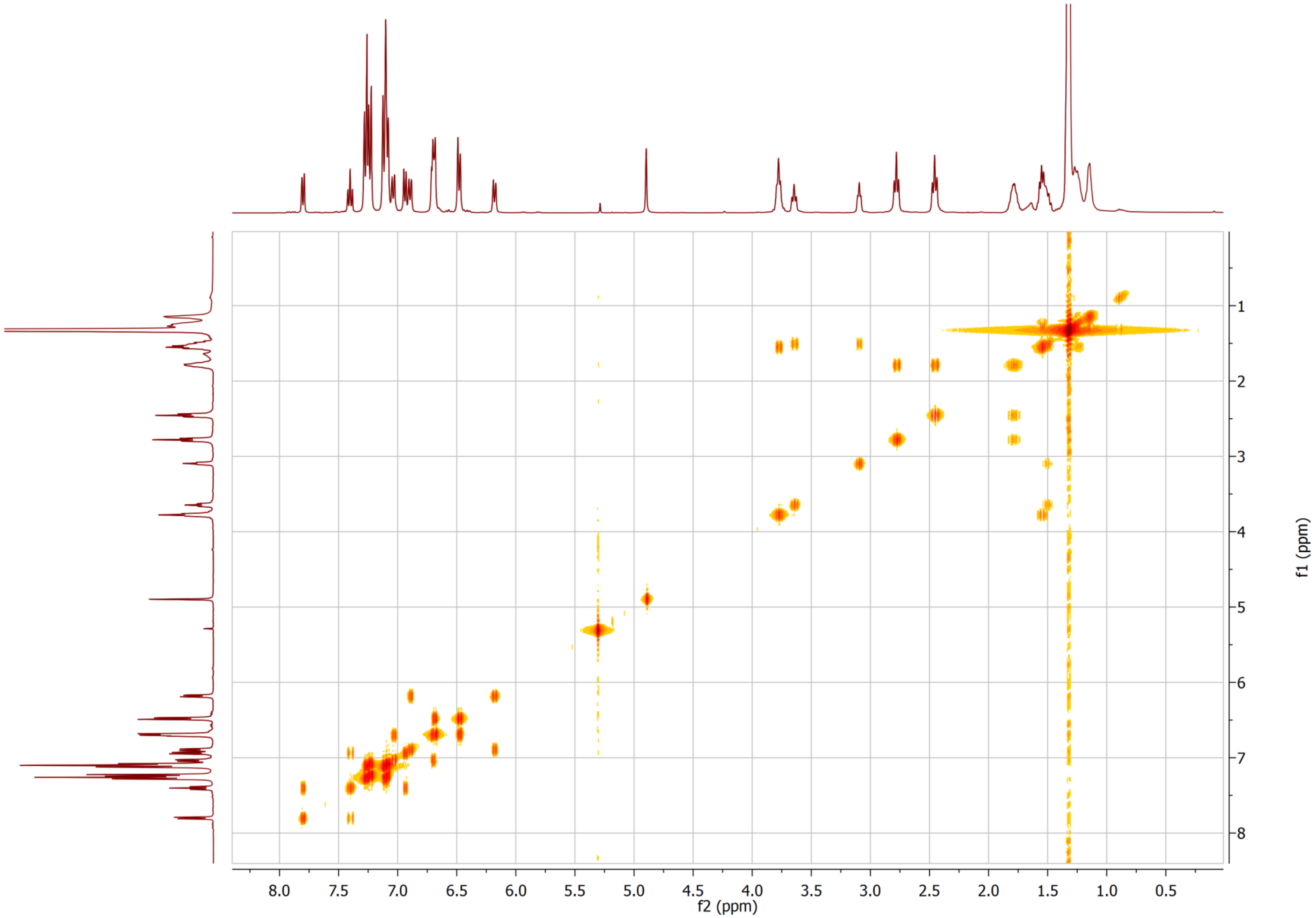

[2]Rotaxane 10b HSQC NMR ($CDCl_3$, 600 MHz, 300 K)

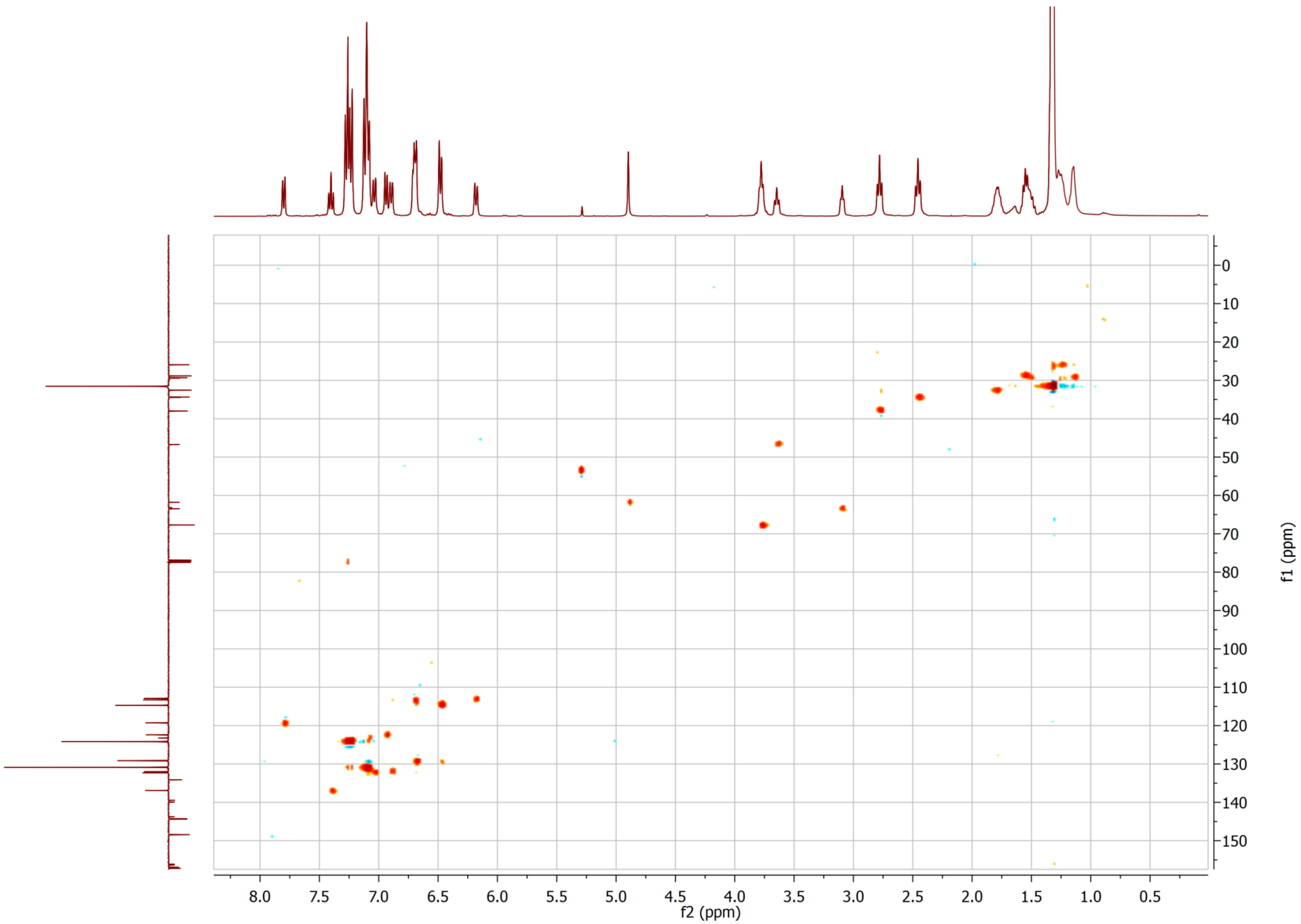

[2]Rotaxane 10b HMBC NMR ($CDCl_3$, 600 MHz, 300 K)

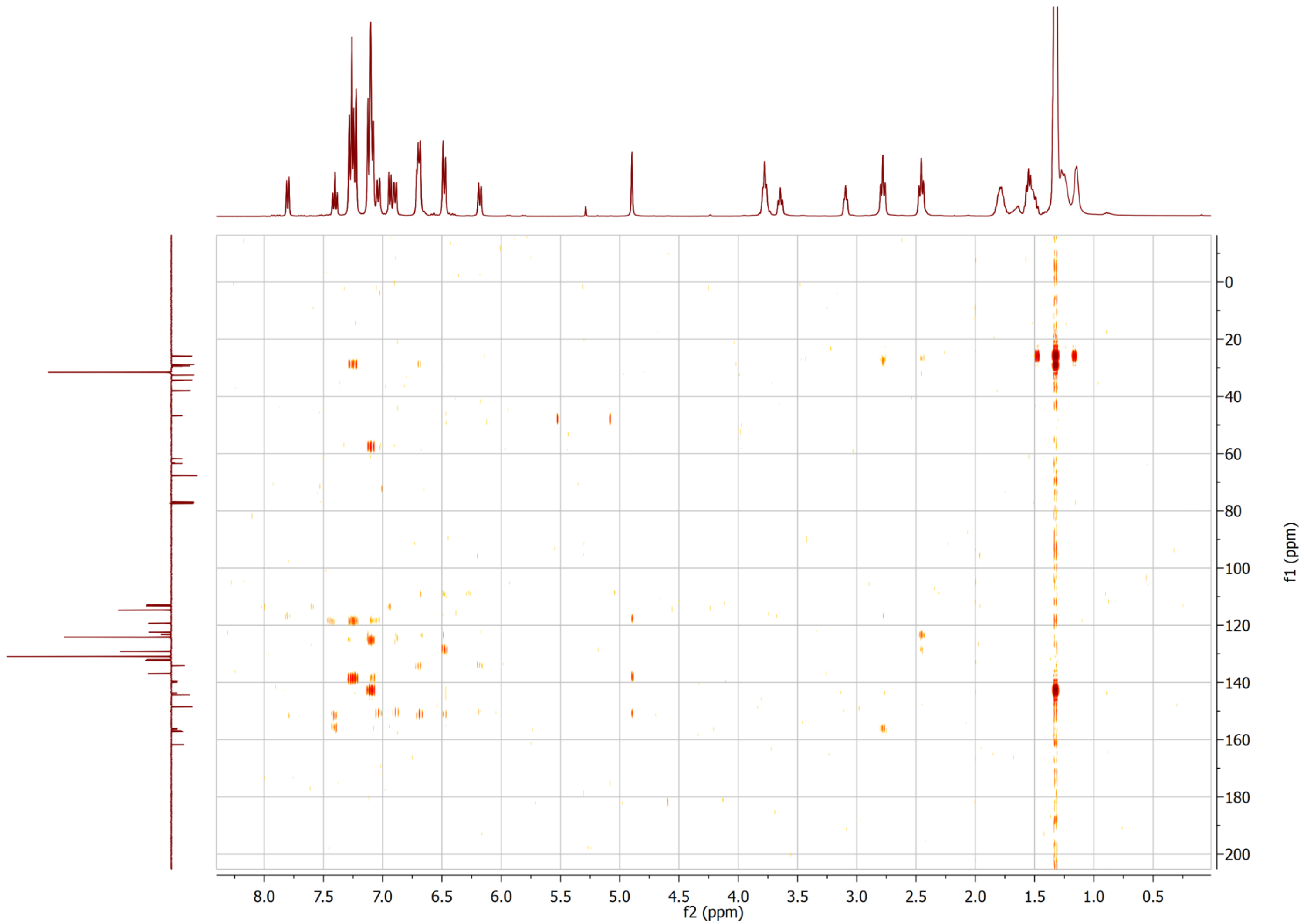

[2]Rotaxane 10c ^1H NMR ($CDCl_3$, 400 MHz, 300 K)

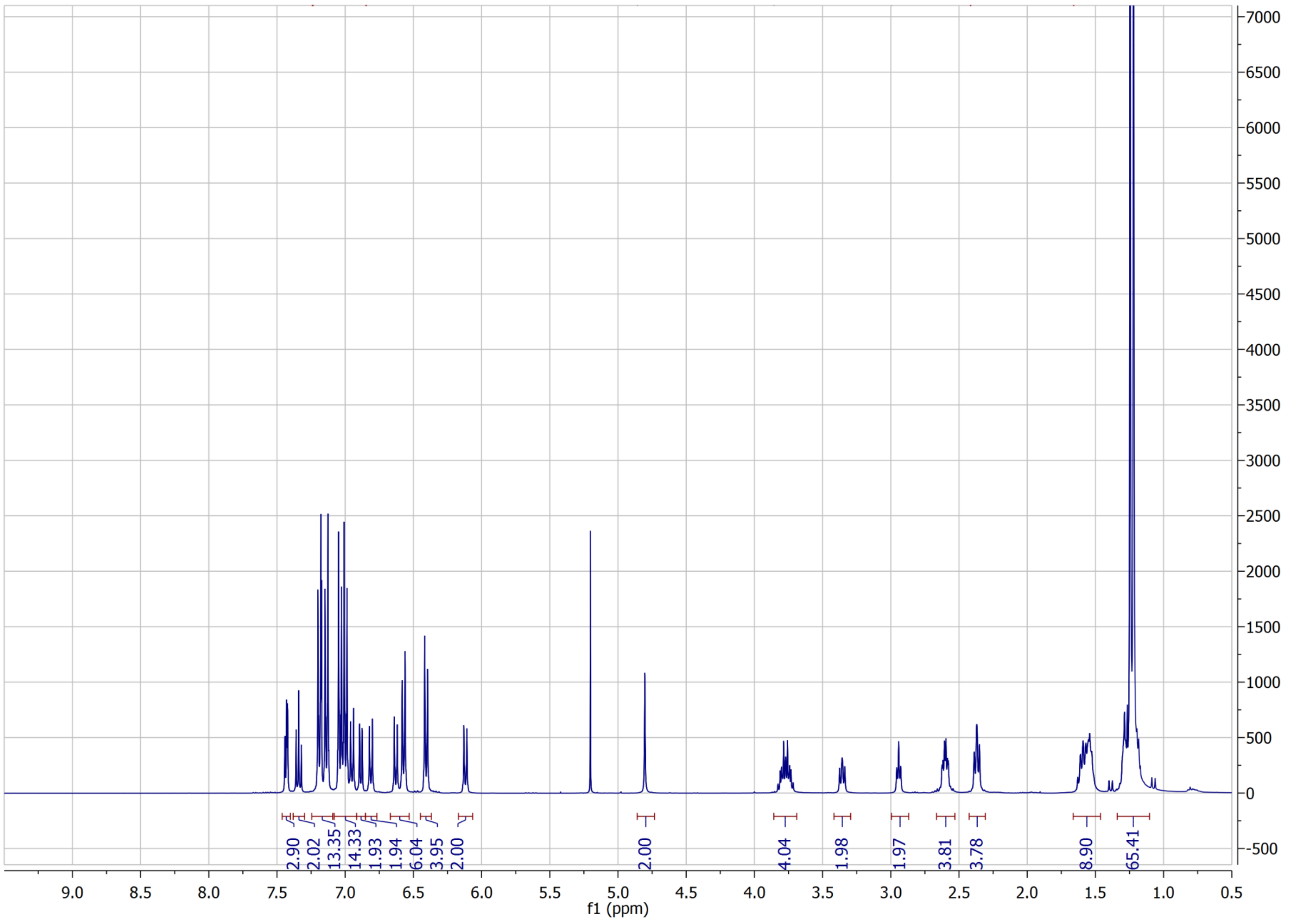

[2]Rotaxane 10c DEPTQ135 NMR ($CDCl_3$, 100 MHz, 300 K)

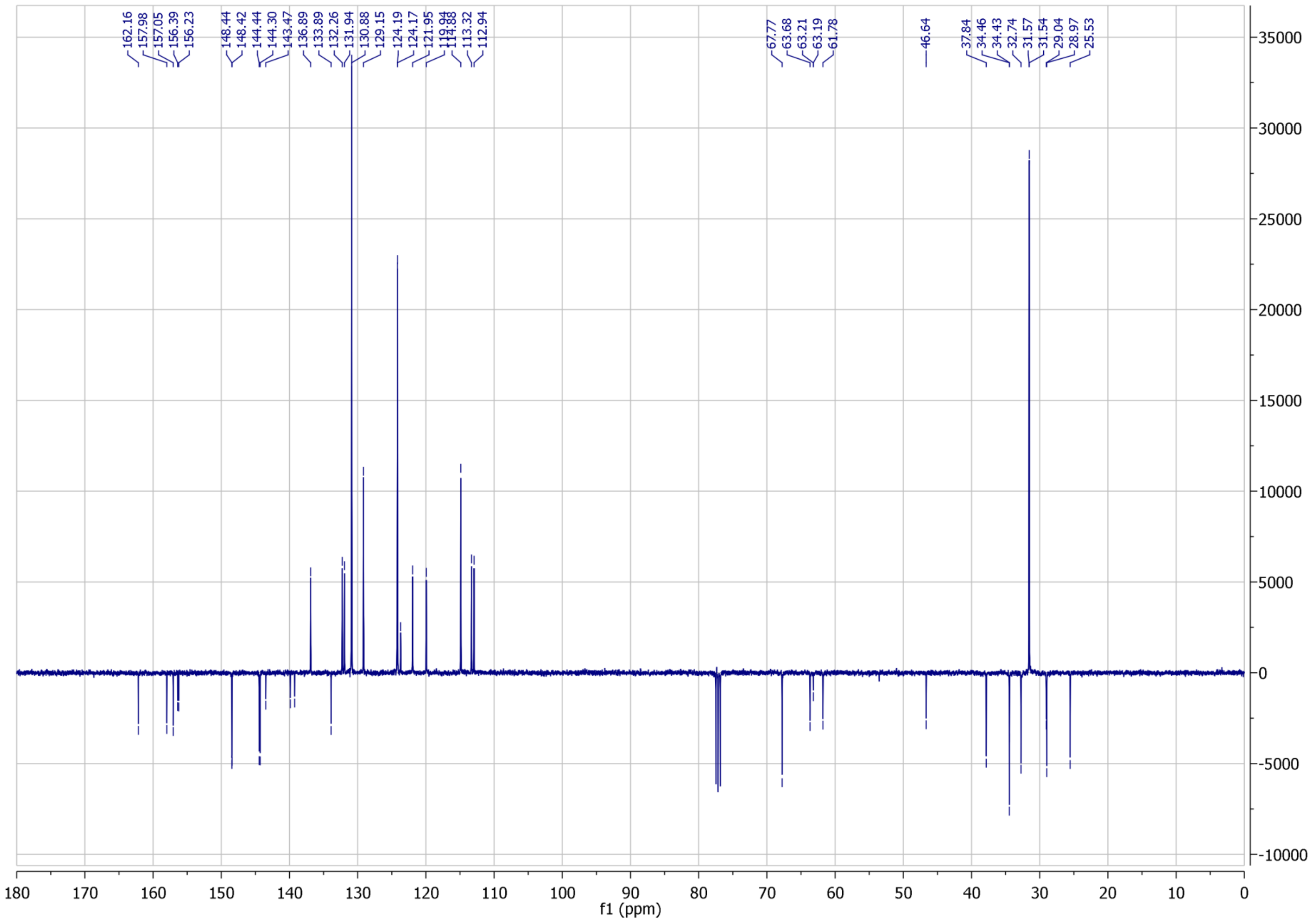

[2]Rotaxane 10c COSY ($CDCl_3$, 400 MHz, 300 K)

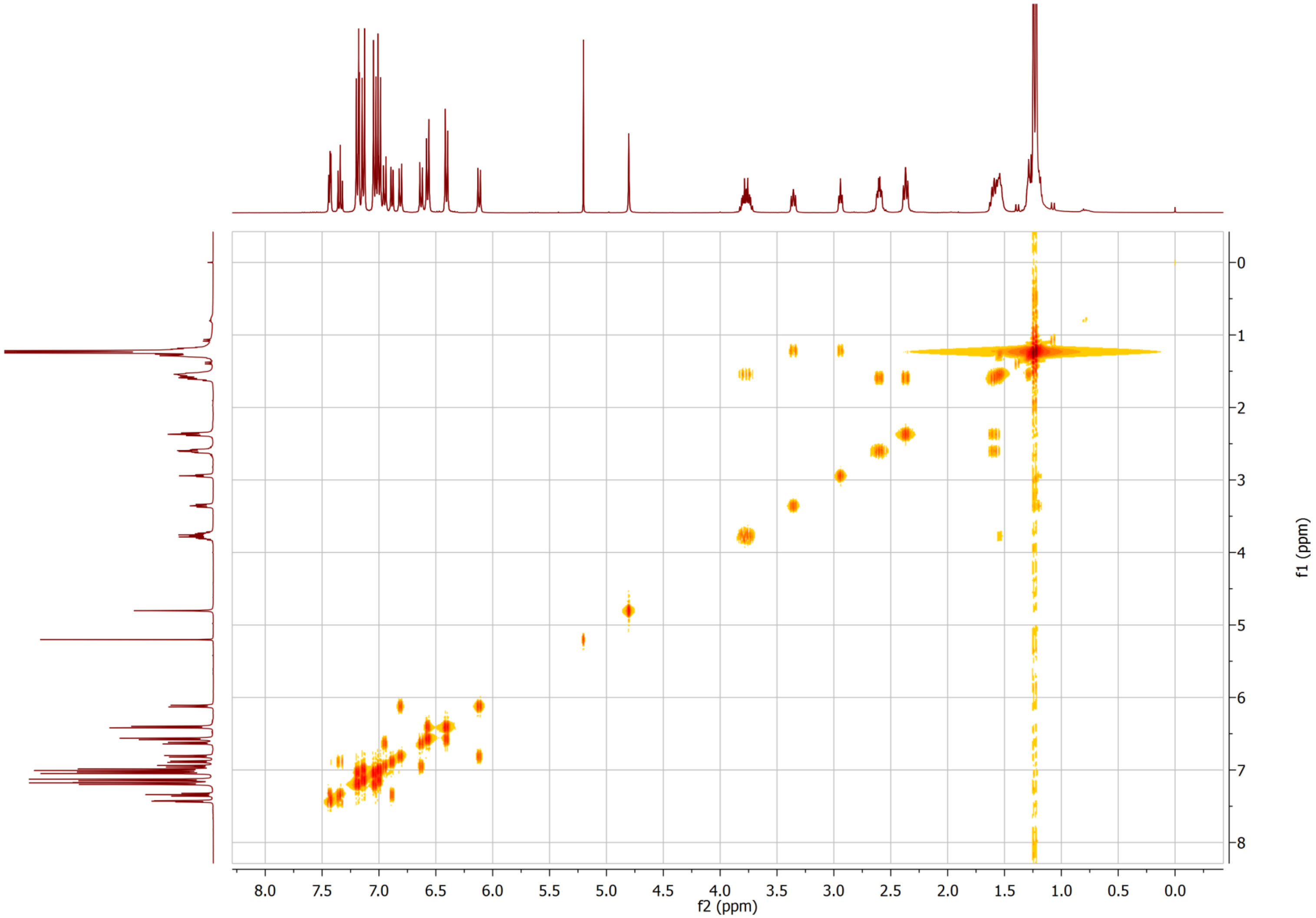

[2]Rotaxane 10c HSQC NMR ($CDCl_3$, 400 MHz, 300 K)

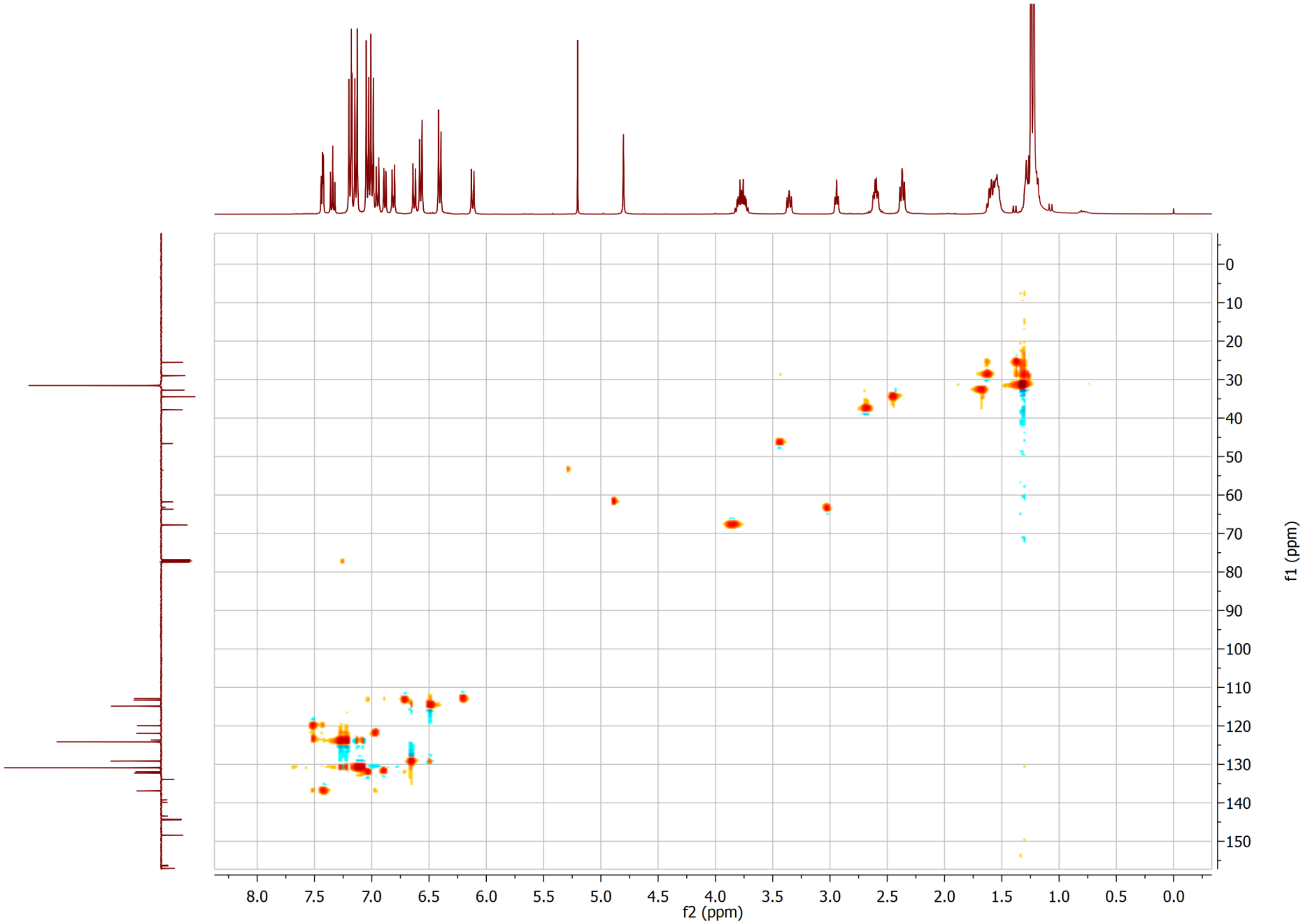

[2]Rotaxane 10c HMBC NMR ($CDCl_3$, 400 MHz, 300 K)

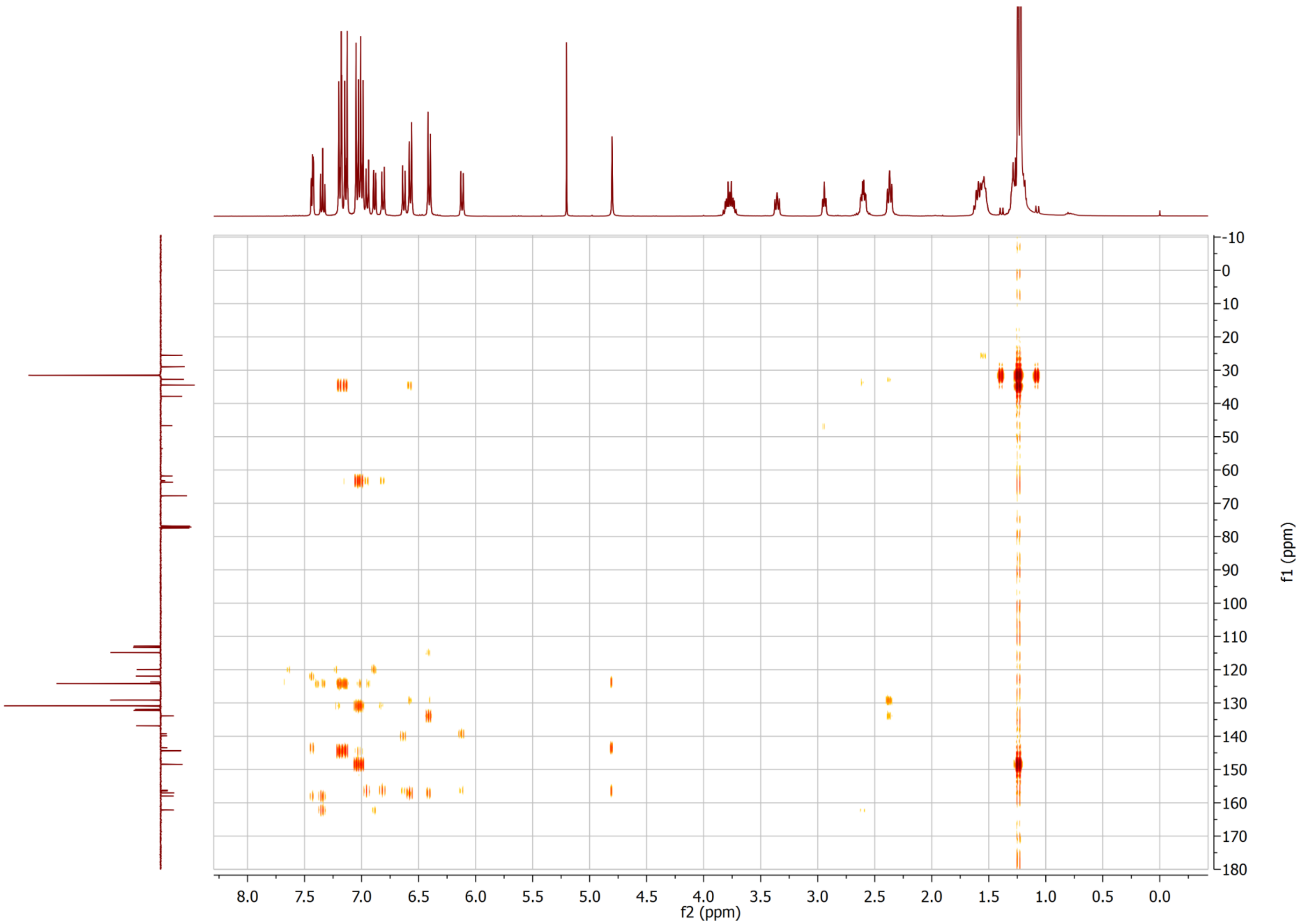

[2]Rotaxane 10d ^{1}H NMR ($CDCl_3$, 400 MHz, 300 K)

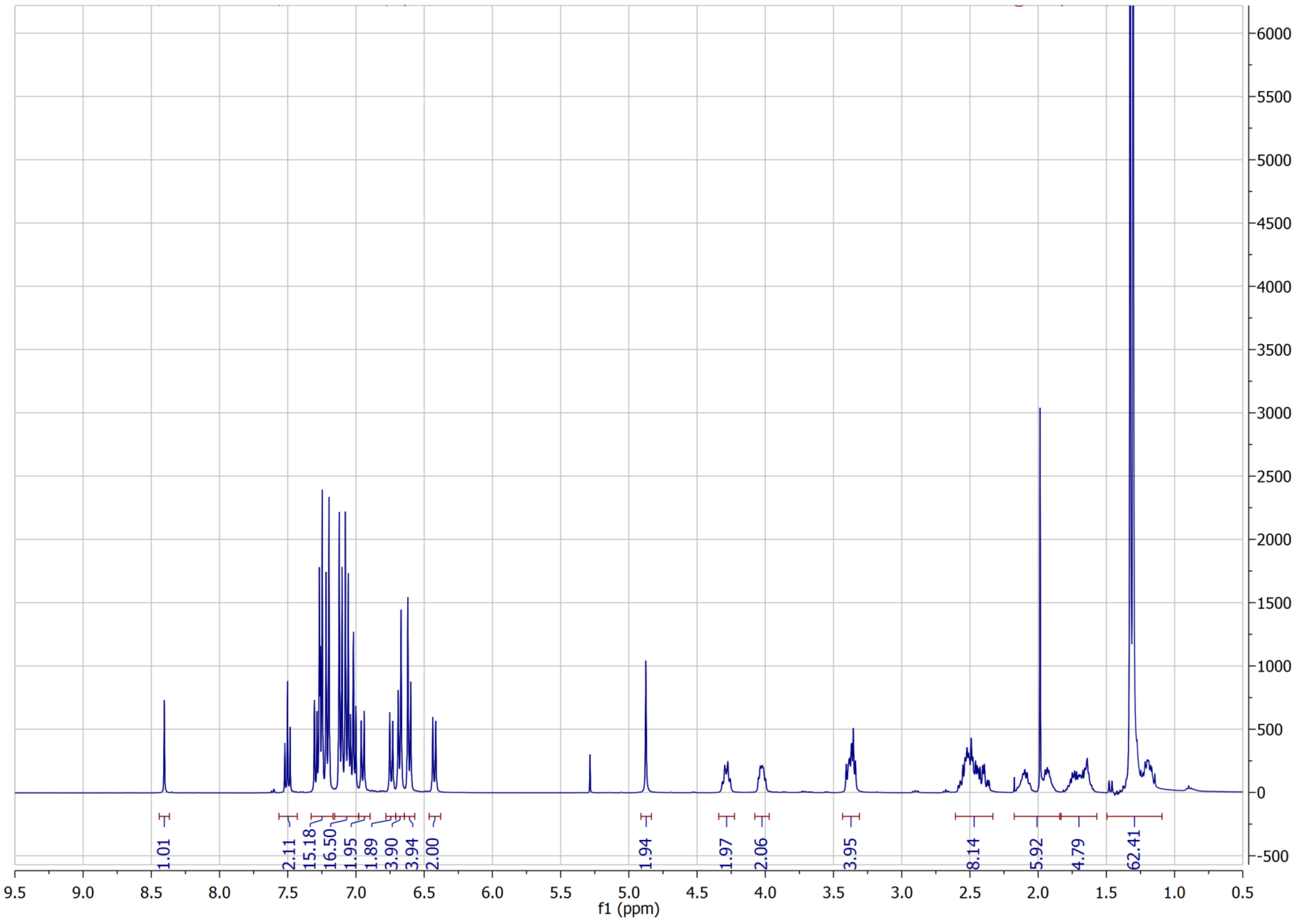

[2]Rotaxane 10d DEPTQ135 NMR ($CDCl_3$, 100 MHz, 300 K)

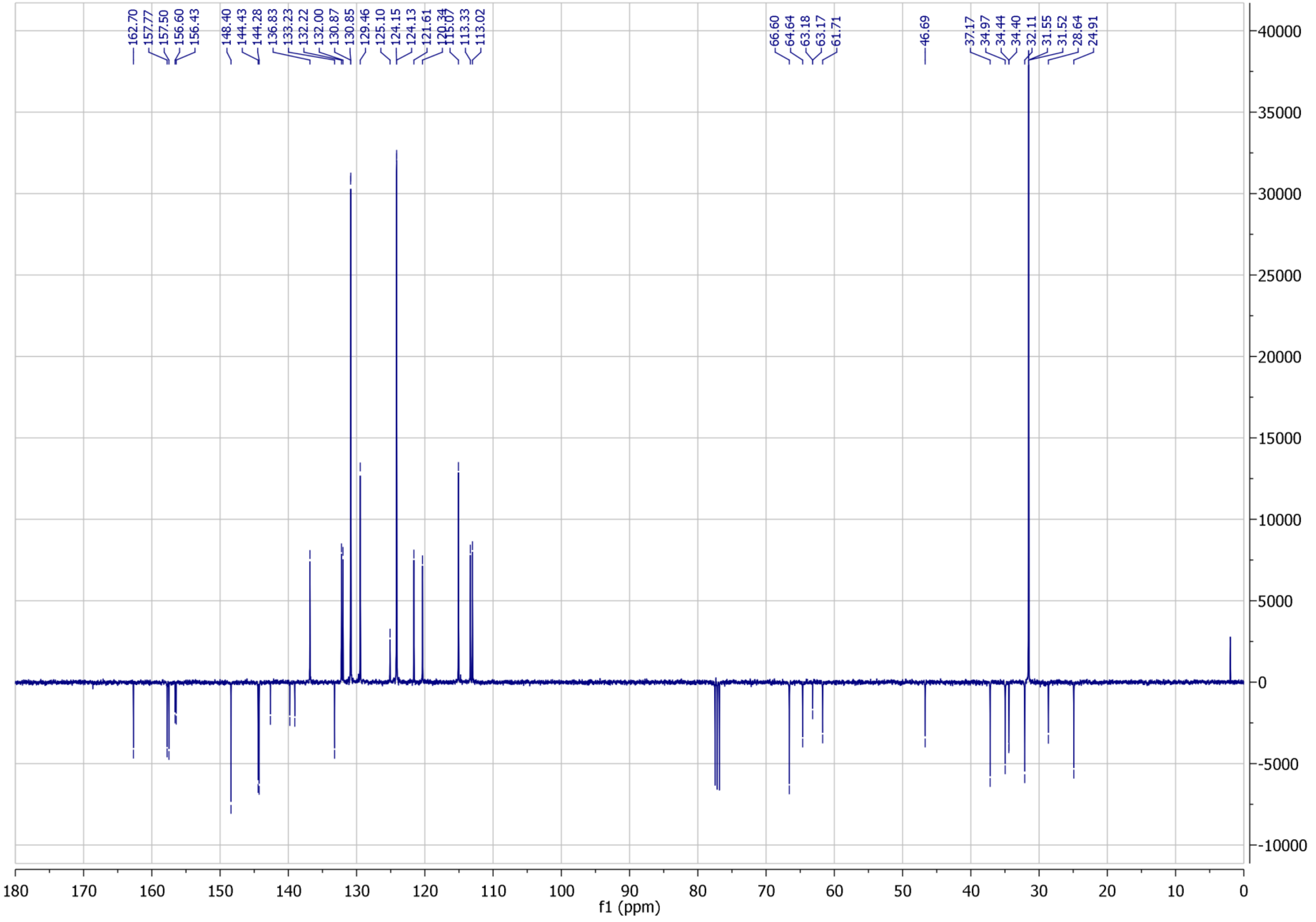

[2]Rotaxane 10d COSY ($CDCl_3$, 400 MHz, 300 K)

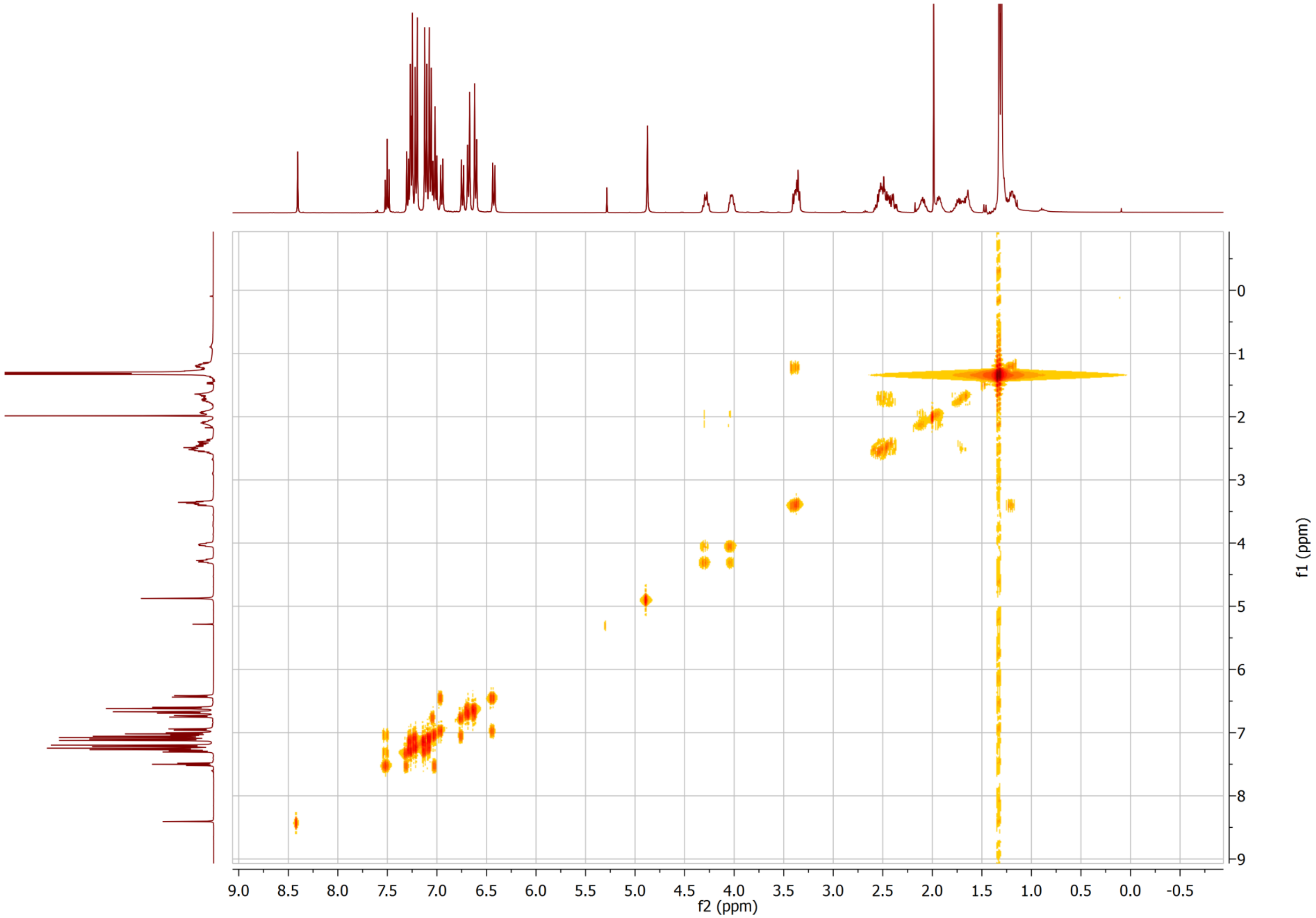

[2]Rotaxane 10d HSQC NMR ($CDCl_3$, 400 MHz, 300 K)

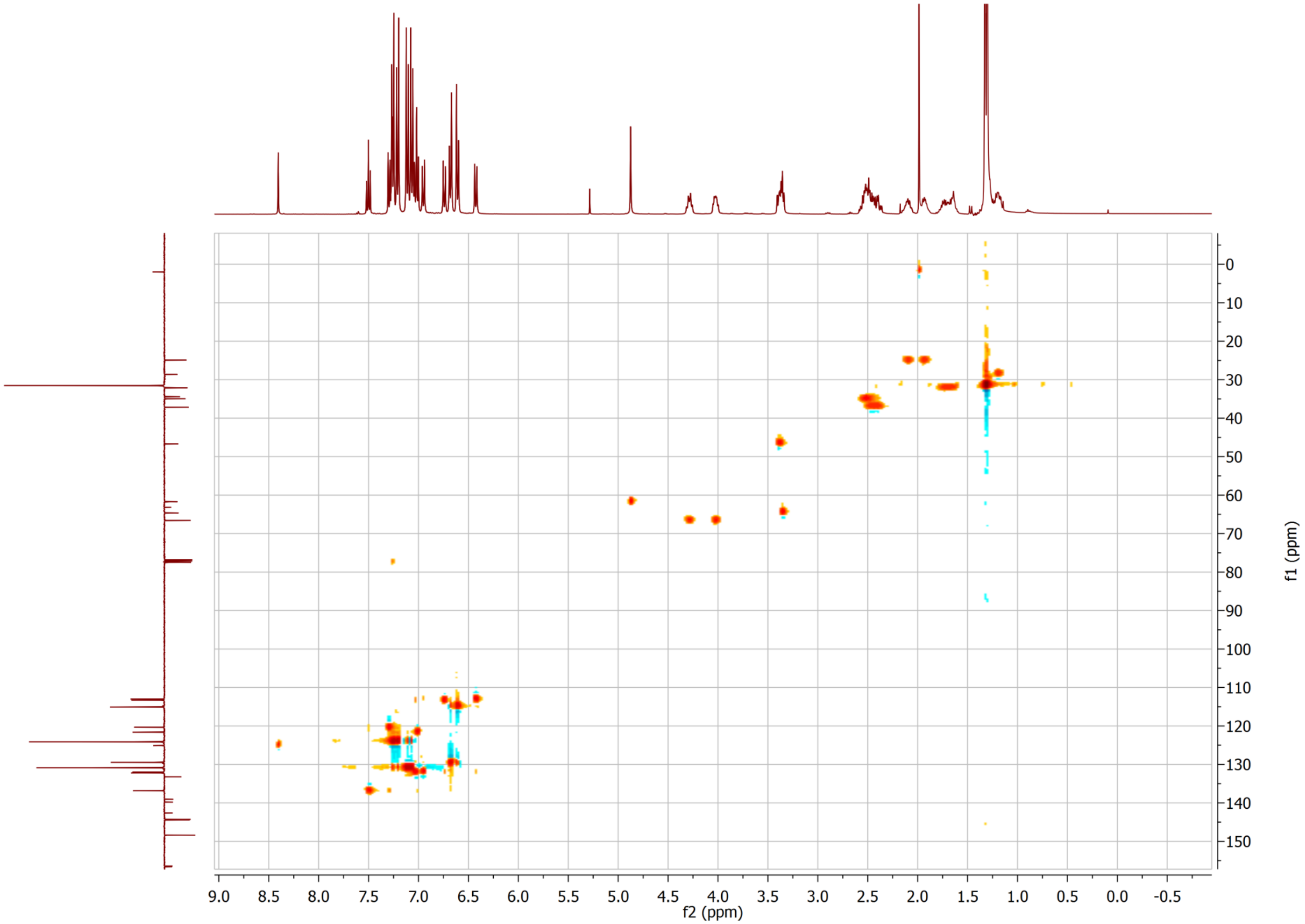

[2]Rotaxane 10d HMBC NMR ($CDCl_3$, 400 MHz, 300 K)

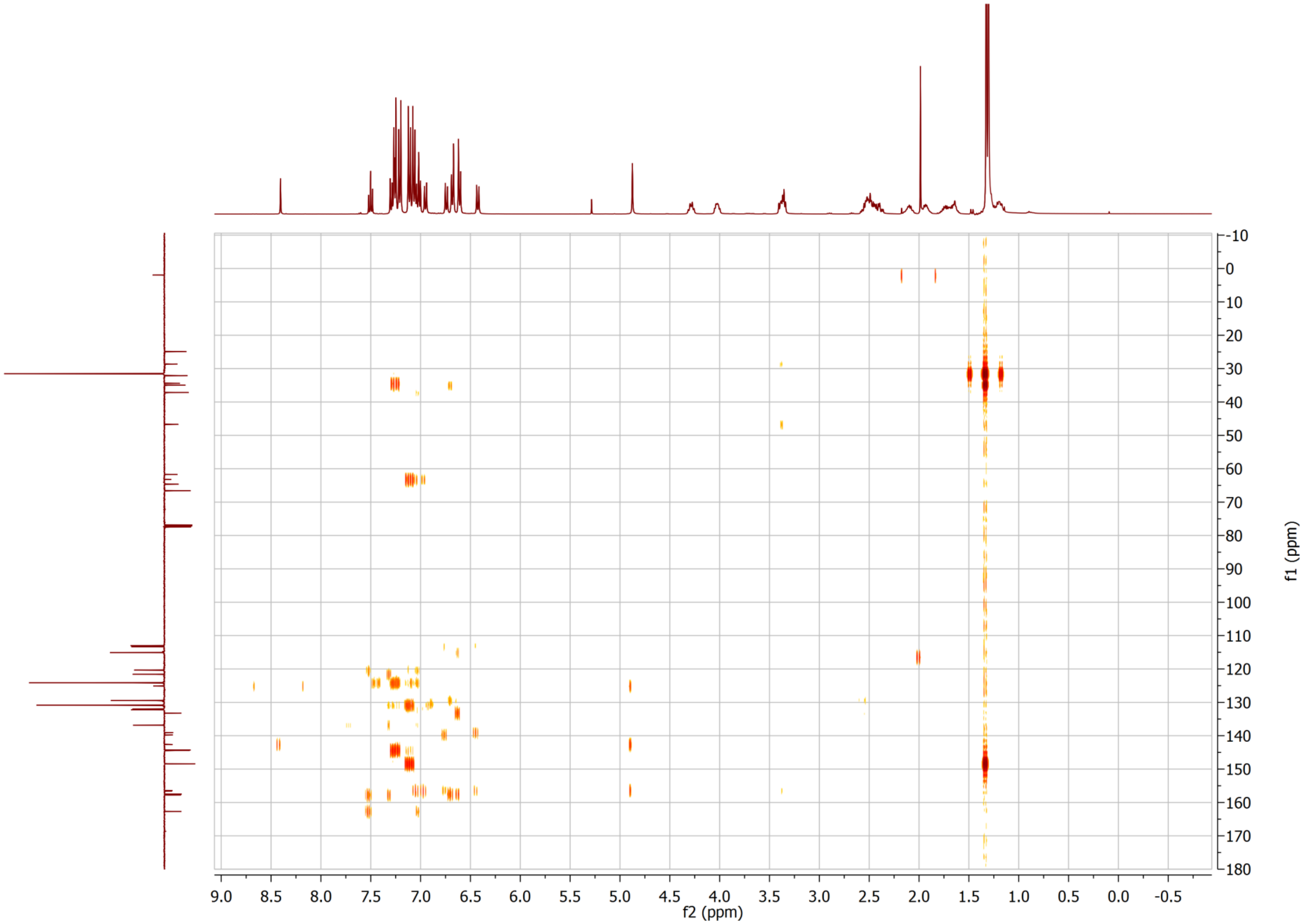

References

1. V. Aucagne, J. Berná, J. D. Crowley, S. M. Goldup, K. D. Hänni, D. A. Leigh, P. J. Lusby, V. E. Ronaldson, A. M. Z. Slawin, A. Viterisi and D. B. Walker, *J. Am. Chem. Soc.*, 2007, **129**, 11950-11963.
2. H. Lahlali, K. Jobe, M. Watkinson and S. M. Goldup, *Angew. Chem. Int. Ed.*, 2011, **50**, 4151-4155.
3. V. Aucagne, K. D. Hänni, D. A. Leigh, P. J. Lusby and D. B. Walker, *J. Am. Chem. Soc.*, 2006, **128**, 2186-2187.
4. L. M. Klivansky, G. Koshkakaryan, D. Cao and Y. Liu, *Angew. Chem. Int. Ed.*, 2009, **48**, 4185-4189.
5. H. W. Gibson, S. H. Lee, P. T. Engen, P. Lecavalier, J. Sze, Y. X. Shen and M. Bheda, *J. Org. Chem.*, 1993, **58**, 3748-3756.
6. P. M. Jackson, C. J. Moody and P. Shah, *J. Chem. Soc. Perkin Trans. 1*, 1990, 2909-2918.
7. G. W. Kabalka, M. Varma, R. S. Varma, P. C. Srivastava and F. F. Knapp, *J. Org. Chem.*, 1986, **51**, 2386-2388.
8. J. Winn, A. Pinczewska and S. M. Goldup, *J. Am. Chem. Soc.*, 2013, **135**, 13318-13321.
